# A Next‐Generation Air‐Stable Palladium(I) Dimer Enables Olefin Migration and Selective C−C Coupling in Air

**DOI:** 10.1002/anie.202009115

**Published:** 2020-09-28

**Authors:** Gourab Kundu, Theresa Sperger, Kari Rissanen, Franziska Schoenebeck

**Affiliations:** ^1^ Institute of Organic Chemistry RWTH Aachen University Landoltweg 1 52074 Aachen Germany; ^2^ Department of Chemistry Nanoscience Center University of Jyvaskyla 40014 JYU Finland

**Keywords:** homogeneous catalysis, C−C coupling, chemoselectivity, olefin migration, palladium

## Abstract

We report a new air‐stable Pd^I^ dimer, [Pd(μ‐I)(PCy_2_^t^Bu)]_2_, which triggers *E*‐selective olefin migration to enamides and styrene derivatives in the presence of multiple functional groups and with complete tolerance of air. The same dimer also triggers extremely rapid C−C coupling (alkylation and arylation) at room temperature in a modular and triply selective fashion of aromatic C−Br, C−OTf/OFs, and C−Cl bonds in poly(pseudo)halogenated arenes, displaying superior activity over previous Pd^I^ dimer generations for substrates that bear substituents *ortho* to C−OTf.

## Graphical abstract

A novel air‐stable Pd^I^ dimer is reported that triggers *E*‐selective olefin migration to enamides and styrene derivatives in the presence of multiple functional groups and with complete tolerance of air. The same dimer also triggers extremely rapid C−C coupling (alkylation and arylation) at room temperature in a modular and triply selective fashion.

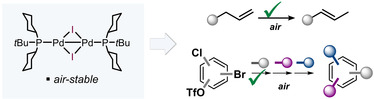

Metal catalyzed cross‐coupling reactions to forge C−C bonds^[1]^ as well as olefin migrations[^2^, ^3^] for the construction of stereochemically defined double bonds are central strategies in the synthesis of key building blocks, pharmaceuticals, materials as well as of societal and industrial relevance, for example, in the production of commodity chemicals, such as gasoline, nylon, detergents, cosmetics, fragrances or food additives.^[4]^ A central challenge in this context is the control of selectivity: whereas the site‐selective C−C bond formation of poly(pseudo)halogenated arenes is a powerful strategy to densely functionalized arenes,^[5]^ the positional as well as geometrical control (*E* vs. *Z*) of an olefin is key for its properties as well as follow up transformations.[^2^, ^6^]

Pd^I^ dimers have emerged as especially powerful catalysts for these challenges.^[7]^ Their catalytic role ranges from engaging in dinuclear Pd^I^ catalysis^[8]^ to being precursors for Pd^0^ in C−C and C‐heteroatom bond formations[^7b^, ^9^] or for Pd^II^‐H in olefin isomerizations.^[10]^ Ultimately, the properties, catalytic role and efficiency are dictated by the nature of the ligand and the bridging unit in the dinuclear Pd^I^ entity. For example, the bromide‐bridged [Pd(*μ*‐Br)(P^*t*^Bu_3_)]_2_ dimer **D1**[^9g^, ^9h^, ^11^] (Figure 1) is highly labile and as such readily transformed to Pd^0^ by weak nucleophiles.^[12]^ It is also highly sensitive to oxygen or coordinating solvents,[^7b^, ^13^] and therefore does not allow cross‐coupling in air. A small modification, that is, swap of bromide to iodide in the bridge, makes the Pd^I^ dimer highly robust: iodide‐bridged dimer **D2**, that is, [Pd(*μ*‐I)(P^*t*^Bu_3_)]_2_, is a bench‐stable species and no longer an efficient pre‐catalyst to Pd^0^; it needs strong nucleophiles for its conversion to Pd^0^.^[12]^ However, nucleophiles which also function as an effective bridge in the dinuclear entity, can be incorporated into arenes via an exchange under direct dinuclear Pd^I^ catalysis.^[8]^ Moreover, **D2** triggers extremely rapid and fully selective, sequential C−C bond formations (both arylation and alkylation) of aromatic C−Br, C−OTf/OFs and C−Cl bonds under open flask conditions (Figure 1).^[14]^ Conversely, owing to its robustness, dimer **D2** is ineffective in olefin isomerization (see below), which requires an initial intramolecular palladation^[10]^ with the ligand for Pd^II^‐H generation. The more labile and air‐sensitive bromide‐bridged Pd^I^ species **D1** is therefore to date the most effective Pd^I^ dimer precursor for olefin isomerizations, as demonstrated by Gooßen.^[10]^ A less precious Ni^I^ dimer was recently shown to also engage in selective olefin migration, albeit under Ni‐radical rather than Ni‐H reactivity.^[6]^ Skrydstrup demonstrated that the in situ generated [(P^*t*^Bu_3_)_2_Pd^II^(H)(Cl)] also triggers effective olefin isomerization.^[15]^

**Figure 1:**
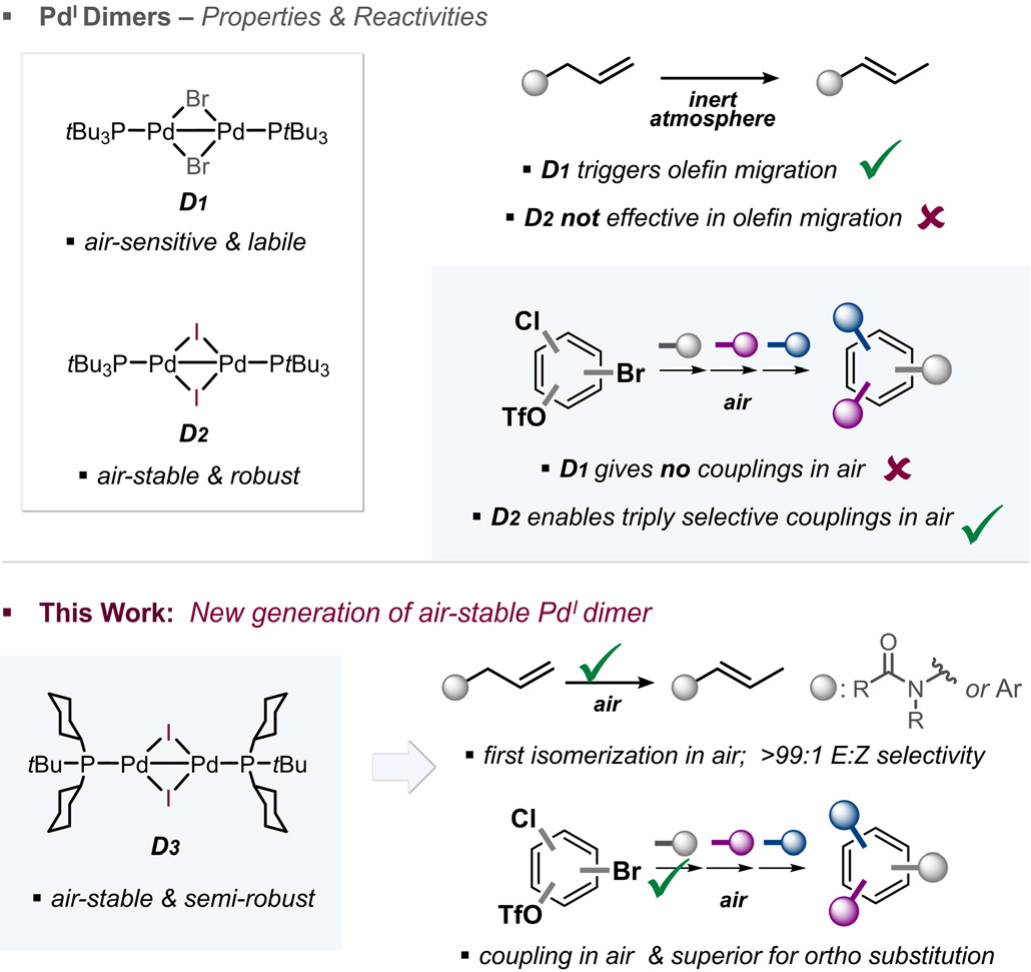
Reactivity and properties of current P^*t*^Bu_3_Pd(I) dimer generations versus this work.

As part of our ongoing program with dinuclear metal catalysts,[^7^, ^9g^, ^9h^, ^13^, ^14^] we questioned whether a hybrid version of the dimers **D1** and **D2** could potentially be created, which would combine the practical air‐stability feature of **D2** with the reactivity modes of **D1** and **D2** to ultimately enable rapid and selective cross‐coupling reactions as well as olefin migrations under complete tolerance of air.

We focused especially on the *E*‐selective generation of enamides, which owing to their exquisite reactivity and stability features make them tuneable enamine equivalents for a wide variety of transformations.^[16]^ Double bond migrations to make fully *N*‐substituted and acyclic enamides are currently largely limited to precious metals however, that is, Ru, Rh and Ir catalysis.^[17]^ Only isolated examples have been reported for other metals,^[18]^ and identification of a Pd‐based methodology would therefore be valuable, especially if paired with air‐tolerance.

We envisioned that a new generation of air‐stable Pd^I^ dimers for olefin isomerization ideally also bears the iodide bridges, which appear to be key for air stability, but then should be combined with a slightly less stabilizing ligand to also allow for intramolecular cyclopalladation and [Pd^II^‐H] generation. We wondered whether a partial replacement of *tert*‐butyl by cyclohexyl^[19]^ would be possible and potentially lead to the desired features. Such a Pd^I^ dimer is unknown. We therefore initially set out to synthesize the corresponding Pd^0^ complex, i.e. Pd(PCy_2_^*t*^Bu)_2_ which was found to have a characteristic ^31^P‐NMR spectroscopic signal at 54.1 ppm. We subsequently attempted a comproportionation of the Pd^0^ complex with Pd^II^I_2_, which resulted in a new species.^1^H, ^13^C and ^31^P NMR spectroscopic analyses (signal at 79.2 ppm in ^31^P NMR) as well as X‐ray diffraction (see Figure 2) unambiguously confirmed that the novel dimer [Pd(μ‐I)(PCy_2_^*t*^Bu)]_2_(**D3**) was generated in high yield (89 %).^[20]^

**Figure 2:**
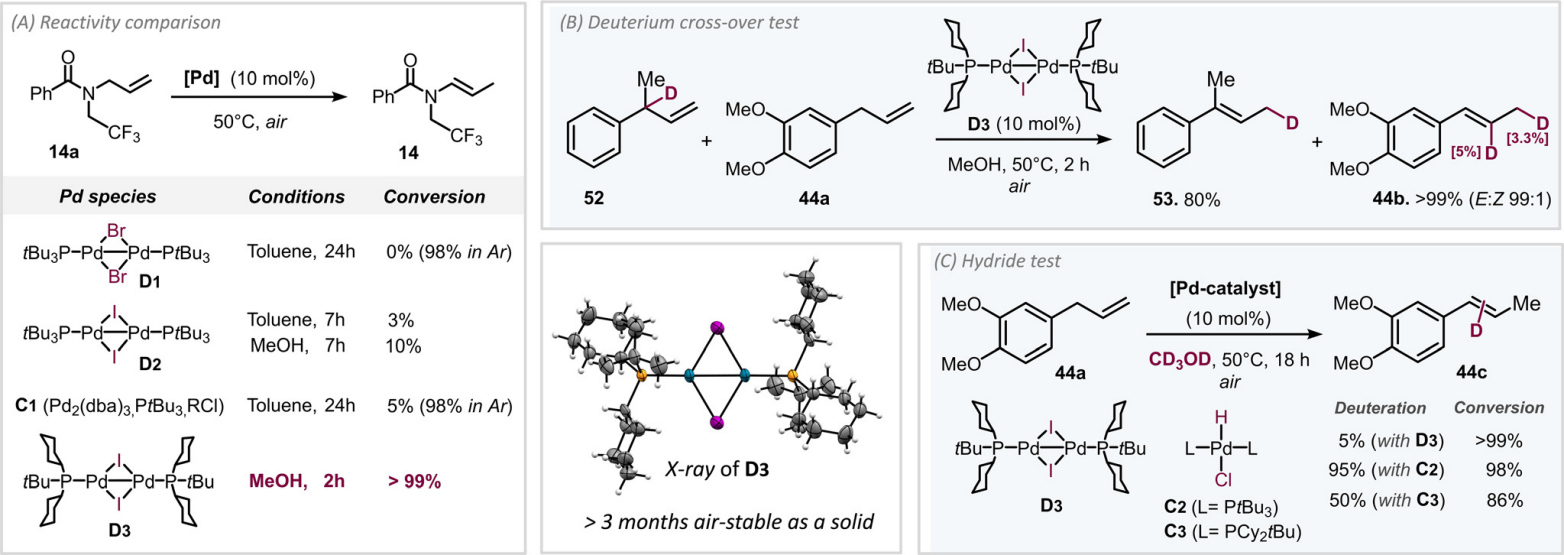
X‐ray structure of new dimer **D3**; systematic comparison of catalyst performance and mechanistic deuteration data.

Our assessment of its air stability indicated that it is bench stable as a solid for at least three months (which constitutes the time of our testing).

With a new generation of air‐stable Pd^I^ dimer **D3** in hand, we subsequently investigated its reactivity in the isomerization of substrate **14 a** (see Figure 2 A). To our delight, quantitative isomerization of **14 a** to *E*‐enamide **14** occurred in MeOH under open‐flask conditions in 2 h at 50 °C.^[21]^ By contrast, [Pd(*μ*‐I)(P^*t*^Bu_3_)]_2_ **D2** gave only 10 % conversion in MeOH (and 3 % in toluene) even after 7 h at 50 °C. The more labile Br‐bridged **D1** gave no conversion whatsoever under open‐flask conditions due to its oxygen‐sensitivity, but effectively yielded **14** under argon. Similarly, Skrydstrup's Pd^II^‐H system **C2** ^[15]^ that is generated in situ from a mixture of Pd_2_(dba)_3_, isobutyryl chloride, and tri‐*tert*‐butylphosphine (**C1**) was effective under argon, but not when the reaction was set‐up and performed in air.

Given the great promise of the newly identified air‐stable dimer **D3** to trigger double bond migrations without the need for inert conditions, we set out to investigate the wider scope. We tested the isomerization of a variety of alternative tertiary amides (see Figure 3). Simple subjection of **D3** to the amides in MeOH for 2 h at 50 °C under open flask conditions yielded the corresponding enamides in excellent yields (>95 %) and high *E*‐selectivity, regardless of the electronic or steric bias imposed by the substrate. Aromatic (**1**–**16**, **25**), aliphatic (**17**–**20**, **32**–**35**) as well as heterocyclic amides (**21**–**24**) were equally efficient. Potentially coordinating or frequently reactive functional groups, such as OMe, NMe_2_, CN or halides I, Br, Cl (**4**, **10**, **11**) did not impede the transformation. Similarly, valuable fluorinated motifs, such as CF_3_ or CH_2_CF_3_ were well tolerated as substituents either on the aromatic ring or on the amide nitrogen. *N*‐benzyl and ethyl substituents were also effective. Notably, the isomerization was equally efficient and selective for allyl sulfonamides (**26**–**29**) and phosphamides (**30**, **31**). The stereochemical integrity of a variety of amino acid derived amides was also fully retained, yielding chiral amides **32**–**35** in excellent yield and selectivity.

**Figure 3:**
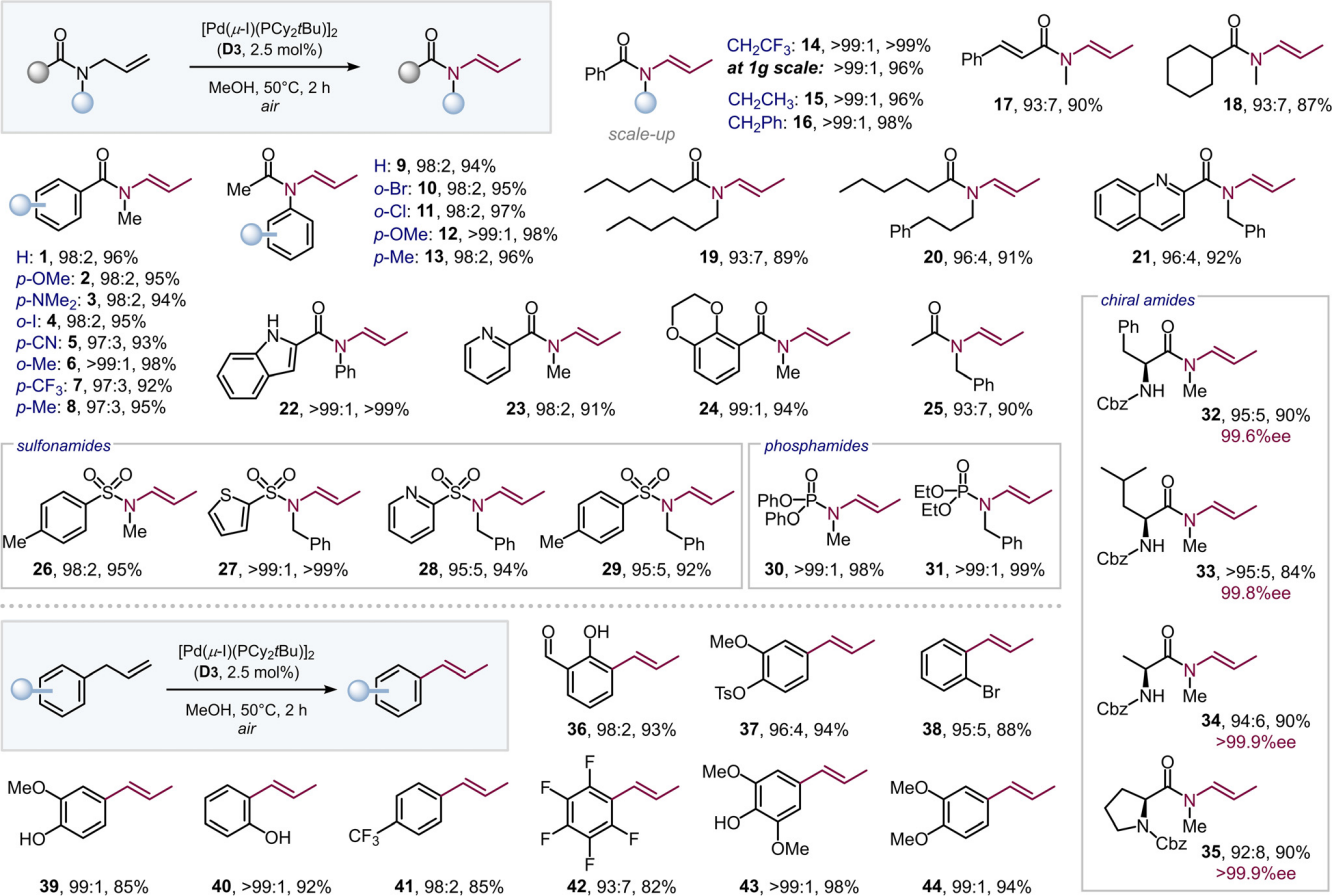
Scope of *E*‐selective olefin migration enabled by new Pd^I^ dimer **D3** in air. Reaction conditions: **D3** (5 μmol), terminal olefin (0.2 mmol), MeOH (1.0 mL), 50 °C, 2 h. Yields of isolated products after chromatography, *E*/*Z* ratios were determined by GC‐MS or quantitative ^1^H NMR.^[25]^

The open‐flask isomerization process is also amenable to scale‐up: when we performed the reaction on 1 gram scale we isolated enamide **14** in 96 % yield with *E*/*Z*>99:1 after simple filtration and solvent removal as purification.

We next set out to test the wider potential of catalyst **D3** in isomerizations other than amide to enamide conversions. Pleasingly, **D3** was just as powerful for all‐carbon‐based olefin transpositions and we successfully converted a number of allyl benzenes. Electron‐rich as well as electron‐poor substituents were tolerated, including OH groups, even if positioned *ortho* to the isomerization site (**40**).[^22^, ^23^]

Our preliminary mechanistic data on the nature of the active species indicated a palladium hydride to be likely. While we found that dimer **D3** is of low solubility in MeOH, in the presence of a substrate in MeOH, we were able to detect two species by ^31^P‐NMR analysis during the course of the reaction, which likely correspond to a [Pd^II^‐H] (63.8 ppm) and a cyclopalladated Pd species (−9.9 ppm). Analogous species were previously seen in isomerizations with **D1**.^[10]^ Interestingly, when we performed the one‐pot simultaneous isomerization of **52**, **44 a** with **D3** in MeOH (see Figure 2 B), we only saw very little deuterium scrambling in the products **53**, **44 b**. Roughly 8 % deuterium content was detected in **44 b**, which indicates that the monophosphine Pd^II^‐H appears to stay largely coordinated to the substrate during the course of the isomerization.

Moreover, when we performed the isomerization of allyl benzene **44 a** in deuterated methanol (CD_3_OD), we observed only very little D incorporation in the substrate (<5 %, Figure 2 C). This suggests that the L_1_Pd^(II)^(H)(X) [L=PCy_2_^*t*^Bu] that is initially formed via cyclopalladation barely undergoes exchange with the solvent. By contrast, when we separately synthesized the corresponding bisphosphine complexes L_2_Pd^II^(H)(Cl) [L=PCy_2_^*t*^Bu and P^*t*^Bu_3_]^[24]^ **C2** and **C3** and subjected these species to the isomerization of **44 a** in CD_3_OD, substantial deuteration in the product was observed. For L=P^*t*^Bu_3_, 95 % of deuterium content was detected in **44 c**, whereas 50 % deuteration was seen for L=PCy_2_^*t*^Bu. These data indicate that the ligation state of the palladium hydride substantially affects the deuterium scrambling and intermolecular cross‐overs. Pd^I^ dimers are in this respect privileged in forming monophosphine [Pd^II^‐H] directly.

Overall, these data showcase that double bond migrations are feasible in open air for a range of substrates and substitution patterns, which was previously not possible with the first generation air‐stable Pd^I^ dimer **D2**.

We previously showed that dimer **D2** gives rise to extremely rapid and fully selective arylation and alkylation of aromatic C−Br bonds in the presence of C−OTf and C−Cl.[^14a^, ^14h^, ^14i^] As opposed to typical Pd^0^ based transformations, the selectivity was independent of any steric or electronic impacts imposed by the substrates. Even if challenged with an adamantyl group *ortho* to C−Br, the coupling still occurs there in favour over a less hindered C−OTf site.^[14a]^ Our data indicated that while Pd^I^ can in principle directly add to C−I or C−Br bonds, the barriers for addition to C−Cl or C−OTf are too high. However, we found that use of a polar solvent along with an organometallic reagent allows for C−OTf functionalization with **D2** in air in less than 10 min (at r.t.),^[14d]^ which is most likely due to an ate complex having been generated which then triggers the transformation. While we found C‐OTf functionalization to be quite broad, it occasionally proceeded inefficiently when there was *ortho* substitution to the coupling site (Figure 4).

**Figure 4:**
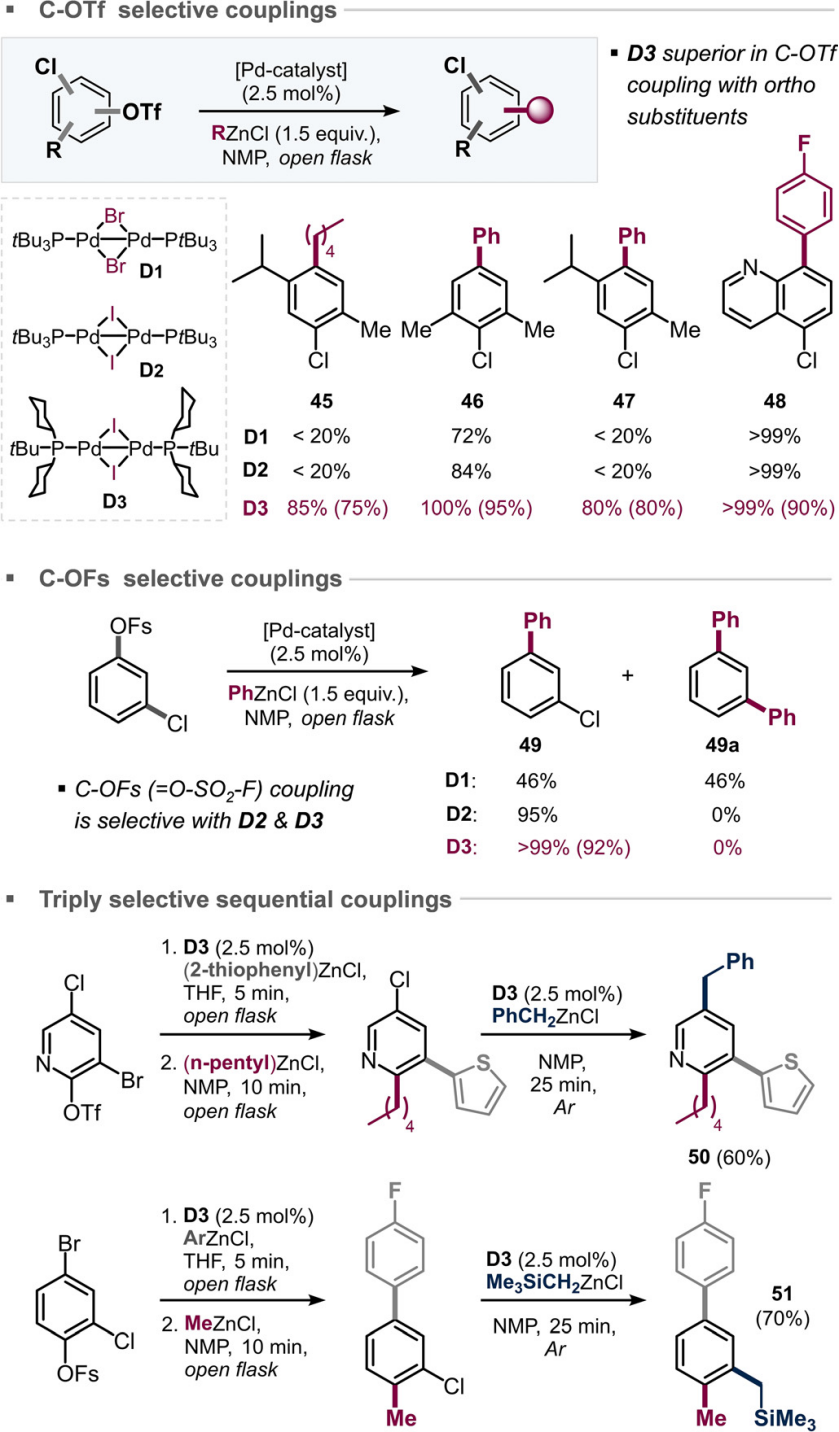
Site selective C−C coupling of poly(pseudo)halogenated arenes and *ortho*‐challenge for C‐OTf derivatization. Reaction conditions: **D3** (5 μmol), substrate (0.2 mmol), RZnCl (1.2–2.0 equiv) NMP (1.0 mL), r.t. Conversions as determined by GC‐MS are shown, yields of isolated product are given in parentheses.^[25]^

We were intrigued whether **D3** can also trigger site‐selective C−C bond formations, especially if sterically challenged. Figure 4 shows the results. The data indicate that dimer **D3** can also trigger selective C${}_{\mathrm{sp}{}^{2}}$ ‐C${}_{\mathrm{sp}{}^{3}}$ as well as C${}_{\mathrm{sp}{}^{2}}$ ‐C${}_{\mathrm{sp}{}^{2}}$ couplings of C‐OTf in NMP under open‐flask conditions in less than 10 min at r.t. Interestingly, while **D2** yielded poor conversion for the hindered **45** and **47** in <20 %, the new dimer **D3** gave excellent conversion and exclusive C‐OTf selectivity. For comparison, bromide‐bridged **D1** also gives poor (<20 %) conversion when subjected to the same transformations under argon (in air the dimer is deactivated prior to reaction). For the functionalization of the analogous fluorosulfate C‐OSO_2_F (OFs) in the presence of C−Cl,^[14b]^ **D1** gave no selectivity, whereas **D2** and **D3** gave fully selective couplings in excellent yields. Moreover, in analogy to **D2**,^[14d]^ the new generation **D3** appears to be similarly effective in C−Br and C−Cl couplings under the analogous reaction conditions, which allows for the triply selective and modular functionalization of arenes also.

In conclusion, disclosed herein was the preparation and reactivity studies of a new generation of air‐stable Pd^I^ dimer. The newly developed [Pd(*μ*‐I)(PCy_2_^*t*^Bu)]_2_ **D3** allowed for the first efficient *E*‐selective olefin migrations in air in just 2 h reaction time to generate *E*‐enamides or styrene derivatives in the presence of various functionalities, including sulfones, (phosph)amides, aldehydes, ester, stereocenters, and even tolerating the frequently reactive aromatic C−I bonds or *ortho*‐OH groups. Our mechanistic data indicate that barely any H/D‐crossover and exchange takes place, which likely is due to a monophosphine [Pd^II^‐H] being generated as active species. Dimer **D3** was shown to also trigger rapid and chemoselective C${}_{\mathrm{sp}{}^{2}}$ ‐C${}_{\mathrm{sp}{}^{2}}$ as well as C${}_{\mathrm{sp}{}^{2}}$ ‐C${}_{\mathrm{sp}{}^{3}}$ cross coupling reactions of poly(pseudo)halogenated arenes in air, showing superior reactivity compared to previous dimer generations for triflate substrates that contain *ortho* substituents.

## Conflict of interest

The authors declare no conflict of interest.

## Supporting information

As a service to our authors and readers, this journal provides supporting information supplied by the authors. Such materials are peer reviewed and may be re‐organized for online delivery, but are not copy‐edited or typeset. Technical support issues arising from supporting information (other than missing files) should be addressed to the authors.

Supplementary
