# Supplementary material for: A Next‐Generation Air‐Stable Palladium(I) Dimer Enables Olefin Migration and Selective C−C Coupling in Air
Source: Angew Chem Int Ed Engl. 2020 Sep 28;59(49):21930–4. doi: 10.1002/anie.202009115 (PMC7756449; doi:10.1002/anie.202009115)
Supplement: Supplementary file 1 — Supplementary [file ANIE-59-21930-s001.pdf]

Angewandte Chemie
Eine Zeitschrift der Gesellschaft Deutscher Chemiker

Supporting Information

A Next-Generation Air-Stable Palladium(I) Dimer Enables Olefin Migration and Selective C−C Coupling in Air

*Gourab Kundu, Theresa Sperger, Kari Rissanen, and Franziska Schoenebeck**

anie_202009115_sm_miscellaneous_information.pdf

Contents

1. General Experimental Details ... 2
2. Synthesis and characterization data of catalyst $[Pd(\mu\text{-}I)(PCy_2tBu)]_2$ ... 3
3. Synthesis and characterization data for the starting materials for olefin isomerisation ... 4
   3.1. General Experimental Procedures ... 4
   3.2. Characterization data of starting materialsfor olefin isomerisation ... 6
4. Synthesis and characterization data for the starting materials for site selective coupling ... 16
   4.1. General Experimental Procedures ... 16
   4.2. Characterization data of starting materials for selective coupling ... 17
5. General procedure for olefin isomerization and characterization data of *E*-olefins ... 18
   5.1. General procedure for olefin isomerization ... 18
   5.2. Characterization data of *E*-olefins ... 18
6. General procedure for site selective couplings and characterization data of products ... 30
   6.1. General procedure for site selective coupling reactions ... 30
   6.2. Characterization data of cross-coupling products ... 31
7. Mechanistic Studies ... 33
   7.1. Reaction Progress ... 33
   7.2. Control experiment with $Pd^{(II)}H$ in air ... 34
   7.3. Cross-over experiment ... 35
   7.4. Deuteration experiments ... 38
8. Crystallographic Data ... 43
9. HPLC traces of chiral compounds ... 44
10. NMR Spectra ... 48
11. References ... 104

1. General Experimental Details

Reagents, starting materials and solvents

Unless otherwise stated, all reagents and starting materials were commercially available and used as received. All anhydrous solvents were either purchased from Acros or dried using an Innovative Technology PS-MD-5 solvent purification system. Technical grade solvents were distilled prior to use for chromatography and extraction.

Purification

Thin layer chromatography (TLC) was performed on Merck Kieselgel 60 F254 aluminium plates with unmodified silica and visualized either under UV light or stained with iodine. Flash column chromatography was performed with Merck silica gel 60 (35 – 70 mesh). Preparative HPLC was performed on a Gilson-Abimed HPLC (employing UV detector model 117) using a Merck LiChrosorb Si60 column (porosity 7 μm, 250 x 25 mm).

Characterization

All ^{1}H, ^{13}C and ^{19}F NMR spectra were recorded on Bruker Avance Neo 600, Varian VNMRS 600 or Varian VNMRS 400 spectrometers at ambient temperature. Chemical shifts (δ) are reported in parts per million (ppm) and were referenced either to residual solvent peak ($CDCl_3$; for ^{1}H and ^{13}C spectra), $CHCl_3$ (δ = 7.26 ppm, added as an internal standard for ^{2}H), $POCl_3$ (δ = 2.79 ppm in THF, added as an internal standard for ^{31}P) or $O{=}P(OMe)_3$ (δ = 3.05 ppm in THF, added as an internal standard for ^{31}P). Coupling constants (*J*) are given in Hertz (Hz).

Gas chromatography coupled with mass spectrometry (GC-MS) was performed on an Agilent Technologies 5975 series MSD mass spectrometer under electrospray ionization (EI) mode coupled with an Agilent Technologies 7820A gas chromatograph employing an Agilent 19091s-433 HP-5MS column (30 m x 0.250 μm x 0.250 μm).

High-resolution mass spectrometry (HRMS) was performed using a Thermo Scientific LTQ Orbitrap XL spectrometer. Low-resolution masses of known compounds were extracted from their GC-MS chromatograms. IR spectra were recorded on a Spectrum 100 spectrometer with an UATR Diamond/KRS-5 crystal with attenuated total reflectance (ATR).

Analytical chiral HPLC was performed on an Agilent 1100 Series instrument with DAD detector using a Daicel Chiralpak IC column (4.6 mm diameter, 150 mm length, 5μm porosity; IC00CD-QF015).

2. Synthesis and characterization data of catalyst $[Pd(\mu\text{-}I)(PCy_2tBu)]_2$

$[Pd(PCy_2tBu)_2]$

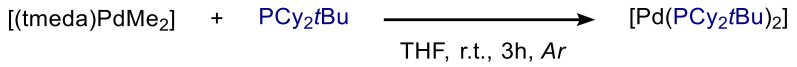

Inside an Argon-filled glovebox, [(tmeda)PdMe₂][1] (253 mg, 1.0 mmol, 1.0 equiv.) and PCy_2tBu (508 mg, 2.0 mmol, 2.0 equiv.) were dissolved in THF (3.3 mL) in a 15 mL vial. The resulting mixture was stirred at ambient temperature for 3 hours. Then THF was removed under reduced pressure and DMF (3.3 mL) was added to the solid. The insoluble solid suspension was filtered and the filtrate was washed with DMF (3 mL) followed by cold pentane (0.2 mL, to facilitate drying). The obtained white solid (480 mg, 0.78 mmol, 78%) was further dried *in vacuo* for 2 hours. ^{1}H NMR (400 MHz, Benzene-d_6) δ 2.32 (d, *J* = 12.0 Hz, 4H), 2.18 (d, *J* = 12.0 Hz, 4H), 1.92 – 1.65 (m, 20H), 1.65 (br, 4H), 1.39 (t, *J* = 5.9 Hz, 18H), 1.31 – 1.22 (m, 12H). ^{13}C NMR (101 MHz, Benzene-d_6) δ 36.0 (t, *J* = 4.1 Hz), 33.8 (t, *J* = 4.8 Hz), 33.0 (t, *J* = 4.8 Hz), 32.1 (t, *J* = 3.1 Hz), 31.5 (t, *J* = 5.1 Hz), 28.1 (dt, *J* = 13.0, 5.0 Hz), 27.1. ^{31}P NMR (121 MHz, Toluene-d_8) δ 54.1.

$[Pd(\mu\text{-}I)(PCy_2tBu)]_2$ (D3)

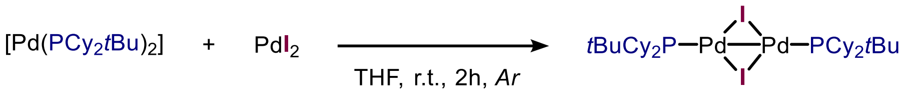

Inside an Argon-filled glovebox, $[Pd(PCy_2tBu)]_2$ (480 mg, 0.78 mmol, 1.0 equiv.), PdI_2 (262 mg, 0.73 mmol, 0.94 equiv.) were suspended in THF (15 mL) in a 250 mL round bottom flask. After 2 hours of stirring at ambient temperature, dry acetone (200 mL) was added and the resulting mixture was left to crystallize at -30°C overnight. Dark purple crystals of $[Pd(\mu\text{-}I)(PCy_2tBu)]_2$ (680 mg, 0.7 mmol, 89%) were collected by filtration and washed with cold dry acetone (50 mL). The obtained crystalline material was analysed by ^{31}P NMR. *Note*: The compound contained a small impurity (δ 48.6), which can be removed by further recrystallization (see below). However, further purification is not necessary as the purity of the catalyst did not show any effect on its catalytic efficiency and selectivity.

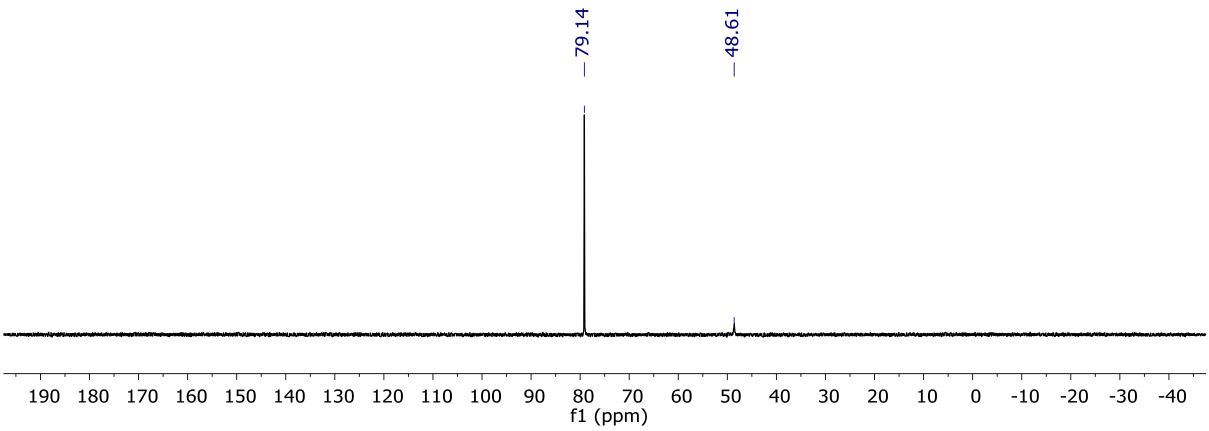

To obtain an analytically pure sample 100 mg of the obtained crystalline solid were recrystallized further by dissolving in toluene (3.5 mL), filtration using a syringe filter and addition of acetone (50 mL). The mixture was stored at -30°C for 48h and the formed crystals collected by filtration (28.3 mg). ^{1}H NMR (600 MHz, Benzene-d_6) δ = 2.00 – 1.84 (m, 8H), 1.65 – 1.56 (m, 20H), 1.51 (d, *J*=11.3, 4H), 1.19 – 1.01 (m, 30H). ^{13}C NMR (151 MHz, Benzene-d_6) δ = 35.2 (t, *J*=6.3), 32.2 (br), 31.7 (t, *J*=6.7), 30.74, 30.68 (t, *J*=3.2), 27.7 (dt, *J*=9.9, 5.4), 26.4. $^{31}P\{^{1}H\}$ NMR (243 MHz, Benzene-d_6) δ 79.2.

3. Synthesis and characterization data for the starting materials for olefin isomerisation

3.1. General Experimental Procedures

Method A[2]

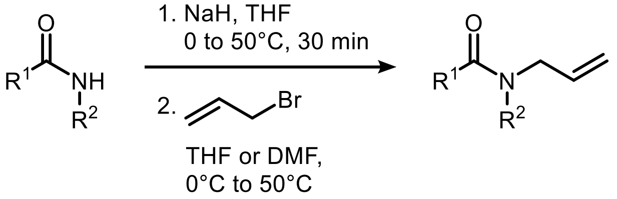

Under Ar atmosphere, *N*-substituted amide (5.0 mmol, 1.0 equiv) dissolved in 5 mL THF or DMF (depending on the solubility of amide) was added to a suspension of sodium hydride (144 mg, 6.0 mmol, 1.2 equiv.) in 10 mL THF or DMF (depending on the solubility of amide) at 0°C and stirred for 30 min at 50°C. Then allyl bromide (0.52 mL, 6.0 mmol, 1.2 equiv.) in THF (5 mL) was added at 0°C, and the mixture was stirred overnight at 50°C. The reaction mixture was cooled in an ice bath and was slowly quenched with water (10-15 mL). The aqueous phase was extracted with diethyl ether (3 x 20 mL) and the combined organic phases were dried over Na_2SO_4 and volatiles were removed under reduced pressure. The obtained crude was subjected to column chromatography on silica for further purification.

Method B

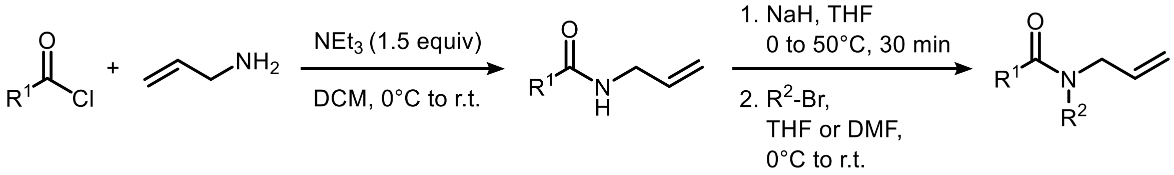

Under Ar atmosphere, allylamine (0.37 mL, 5.0 mmol, 1.0 equiv.) and triethylamine (1.05 mL, 7.5 mmol, 1.5 equiv.) were dissolved in DCM (10 mL) and were added slowly to a flame dried round-bottom flask containing acyl chloride (5.5 mmol, 1.1 equiv.) dissolved in DCM (10 mL) at 0°C and then the reaction mixture was stirred at room temperature overnight. After completion, it was cooled in an ice bath and was slowly quenched with water (10-15 mL). The aqueous phase was extracted with diethyl ether (3 x 20 mL) and the combined organic phases were dried over Na_2SO_4 and volatiles were removed under reduced pressure.

Without further purification, the crude material (5.0 mmol, 1 equiv.) dissolved in 5 mL THF or DMF (depending on the solubility of amide) was added to a suspension of sodium hydride (144 mg, 6.0 mmol, 1.2 equiv.) in 10 mL THF or DMF (depending on the solubility of amide) at 0°C and stirred for 30 min at 50°C. Then the corresponding bromide (6.0 mmol, 1.2 equiv.) in THF (5 mL) was added at 0°C and the mixture was stirred overnight at 50°C. The reaction mixture was cooled in an ice bath and was slowly quenched with water (10-15 mL). The aqueous phase was washed with diethyl ether (3 x 20 mL) and the combined organic phases were dried over Na_2SO_4 and volatiles were removed under reduced pressure. The obtained crude was subjected to column chromatography on silica for further purification.

Method C

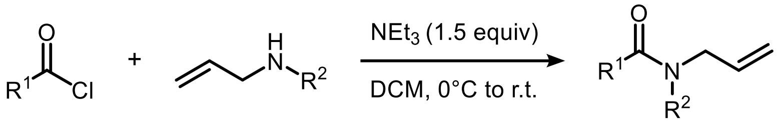

Under Ar atmosphere, *N*-substituted allylamine (5.0 mmol, 1.0 equiv.) and triethylamine (1.05 mL, 7.5 mmol, 1.5 equiv.) were dissolved in DCM (10 mL) and were added slowly to a flame dried round-bottom flask containing acyl chloride (5.5 mmol, 1.1 equiv.) dissolved in DCM (10 mL) at 0°C and then the reaction mixture was stirred at room temperature overnight. After completion, it was cooled in an ice bath and was slowly quenched with water (10-15 mL). The aqueous phase was extracted with diethyl ether (3 x 20 mL) and the combined organic phases were dried over Na_2SO_4 and volatiles were removed under reduced pressure. The obtained crude was subjected to column chromatography on silica for further purification.

Method D

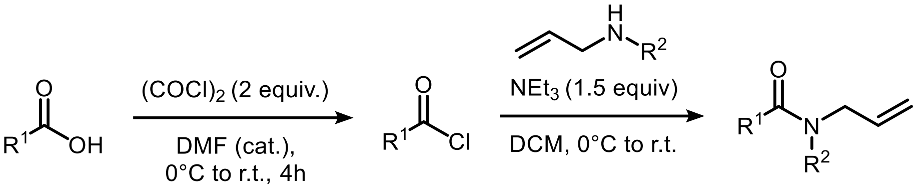

Under Ar atmosphere, two drops of DMF were added to a flame dried round-bottom flask containing carboxylic acid (5.0 mmol, 1.0 equiv.) dissolved in DCM (10 mL) at 0°C. Oxalyl chloride (0.86 mL, 10.0 mmol, 2.0 equiv.) was added slowly and the reaction mixture was stirred at room temperature for 4 hours. After completion, the solvent was removed under reduced pressure and the crude mixture was further dried under high vacuum.

Without further purification, the crude material (5.0 mmol, 1 equiv.) was dissolved in DCM (10 mL) at 0°C under Ar atmosphere. *N*-substituted allylamine (4.5 mmol, 0.9 equiv.) and triethylamine (1.05 mL, 7.5 mmol, 1.5 equiv.) were dissolved in DCM (10 mL) and were added slowly at 0°C and the mixture was stirred overnight at ambient temperature. The reaction mixture was cooled in an ice bath and was slowly quenched with water (10-15 mL). The aqueous phase was extracted with diethyl ether (3 x 20 mL) and the combined organic phases were dried over Na_2SO_4 and volatiles were removed under reduced pressure. The obtained crude was subjected to column chromatography on silica for further purification.

Method E

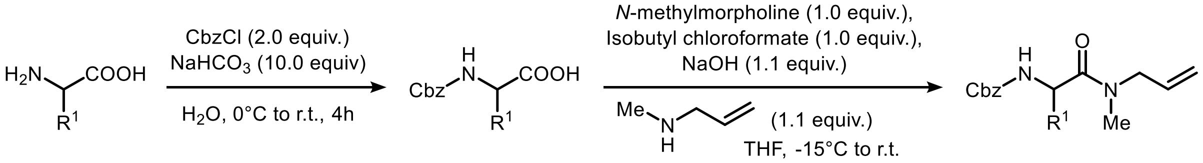

To an aquous solution (20 mL) of amino acid (5.0 mmol, 1.0 equiv.), $NaHCO_3$ (4.2 g, 50.0 mmol, 10.0 equiv.) was carefully added portionwise at 0°C. To the alkaline reaction mixture Cbz-Cl (3.4 mL of a 3.0 M solution in toluene, 10.0 mmol, 2.0 equiv.) was added dropwise and the resulting reaction mixture was slowly allowed to warm to room temperature and stirred until all starting material was consumed (typically 4 h, monitored by TLC). After completion of the reaction the aqueous phase was extracted with diethyl ether (5 x 20 mL) and the aquous phase was acidified with 1N HCl until the pH reached 1. Then

the aquous layer was extracted with EtOAc (3 x 20 mL) and the combined organic phases were dried over Na_2SO_4 and volatiles were removed under reduced pressure.

Without further purification, the crude material (5.0 mmol, 1 equiv.) was dissolved in THF (10 mL) and cooled to -15°C under Ar atmosphere. To the stirred solution, *N*-methylmorpholine (0.55 mL, 5.0 mmol, 1.0 equiv.) and isobutylchloroformate (682.9 mg, 5.0 mmol, 1.0 equiv.) were added successively. 1N NaOH (5.5 mL) was added to a solution of *N*-methylallylamine (0.53 mL, 5.5 mmol, 1.1 equiv) in a mixture of THF (5 mL) and water (5 mL) and the combined mixture was added to the reaction mixture. The reaction mixture was slowly allowed to warm to room temperature and stirred until all starting material was consumed (typically 2 h, monitored by TLC). After completion of the reaction the organic solvent was evaporated and the pH of the aquous phase was adjusted very slowly to 1 with 1 N HCl at -15°C. Then the aquous layer was extracted with EtOAc (5 x 20 mL). The combined organic phases were washed with $NaHCO_3$ and dried over Na_2SO_4 and volatiles were removed under reduced pressure. The obtained crude was subjected to column chromatography on silica for further purification.

3.2. Characterization data of starting materialsfor olefin isomerisation

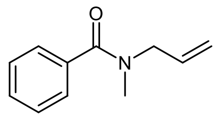

***N*-allyl-*N*-methylbenzamide** (**1a**): Prepared, following method A from *N*-methylbenzamide. The title product was obtained after purification by column chromatography (Hexane/EtOAc 70:30) as a yellow liquid (750 mg, 4.3 mmol, 86%). R_f = 0.33 (Hexane/EtOAc 70:30). ^{1}H NMR (600 MHz, $CDCl_3$): (mixture of rotamers) δ 7.42 – 7.37 (m, 5H), 5.90 – 5.84 (minor rotamer, m, 0.4H), 5.73 (major rotamer, ddt, *J* = 16.2, 10.4, 5.1 Hz, 0.6H), 5.28 – 5.19 (m, 2H), 4.15 – 4.14 (minor rotamer, m, 0.8H), 3.84 (major rotamer, s, 1.2H), 3.05 (major rotamer, s, 1.8H), 2.90 (minor rotamer, s, 1.2H).^{13}C NMR (151 MHz, $CDCl_3$): (mixture of rotamers) δ 172.2, 171.4, 136.5, 136.3, 133.2, 132.8, 129.7, 128.5, 127.1, 126.7, 117.8, 117.6, 54.1, 50.1, 37.0, 33.1. MS (70 eV, EI): *m/z* (%): 175 (5) [M$^+$], 160 (10), 105 (100), 77 (78), 70 (16), 51 (27). These data are in agreement with those reported previously in the literature.[3]

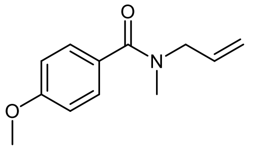

***N*-allyl-4-methoxy-*N*-methylbenzamide** (**2a**): Prepared, following method B from 4-methoxybenzoyl chloride. The title product was obtained after purification by column chromatography (Hexane/EtOAc 70:30) as a colorless oil (903 mg, 4.4 mmol, 88%). R_f = 0.53 (Hexane/EtOAc 50:50). ^{1}H NMR (600 MHz, $CDCl_3$, 50°C): δ 7.41 (d, *J* = 7.9 Hz, 2H), 6.89 (d, *J* = 7.9 Hz, 2H), 5.87 – 5.77 (m, 1H), 5.26 – 5.21 (m, 2H), 4.00 – 3.99 (m, 2H), 3.83 – 3.82 (m, 3H), 3.00 (s, 3H). ^{13}C NMR (151 MHz, $CDCl_3$): δ 160.7, 152.5, 133.2, 128.8, 128.4, 117.5, 113.6, 55.4, 54.2, 33.3. IR (neat, cm^{-1}): 2928, 2840, 1625, 1451, 1393, 1297, 1247, 1174, 1068, 1027, 840. MS (70 eV, EI): *m/z* (%): 205 (6) [M$^+$], 135 (100), 92 (13), 77 (16). HRMS (ESI) calculated for $C_{12}H_{15}O_2NNa$: 228.0995 [M+Na]$^+$, found: 228.0999.

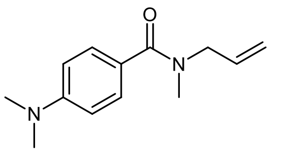

***N*-allyl-4-(dimethylamino)-*N*-methylbenzamide** (**3a**): Prepared, following method B from 4-(dimethylamino)benzoyl chloride. The title product was obtained after purification by column chromatography (Hexane/EtOAc 70:30) as a yellowliquid (895 mg, 4.1 mmol, 82%). R_f = 0.38 (Hexane/EtOAc 50:50). ^{1}H NMR (400 MHz, $CDCl_3$): (mixture of rotamers) δ 7.52 (minor rotamer, d, *J* = 9.0 Hz, 0.4H), 7.39 (major rotamer, d, *J* = 8.8 Hz, 1.6H), 6.66 (major rotamer, d, *J* = 8.8 Hz, 1.6H), 6.45 (minor rotamer, d, *J* = 8.9 Hz, 0.4H), 6.04 (minor rotamer, ddt, *J* = 16.3, 10.8, 5.7 Hz, 0.2H), 5.84 (major rotamer, ddt, *J* = 15.2, 10.3, 5.3 Hz, 0.8H), 5.29 – 5.22 (m, 2H), 4.02 (s, 2H), 3.01 – 2.95 (m, 9H).^{13}C NMR (101 MHz, $CDCl_3$): δ 151.6, 133.7,

131.3, 129.1, 123.2, 117.3, 111.2, 40.4, 40.1. IR (neat, cm^{-1}): 2899, 2808, 1607, 1388, 1258, 825. MS (70 eV, EI): *m/z* (%): 218 (13) [M^+], 148 (100), 119 (7), 77 (5). HRMS (ESI) calculated for $C_{13}H_{18}ON_2Na$: 241.1311 $[M+Na]^+$, found: 241.1315.

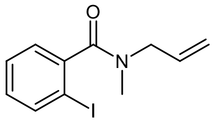

***N*-allyl-2-iodo-*N*-methylbenzamide** (**4a**): Prepared, following method C from 2-iodobenzoyl chloride. The title product was obtained after purification by column chromatography (Hexane/EtOAc 85:15) as a yellow liquid (1.2 g, 4.0 mmol, 80%). R_f = 0.43 (Hexane/EtOAc 70:30). ^{1}H NMR (400 MHz, $CDCl_3$): (mixture of rotamers) δ 7.82 (d, *J* = 8.0 Hz, 1H), 7.41 – 7.34 (m, 1H), 7.23 – 7.18 (m, 1H), 7.09 – 7.04 (m, 1H), 5.92 (rotamer, ddt, *J* = 16.4, 10.2, 6.1 Hz, 0.5H), 5.71 (rotamer, ddt, *J* = 16.0, 10.4, 5.7 Hz, 0.5H), 5.35 – 5.13 (m, 2H), 4.38 – 4.11 (m, 1H), 3.73- 3.68 (m, 1H), 3.08 (rotamer, s, 1.5H), 2.77 (rotamer, s, 1.5H). ^{13}C NMR (101 MHz, $CDCl_3$): δ 173.2, 171.0, 142.9, 142.6, 139.3, 132.7, 132.5, 130.3, 130.2, 128.6, 128.3, 127.2, 118.4, 118.2, 92.8, 92.5, 53.7, 49.6, 35.9, 32.4. IR (neat, cm^{-1}): 2921, 1633, 1399, 770, 746. MS (70 eV, EI): *m/z* (%): 301 (10) [M^+], 231 (100), 202 (34), 174 (54), 105 (11), 76(29). HRMS (ESI) calculated for $C_{11}H_{12}ONINa$: 323.9856 $[M+Na]^+$, found: 323.9864.

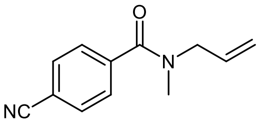

***N*-allyl-4-cyano-*N*-methylbenzamide** (**5a**): Prepared, following method B from 4-cyanobenzoyl chloride. The title product was obtained after purification by column chromatography (Hexane/EtOAc 70:30) as a colorless liquid (640 mg, 3.2 mmol, 64%). R_f = 0.34 (Hexane/EtOAc 50:50). ^{1}H NMR (600 MHz, $CDCl_3$): (mixture of rotamers) δ 7.72 – 7.68 (m, 2H), 7.53 (d, *J* = 7.5 Hz, 2H), 5.86 (minor rotamer, ddt, *J* = 16.4, 10.5, 5.9 Hz, 0.4H), 5.72 (major rotamer, ddt, *J* = 15.7, 9.9, 4.8 Hz, 0.6H), 5.30 – 5.20 (m, 2H), 4.16 – 4.15 (minor rotamer, m, 0.8H), 3.80 (major rotamer, s, 1.2H), 3.08 (major rotamer, s, 1.8H), 2.88 (minor rotamer, s, 1.2H). ^{13}C NMR (151 MHz, $CDCl_3$): (mixture of rotamers) δ 170.2, 169.3, 140.8, 140.7, 132.5 (132.48), 132.5 (132.45), 132.2, 127.9, 127.5, 118.5, 118.3, 118.0, 113.6, 113.5, 53.8, 50.1, 36.9, 33.4. IR (neat, cm^{-1}): 2925, 2230, 1631, 1400, 1263, 1067, 848. MS (70 eV, EI): *m/z* (%): 200 (10) [M^+], 185 (24), 130 (100), 102 (49), 70 (19). HRMS (ESI) calculated for $C_{12}H_{12}ON_2Na$: 223.0842 $[M+Na]^+$, found: 223.0844.

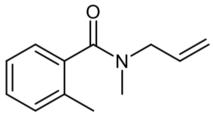

***N*-allyl-*N*,2-dimethylbenzamide** (**6a**): Prepared, following method B from 2-methylbenzoyl chloride. The title product was obtained after purification by column chromatography (Hexane/EtOAc 80:20) as a colorless liquid (737 mg, 3.9 mmol, 78%). R_f = 0.58 (Hexane/EtOAc 50:50). ^{1}H NMR (600 MHz, $CDCl_3$): (mixture of rotamers) δ 7.28 – 7.24 (m, 1H), 7.21 – 7.15 (m, 3H), 5.88 (rotamer, ddt, *J* = 16.4, 10.0, 6.0 Hz, 0.5H), 5.65 (rotamer, ddt, *J* = 16.9, 9.9, 5.5 Hz, 0.5H), 5.28 – 5.25 (rotamer, m, 1H), 5.19 – 5.11 (rotamer, m, 1H), 4.19 (rotamer, s, 1H), 3.71 (rotamer, s, 1H), 3.08 (rotamer, s, 1.5 H), 2.75 (rotamer, s, 1.5 H), 2.30 – 2.29 (m, 3H). ^{13}C NMR (151 MHz, $CDCl_3$): (mixture of rotamers) δ 171.9, 171.3, 136.8, 136.6, 134.2, 134.0, 132.9, 132.8, 130.5 (130.51), 130.5 (130.48), 128.9 (128.93), 128.9 (128.88), 126.1, 126.0, 125.9, 125.8, 118.0, 53.5, 49.4, 35.9, 32.3, 19.2, 19.0. IR (neat, cm^{-1}): 2922, 1632, 1396, 771, 740. MS (70 eV, EI): *m/z* (%): 189 (13) [M^+], 174 (17), 119 (100), 91 (49), 65 (16). HRMS (ESI) calculated for $C_{12}H_{15}ONNa$: 212.1046 $[M+Na]^+$, found: 212.1048.

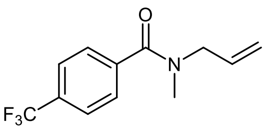

***N*-allyl-*N*-methyl-4-(trifluoromethyl)benzamide** (**7a**): Prepared, following method B from 4-(trifluoromethyl)benzoyl chloride. The title product was obtained after purification by column chromatography (Hexane/EtOAc 75:25) as a colorless oil (1.10 g, 4.5 mmol, 90%). R_f = 0.57 (Hexane/EtOAc 50:50). ^{1}H NMR (600 MHz, $CDCl_3$): (mixture of rotamers) δ 7.69 – 7.65 (m, 2H), 7.54 (d, *J* = 7.8 Hz, 2H), 5.88 (minor

rotamer, ddt, *J* = 16.7, 11.1, 6.0 Hz, 0.4H), 5.74 (major rotamer, ddt, *J* = 15.9, 10.1, 4.9 Hz, 0.6H), 5.29 – 5.21 (m, 2H), 4.17 – 4.16 (minor rotamer, m, 0.9H), 3.82 (major rotamer, s, 1.1H), 3.08 (major rotamer, s, 1.8H), 2.89 (minor rotamer, s, 1.2H). ^{13}C NMR (151 MHz, $CDCl_3$): (mixture of rotamers) δ 170.8, 170.0, 140.0, 139.9, 132.7, 132.4, 131.8, 131.6, 127.5, 127.1, 125.6, 123.9 (q, *J* = 272.4 Hz,), 118.3, 117.9, 53.9, 50.1, 36.9, 33.3. ^{19}F NMR (564 MHz, $CDCl_3$): δ −62.91 (s). IR (neat, cm^{-1}): 2928, 1636, 1403, 1322, 1165, 1121, 1067, 850. MS (70 eV, EI): *m/z* (%): 243 (10) [M$^+$], 228 (21), 173 (100), 145 (60), 70(14). HRMS (ESI) calculated for $C_{12}H_{12}ONF_3Na$: 266.0763 [M+Na]$^+$, found: 266.0766.

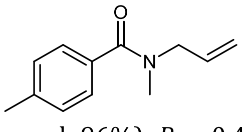

***N*-allyl-*N*,4-dimethylbenzamide** (**8a**): Prepared, following method B from 4-methylbenzoyl chloride. The title product was obtained after purification by column chromatography (Hexane/EtOAc 80:20) as a yellow liquid (908 mg, 4.8 mmol, 96%). R_f = 0.41 (Hexane/EtOAc 60:40). ^{1}H NMR (400 MHz, $CDCl_3$): (mixture of rotamers) δ 7.93 (minor rotamer, d, *J* = 8.2Hz, 0.1H), 7.32 (major rotamer, d, *J* = 8.0 Hz, 1.9H), 7.17 (d, *J* = 7.3 Hz, 2H), 5.91–5.73 (m, 1H), 5.25 – 5.19 (m, 2H), 4.13 – 4.12 (m, 1H), 3.85 – 3.84 (m, 1H), 3.03 – 2.91 (m, 3H), 2.35 (s, 3H). ^{13}C NMR (101 MHz, $CDCl_3$): δ 172.4, 171.5, 139.8, 133.4, 133.3, 132.9, 130.1, 129.0, 127.2, 126.8, 117.7, 117.4, 54.1, 50.1, 37.1, 33.2, 21.5. IR (neat, cm^{-1}): 2921, 1629, 1395, 1262, 1066, 921, 829, 751. MS (70 eV, EI): *m/z* (%): 189 (12) [M$^+$], 119 (100), 91 (35), 70 (11), 65 (13). HRMS (ESI) calculated for $C_{12}H_{15}ONNa$: 212.1046 [M+Na]$^+$, found: 212.1048.

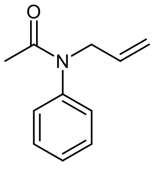

***N*-allyl-*N*-phenylacetamide** (**9a**): Prepared, following method A from *N*-phenylacetamide. The title product was obtained after purification by column chromatography (Hexane/EtOAc 80:20) as a yellow liquid (788 mg, 4.5 mmol, 90%). R_f = 0.33 (Hexane/EtOAc 70:30). ^{1}H NMR (400 MHz, $CDCl_3$): δ 7.40 (dd, *J* = 7.4, 7.4 Hz, 2H) 7.33 (dd, *J* = 7.3, 7.3 Hz, 1H), 7.15 (d, *J* = 7.4 Hz, 2H), 5.86 (ddt, *J* = 16.7, 10.2, 6.3 Hz, 1H), 5.11–5.04 (m, 2H), 4.29 (d, *J* = 6.2 Hz, 2H), 1.86 (s, 3H). ^{13}C NMR (101 MHz, $CDCl_3$): δ 170.2, 143.2, 133.3, 129.7, 128.3, 128.0, 117.9, 52.2, 22.9. MS (70 eV, EI): *m/z* (%): 175 (22) [M$^+$], 132 (100), 106 (77), 77 (34). These data are in agreement with those reported previously in the literature.[3]

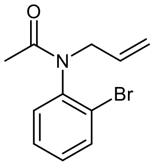

***N*-allyl-*N*-(2-bromophenyl)acetamide** (**10a**): Prepared, following method A from *N*-(2-bromophenyl)acetamide. The title product was obtained after purification by column chromatography (Hexane/EtOAc 70:30) as a colorless oil (1.14 g, 4.5 mmol, 90%). R_f = 0.5 (Hexane/EtOAc 60:40). ^{1}H NMR (600 MHz, $CDCl_3$): δ 7.68 (dd, *J* = 8.0, 1.3 Hz, 1H), 7.37 – 7.34 (m, 1H), 7.25 – 7.19 (m, 2H), 5.88 (ddt, *J* = 17.4, 10.1, 5.7 Hz, 1H), 5.10 – 5.03 (m, 2H), 4.79 – 4.75 (m, 1H), 3.73 – 3.70 (m, 1H), 1.81 (s, 3H). ^{13}C NMR (151 MHz, $CDCl_3$): δ 170.2, 141.5, 134.0, 132.9, 131.2, 129.9, 128.7, 124.1, 118.6, 51.0, 22.6. MS (70 eV, EI): *m/z* (%): 254 (1) [M$^+$], 211 (13), 184 (21), 174 (100), 132 (35), 105 (10), 77 (10). These data are in agreement with those reported previously in the literature.[3]

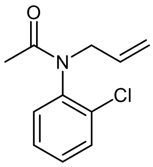

***N*-allyl-*N*-(2-chlorophenyl)acetamide** (**11a**): Prepared, following method A from *N*-(2-chlorophenyl)acetamide. The title product was obtained after purification by column chromatography (Hexane/EtOAc 70:30) as a colorless oil (996 mg, 4.77 mmol, 95%). R_f = 0.55 (Hexane/EtOAc 60:40). ^{1}H NMR (600 MHz, $CDCl_3$): δ 7.51 – 7.49 (m, 1H), 7.32 – 7.30 (m, 2H), 7.22 – 7.20 (m, 1H), 5.86 (ddt, *J* = 16.1, 10.1, 5.9 Hz, 1H), 5.09 – 5.02 (m, 2H), 4.71 – 4.64 (m, 1H), 3.84 – 3.80 (m, 1H), 1.81 (s, 3H). ^{13}C NMR (151 MHz, $CDCl_3$): δ 170.3, 140.0, 133.5, 133.0, 131.0, 130.8, 129.7, 128.0, 118.5, 51.0, 22.4. IR (neat, cm^{-1}): 3074, 2925, 1664, 1479, 1384, 1288, 753. MS

(70 eV, EI): *m/z* (%): 209 (2) [M+], 174 (100), 167 (30), 140 (38), 132 (57), 130 (24). HRMS (ESI) calculated for $C_{11}H_{12}ONClNa$: 232.0500[M+Na]+, found: 232.0505.

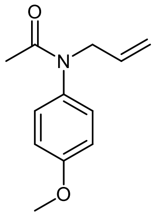

***N*-allyl-*N*-(4-methoxyphenyl)acetamide** (**12a**): Prepared, following method A from *N*-(4-methoxyphenyl)acetamide. The title product was obtained after purification by column chromatography (Hexane/EtOAc 70:30) as a colorless oil (985 mg, 4.8 mmol, 96%). R_f = 0.39 (Hexane/EtOAc 50:50). ^{1}H NMR (600 MHz, $CDCl_3$): δ 7.07 – 7.05(m, 2H), 6.91– 6.88 (m, 2H), 5.84 (ddt, *J* = 16.6, 10.2, 6.4 Hz, 1H), 5.10 – 5.03 (m, 2H), 4.25 (d, *J* = 6.3 Hz, 2H), 3.82 (s, 3H), 1.84 (s, 3H). ^{13}C NMR (151 MHz, $CDCl_3$): δ 170.7, 159.0, 135.9, 133.4, 129.3, 117.9, 114.8, 55.6, 52.2, 22.8. MS (70eV, EI): *m/z* (%): 205 (64) [M+], 163 (100), 149 (12), 136 (28), 122 (60), 95 (20), 82 (19). These data are in agreement with those reported previously in the literature.[3]

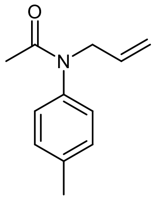

***N*-allyl-*N*-(p-tolyl)acetamide** (**13a**): Prepared, following method A from *N*-(*p*-tolyl)acetamide. The title product was obtained after purification by column chromatography (Hexane/EtOAc 80:20) as a colorless oil (842 mg, 4.45 mmol, 88%). R_f = 0.47 (Hexane/EtOAc 60:40). ^{1}H NMR (600 MHz, $CDCl_3$): δ 7.19 (d, *J* = 7.9 Hz, 2H), 7.03 (d, *J* = 7.9 Hz, 2H), 5.85 (ddt, *J* = 16.6, 9.7, 6.3 Hz, 1H), 5.10 – 5.04 (m, 2H), 4.27 (dd, *J* = 6.1, 1.5 Hz, 2H), 2.37 (s, 3H), 1.85 (s, 3H). ^{13}C NMR (151 MHz, $CDCl_3$): δ 170.4, 140.5, 137.9, 133.4, 130.3, 128.0, 117.8, 52.1, 22.8, 21.2. MS (70 eV, EI): *m/z* (%): 189 (37) [M+], 147 (100), 132 (49), 120 (67), 91 (33), 77 (14), 65 (15). These data are in agreement with those reported previously in the literature.[3]

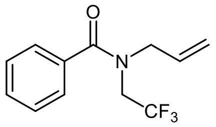

***N*-allyl-*N*-(2,2,2-trifluoroethyl)benzamide (14a):** Under Ar atmosphere, 2,2,2-trifluoroethanamine (495.3 mg, 5.0 mmol, 1.0 equiv.) and triethylamine (1.05 mL, 7.5 mmol, 1.5 equiv.) dissolved in DMF (10 mL) were added slowly to a flame dried round-bottom flask containing benzoyl chloride (0.64 mL, 5.5 mmol, 1.1 equiv.) dissolved in DMF (10 mL) at 0°C and then the reaction mixture was stirred at room temperature overnight. After completion, the reaction mixture was cooled in an ice bath and was slowly quenched with water (15 mL). The aqueous phase was extractedwith diethyl ether (3 x 20 mL) and the combined organic phases were dried over Na_2SO_4 and volatiles were removed under reduced pressure. The obtained crude was subjected to method A to prepare *N*-allyl-*N*-(2,2,2-trifluoroethyl)benzamide (**14a**). The title product was obtained after purification by column chromatography (Hexane/EtOAc 90:10) as a colorless oil (1.0 g, 4.15 mmol, 83%). R_f = 0.48 (Hexane/EtOAc 80:20). ^{1}H NMR (400 MHz, $CDCl_3$): δ 7.45 – 7.42 (m, 5H), 5.76 – 5.61 (m, 1H), 5.31 – 5.28 (m, 1H), 5.22 – 5.18 (m, 1H), 4.26 – 3.97 (m, 4H). ^{13}C NMR (101 MHz, $CDCl_3$, ^{19}F decoupled): δ 172.8, 135.1, 132.1, 130.2, 128.7, 126.7, 124.7, 119.2, 52.3, 44.2. ^{19}F NMR (376 MHz, $CDCl_3$): δ –69.05 (s). IR (neat, cm^{-1}): 3065, 2957, 1653, 1404, 1263, 1152, 1102, 930, 699. MS (70 eV, EI): *m/z* (%): 243 (4) [M+], 138 (12), 105 (100), 77 (44), 51 (12). HRMS (ESI) calculated for $C_{12}H_{12}ONF_3Na$: 266.0763 [M+Na]+, found: 266.0768.

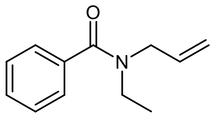

***N*-allyl-*N*-ethylbenzamide** (**15a**): Prepared, following method B (except ethyl iodide was used instead of ethyl bromide) from benzoyl chloride. The title product was obtained after purification by column chromatography (Hexane/EtOAc 85:15) as a colorless oil (492 mg, 2.60 mmol, 52%). R_f = 0.42 (Hexane/EtOAc 70:30). ^{1}H NMR (400 MHz, $CDCl_3$): δ 7.38 (s, 5H), 5.94 – 5.69 (m, 1H), 5.25 – 5.16 (m, 2H), 4.14 – 4.14 (m, 1H), 3.83 – 3.82 (m, 1H), 3.54 – 3.53 (m, 1H), 3.25 – 3.24 (m, 1H), 1.22 – 1.08 (m, 3H). ^{13}C NMR (101 MHz, $CDCl_3$): (mixture of rotamers) δ 171.6, 136.8, 133.7, 129.4, 128.5, 126.5, 117.4, 51.3, 46.7, 43.1, 39.8, 14.0, 12.6. MS (70 eV, EI): *m/z* (%):

189 (6) [M+], 174 (7), 105 (100), 84 (12), 77 (59), 51 (20). These data are in agreement with those reported previously in the literature.[4]

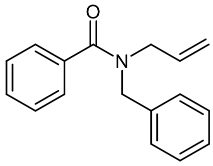

***N*-allyl-*N*-benzylbenzamide** (**16a**): Prepared, following method B from benzoyl chloride. The title product was obtained after purification by column chromatography (Hexane/EtOAc 85:15) as a white solid (980 mg, 3.90 mmol, 78%). R_f = 0.58 (Hexane/EtOAc 70:30). ^{1}H NMR (400 MHz, $CDCl_3$): (mixture of rotamers) δ 7.48 – 7.17 (m, 10H), 5.94 – 5.66 (m, 1H), 5.26 – 5.15 (m, 2H), 4.77 – 4.51 (m, 2H), 4.11 – 4.76 (m, 2H). ^{13}C NMR (101 MHz, $CDCl_3$): (mixture of rotamers) δ 172.1, 136.3, 133.1, 129.7, 128.8, 128.6, 128.5, 127.6, 127.0, 126.7, 118.0, 51.8, 50.6, 47.2, 46.8. MS (70 eV, EI): *m/z* (%): 251 (4) [M+], 210 (20), 105 (100), 91 (28), 77 (52). These data are in agreement with those reported previously in the literature, except that rotamers were observed at ambient temperature.[5]

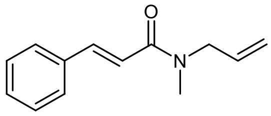

***N*-allyl-*N*-methylcinnamamide** (**17a**): Prepared, following method B from cinnamoyl chloride. The title product was obtained after purification by column chromatography (Hexane/EtOAc 75:25) as a yellow liquid (965 mg, 4.8 mmol, 96%). R_f = 0.5 (Hexane/EtOAc 50:50). ^{1}H NMR (600 MHz, $CDCl_3$): δ 7.72 – 7.68 (m, 1H), 7.54 – 7.49 (m, 2H), 7.37 – 7.33 (m, 3H), 6.91 – 6.76 (m, 1H), 5.89 – 5.78 (m, 1H), 5.27 – 5.17 (m, 2H), 4.12 – 4.05 (m, 2H), 3.11 – 3.05 (m, 3H). ^{13}C NMR (151 MHz, $CDCl_3$): (mixture of rotamers) δ 167.1, 166.4, 143.0, 142.6, 135.5, 133.1, 132.9, 129.7 (129.72), 129.7 (129.66), 128.9, 127.9 (127.94), 127.9 (127.90), 117.6, 117.5 (117.51), 117.5 (117.47), 117.0, 52.4, 50.6, 35.0, 34.4. IR (neat, cm^{-1}): 3062, 2927, 1649, 1607, 1399, 1103, 981, 922, 763, 701. MS (70 eV, EI): *m/z* (%): 201 (15) [M+], 131 (100), 110 (22), 103 (64), 77 (36). HRMS (ESI) calculated for $C_{13}H_{15}ONNa$: 224.1046 $[M+Na]^+$, found: 224.1050.

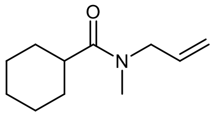

***N*-allyl-*N*-methylcyclohexanecarboxamide** (**18a**): Prepared, following method B from cyclohexanecarbonyl chloride. The title product was obtained after purification by column chromatography (Hexane/EtOAc 75:25) as a colorless oil (607 mg, 3.35 mmol, 67%). R_f = 0.61 (Hexane/EtOAc 50:50). ^{1}H NMR (600 MHz, $CDCl_3$): δ 5.81 – 5.69 (m, 1H), 5.21 – 5.09 (m, 2H), 3.98 – 3.92 (m, 2H), 2.97 – 2.90 (m, 3H), 2.51 – 2.38 (m, 1H), 1.79 – 1.67 (m, 5H), 1.55 – 1.51 (m, 2H), 1.26 – 1.25 (m, 3H). ^{13}C NMR (151 MHz, $CDCl_3$): (mixture of rotamers) δ 176.7, 176.0, 133.4 (133.44), 133.4 (133.38), 116.9, 116.5, 52.1, 50.1, 41.0, 40.8, 34.7, 33.8, 29.8, 29.3, 26.0, 25.9. IR (neat, cm^{-1}): 2926, 2854, 1637, 1449, 1404, 918. MS (70 eV, EI): *m/z* (%): 181 (48) [M+], 166 (46), 152 (24), 138 (34), 126 (100), 113 (37), 98 (87), 83 (92), 71 (50), 55 (87). HRMS (ESI) calculated for $C_{11}H_{19}ONNa$: 204.1359 $[M+Na]^+$, found: 204.1362.

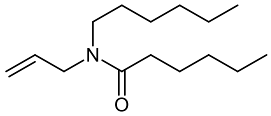

***N*-allyl-*N*-hexylhexanamide** (**19a**): Prepared, following method B (except hexyliodide was used instead of the corresponding bromide) from hexanoyl chloride. The title product was obtained after purification by column chromatography (Hexane/EtOAc 80:20) as a colorless liquid (838 mg, 3.5 mmol, 70%). R_f = 0.89 (Hexane/EtOAc 50:50). ^{1}H NMR (600 MHz, $CDCl_3$): δ 5.79 – 5.72 (m, 1H), 5.19 – 5.09 (m, 2H), 3.97 – 3.96 (m, 1H), 3.88 – 3.87 (m, 1H), 3.31 – 3.29 (m, 1H), 3.21 – 3.19 (m, 1H), 2.32 – 2.24 (m, 2H), 1.66 – 1.60 (m, 2H), 1.56 – 1.48 (m, 2H), 1.32 – 1.27 (m, 10H), 0.91 – 0.85 (m, 6H). ^{13}C NMR (151 MHz, $CDCl_3$): (mixture of rotamers) δ 173.3, 172.9, 134.0, 133.6, 116.7, 116.4, 50.3, 48.1, 47.5, 46.2, 33.2 (33.24), 33.2 (33.19), 31.9, 31.8 (31.79), 31.8 (31.76), 31.6, 29.0, 27.9, 26.8, 26.7, 25.4, 25.2, 22.7 (22.74), 22.7 (22.72), 22.7 (22.68), 14.2, 14.1. IR (neat, cm^{-1}): 2927, 2860, 1644, 1460, 1418, 917. MS (70 eV, EI): *m/z* (%): 239 (4) [M+], 224

(12), 210 (19), 196 (58), 182 (24), 168 (49), 154 (14), 140 (23), 126 (22), 112 (39), 99 (18), 70 (100). HRMS (ESI) calculated for $C_{15}H_{29}ONNa$: 262.2141 $[M+Na]^+$, found: 262.2145.

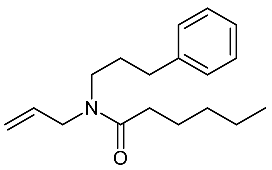

***N*-allyl-*N*-(3-phenylpropyl)hexanamide** (**20a**): Prepared, following method B (except (3-iodopropyl)benzene was used instead of the corresponding bromide) from hexanoyl chloride. The title product was obtained after purification by column chromatography (Hexane/EtOAc 90:10) as a colorless oil (848 mg, 3.1 mmol, 62%). R_f = 0.48 (Hexane/EtOAc 80:20). ^{1}H NMR (400 MHz, $CDCl_3$): δ 7.30 – 7.15 (m, 5H), 5.80 – 5.70 (m, 1H), 5.18 – 5.04 (m, 2H), 3.98 – 3.96 (m, 1H), 3.86 – 3.85 (m, 1H), 3.40 – 3.36 (m, 1H), 3.24 – 3.20 (m, 1H), 2.64 – 2.59 (m, 2H), 2.27 – 2.23 (m, 1H), 2.20 – 2.17 (m, 1H), 1.93 – 1.82 (m, 2H), 1.67 – 1.56 (m, 2H), 1.36 – 1.20 (m, 4H), 0.89 (t, *J* = 6.9Hz, 3H). ^{13}C NMR (101 MHz, $CDCl_3$): (mixture of rotamers) δ 173.4, 172.9, 141.9, 141.0, 133.9, 133.4, 128.7, 128.4 (128.44), 128.4 (128.39), 128.4 (128.37), 126.3, 125.9, 116.9, 116.5, 50.3, 48.1, 46.7, 46.0, 33.4, 33.2 (33.21), 33.2 (33.15), 33.1, 31.8, 30.4, 29.5, 25.3, 25.2, 22.6 (22.64), 22.6 (22.59), 14.1. IR (neat, cm^{-1}): 2928, 2862, 1642, 1456, 1418, 1207, 918, 745, 699. MS (70eV, EI): *m/z* (%): 273 (14) [M$^+$], 244 (11), 230 (52), 217 (19), 202 (40), 182 (22), 146 (33), 126 (30), 113 (54), 99 (22), 91 (80), 84 (21), 70 (100). HRMS (ESI) calculated for $C_{18}H_{27}ONNa$: 296.1985 $[M+Na]^+$, found: 296.1988.

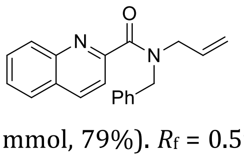

***N*-allyl-*N*-benzylquinoline-2-carboxamide** (**21a**): Prepared, following method D from quinoline-2-carboxylic acid. The title product was obtained after purification by column chromatography (Hexane/EtOAc 80:20) as a white solid (1.19 g, 3.95 mmol, 79%). R_f = 0.59 (Hexane/EtOAc 60:40). ^{1}H NMR (400 MHz, $CDCl_3$): δ 8.24 (dd, *J* = 8.4, 8.4 Hz, 1H), 8.12 – 8.02 (m, 1H), 7.86 – 7.69 (m, 3H), 7.61 – 7.55 (m, 1H), 7.45 – 7.25 (m, 5H), 6.04 – 5.91 (m, 1H), 5.28 – 5.08 (m, 2H), 4.84 – 4.80 (m, 2H), 4.16 (d, *J* = 6.1 Hz, 1H), 4.04 (d, *J* = 5.9 Hz, 1H). ^{13}C NMR (101 MHz, $CDCl_3$): (mixture of rotamers) δ 169.4, 169.1, 154.1, 154.0, 146.7, 146.6, 137.2 (137.20), 137.2 (137.16), 137.1, 134.0, 132.6, 130.1, 130.0, 129.9, 128.8, 128.6 (126.64), 128.6 (128.60), 128.1 (128.14), 128.1 (128.11), 128.0, 127.7 (127.73), 127.7 (127.68), 127.6 (127.62), 127.6 (127.55), 120.9 (120.92), 120.9 (120.86), 118.3, 118.2, 51.6, 50.9, 48.0, 47.6. IR (neat, cm^{-1}): 3059, 2974, 2908, 1618, 1468, 1408, 1150, 924, 837, 768, 710. MS (70 eV, EI): *m/z* (%): 302 (13) [M$^+$], 261 (13), 219 (9), 146 (100), 128 (77), 101 (17), 91 (40). HRMS (ESI) calculated for $C_{20}H_{18}ON_2Na$: 325.1311 $[M+Na]^+$, found: 325.1322.

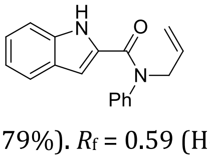

***N*-allyl-*N*-benzylquinoline-2-carboxamide** (**22a**): Prepared, following method D from quinoline-2-carboxylic acid. The title product was obtained after purification by column chromatography (Hexane/EtOAc 80:20) as a white solid (1.19 g, 3.95 mmol, 79%). R_f = 0.59 (Hexane/EtOAc 60:40). ^{1}H NMR (400 MHz, $CDCl_3$): δ 9.83 (s, 1H), 7.47 – 7.45 (m, 3H), 7.40 (d, *J* = 8.3 Hz, 1H), 7.35 – 7.29 (m, 3H), 7.23 – 7.19 (m, 1H), 7.01 – 6.98 (m, 1H), 6.02 (ddt, *J* = 17.8, 9.7, 6.2 Hz, 1H), 5.24 – 5.17 (m, 3H), 4.54 (d, *J* = 6.2 Hz, 2H). ^{13}C NMR (101 MHz, $CDCl_3$): δ 161.7, 142.6, 135.4, 132.7, 129.7, 129.5, 128.9, 128.6, 127.6, 124.5, 122.2, 120.1, 118.3, 111.7, 107.2, 53.8. MS (70 eV, EI): *m/z* (%): 276 (72) [M$^+$], 207 (51), 144 (96), 133 (100), 116 (35), 89 (91). These data are in agreement with those reported previously in the literature.[6]

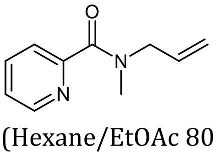

***N*-allyl-*N*-methylpicolinamide** (**23a**): Prepared, following method D from picolinic acid. The title product was obtained after purification by column chromatography (Hexane/EtOAc 20:80) as a brown liquid (440 mg, 2.5 mmol, 62%). R_f = 0.26 (Hexane/EtOAc 80:20). ^{1}H NMR (400 MHz, $CDCl_3$): δ 8.58 (dd, *J* = 8.4, 4.9 Hz, 1H), 7.81 – 7.75 (m, 1H), 7.62 (dd, *J* = 8.4, 8.4 Hz, 1H), 7.34 – 7.30 (m, 1H), 5.94 – 5.78 (m, 1H), 5.30 – 5.12 (m, 2H), 4.17 (d, *J* = 5.9 Hz,

1H), 4.01 (d, J = 5.7 Hz, 1H), 3.08 – 3.01 (m, 3H). ^{13}C NMR (101 MHz, $CDCl_3$): (mixture of rotamers) δ 169.3, 168.8, 154.7, 154.6, 148.4 (148.41), 148.4 (148.39), 137.1 (137.11), 137.1 (137.05), 133.7, 132.6, 124.5, 123.7, 123.6, 118.0, 117.7, 53.9, 50.6, 36.6, 33.4. IR (neat, cm^{-1}): 2926, 1632, 1400, 1075, 749. MS (70eV, EI): m/z (%): 176 (4) [M$^+$], 148 (13), 135 (15), 106 (19), 79 (70), 70 (100), 51 (21). HRMS (ESI) calculated for $C_{10}H_{12}ON_2Na$: 199.0842 [M+Na]$^+$, found: 199.0844.

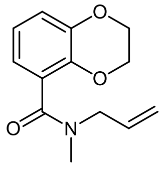

***N*-allyl-*N*-methyl-2,3-dihydrobenzo[b][1,4]dioxine-5-carboxamide** (**24a**): Prepared, following method D from 2,3-dihydrobenzo[b][1,4]dioxine-5-carboxylic acid. The title product was obtained after purification by column chromatography (Hexane/EtOAc 50:50) as a colorless oil (793.1 mg, 3.4 mmol, 68%). R_f = 0.47 (Hexane/EtOAc 30:70). ^{1}H NMR (400 MHz, $CDCl_3$): (mixture of rotamers) δ 6.88 – 6.75(m, 3H), 5.84 (rotamer, ddt, J = 17.2, 10.2, 5.6 Hz, 0.5H), 5.68 (rotamer, ddt, J = 17.1, 10.4, 5.7 Hz, 0.5H), 5.28 – 5.11 (m, 2H), 4.28 – 4.25 (m, 4H), 4.15 (d, J = 5.6 Hz, 1H), 3.76 (d, J = 5.7 Hz, 1H), 3.04 (rotamer, s, 1.5H), 2.82 (rotamer, s, 1.5H). ^{13}C NMR (101 MHz, $CDCl_3$): (mixture of rotamers) δ 168.8, 168.4, 143.7 (143.74), 143.7 (143.72), 139.8 (139.82), 139.8 (139.77), 133.3, 132.7, 126.4, 126.1, 121.6, 121.5, 119.8, 119.6, 118.0 (118.03), 118.0 (118.01), 117.8, 117.2, 64.6, 64.5, 64.3 (64.31), 64.3 (64.29), 53.7, 49.5, 35.8, 32.3. IR (neat, cm^{-1}): 2980, 2927, 2879, 1630, 1466, 1399, 1282, 1082, 793. MS (70 eV, EI): m/z (%): 233 (17) [M$^+$], 163 (100), 106 (30), 70 (15). HRMS (ESI) calculated for $C_{13}H_{15}O_3NNa$: 256.0944 [M+Na]$^+$, found: 256.0947.

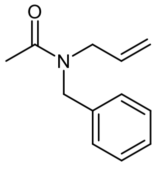

***N*-allyl-*N*-benzylacetamide** (**25a**): Prepared, following method B from acetyl chloride. The title product was obtained after purification by column chromatography (Hexane/EtOAc 60:40) as a yellow oil (605 mg, 3.2 mmol, 64%). R_f = 0.59 (Hexane/EtOAc 40:60). ^{1}H NMR (400 MHz, $CDCl_3$): δ 7.36 – 7.15 (m, 5H), 5.81 – 5.67 (m, 1H), 5.22 – 5.06 (m, 2H), 4.57 – 4.49 (m, 2H), 4.00 – 3.79 (m, 2H), 2.14 – 2.13 (m, 3H). ^{13}C NMR (101 MHz, $CDCl_3$): (mixture of rotamers) δ 171.1, 170.9, 137.7, 136.8, 133.1, 132.6, 129.1, 128.7, 128.4, 127.7, 127.5, 126.5, 117.6, 117.0, 51.0, 50.0, 48.2, 47.9, 21.8, 21.6. MS (70 eV, EI): m/z (%): 189 (13) [M$^+$], 148 (44), 106 (100), 91 (54), 56 (15). These data are in agreement with those reported previously in the literature.[2]

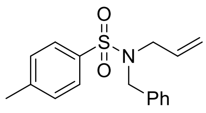

***N*-allyl-*N*-benzyl-4-methylbenzenesulfonamide** (**26a**): Prepared, following method B from 4-methylbenzene-1-sulfonyl chloride. The title product was obtained after purification by column chromatography (Hexane/EtOAc 90:10) as a colorless oil (1.1 g, 3.5 mmol, 70%). R_f = 0.71 (Hexane/EtOAc 70:30). ^{1}H NMR (400 MHz, $CDCl_3$): δ 7.75 (d, J = 8.3 Hz, 2H), 7.33 – 7.24 (m, 7H), 5.47 (ddt, J = 16.8, 10.1, 6.5 Hz, 1H), 5.08 – 4.97(m, 2H), 4.34 (s, 2H), 3.75 (d, J = 6.5 Hz, 2H), 2.44 (s, 3H). ^{13}C NMR (101 MHz, $CDCl_3$): δ 143.4, 137.7, 136.1, 132.4, 129.9, 128.7, 128.6, 127.8, 127.4, 119.5, 50.3, 49.6, 21.7. MS (70eV, EI): m/z (%): 301 (2) [M$^+$], 210 (13), 155 (13), 146 (65), 91 (100), 65 (17). These data are in agreement with those reported previously in the literature.[7]

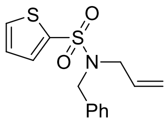

***N*-allyl-*N*-benzylthiophene-2-sulfonamide** (**27a**): Prepared, following method C from thiophene-2-sulfonyl chloride. The title product was obtained after purification by column chromatography (Hexane/EtOAc 90:10) as a green liquid (1.23 g, 4.2 mmol, 84%). R_f = 0.49 (Hexane/EtOAc 80:20). ^{1}H NMR (400 MHz, $CDCl_3$): (mixture of rotamers) δ 7.90 – 7.89(minor rotamer, m, 0.1H), 7.84 – 7.82 (minor rotamer, m, 0.1H), 7.59 (overlap, d, J = 4.0 Hz, 1.9H), 7.34 – 7.27 (overlap, m, 4.9H), 7.20 – 7.17 (minor rotamer, m, 0.1H), 7.11 (major rotamer, dd, J = 4.4, 4.4 Hz, 0.9H), 5.53 (ddt, J = 16.8, 10.2, 6.6 Hz, 1H), 5.11– 5.02(m, 2H), 4.37 (s, 2H), 3.80 (d, J = 6.6 Hz, 2H). ^{13}C NMR (101 MHz, $CDCl_3$): δ 141.2, 135.7, 132.0, 131.9, 131.7, 128.7, 128.6, 128.0, 127.5, 119.8, 50.6, 50.0. IR (neat, cm^{-1}): 3093, 3031, 2920, 1343, 1153, 923, 729. MS (70 eV, EI): m/z (%): 293 (2) [M$^+$], 202

(14), 147 (25), 144 (51), 91 (100), 65 (14). HRMS (ESI) calculated for $C_{14}H_{15}O_2NNaS_2$: 316.0436 [M+Na]$^+$, found: 316.0442.

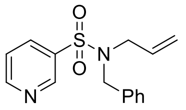

***N*-allyl-*N*-benzylpyridine-3-sulfonamide** (**28a**): Prepared, following method C from pyridine-3-sulfonyl chloride. The title product was obtained after purification by column chromatography (Hexane/EtOAc 70:30) as a yellow liquid (1.02 g, 3.55 mmol, 71%). R_f = 0.43 (Hexane/EtOAc 50:50). ^{1}H NMR (400 MHz, $CDCl_3$): δ 9.04 – 9.03 (m, 1H), 8.79 – 8.78 (m, 1H), 8.09 – 8.06 (m, 1H), 7.43 (dd, *J* = 8.0, 4.9 Hz, 1H), 7.32 – 7.22 (m, 5H), 5.49 (ddt, *J* = 16.8, 10.1, 6.5 Hz, 1H), 5.12 – 5.02 (m, 2H), 4.38 (s, 2H), 3.80 (d, *J* = 6.5 Hz, 2H). ^{13}C NMR (101 MHz, $CDCl_3$): δ 153.2, 148.2, 137.5, 135.4, 134.9, 131.7, 128.8, 128.6, 128.2, 123.8, 120.1, 50.4, 49.6. IR (neat, cm^{-1}): 3063, 3032, 2981, 2921, 2326, 2091, 1571, 1414, 1342, 1162, 1109, 924, 779, 744, 700. MS (70eV, EI): *m/z* (%): 288 (1) [M$^+$], 197 (29), 146 (42), 144 (75), 91 (100), 78 (23). HRMS (ESI) calculated for $C_{15}H_{16}O_2N_2NaS$: 311.0825 [M+Na]$^+$, found: 311.0830.

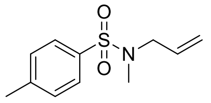

***N*-allyl-*N*,4-dimethylbenzenesulfonamide** (**29a**): Prepared, following method B from 4-methylbenzene-1-sulfonyl chloride. The title product was obtained after purification by column chromatography (Hexane/EtOAc 90:10) as a colorless oil (844 mg, 3.75 mmol, 75%). R_f = 0.57 (Hexane/EtOAc 80:20). ^{1}H NMR (400 MHz, $CDCl_3$): δ 7.67 (d, *J* = 8.3 Hz, 2H), 7.32 (d, *J* = 8.0 Hz, 2H), 5.70 (ddt, *J* = 17.3, 9.8, 6.3 Hz, 1H), 5.20 – 5.16 (m, 2H), 3.61 (d, *J* = 6.3 Hz, 2H), 2.65 (s, 3H), 2.42 (s, 3H). ^{13}C NMR (101 MHz, $CDCl_3$): δ 143.5, 134.5, 132.7, 129.8, 127.6, 119.2, 53.2, 34.3, 21.6. MS (70 eV, EI): *m/z* (%): 225 (4) [M$^+$], 198 (6), 154 (30), 91 (100), 70 (69), 65 (49), 68 (22). These data are in agreement with those reported previously in the literature.[8]

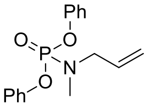

Diphenyl allyl(methyl)phosphoramidate (**30a**): Prepared, following method C from diphenyl phosphorochloridate. The title product was obtained after purification by column chromatography (Hexane/EtOAc 90:10) as a colorless liquid (1.3 g, 4.15 mmol, 83%). R_f = 0.37 (Hexane/EtOAc 80:20). ^{1}H NMR (400 MHz, $CDCl_3$): δ 7.33 (dd, *J* = 7.8, 7.8 Hz, 4H), 7.26 – 7.24 (m, 4H), 7.16 (dd, *J* = 7.3, 7.3 Hz, 2H), 5.59 (ddt, *J* = 16.5, 10.1, 6.2 Hz, 1H), 5.17 – 5.11 (m, 2H), 3.75 – 3.71 (m, 2H), 2.75 – 2.73 (m, 3H). ^{13}C NMR (101 MHz, $CDCl_3$): δ 151.0 (d, *J* = 6.5 Hz), 133.9 (m), 129.8, 125.0, 120.3 (d, *J* = 5.1 Hz), 118.3, 52.1 (d, *J* = 4.4 Hz), 33.4 (d, *J* = 3.8 Hz). IR (neat, cm^{-1}): 3069, 2909, 1591, 1486, 1270, 1190, 1162, 1017, 919, 760, 688. MS (70 eV, EI): *m/z* (%): 303 (90) [M$^+$], 276 (22), 146 (40), 94 (45), 77 (85), 70 (100). HRMS (ESI) calculated for $C_{16}H_{18}O_3NNaP$: 326.0917 [M+Na]$^+$, found: 326.0922.

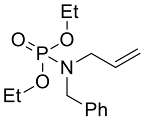

diethyl allyl(benzyl)phosphoramidate (**31a**): Prepared, following method C from diethyl phosphorochloridate. The title product was obtained after purification by column chromatography (Hexane/EtOAc 70:30) as a colorless liquid (1.35 g, 4.75 mmol, 95%). R_f = 0.45 (Hexane/EtOAc 50:50). ^{1}H NMR (400 MHz, $CDCl_3$): δ 7.33 – 7.24 (m, 5H), 5.75 (ddt, *J* = 16.7, 10.2, 6.4 Hz, 1H), 5.19 – 5.08 (m, 2H), 4.20 (d, *J* = 9.7 Hz, 2H), 4.15 – 3.98 (m, 4H), 3.51 – 3.47 (m, 2H), 1.32 (t, *J* = 7.1 Hz, 6H). ^{13}C NMR (101 MHz, $CDCl_3$): δ 138.0 (d, *J* = 3.7 Hz), 134.4, 128.7, 128.5, 127.4, 118.3, 62.4 (d, *J* = 5.4 Hz), 48.7 (d, *J* = 4.9 Hz), 47.7 (d, *J* = 4.1 Hz), 16.3 (d, *J* = 7.3 Hz). MS (70 eV, EI): *m/z* (%): 283 (3) [M$^+$], 242 (98), 214 (18), 192 (16), 186 (62), 136 (28), 91 (100). These data are in agreement with those reported previously in the literature.[9]

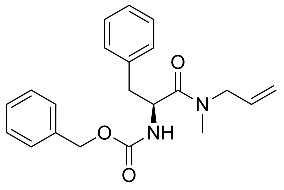

(*S*)-benzyl (1-(allyl(methyl)amino)-1-oxo-3-phenylpropan-2-yl) carbamate (**32a**): Prepared, following method E from (*S*)-phenylalanine. The title product was obtained after purification by column chromatography (Hexane/EtOAc 70:30) as a colorless oil (1.23 g, 3.5 mmol, 70%). R_f = 0.33 (Hexane/EtOAc 70:30). ^{1}H NMR (400 MHz, $CDCl_3$): (mixture of rotamers) δ 7.37 – 7.10 (m, 10H), 5.76 – 5.67 (m, 1H), 5.61 (major rotamer, ddt, *J* = 16.4, 10.3, 6.1 Hz, 0.6H), 5.49 (minor rotamer, ddt, *J* = 15.9, 10.4, 5.3 Hz, 0.4H), 5.14 – 4.99 (m, 4H), 4.89 (major rotamer, q, *J* = 7.9 Hz, 0.6H), 4.82 (minor rotamer, q, *J* = 8.0 Hz, 0.4H), 3.98 – 3.93 (major rotamer, m, 0.6H), 3.87 – 3.82 (minor rotamer, m, 0.4H), 3.65 – 3.64 (m, 1H), 3.04 – 2.94 (m, 2H), 2.85 (minor rotamer, s, 1.2H), 2.62 (major rotamer, s, 1.8H). ^{13}C NMR (101 MHz, $CDCl_3$): (mixture of rotamers) δ 171.7, 171.3, 155.8, 155.7, 136.5, 136.4, 136.2, 132.3, 129.6 (129.63), 129.6 (129.56), 128.6, 128.2, 128.1 (128.08), 128.1 (128.05), 127.2, 127.1, 118.0, 117.6, 66.9, 52.2, 52.1, 52.0, 50.5, 40.3, 40.1, 34.5, 33.7. IR (neat, cm^{-1}): 3270, 3062, 2945, 1707, 1628, 1540, 1252, 1022, 743. MS (70 eV, EI): *m/z* (%): 352 (1) [M$^+$], 245 (2), 202 (100), 131 (66), 110 (33), 98 (67). HRMS (ESI) calculated for $C_{21}H_{24}O_3N_2Na$: 375.1679 [M+Na]$^+$, found: 375.1683.

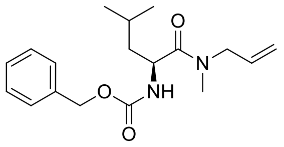

(*S*)-benzyl (1-(allyl(methyl)amino)-4-methyl-1-oxopentan-2-yl) carbamate (**33a**): Prepared, following method E from (*S*)-leucine. The title product was obtained after purification by column chromatography (Hexane/EtOAc 70:30) as a colorless oil (1.00 g, 3.15 mmol, 63%). R_f = 0.4 (Hexane/EtOAc 70:30). ^{1}H NMR (400 MHz, $CDCl_3$): (mixture of rotamers) δ 7.35 – 7.27 (m, 5H), 5.86 – 5.77 (rotamer, m, 0.5H), 5.71 (rotamer, ddt, *J*=16.2, 11.3, 5.9 Hz, 0.5H), 5.63 – 5.55 (m, 1H), 5.27 – 5.04 (m, 4H), 4.75 – 4.64 (m, 1H), 4.09 – 4.01 (m, 1H), 3.95 – 3.90 (m, 1H), 3.02 (rotamer, s, 1.5H), 2.92 (rotamer, s, 1.5H), 1.78 – 1.68 (m, 1H), 1.59 – 1.49 (m, 1H), 1.42 – 1.33(m, 1H), 1.02 – 0.86 (m, 6H). ^{13}C NMR (101 MHz, $CDCl_3$): (mixture of rotamers) δ 173.1, 172.6, 156.4, 156.2, 136.5, 132.5, 132.3, 128.6, 128.2, 128.1, 117.8 (117.80), 117.8 (117.76), 66.9, 52.1, 50.5, 49.5, 49.3, 43.0, 42.7, 34.6, 33.9, 24.7 (24.71), 24.7 (24.69), 23.6, 21.8. IR (neat, cm^{-1}): 3282, 3064, 3033, 2955, 2871, 1712, 1639, 1529, 1223, 1044, 738. HRMS (ESI) calculated for $C_{18}H_{26}O_3N_2Na$: 341.1836 [M+Na]$^+$, found: 341.1841.

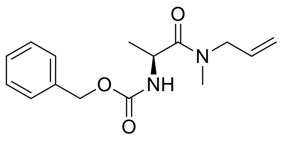

(*S*)-benzyl (1-(allyl(methyl)amino)-1-oxopropan-2-yl)carbamate (**34a**): Prepared, following method E from (*S*)-alanine. The title product was obtained after purification by column chromatography (Hexane/EtOAc 50:50) as a colorless oil (981 mg, 3.55 mmol, 71%). R_f = 0.5 (Hexane/EtOAc 50:50). ^{1}H NMR (400 MHz, $CDCl_3$): δ 7.34 – 7.27 (m, 5H), 5.90 – 5.66 (m, 2H), 5.26 – 5.07 (m, 4H), 4.71 – 4.59 (m, 1H), 4.03 – 3.90 (m, 2H), 2.99 – 2.92 (m, 3H), 1.35 – 1.31 (m, 3H). ^{13}C NMR (101 MHz, $CDCl_3$): (mixture of rotamers) δ 172.8, 172.3, 155.7, 155.6, 136.5 (136.54), 136.5 (136.51), 132.3 (132.30), 132.3 (132.25), 128.6, 128.1, 128.0, 117.7 (117.72), 117.7 (117.66), 66.8, 52.0, 50.4, 47.1, 46.8, 34.5, 33.8, 19.6, 19.0. IR (neat, cm^{-1}): 3410, 3293, 3033, 2980, 2937, 1712, 1640, 1493, 1245, 1057, 740. HRMS (ESI) calculated for $C_{15}H_{20}O_3N_2Na$: 299.1366 [M+Na]$^+$, found: 299.1371.

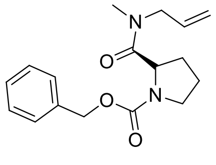

(*R*)-benzyl 2-(allyl(methyl)carbamoyl)pyrrolidine-1-carboxylate (**35a**): Prepared, following method E from (*R*)-proline. The title product was obtained after purification by column chromatography (Hexane/EtOAc 20:80) as a colorless oil (635 mg, 2.1 mmol, 42%). R_f = 0.5 (EtOAc). ^{1}H NMR (400 MHz, $CDCl_3$): δ 7.35 – 7.30 (m, 5H), 5.90 – 5.47 (m, 1H), 5.27 – 4.94 (m, 4H), 4.74 – 4.46 (m, 1H), 4.19 – 3.72 (m, 2H), 3.71 – 3.48 (m, 2H), 3.05 – 2.82 (m, 3H), 2.23 – 2.02 (m, 2H), 1.94 – 1.81 (m, 2H). ^{13}C NMR (101 MHz, $CDCl_3$): (mixture of rotamers) δ 172.8, 172.7, 172.2, 172.1, 155.0, 154.3, 137.0, 136.9 (136.90), 136.9 (136.85), 136.7, 132.8,

132.7, 132.2, 128.5 (128.53), 128.5 (128.46), 128.1, 128.0 (128.01), 128.0 (127.99), 128.0 (127.96), 127.9 (127.90), 127.9 (127.85), 117.4, 117.3, 117.1, 117.0, 67.3, 67.1, 67.0, 57.1, 56.8, 56.6, 56.4, 52.1, 51.7, 50.6, 50.5, 47.4, 47.3, 46.9, 46.8, 34.7, 34.4, 34.3, 34.1, 31.3, 30.7, 30.4, 29.7, 24.5, 24.4, 23.7. IR (neat, cm^{-1}): 2954, 2881, 1701, 1653, 1412, 1352, 1116, 743. MS (70 eV, EI): *m/z* (%): 302 (1) [M^+], 204 (27), 160 (25), 91 (100). HRMS (ESI) for $C_{17}H_{22}O_3N_2Na$: 325.1523 [$M+Na]^+$, found: 325.1528.

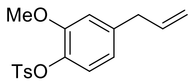

4-allyl-2-methoxyphenyl 4-methylbenzenesulfonate (**37a**): Under Argon atmosphere eugenol (821 mg, 5.0 mmol, 1.0 equiv.), *para*-toluenesulfonyl chloride (1.1 g, 6.0 mmol, 1.2 equiv.), triethylamine (0.7 mL, 6.0 mmol, 1.2 equiv.) and 4-dimethylaminopyridine (DMAP, 103 mg, 0.85 mmol, 17 mol%) were dissolved in DCM (15 mL). The reaction was stirred at ambient temperature overnight and after completion (as confirmed by TLC), the reaction mixture was quenched by the addition of water (20 mL). The aqueous phase was extracted with EtOAc (3 x 20 mL) and the combined organic layer dried over Na_2SO_4 and concentrated under vacuum. The obtained crude was purified by column chromatography (hexane/ethyl acetate 90:10) to afford the product in 95% yield (914 mg, 2.8 mmol) as a white solid. R_f = 0.3 (hexane/ethyl acetate 90:10). ^{1}H NMR (400 MHz, $CDCl_3$): δ 7.75 (d, *J* = 8.2 Hz, 2H), 7.29 (d, *J* = 8.1 Hz, 2H), 7.03 (d, *J* = 8.1 Hz, 1H), 6.71 – 6.65 (m, 2H), 5.92 (ddt, *J* = 16.9, 10.4, 6.7 Hz, 1H), 5.10 – 5.04 (m, 2H), 3.55 (s, 3H), 3.34 (d, *J* = 6.7 Hz, 2H), 2.44 (s, 3H). ^{13}C NMR (100 MHz, $CDCl_3$): δ 151.6, 144.8, 140.2, 136.6 (br), 133.4, 129.3, 128.6, 123.7, 120.5, 116.4, 112.9, 55.5, 40.0, 21.7. MS (EI): m/z (%): 318 (9) [M^+], 163 (100), 135 (8), 103 (25), 91 (38), 65 (20). Spectroscopic data match with those reported previously in the literature.[8]

4. Synthesis and characterization data for the starting materials for site selective coupling

4.1. General Experimental Procedures

Method F: Synthesis of aryl fluorosulfates[10]

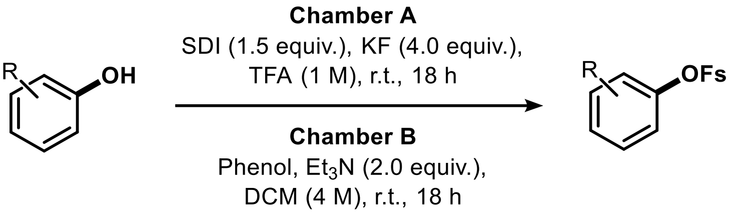

Chamber A of an oven-dried two-chamber reactor was charged with 1,1'-sulfonyldiimidazole (1.49 g, 7.5 mmol, 1.5 equiv.) and KF (1.16 g, 20.0 mmol, 4.0 equiv.) and chamber B was filled with the substituted phenol (5.0 mmol) dissolved in DCM (20 mL) and treated with triethylamine (1.39 mL, 10.0 mmol, 2.0 equiv.). Trifluoroacetic acid (5 mL, 3.26 M) was added carefully with a syringe through the septum in chamber A (Caution! Instant gas formation!) and the reaction mixture stirred at room temperature for 24 hours. After completion the reactor was gently opened inside a well ventilated fume hood to release the residual gas. The reaction was stirred for another 15 min to make sure that all of the remaining sulfuryl fluoride has evaporated. Chamber A was neutralized with saturated $NaHCO_3$ and the reaction mixture of chamber B was transferred to a 100 mL round-bottomed flask rinsing with DCM (5 × 5 mL). The combined organic phases were dried over Na_2SO_4 and volatiles were removed under reduced pressure. The obtained crude was subjected to column chromatography on silica for further purification.

Method G: Synthesis of aryl triflates[11]

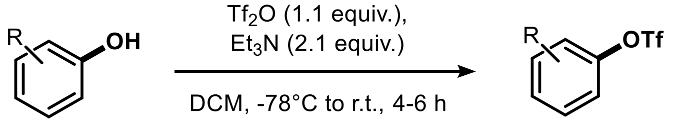

Under Ar atmosphere, NEt_3 (1.5 mL, 10.5 mmol, 2.1 equiv.) was added slowly to substituted phenol (5.0 mmol, 1.0 equiv) dissolved in 10 mL dry DCM. Then the soluton was cooled to -78°C and Tf_2O (0.93 mL, 5.5 mmol, 1.1 equiv) was added dropwise to the mixture. Then the reaction mixture was slowly allowed to warm to room temperature and stirred overnight. After completion, the reaction mixture was cooled in an ice bath and was slowly quenched with water (10-15 mL). The aqueous phase was extracted with diethyl ether (3 x 20 mL) and the combined organic phases were dried over Na_2SO_4 and volatiles were removed under reduced pressure. The obtained crude was subjected to column chromatography on silica for further purification.

4.2. Characterization data of starting materials for selective coupling

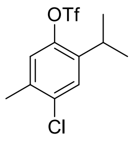

4-chloro-2-isopropyl-5-methylphenyl trifluoromethanesulfonate (**45a**): Prepared, following method G from 4-chloro-2-isopropyl-5-methylphenol. The title product was obtained after purification by column chromatography (Pentane/Et_2O 95:5) as a colourless liquid (1.23 g, 3.9 mmol, 79%). R_f = 0.70 (Pentane/Et_2O 90:10). ^1H NMR (400 MHz, $CDCl_3$) δ 7.34 (s, 1H), 7.10 (s, 1H), 3.22 (hept, *J* = 6.8 Hz, 1H), 2.36 (s, 3H), 1.25 (d, *J* = 6.9 Hz, 6H). ^{13}C NMR (101 MHz, $CDCl_3$): δ 145.1, 140.4, 135.8, 134.6, 128.3, 123.5, 118.7 (q, *J* = 320.1 Hz), 27.2, 23.2, 20.0. ^{19}F NMR (376 MHz, $CDCl_3$): δ −73.88 (s). IR (neat, cm^{-1}): 2971, 1485, 1416, 1211, 1138, 1095, 845. HRMS (EI) calculated for $C_{11}H_{12}O_3ClF_3S$: 316.0148 $[M]^+$, found: 316.0145.

Aryl triflates (**46a**, **48a**, **50a**)[11] and aryl fluorosulfates (**49a**, **51a**)[10] were prepared as previously reported and analyses matched previously reported data.

5. General procedure for olefin isomerization and characterization data of *E*-olefins

5.1. General procedure for olefin isomerization

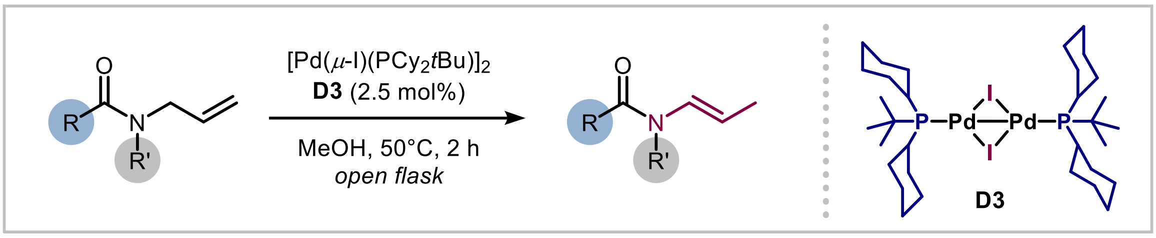

In air, $[Pd(\mu\text{-}I)(PCy_2tBu)]_2$ **D3** (4.9 mg, 5 μmol, 0.025 equiv.) was weighed into a 5 mL vial and a solution of terminal olefin (0.2 mmol, 1.0 equiv.) dissolved in MeOH (1.0 mL) was added. The resulting mixture was stirred at 50°C in an open flask for 2 hours. Once the reaction time was completed, the crude mixture was directly loaded onto a short silica column for purification to obtain the *E*-olefin product.

Gram scale reaction

In air, $[Pd(\mu\text{-}I)(PCy_2tBu)]_2$ **D3** (100 mg, 0.1 mmol, 0.025 equiv.) and *N*-allyl-*N*-(2,2,2-trifluoroethyl) benzamide (**14a**) (1.0 g, 4.1 mmol, 1.0 equiv.) were dissolved in MeOH (20 mL). After reaction completion (2 hours), MeOH was evaporated under reduced pressure and the obtained mixture was filtered through a short pad of silica using Hexane/EtOAc 80:20 as eluent to afford *(E)*-*N*-(prop-1-en-1-yl)-*N*-(2,2,2-trifluoroethyl)benzamide (**14**) as a yellow liquid (962 mg, 3.95 mmol, 96%, *E:Z* >99:1, based on GC-MS).

5.2. Characterization data of *E*-olefins

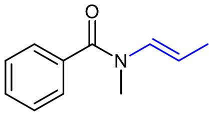

***(E)*-*N*-methyl-*N*-(prop-1-en-1-yl)benzamide** (**1**): Prepared, following the general procedure from *N*-allyl-*N*-methylbenzamide (**1a**). The title product was obtained after purification by column chromatography (Hexane/EtOAc 80:20) as a colorless oil (33.6 mg, 0.19 mmol, 96%, *E:Z* 98:2, based on GC-MS). R_f = 0.45 (Hexane/EtOAc 80:20). ^{1}H NMR (400 MHz, $CDCl_3$, 50°C): δ 7.42– 7.38 (m, 5H), 6.71 – 6.41 (m, 1H), 5.08 – 5.03 (m, 1H), 3.19 (s, 3H), 1.65 (s, 3H). ^{13}C NMR (101 MHz, $CDCl_3$, 50°C): δ 170.1, 136.1, 130.1, 128.5, 127.9 (127.94), 127.9 (127.93), 106.2, 29.8, 15.3. MS (70 eV, EI): *m/z* (%): 175 (15) [M+], 160 (12), 105 (100), 77 (71), 51 (22). These data are in agreement with those reported previously in the literature.[12]

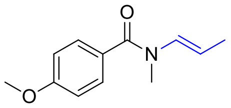

***(E)*-4-methoxy-*N*-methyl-*N*-(prop-1-en-1-yl)benzamide** (**2**): Prepared, following the general procedure from *N*-allyl-4-methoxy-*N*-methylbenzamide (**2a**). The title product was obtained after purification by column chromatography (Hexane/EtOAc 70:30) as a yellow low-melting solid (40.1 mg, 0.2 mmol, 95%, *E:Z* 98:2, based on GC-MS). R_f = 0.53 (Hexane/EtOAc 70:30). ^{1}H NMR (400 MHz, $CDCl_3$): δ 7.44– 7.41 (m, 2H), 6.92– 6.89 (m, 2H), 6.73– 6.53 (m, 1H), 5.07– 4.99 (m, 1H), 3.83 (s, 3H), 3.18 (s, 3H), 1.65 (s, 3H). ^{13}C NMR (101 MHz, $CDCl_3$): δ 169.9, 161.1, 130.1 (130.14), 130.1 (130.12), 127.9, 113.7, 105.6, 55.5 (55.463), 55.5 (55.456), 15.5. IR (neat, cm^{-1}): 3351, 2936, 1606, 1618, 1554, 1504, 1250, 1175, 1026, 842, 765. MS (70 eV, EI): *m/z* (%): 205 (9) [M^+], 135 (100), 92 (22), 77 (22). HRMS (ESI) calculated for $C_{12}H_{15}O_2NNa$: 228.0995 $[M+Na]^+$, found: 228.1001.

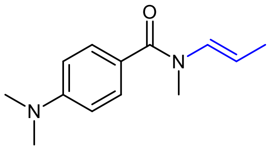

***(E)*-4-(dimethylamino)-*N*-methyl-*N*-(prop-1-en-1-yl)benzamide** (**3**): Prepared, following the general procedure from *N*,*N*-allyl-4-(dimethylamino)-*N*-methylbenzamide (**3a**). The title product was obtained after purification by column chromatography (Hexane/EtOAc 70:30) as a yellow low-melting solid (47.1 mg, 0.21 mmol, 94%, *E:Z* 98:2, based on GC-MS). R_f = 0.42 (Hexane/EtOAc 70:30). ^{1}H NMR (400 MHz, $CDCl_3$): δ 7.42 (d, *J* = 8.8 Hz, 2H), 6.98- 6.76 (m, 1H), 6.67 (d, *J* = 8.8 Hz, 2H), 4.98 (dq, *J* = 13.4, 6.6 Hz, 1H), 3.19 (s, 3H), 3.01 (s, 6H), 1.67 (d, *J*= 6.4 Hz, 3H). ^{13}C NMR (101 MHz, $CDCl_3$): δ 170.6, 151.8, 131.4, 130.4, 122.4, 111.1, 104.7, 40.3, 15.5. IR (neat, cm^{-1}): 3453, 3363, 2898, 1603, 1515, 1301, 1066, 827, 765. MS (70 eV, EI): *m/z* (%): 218 (10) [M$^+$], 148 (100), 104 (8), 77 (10). HRMS (ESI) calculated for $C_{13}H_{18}ON_2Na$: 241.1311 [M+Na]$^+$, found: 241.1322.

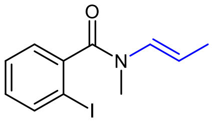

***(E)*-2-iodo-*N*-methyl-*N*-(prop-1-en-1-yl)benzamide** (**4**): Prepared, following the general procedure from *N*-allyl-2-iodo-*N*-methylbenzamide (**4a**). The title product was obtained after purification by column chromatography (Hexane/EtOAc 80:20) as a yellow low-melting solid (56.2 mg, 0.19 mmol, 95%, *E:Z* 98:2, based on GC-MS). R_f = 0.64 (Hexane/EtOAc 70:30). ^{1}H NMR (400 MHz, $CDCl_3$): (mixture of rotamers) δ 7.85 – 7.81 (m, 1H), 7.49 – 7.37 (m, 1.3H, overlap with minor rotamer), 7.22 – 7.19 (m, 1H), 7.12 – 7.06 (m, 1H), 6.13 (major rotamer, dd, *J* = 13.9, 1.6 Hz, 0.7H), 5.19 (minor rotamer, dq, *J* = 14.7, 6.7 Hz, 0.3H), 5.08 (major rotamer, dq, *J* = 13.5, 6.7 Hz, 0.7H), 3.25 (major rotamer, s, 2H), 2.91 (minor rotamer, s, 1H), 1.80 (minor rotamer, dd, *J* = 6.7, 1.6 Hz, 1H), 1.57 (major rotamer, dd, *J* = 6.7, 1.2 Hz, 2H). ^{13}C NMR (101 MHz, $CDCl_3$): (mixture of rotamers) δ 169.3, 168.8, 142.5, 142.1, 139.4, 139.2, 130.5 (130.52), 130.5 (130.45), 129.4, 128.5 (128.53), 128.5 (128.45), 127.8, 127.4, 127.2, 108.6, 107.1, 92.9, 92.4, 34.0, 29.7, 15.6, 15.4. IR (neat, cm^{-1}): 3282, 2926, 1634, 1370, 1317, 1075, 1016, 936, 765. MS (70 eV, EI): *m/z* (%): 301 (7) [M$^+$], 231 (100), 202 (29), 174 (49), 76 (19). HRMS (ESI) calculated for $C_{11}H_{12}ONINa$: 323.9856 [M+Na]$^+$, found: 323.9867.

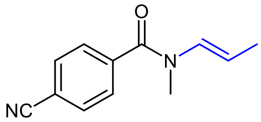

***(E)*-4-cyano-*N*-methyl-*N*-(prop-1-en-1-yl)benzamide** (**5**): Prepared, following the general procedure from *N*-allyl-4-cyano-*N*-methylbenzamide (**5a**). The title product was obtained after purification by column chromatography (Hexane/EtOAc 75:25) as a white low-melting solid (38.2 mg, 0.19 mmol, 93%, *E:Z* 97:3, based on GC-MS). R_f = 0.41 (Hexane/EtOAc 70:30). ^{1}H NMR (400 MHz, $CDCl_3$): (mixture of rotamers) δ 7.74 – 7.71 (m, 2H), 7.54 (d, *J* = 8.2 Hz, 2H), 7.45 – 7.38 (minor rotamer, m, 0.2H), 6.31 (major rotamer, d, *J* = 13.9 Hz, 0.8H), 5.24 – 5.07(m, 1H), 3.25 (major rotamer, s, 2.2H), 3.02 (minor rotamer, s, 0.8H), 1.81 – 1.80 (major rotamer, m, 2.2H), 1.62 – 1.60 (minor rotamer, m, 0.8H). ^{13}C NMR (101 MHz, $CDCl_3$): δ 168.1, 140.1, 132.6, 130.0, 128.7, 118.2, 113.9, 108.0, 30.8, 15.4. IR (neat, cm^{-1}): 2927, 1637, 1377, 1066, 944, 849, 751. MS (70 eV, EI): *m/z* (%): 200 (16) [M$^+$], 185 (23), 130 (100), 102 (50). HRMS (ESI) calculated for $C_{12}H_{12}ON_2Na$: 223.0842 [M+Na]$^+$, found: 223.0844.

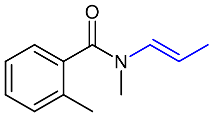

***(E)*-*N*,2-dimethyl-*N*-(prop-1-en-1-yl)benzamide** (**6**): Prepared, following the general procedure from *N*-allyl-*N*,2-dimethylbenzamide (**6a**). The title product was obtained after purification by column chromatography (Hexane/EtOAc 75:25) as a white low-melting solid (33.1 mg, 0.18 mmol, 98%, *E:Z* >99:1, based on GC-MS). R_f = 0.49 (Hexane/EtOAc 80:20). ^{1}H NMR (400 MHz, $CDCl_3$): (mixture of rotamers) δ 7.51 (minor rotamer, d, *J* = 14.5 Hz, 0.3H), 7.32 – 7.27(m, 1H), 7.24– 7.16(m, 3H), 6.22 (major rotamer, dd, *J* = 13.9, 1.5 Hz, 0.7H), 5.18 – 5.09 (minor rotamer, m, 0.3H), 5.01 (major rotamer, dq, *J* = 13.4, 6.7 Hz, 0.7H), 3.25 (major rotamer, s, 2.1H), 2.90 (minor rotamer, s, 0.9H), 2.27 – 2.24 (m, 3H), 1.80 (minor rotamer,dd, *J* = 6.6, 1.6 Hz, 0.9H), 1.55 (major rotamer, dd, *J* = 6.7, 1.6 Hz, 2.1H). ^{13}C NMR (101 MHz, $CDCl_3$): (mixture of rotamers) δ 170.3, 169.6, 136.4,

136.1, 134.6, 134.3, 130.5, 129.9, 129.3, 129.2, 127.4, 126.6, 126.1, 107.6, 106.0, 33.9, 29.4, 19.1, 19.0, 15.6, 15.4. IR (neat, cm^{-1}): 3333, 2922, 1628, 1546, 1300, 748. MS (70eV, EI): *m/z* (%): 189 (17) [M^+], 119 (100), 91 (47), 65 (16). HRMS (ESI) calculated for $C_{12}H_{16}ON$: 190.1226 [M+H]$^+$, found: 190.1232.

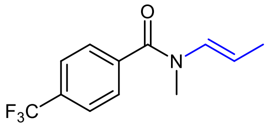

***(E)*-*N*-methyl-*N*-(prop-1-en-1-yl)-4-(trifluoromethyl)benzamide** (**7**): Prepared, following the general procedure from *N*-allyl-*N*-methyl-4-(trifluoromethyl)benzamide (**7a**). The title product was obtained after purification by column chromatography (Hexane/EtOAc 85:15) as a white solid (45.3 mg, 0.19 mmol, 92%, *E:Z* 97:3, based on GC-MS). R_f = 0.49 (Hexane/EtOAc 80:20). M.p. = 54 – 56°C. ^{1}H NMR (400 MHz, $CDCl_3$): (mixture of rotamers) δ 7.69 (d, *J* = 7.9 Hz, 2H), 7.55 (d, *J* = 7.7 Hz, 2H), 7.48 – 7.38 (minor rotamer, m, 0.3H), 6.38 (major rotamer, d, *J* = 13.5 Hz, 0.7H), 5.19 – 5.07 (m, 1H), 3.26- 3.03 (m, 3H), 1.80 – 1.60(m, 3H). ^{13}C NMR (101 MHz, $CDCl_3$): δ 168.7, 139.4, 132.0 (q, *J* = 33.0 Hz), 130.3, 127.9 – 127.6 (m), 125.6 (d, *J* = 3.2 Hz), 123.8 (d, *J* = 272.2 Hz), 107.3, 30.6, 15.4. ^{19}F NMR (376 MHz, $CDCl_3$): δ −62.99 (s). IR (neat, cm^{-1}): 3074, 2929, 1619, 1317, 1130, 1065, 856. MS (70 eV, EI): *m/z* (%): 243 (17) [M^+], 228 (21), 173 (100), 145 (70). HRMS (ESI) calculated for $C_{12}H_{12}ONF_3Na$: 266.0763 [M+Na]$^+$, found: 266.0772. *Note:* The expected quartet of the CF_3 carbon in ^{13}C appears as an apparent doublet.

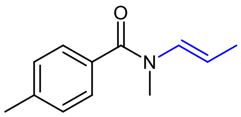

***(E)*-*N*,4-dimethyl-*N*-(prop-1-en-1-yl)benzamide** (**8**): Prepared, following the general procedure from *N*-allyl-*N*,4-dimethylbenzamide (**8a**). The title product was obtained after purification by column chromatography (Hexane/EtOAc 80:20) as a white solid (37.2 mg, 0.2 mmol, 95%, *E:Z* 97:3, based on GC-MS). R_f = 0.41 (Hexane/EtOAc 80:20). M.p. = 140 – 142°C. ^{1}H NMR (400 MHz, $CDCl_3$): δ 7.34 (d, *J* = 8.0 Hz, 2H), 7.22 – 7.20 (m, 2H), 6.62 – 6.46 (m, 1H), 5.03 – 5.01 (m, 1H), 3.21 (s, 3H), 2.39 (s, 3H), 1.62 (s, 3H). ^{13}C NMR (101 MHz, $CDCl_3$): δ 170.5, 140.4, 132.9, 131.2, 129.1, 128.1, 105.4, 30.5, 21.6, 15.5. MS (70 eV, EI): *m/z* (%): 189 (17) [M^+], 119 (100), 91 (33), 65 (12). These data are in agreement with those reported previously in the literature.[13]

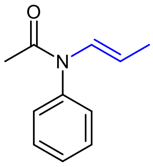

***(E)*-*N*-phenyl-*N*-(prop-1-en-1-yl)acetamide** (**9**): Prepared, following the general procedure from *N*-allyl-*N*-phenylacetamide (**9a**). The title product was obtained after purification by column chromatography (Hexane/EtOAc 80:20) as a white solid (33.2 mg, 0.19 mmol, 94%, *E:Z* 98:2, based on GC-MS). R_f = 0.67 (Hexane/EtOAc 60:40). M.p. = 69 – 71°C. ^{1}H NMR (400 MHz, $CDCl_3$): δ 7.48 – 7.37 (m, 4H), 7.16 – 7.14 (m, 2H), 4.38 (dq, *J* = 13.1, 6.4 Hz, 1H), 1.82 (s, 3H), 1.60 (dd, *J* = 6.7, 1.5 Hz, 3H). ^{13}C NMR (101 MHz, $CDCl_3$): δ 168.3, 140.3, 130.0, 129.1, 129.0, 128.6, 109.4, 23.3, 15.2. MS (70 eV, EI): *m/z* (%): 175 (34) [M^+], 160 (6), 132 (100), 117 (21), 104 (22), 77 (39), 51 (15). These data are in agreement with those reported previously in the literature.[14]

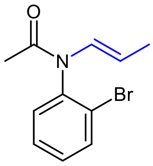

***(E)*-*N*-(2-bromophenyl)-*N*-(prop-1-en-1-yl)acetamide** (**10**): Prepared, following the general procedure from *N*-allyl-*N*-(2-bromophenyl)acetamide (**10a**). The title product was obtained after purification by column chromatography (Hexane/EtOAc 80:20) as a white solid (48.5 mg, 0.19 mmol, 95%, *E:Z* 98:2, based on GC-MS). R_f = 0.35 (Hexane/EtOAc 80:20). M.p. = 68 – 70°C. ^{1}H NMR (400 MHz, $CDCl_3$): (mixture of rotamers) δ 7.72 (major rotamer, dd, *J* = 8.0, 1.3 Hz, 0.9H), 7.66 (minor rotamer, d, *J* = 8.7 Hz, 0.1H), 7.45 – 7.24 (m, 4H), 4.45 (minor rotamer, dq, *J* = 13.4, 6.6 Hz, 0.1H), 4.30 (major rotamer, dq, *J* = 13.5, 6.7 Hz, 0.9H), 1.81 (s, 3H), 1.62 (dd, *J* = 6.7, 1.6 Hz, 3H). ^{13}C NMR (101 MHz, $CDCl_3$): δ 168.0, 139.2, 134.3, 131.0, 130.3, 129.2, 127.3, 124.1, 108.7, 22.9, 15.2. IR (neat, cm^{-1}): 2921, 1670, 1470, 1365, 1304, 951, 749. MS (70 eV, EI):

m/z (%): 255 [^{81}Br-M$^+$], 253 [^{79}Br-M$^+$], 213 (62), 211 (64), 184 (10), 174 (38), 155 (70), 132 (100), 117 (32). HRMS (ESI) calculated for $C_{11}H_{12}ONBrNa$: 275.9995 [M+Na]$^+$, found: 276.0005.

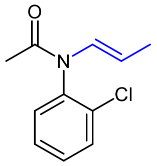

***(E)*-*N*-(2-bromophenyl)-*N*-(prop-1-en-1-yl)acetamide** (**11**): Prepared, following the general procedure from *N*-allyl-*N*-(2-bromophenyl)acetamide (**11a**). The title product was obtained after purification by column chromatography (Hexane/EtOAc 80:20) as a white solid (41.8 mg, 0.2 mmol, 97%, *E:Z* 98:2, based on GC-MS). R_f = 0.27 (Hexane/EtOAc 85:15). M.p. = 79 – 81°C. ^{1}H NMR (400 MHz, $CDCl_3$): (mixture of rotamers) δ 7.57– 7.55 (m, 1H), 7.41 – 7.37 (m, 3H), 7.28 – 7.25 (m, 1H), 4.49 (minor rotamer, dq, *J* = 13.5, 6.7 Hz, 0.1H), 4.33 (major rotamer, dq, *J* = 13.5, 6.7 Hz, 0.9H), 1.82 (s, 3H), 1.63 (dd, *J* = 6.7, 1.7 Hz, 3H). ^{13}C NMR (101 MHz, $CDCl_3$): δ 168.1, 137.6, 133.7, 131.0, 130.2, 128.5, 127.4, 108.6, 22.7, 15.1. IR (neat, cm^{-1}): 2922, 1672, 1477, 1305, 1304, 1127, 953, 754. MS (70 eV, EI): *m/z* (%): 209 (26) [M$^+$], 187 (86), 132 (100), 111 (24), 75 (20). HRMS (ESI) calculated for $C_{11}H_{12}ONClNa$: 232.0500 [M+Na]$^+$, found: 232.0507.

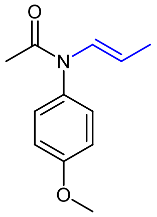

***(E)*-*N*-(4-methoxyphenyl)-*N*-(prop-1-en-1-yl)acetamide** (**12**): Prepared, following the general procedure from *N*-allyl-*N*-(4-methoxyphenyl)acetamide (**12a**). The title product was obtained after purification by column chromatography (Hexane/EtOAc 70:30) as a white low-melting (40.1 mg, 0.2 mmol, 98%, *E:Z* >99:1, based on GC-MS). R_f = 0.41 (Hexane/EtOAc 70:30). ^{1}H NMR (400 MHz, $CDCl_3$, 50°C): δ 7.43 (d, *J* = 14.0 Hz, 1H), 7.06 – 7.04 (m, 2H), 6.95 (d, *J* = 8.8 Hz, 2H), 4.42 (dq, *J* = 13.8, 6.7 Hz, 1H), 3.83 (s, 3H), 1.82 (s, 3H), 1.60 (d, *J* = 6.7 Hz, 3H). ^{13}C NMR (151 MHz, $CDCl_3$, 50°C): δ 168.7, 159.6, 133.2, 130.0, 129.5, 115.2, 109.0, 55.6, 23.1, 15.0. MS (70 eV, EI): *m/z* (%): 205 (59) [M$^+$], 163 (100), 148 (94), 121 (20), 92 (18), 77 (20), 64 (15). These data are in agreement with those reported previously in the literature.[15]

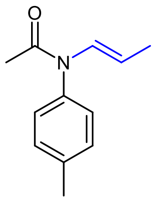

***(E)*-*N*-(prop-1-en-1-yl)-*N*-(*p*-tolyl)acetamide** (**13**): Prepared, following the general procedure from *N*-allyl-*N*-(p-tolyl)acetamide (**13a**). The title product was obtained after purification by column chromatography (Hexane/EtOAc 80:20) as a white solid (38.5 mg, 0.19 mmol, 96%, *E:Z* 98:2, based on GC-MS). R_f = 0.41 (Hexane/EtOAc 80:20). M.p. = 68 – 70°C. ^{1}H NMR (400 MHz, $CDCl_3$): δ 7.43 (d, *J* = 14.3 Hz, 1H), 7.25 (d, *J* = 8.1 Hz, 2H), 7.02 (d, *J* = 8.2 Hz, 2H), 4.40 (dq, *J* = 13.6, 6.7 Hz, 1H), 2.39 (s, 3H), 1.82 (s, 3H), 1.60 (dd, *J* = 6.7, 1.5 Hz, 3H) .^{13}C NMR (101 MHz, $CDCl_3$): δ 168.5, 138.5, 137.6, 130.6, 129.1, 128.7, 109.2, 23.3, 21.3, 15.2. MS (70 eV, EI): *m/z* (%): 189 (51) [M$^+$], 147 (100), 132 (54), 91 (34), 65 (21). These data are in agreement with those reported previously in the literature.[16]

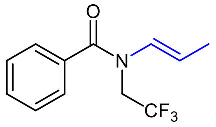

***(E)*-*N*-(prop-1-en-1-yl)-*N*-(2,2,2-trifluoroethyl)benzamide** (**14**): Prepared, following the general procedure from *N*-allyl-*N*-(2,2,2-trifluoroethyl)benzamide (**14a**). The title product was obtained after purification by column chromatography (Hexane/EtOAc 80:20) as a yellow liquid (47.8 mg, 0.2 mmol, 99%, *E:Z* >99:1, based on GC-MS). R_f = 0.57 (Hexane/EtOAc 80:20). ^{1}H NMR (400 MHz, $CDCl_3$): δ 7.52 – 7.42 (m, 5H), 6.45 (d, *J* = 12.1 Hz, 1H), 5.21 (dq, *J* = 13.5, 6.7 Hz, 1H), 4.45 (q, *J* = 8.8 Hz, 2H), 1.61 (dd, *J* = 6.6, 1.7 Hz, 3H). ^{13}C NMR (101 MHz, $CDCl_3$): δ 170.7, 134.6, 131.0, 130.1, 128.6, 128.5, 124.5 (d, *J* = 280.9Hz), 109.2, 45.6 (q, *J*=32.1 Hz), 15.3. ^{19}F NMR (376 MHz, $CDCl_3$): δ −68.42 (s). IR (neat, cm^{-1}): 1652, 1392, 1369, 1260, 1152, 1100, 941, 720. MS (70 eV, EI): *m/z* (%): 243 (9) [M$^+$], 105 (100), 77 (52). HRMS (ESI) calculated for $C_{12}H_{12}ONF_3Na$: 266.0763 [M+Na]$^+$, found: 266.0773.

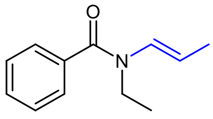

***(E)*-*N*-ethyl-*N*-(prop-1-en-1-yl)benzamide** (**15**): Prepared, following the general procedure from *N*-allyl-*N*-ethylbenzamide (**15a**). The title product was obtained after purification by column chromatography (Hexane/EtOAc 80:20) as a colorless oil (36.7 mg, 0.19 mmol, 96%, *E:Z* >99:1, based on GC-MS). R_f = 0.49 (Hexane/EtOAc 80:20). ^{1}H NMR (400 MHz, $CDCl_3$): δ 7.48 – 7.36 (m, 5H), 6.36 – 6.32 (m, 1H), 5.08 – 5.06 (m, 1H), 3.82 – 3.53 (m, 2H), 1.61 (s, 3H), 1.22 (s, 3H). ^{13}C NMR (101 MHz, $CDCl_3$): δ 169.8, 136.2, 130.0, 129.4, 128.5, 128.0, 106.0, 38.5, 15.6, 12.3. MS (70eV, EI): *m/z* (%): 189 (13) [M$^+$], 174 (13), 105 (100), 77 (57), 51 (14). These data are in agreement with those reported previously in the literature.[12]

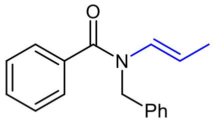

***(E)*-*N*-benzyl-*N*-(prop-1-en-1-yl)benzamide** (**16**): Prepared, following the general procedure from *N*-allyl-*N*-benzylbenzamide (**16a**). The title product was obtained after purification by column chromatography (Hexane/EtOAc 85:15) as a white low-melting solid (51.8 mg, 0.2 mmol, 98%, *E:Z* >99:1, based on GC-MS). R_f = 0.58 (Hexane/EtOAc 80:20). ^{1}H NMR (400 MHz, $CDCl_3$): δ 7.49 – 7.22 (m, 10H), 6.71 – 6.32 (m, 1H), 5.04 – 4.96 (m, 3H), 1.54 (s, 3H). ^{13}C NMR (101 MHz, $CDCl_3$): δ 170.4, 137.4, 135.7, 130.3, 130.0, 128.7, 128.5, 128.2, 127.1, 126.9, 107.5, 47.4, 15.5. MS (70eV, EI): *m/z* (%): 251 (14) [M$^+$], 160 (20), 105 (100), 91 (50), 77 (60). These data are in agreement with those reported previously in the literature.[12]

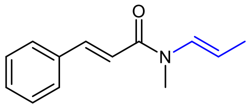

***N*-methyl-*N*-(*(E)*-prop-1-en-1-yl)cinnamamide** (**17**): Prepared, following the general procedure from *N*-allyl-*N*-methylcinnamamide (**17a**). The title product was obtained after purification by column chromatography (Hexane/EtOAc 80:20) as a yellow low-melting solid (37.8 mg, 0.19 mmol, 90%, *E:Z* 93:7, based on GC-MS). R_f = 0.37 (Hexane/EtOAc 80:20). ^{1}H NMR (400 MHz, $CDCl_3$): (mixture of rotamers) δ 7.75 – 7.65 (m, 1.2H, overlap with minor rotamer), 7.55 – 7.53 (m, 2H), 7.40 – 7.33 (m, 3H), 6.94 (d, *J* = 15.5 Hz, 1H), 6.78 (major rotamer, d, *J* = 13.7 Hz, 0.8H), 5.14 (dq, *J* = 14.2, 7.5 Hz, 1H), 3.22 – 3.18 (m, 3H), 1.77 (d, *J* = 6.6 Hz, 3H). ^{13}C NMR (101 MHz, $CDCl_3$): (mixture of rotamers) δ 165.5, 143.8, 143.1, 135.3, 129.8, 129.2, 128.9, 128.0, 117.8, 109.2, 107.0, 32.7, 31.2, 15.7. IR (neat, cm^{-1}): 3282, 3032, 2918, 1641, 1601, 1388, 1368, 1282, 1119, 1003, 944, 756, 685. MS (70 eV, EI): *m/z* (%): 201 (18) [M$^+$], 131 (100), 103 (70), 77 (45). HRMS (ESI) calculated for $C_{13}H_{15}ONNa$: 224.1046 [M+Na]$^+$, found: 224.1051.

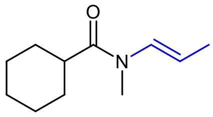

***(E)*-*N*-methyl-*N*-(prop-1-en-1-yl)cyclohexanecarboxamide** (**18**): Prepared, following the general procedure from *N*-allyl-*N*-methylcyclohexanecarboxamide (**18a**). The title product was obtained after purification by column chromatography (Hexane/EtOAc 75:25) as a colorless liquid (33.1 mg, 0.18 mmol, 87%, *E:Z* 93:7, based on GC-MS). R_f = 0.6 (Hexane/EtOAc 80:20). ^{1}H NMR (400 MHz, $CDCl_3$): (mixture of rotamers) δ 7.36 – 7.30 (minor rotamer, m, 0.3H), 6.69 (major rotamer, d, *J* = 13.7 Hz, 0.7H), 5.02 (dq, *J* = 14.1, 7.4 Hz, 1H), 3.05 (s, 3H), 2.63 – 2.57 (m, 1H), 1.82 – 1.68 (m, 8H), 1.57 – 1.48 (m, 2H), 1.35 – 1.24 (m, 3H). ^{13}C NMR (101 MHz, $CDCl_3$): δ 174.7, 129.2, 107.0, 41.4, 30.5, 29.5, 26.1, 26.0, 15.6. IR (neat, cm^{-1}): 3323, 2926, 2854, 1637, 1449, 1404, 918. MS (EI): m/z (%): 181 (28) [M$^+$], 83 (62), 71 (100), 55 (49). HRMS (ESI) calculated for $C_{11}H_{19}ONNa$: 204.1359 [M+Na]$^+$, found: 204.1364.

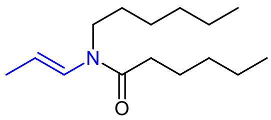

***(E)*-*N*-hexyl-*N*-(prop-1-en-1-yl)hexanamide** (**19**): Prepared, following the general procedure from *N*-allyl-*N*-hexylhexanamide (**19a**). The title product was obtained after purification by column chromatography (Hexane/EtOAc 97:3) and preparative HPLC (Pentane/EtOAc 1:1) as a colourless liquid (42.6 mg, 0.18 mmol, 89%, *E:Z* 93:7, based on GC-MS). R_f = 0.59 (Hexane/EtOAc 90:10). ^{1}H NMR (600 MHz, $CDCl_3$): (mixture of rotamers)

δ 7.18 (minor rotamer, d, J = 14.6 Hz, 0.3H), 6.46 (major rotamer, d, J = 13.9 Hz, 0.7H), 5.06 – 4.94 (m, 1H), 3.56 – 3.53 (major rotamer, m, 1.4H), 3.43 – 3.40 (minor rotamer, m, 0.6H), 2.39 – 2.34 (m, 2H), 1.71 (d, J = 6.6 Hz, 3H), 1.68 – 1.60 (m, 2H), 1.56 – 1.47 (m, 2H), 1.36 – 1.24 (m, 10H), 0.90 – 0.85 (m, 6H). ^{13}C NMR (151 MHz, $CDCl_3$): (mixture of rotamers) δ 171.4, 171.2, 128.1, 126.6, 107.4, 105.5, 45.2, 43.3, 34.1, 33.8, 31.8, 31.7 (31.70), 31.7 (31.69), 31.6, 29.8, 28.0, 27.0, 26.8 (26.84), 26.8 (26.81), 25.1, 24.9, 22.7 (22.73), 22.7 (22.65), 22.6, 15.8, 15.7, 14.2, 14.1. IR (neat, cm^{-1}): 2929, 2862, 1642, 1465, 917. MS (70 eV, EI): *m/z* (%): 239 (10) [M^+], 224 (11), 196 (27), 168 (10), 140 (11), 112 (100) 70 (76). HRMS (ESI) calculated for $C_{15}H_{29}ONNa$: 262.2141 $[M+Na]^+$, found: 262.2149.

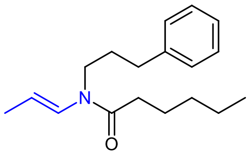

***(E)*-*N*-(3-phenylpropyl)-*N*-(prop-1-en-1-yl)hexanamide** (**20**): Prepared, following the general procedure from *N*-allyl-*N*-(3-phenylpropyl)hexanamide (**20a**). The title product was obtained after purification by column chromatography (Hexane/EtOAc 95:5) and preparative HPLC (Pentane/EtOAc 1:1) as a colourless liquid (50.1 mg, 0.18 mmol, 91%, *E:Z* 96:4, based on GC-MS). R_f = 0.35 (Hexane/EtOAc 90:10). ^{1}H NMR (400 MHz, $CDCl_3$): (mixture of rotamers) δ 7.33– 7.16 (overlap with minor rotamer, m, 5.2H), 6.47– 6.43 (major rotamer, m, 0.8H), 4.98 – 4.83 (m, 1H), 3.64 – 3.60 (major rotamer, m, 1.5H), 3.45 – 3.41 (minor rotamer, m, 0.5H), 2.67 – 2.60(m, 2H), 2.41 – 2.37 (major rotamer, m, 1.5H), 2.25 – 2.21 (minor rotamer, m, 0.5H), 1.97 – 1.81(m, 2H), 1.70 – 1.61 (overlapped rotamers, m, 4.6H), 1.39 – 1.22 (overlapped rotamers, m, 4.4H), 0.92 – 0.87 (m, 3H). ^{13}C NMR (101 MHz, $CDCl_3$): δ 171.6, 141.8, 128.7, 128.5, 128.0, 126.0, 107.8, 43.0, 34.1, 33.5, 31.7, 28.5, 24.9, 22.6, 15.8, 14.1. IR (neat, cm^{-1}): 2928, 2861, 1645, 1406, 1158, 744, 698. MS (70 eV, EI): *m/z* (%): 273 (12) [M^+], 244 (6), 230 (23), 146 (100), 113 (18), 91 (60), 70 (80). HRMS (ESI) calculated for $C_{18}H_{27}ONNa$: 296.1985 $[M+Na]^+$, found: 296.1993.

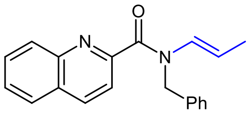

***(E)*-*N*-benzyl-*N*-(prop-1-en-1-yl)quinoline-2-carboxamide** (**21**): Prepared, following the general procedure from *N*-allyl-*N*-benzylquinoline-2-carboxamide (**21a**). The title product was obtained after purification by column chromatography (Hexane/EtOAc 70:30) as a white solid (56.1 mg, 0.19 mmol, 92%, *E:Z* 96:4, based on GC-MS). R_f = 0.42 (Hexane/EtOAc 50:50). M.p. = 125 – 127°C. ^{1}H NMR (600 MHz, $CDCl_3$): (mixture of rotamers) δ 8.31 – 8.29 (major rotamer, m, 0.6H), 8.19 (dd, J = 8.5, 8.5 Hz, 1H), 7.92 – 7.88 (m, 1H), 7.80 – 7.62 (m, 3H), 7.57 – 7.55 (minor rotamer, m, 0.4H), 7.49 (minor rotamer, d, J = 14.5, 0.4H), 7.39 – 7.21 (m, 5H), 6.76 (major rotamer, dd, J = 13.9, 1.7 Hz, 0.6H), 5.20 – 5.05 (m, 3H), 1.73 – 1.71 (minor rotamer, m, 1H), 1.49 – 1.48 (major rotamer, m, 2H). ^{13}C NMR (151 MHz, $CDCl_3$): (mixture of rotamers) δ 168.1, 167.7, 153.8, 153.5, 147.0, 146.5, 137.4, 137.3, 137.2, 137.0, 130.3, 130.2, 130.1, 129.9, 129.1, 128.7, 128.5, 128.2 (128.18), 128.2 (128.15), 127.9, 127.8, 127.7, 127.2, 127.1 (127.10),127.1 (127.05), 126.9, 126.4, 121.0, 120.8, 110.4, 108.6, 50.8, 47.3, 15.8, 15.5. IR (neat, cm^{-1}): 2926, 1636, 1407, 1160, 740. MS (70 eV, EI): *m/z* (%): 302 (12) [M^+], 287 (16), 211 (7), 146 (100), 128 (80), 91 (66). HRMS (ESI) calculated for $C_{20}H_{18}ON_2Na$: 325.1311 $[M+Na]^+$, found: 325.1320.

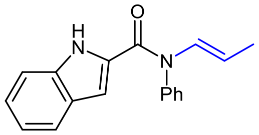

***(E)*-*N*-phenyl-*N*-(prop-1-en-1-yl)-1*H*-indole-2-carboxamide** (**22**): Prepared, following the general procedure from *N*-allyl-*N*-phenyl-1*H*-indole-2-carboxamide (**22a**). The title product was obtained after purification by a small column chromatography (Hexane/EtOAc 60:40) as a white solid (55.9 mg, 0.2 mmol, 99.9%, *E:Z* >99:1, based on GC-MS). R_f = 0.67 (Hexane/EtOAc 70:30). M.p. = 217 – 219°C. ^{1}H NMR (400 MHz, $CDCl_3$): δ 9.82(s, 1H), 7.74 – 7.70 (m, 1H), 7.58 – 7.56 (m, 3H), 7.42 – 7.33 (m, 4H), 7.24 (dd, J = 7.6, 7.6 Hz, 1H), 7.03 (dd, J = 7.5, 7.5 Hz, 1H), 5.14 (s, 1H), 4.69 (dq, J = 13.7, 6.8 Hz, 1H), 1.75 (dd, J = 6.8, 1.6 Hz, 3H). ^{13}C NMR (101 MHz, $CDCl_3$): δ 159.7, 139.6, 135.7, 130.2, 130.1, 129.9, 129.3 (129.33), 129.3

(129.29), 127.8, 124.9, 122.4, 120.4, 111.7, 111.1, 107.8, 15.5. IR (neat, cm^{-1}): 3279, 1608, 1514, 1396, 1318, 1272, 806, 741, 694. MS (70 eV, EI): *m/z* (%): 276 (21) [M^+], 248 (14), 144 (100), 133 (52), 116 (24), 89 (74), 77 (26). HRMS (ESI) calculated for $C_{18}H_{16}ON_2Na$: 299.1155 $[M+Na]^+$, found: 299.1165.

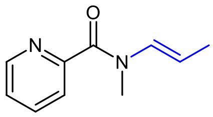

***(E)-N*-methyl-*N*-(prop-1-en-1-yl)picolinamide** (**23**): Prepared, following the general procedure from *N*-allyl-*N*-methylpicolinamide (**23a**). The title product was obtained after purification by column chromatography (Hexane/EtOAc 40:60) as a brownliquid (32.8 mg, 0.19 mmol, 91%, *E:Z* 98:2, based on GC-MS). R_f = 0.42 (Hexane/EtOAc 50:50). ^1H NMR (400 MHz, $CDCl_3$): (mixture of rotamers) δ 8.64 – 8.60 (m, 1H), 7.82 (dd, *J* = 7.5, 7.5 Hz, 1H), 7.66 – 7.62 (m, 1H), 7.45 (minor rotamer, d, *J* = 14.4 Hz, 0.4H), 7.38 – 7.34(m, 1H), 6.66 (major rotamer dd, *J* = 14.0, 1.9 Hz, 0.6H), 5.21 (minor rotamer, dq, *J* = 13.6, 6.6 Hz, 0.4H), 5.09 (major rotamer, dq, *J* = 13.4, 6.7 Hz, 0.6H), 3.28 (major rotamer, s, 2H), 3.13 (minor rotamer, s, 1H), 1.80 (minor rotamer, d, *J* = 6.7 Hz, 1H), 1.61 (major rotamer, dd, *J* = 6.6, 1.5 Hz, 2H). ^{13}C NMR (101 MHz, $CDCl_3$): (mixture of rotamers) δ 167.7, 154.3, 154.1, 148.7, 148.5, 137.2 (137.23), 137.2 (137.19), 130.4, 128.2, 124.8, 124.4, 124.2, 108.2, 106.7, 34.7, 30.5, 15.6, 15.5. IR (neat, cm^{-1}): 2925, 1637, 1372, 1075, 748. MS (70 eV, EI): *m/z* (%): 176 (8) [M^+], 161 (73), 133 (8), 106 (13), 78 (100), 70 (31), 51 (30). HRMS (ESI) calculated for $C_{10}H_{12}ON_2Na$: 199.0842 $[M+Na]^+$, found: 199.0847.

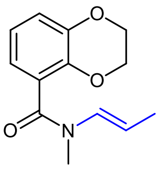

***(E)-N*-methyl-*N*-(prop-1-en-1-yl)-2,3-dihydrobenzo[*b*][1,4]dioxine-5-carboxamide** (**24**): Prepared, following the general procedure from *N*-allyl-*N*-methyl-2,3-dihydrobenzo[*b*][1,4]dioxine-5-carboxamide (**24a**). The title product was obtained after purification by column chromatography (Hexane/EtOAc 70:30) as a white solid (56.1 mg, 0.2 mmol, 94%, *E:Z* 99:1, based on GC-MS). R_f = 0.32 (Hexane/EtOAc 70:30). M.p. = 94 – 96°C. ^1H NMR (400 MHz, $CDCl_3$): (mixture of rotamers) δ 7.47 (minor rotamer, dd, *J* = 14.5, 1.9Hz, 0.4H), 6.92 – 6.76(m, 3H), 6.35 (major rotamer, d, *J* = 13.9 Hz, 0.6H), 5.13 (minor rotamer, dq, *J* = 13.6, 6.6 Hz, 0.4H), 5.00 (major rotamer, dq, *J* = 13.5, 6.6 Hz, 0.6H), 4.26 (s, 4H), 3.23 (major rotamer, s, 2H), 2.97 (minor rotamer, s, 1H), 1.78 (minor rotamer, dd, *J* = 6.7, 1.6 Hz, 1H), 1.59 (major rotamer, dd, *J* = 6.6, 1.6 Hz, 2H). ^{13}C NMR (101 MHz, $CDCl_3$): (mixture of rotamers) δ 167.3, 166.8, 143.8, 143.7, 140.2, 140.1, 130.2, 127.6, 125.9, 125.5, 121.7, 121.6, 120.3, 120.2, 118.5, 107.4, 105.7, 64.6 (64.61), 64.6 (64.57), 64.4, 64.3, 33.9, 29.7, 15.6, 15.5. IR (neat, cm^{-1}): 3265, 2923, 1621, 1589, 1546, 1553, 1281, 1549, 1220, 1085. MS (70 eV, EI): *m/z* (%): 233 (13) [M^+], 163 (100), 107 (30), 79 (8). HRMS (ESI) calculated for $C_{13}H_{15}O_3NNa$: 256.0944 $[M+Na]^+$, found: 256.0950.

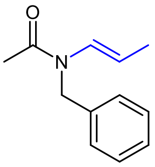

***(E)-N*-benzyl-*N*-(prop-1-en-1-yl)acetamide** (**25**): Prepared, following the general procedure from *N*-allyl-*N*-benzylacetamide (**25a**). The title product was obtained after purification by column chromatography (Hexane/EtOAc 75:25) as a yellow liquid (38.6 mg, 0.2 mmol, 90%, *E:Z* 93:7, based on GC-MS). R_f = 0.26 (Hexane/EtOAc 85:15). ^1H NMR (400 MHz, $CDCl_3$): (mixture of rotamers) δ 7.38 – 7.14 (overlap with minor rotamer, m, 5.3H), 6.55 (major rotamer, dd, *J* = 13.8, 1.5 Hz, 0.7H), 5.01 (major rotamer, dq, *J* = 13.3, 6.6 Hz, 0.7H), 4.92 (minor rotamer, dq, *J* = 13.4, 6.7 Hz, 0.3H), 4.85 (major rotamer, s, 1.4H), 4.74 (minor rotamer, s, 0.6H), 2.29 (major rotamer, s, 2.1H), 2.14 (minor rotamer, s, 0.9H), 1.67 – 1.63 (m, 3H). ^{13}C NMR (101 MHz, $CDCl_3$): (mixture of rotamers) δ 169.4, 169.3, 137.4, 136.5, 129.0, 128.6, 128.5, 127.4, 127.0, 126.9, 125.7, 109.1, 107.3, 49.8, 46.9, 22.4 (22.41), 22.4 (22.35), 15.7, 15.5. MS (70 eV, EI): *m/z* (%): 189 (25) [M^+], 174 (10), 146 (22), 132 (17), 91 (100), 65 (13). These data are in agreement with those reported previously in the literature except that rotamers were observed.[15]

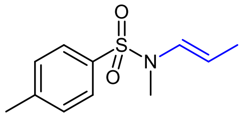

***(E)*-*N*,4-dimethyl-*N*-(prop-1-en-1-yl)benzenesulfonamide** (**26**): Prepared, following the general procedure from *N*-allyl-*N*,4-dimethylbenzenesulfonamide (**26a**). The title product was obtained after purification by column chromatography (Hexane/EtOAc 80:20) as a white solid (43.0 mg, 0.19 mmol, 95%, *E:Z* >98:2, based on GC-MS). R_f = 0.58 (Hexane/EtOAc 80:20). M.p. = 46 – 48°C. ^{1}H NMR (400 MHz, $CDCl_3$): δ 7.62 (d, *J* = 7.8 Hz, 2H), 7.29 (d, *J* = 8.0 Hz, 2H), 6.69 (dq, *J* = 14.0, 1.4 Hz, 1H), 4.71 (dq, *J* = 13.3, 6.6 Hz, 1H), 2.81 (s, 3H), 2.41 (s, 3H), 1.67 – 1.65 (m, 3H). ^{13}C NMR (101 MHz, $CDCl_3$): δ 143.5, 134.6, 129.6, 128.2, 127.0, 106.3, 32.2, 21.5, 15.1. MS (70 eV, EI): *m/z* (%): 225 (9) [M$^+$], 155 (10), 140 (9), 120 (54), 91 (100), 70 (45). Spectroscopic data match with those reported previously in the literature.[8]

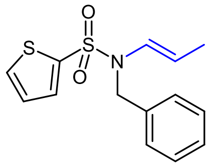

***(E)*-*N*-benzyl-*N*-(prop-1-en-1-yl)thiophene-2-sulfonamide** (**27**): Prepared, following the general procedure from *N*-allyl-*N*-benzylthiophene-2-sulfonamide (**27a**). The title product was obtained after purification by column chromatography (Hexane/EtOAc 50:50) as a white solid (56.1 mg, 0.2 mmol, >99%, *E:Z* >99:1, based on GC-MS). R_f = 0.49 (Hexane/EtOAc 80:20). M.p. = 78 – 80°C. ^{1}H NMR (400 MHz, $CDCl_3$): δ 7.87 – 7.78 (m, 2H), 7.74 – 7.55 (m, 1H), 7.44 – 7.27 (m, 5H), 7.09- 6.79 (m, 1H), 5.29 – 5.16 (m, 1H), 5.06 (s, 2H), 1.67 (d, *J* = 6.8 Hz, 3H). ^{13}C NMR (101 MHz, $CDCl_3$): (mixture of rotamers) δ 163.6, 141.0, 138.7, 137.3, 137.1, 128.8, 127.3, 127.0, 127.9, 126.3, 125.1, 124.9, 122.5, 111.1, 49.3, 15.6. IR (neat, cm^{-1}): 3277, 3059, 2920, 1622, 1516, 1400, 1228, 1186, 975, 942, 752, 726. MS (70 eV, EI): *m/z* (%): 293 (1) [M$^+$], 202 (7), 147 (20), 144 (35), 91 (100), 65 (18). HRMS (ESI) calculated for $C_{14}H_{15}O_2NNaS_2$: 316.0436 [M+Na]$^+$, found: 316.0440.

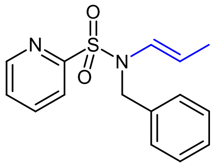

***(E)*-*N*-benzyl-*N*-(prop-1-en-1-yl)pyridine-2-sulfonamide** (**28**): Prepared, following the general procedure from *N*-allyl-*N*-benzylpyridine-3-sulfonamide (**28a**). The title product was obtained after purification by column chromatography (Hexane/EtOAc 40:60) as a white solid (57.5 mg, 0.2 mmol, 94%, *E:Z* 95:5, based on GC-MS). R_f = 0.58 (Hexane/EtOAc 50:50). M.p. = 68 – 70°C. ^{1}H NMR (400 MHz, $CDCl_3$): δ 9.00 (s, 1H), 8.79 – 8.77 (m, 1H), 8.03 – 8.01 (m, 1H), 7.42 (dd, *J* = 8.0, 4.9 Hz, 1H), 7.29 – 7.22 (m, 5H), 6.54 (d, *J* = 14.1 Hz, 1H), 4.85 (dq, *J* = 13.5, 6.7 Hz, 1H), 4.52 (s, 2H), 1.56 (dd, *J* = 6.7, 1.6 Hz, 3H). ^{13}C NMR (101 MHz, $CDCl_3$): δ 153.4, 147.9, 135.9, 135.3, 134.6, 128.7, 127.7, 127.2, 125.7, 123.9, 110.7, 50.0, 15.5. IR (neat, cm^{-1}): 2925, 1343, 1160, 1050, 929, 823, 734, 694. MS (70 eV, EI): *m/z* (%): 288 (3) [M$^+$], 197 (7), 91 (100), 78 (11), 65 (12). HRMS (ESI) calculated for $C_{15}H_{16}O_2N_2NaS$: 311.0825 [M+Na]$^+$, found: 311.0834.

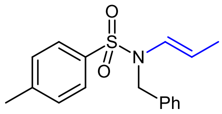

***(E)*-*N*-benzyl-4-methyl-*N*-(prop-1-en-1-yl)benzenesulfonamide** (**29**): Prepared, following the general procedure from *N*-allyl-*N*-benzyl-4-methylbenzenesulfonamide (**29a**). The title product was obtained after purification by column chromatography (Hexane/EtOAc 85:15) as a white solid (57.1 mg, 0.19 mmol, 92%, *E:Z* 95:5, based on GC-MS). R_f = 0.58 (Hexane/EtOAc 80:20). M.p. = 112 – 114°C. ^{1}H NMR (400 MHz, $CDCl_3$, 50°C): (mixture of rotamers) δ 7.75 (minor rotamer, d, *J* = 7.9 Hz, 0.2H), 7.67 (major rotamer, d, *J* = 8.0 Hz, 1.8H), 7.29 – 7.17 (m, 7H), 6.56 (d, *J* = 14.1 Hz, 1H), 4.74 (dq, *J* = 13.8, 6.6 Hz, 1H), 4.47 (s, 2H), 2.41 (s, 3H), 1.54 (d, *J* = 6.5 Hz, 3H). ^{13}C NMR (101 MHz, $CDCl_3$, 50°C): δ 143.7, 136.6, 136.2, 129.9, 128.6, 128.0, 127.5, 127.2, 126.8, 108.9, 50.1, 21.6, 15.4. MS (EI): m/z (%): 301 (3) [M$^+$], 237 (5), 146 (9), 120 (15), 91 (100). These data are in agreement with those reported previously in the literature.[17]

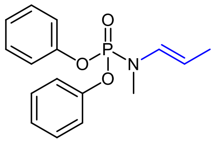

***(E)*-diphenyl methyl(prop-1-en-1-yl)phosphoramidate** (**30**): Prepared, following the general procedure from diphenyl allyl(methyl)phosphoramidate (**30a**). The title product was obtained after purification by column chromatography (Hexane/EtOAc 50:50) as a yellow low-melting solid (60.1 mg, 0.2 mmol, 98%, *E:Z* >99:1, based on GC-MS). R_f = 0.51 (Hexane/EtOAc 80:20). ^{1}H NMR (400 MHz, $CDCl_3$): δ 7.36 – 7.16 (m, 10H), 6.64 – 6.59 (m, 1H), 4.75 (dq, *J* = 13.4, 6.5 Hz, 1H), 2.92 – 2.90 (m, 3H), 1.68 (d, *J* = 6.5 Hz, 3H). ^{13}C NMR (101 MHz, $CDCl_3$): 150.6 (d, *J* = 6.5 Hz), 129.9, 129.8 (d, *J* = 8.3 Hz), 125.3 (d, *J* = 1.0 Hz), 120.2 (d, *J* = 5.0 Hz), 102.4 (d, *J* = 10.9 Hz), 31.9 (d, *J* = 3.3 Hz), 15.2. IR (neat, cm^{-1}): 3237, 1590, 1486, 1190, 922, 761, 688. MS (70 eV, EI): *m/z* (%): 303 (100) [M$^+$], 288 (16), 210 (13), 146 (43), 77 (50), 70 (76). HRMS (ESI) calculated for $C_{16}H_{19}O_3NP$: 304.1097 [M+H]$^+$, found: 304.1107.

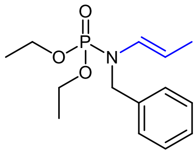

***(E)*-diethyl benzyl(prop-1-en-1-yl)phosphoramidate** (**31**): Prepared, following the general procedure from diethyl allyl(benzyl)phosphoramidate (**31a**). The title product was obtained after purification by column chromatography (Hexane/EtOAc 50:50) as a yellow low-melting solid (62.0 mg, 0.22 mmol, 99%, *E:Z* >99:1, based on GC-MS). R_f = 0.5 (Hexane/EtOAc 50:50). ^{1}H NMR (400 MHz, $CDCl_3$): δ 7.32 – 7.19 (m, 5H), 6.38 – 6.33 (m, 1H), 4.61 (dq, *J* = 13.3, 6.4 Hz, 1H), 4.51 – 4.48 (m, 2H), 4.16 – 3.97 (m, 4H), 1.55 (dd, *J* = 6.5, 1.6 Hz, 3H), 1.29 (t, *J* = 7.0 Hz, 6H). ^{13}C NMR (101 MHz, $CDCl_3$): δ 138.0 (d, *J* = 2.5 Hz), 128.8 (d, *J* = 6.0 Hz), 128.4, 126.9, 126.8, 103.2 (d, *J* = 9.9 Hz), 63.0 (d, *J* = 5.3 Hz), 48.7 (d, *J* = 4.1 Hz), 16.2 (d, *J* = 7.1 Hz), 15.4. MS (70eV, EI): *m/z* (%): 283 (3) [M$^+$], 242 (98), 214 (18), 192 (16), 186 (62), 136 (28), 91 (100). These data are in agreement with those reported previously in the literature.[18]

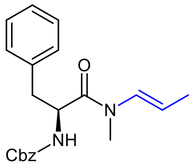

(*S,E*)-benzyl (1-(methyl(prop-1-en-1-yl)amino)-1-oxo-3-phenylpropan-2-yl) carbamate (**32**): Prepared, following the general procedure from (*S*)-benzyl (1-(allyl(methyl)amino)-1-oxo-3-phenylpropan-2-yl)carbamate (**32a**). The title product was obtained after purification by column chromatography (Hexane/EtOAc 60:40) as a white solid (61.4 mg, 0.17 mmol, 90%, *E:Z* 95:5, based on GC-MS). R_f = 0.26 (Hexane/EtOAc 80:20). M.p. = 56 – 58°C. $[\alpha]_D^{30}$ +66.6 (c 1.0, $CHCl_3$); 99.6% ee as determined by HPLC (IC, 9:1 *n*-hexane/*i*-PrOH, 1.0 mL/min). ^{1}H NMR (400 MHz, $CDCl_3$): (mixture of rotamers) δ 7.35 – 7.09 (overlap with minor rotamer, m, 10.3H), 6.39 (major rotamer, d, *J* = 13.6 Hz, 0.7H), 5.73 – 5.71 (m, 1H), 5.10 – 4.94 (m, 4H), 3.04 – 2.94 (m, 4H), 2.75 (s, 1H), 1.70 (minor rotamer, dd, *J* = 6.6, 1.7 Hz, 1H), 1.60 (major rotamer, dd, *J* = 6.8, 1.6 Hz, 2H). ^{13}C NMR (101 MHz, $CDCl_3$): (mixture of rotamers) δ 169.9, 169.6, 155.7, 136.6, 136.5 (136.52), 136.5 (136.47), 136.3, 136.0, 135.9, 129.5 (129.53), 129.5 (129.45), 128.6, 128.5, 128.2, 128.1, 128.0, 127.6, 127.2, 127.1, 110.6, 108.4, 66.9, 52.8, 52.7, 40.1, 39.7, 32.3, 31.2, 15.5 (15.52), 15.5 (15.46). IR (neat, cm^{-1}): 3328, 2921, 1710, 1633, 1535, 1247, 1046, 737, 692. MS (70 eV, EI): *m/z* (%): 352 (1) [M$^+$], 245 (3), 202 (100), 131 (68), 110 (40), 98 (55). HRMS (ESI) calculated for $C_{21}H_{24}O_3N_2Na$: 375.1679 [M+Na]$^+$, found: 375.1691.

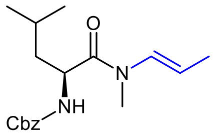

(*S,E*)-benzyl (4-methyl-1-(methyl(prop-1-en-1-yl)amino)-1-oxopentan-2-yl) carbamate (**33**): Prepared, following the general procedure from (*S*)-benzyl (1-(allyl(methyl)amino)-4-methyl-1-oxopentan-2-yl)carbamate (**33a**). The title product was obtained after purification by column chromatography (Hexane/EtOAc 60:40) as a liquid (55.9 mg, 0.18 mmol, 84%, *E:Z* >95:5, based on ^{1}H-NMR). R_f = 0.58 (Hexane/EtOAc 70:30). $[\alpha]_D^{25}$ +68.6 (c 1.0, $CHCl_3$); 99.8% ee as determined by HPLC (IC, 4:1 *n*-hexane/EtOH, 1.0 mL/min). ^{1}H NMR (400 MHz, $CDCl_3$): δ 7.33 – 7.19 (m, 5H), 6.63 (d, *J* = 13.6 Hz, 1H), 5.57 – 5.55 (m, 1H), 5.15 (dq, *J* = 13.9, 6.9 Hz, 1H), 5.07 (s, 2H), 4.86 – 4.76 (m, 1H), 3.12 – 3.06 (m, 3H), 1.73 – 1.71 (m, 4H), 1.52 – 1.38 (m,

2H), 1.01 – 0.88 (m, 6H). ^{13}C NMR (101 MHz, $CDCl_3$): (mixture of rotamers) δ 171.4, 170.9, 156.3, 136.5, 128.6, 128.2, 128.1, 127.8, 110.1, 107.8, 67.0, 50.1, 50.0, 42.8, 32.3, 31.1, 24.8, 23.5, 21.8, 21.7, 15.6, 15.5. IR (neat, cm^{-1}): 3304, 2955, 1714, 1527, 1244, 1113, 1043, 738, 697. HRMS (ESI) calculated for $C_{18}H_{26}O_3N_2Na$: 341.1836 [M+Na]$^+$, found: 341.1848.

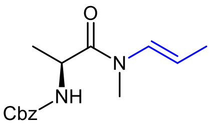

(*S,E*)-benzyl (1-(methyl(prop-1-en-1-yl)amino)-1-oxopropan-2-yl) carbamate (**34**): Prepared, following the general procedure from (*S*)-benzyl (1-(allyl(methyl)amino)-1-oxopropan-2-yl)carbamate (**34a**). The title product was obtained after purification by column chromatography (Hexane/EtOAc 70:30) as a liquid (52.1 mg, 0.19 mmol, 90%, *E:Z* 94:6, based on ^{1}H-NMR). R_f = 0.67 (Hexane/EtOAc 50:50). $[\alpha]_D^{25}$ +68.2 (c 1.0, $CHCl_3$); >99.9% ee as determined by HPLC (IC, 4:1 *n*-hexane/EtOH, 1.0 mL/min). ^{1}H NMR (400 MHz, $CDCl_3$): δ 7.35 – 7.22 (m, 5H), 6.62 (d, *J* = 13.6 Hz, 1H), 5.85 – 5.84 (m, 1H), 5.17 (dq, *J* = 13.3, 6.9 Hz, 1H), 5.10 (s, 2H), 4.84 – 4.72 (m, 1H), 3.11 – 3.09 (m, 3H), 1.74 (d, *J* = 6.6 Hz, 3H), 1.35 (d, *J* = 6.8 Hz, 3H). ^{13}C NMR (101 MHz, $CDCl_3$): (mixture of rotamers) δ 171.1, 170.6, 155.6, 136.5, 128.6, 128.2, 128.1, 127.9, 127.7, 109.9, 107.9, 66.9, 47.7, 47.5, 32.3, 31.0, 19.3, 19.2, 15.5 (15.54), 15.5 (15.47). IR (neat, cm^{-1}): 3404, 2970, 1714, 1642, 1244, 1057, 738. HRMS (ESI) calculated for $C_{15}H_{20}O_3N_2Na$: 299.1366 [M+Na]$^+$, found: 299.1376.

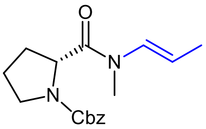

(*R,E*)-benzyl 2-(methyl(prop-1-en-1-yl)carbamoyl)pyrrolidine-1-carboxylate (**35**): Prepared, following the general procedure from (*R*)-benzyl 2-(allyl(methyl)carbamoyl)pyrrolidine-1-carboxylate (**35a**). The title product was obtained after purification by column chromatography (Hexane/EtOAc 50:50) preparative HPLC (EtOAc) as a liquid (51.2 mg, 0.17 mmol, 84%, *E:Z* 92:8, based on GC-MS). R_f = 0.7 (Hexane/EtOAc 20:80). $[\alpha]_D^{25}$ -7.1 (c 1.0, $CHCl_3$); >99.9% ee as determined by HPLC (IC, 4:1 *n*-hexane/EtOH, 1.0 mL/min). ^{1}H NMR (400 MHz, $CDCl_3$): (mixture of rotamers) δ 7.37 – 7.23 (m, 5H), 6.68 (major rotamer, dd, *J* = 13.7, 1.8 Hz, 0.6H), 6.51 (minor rotamer, dd, *J* = 13.7, 1.9 Hz, 0.4H), 5.21 – 4.99 (m, 3H), 4.84 – 4.66(m, 1H), 3.72 – 3.49 (m, 2H), 3.14– 2.96 (m, 3H), 2.24 – 1.99 (m, 2H), 1.95 – 1.86 (m, 2H), 1.72 (major rotamer, dd, *J* = 6.7, 1.6 Hz, 2H), 1.67 (minor rotamer, dd, *J* = 6.7, 1.6 Hz, 1H). ^{13}C NMR (101 MHz, $CDCl_3$): (both amides are forming rotamers) δ 170.8, 170.5, 170.0, 155.0, 155.3, 136.9, 136.8, 128.5, 128.4 (128.40), 128.4 (128.36), 128.3, 128.2, 128.1, 127.9 (127.94), 127.9 (127.90), 127.8, 127.7, 109.6, 109.2, 106.6, 106.5, 67.1, 67.0, 66.9, 57.8, 57.7, 57.2, 57.1, 47.3, 46.7, 32.2, 32.0, 31.1, 30.8, 30.6, 29.8, 29.7, 24.2, 23.5, 15.6 (15.59), 15.6 (15.55), 15.5 (15.49), 15.5 (15.48). IR (neat, cm^{-1}): 2955, 2883, 1704, 1652, 1414, 1352, 1120, 738, 698. MS (70 eV, EI): *m/z* (%): 302 (1) [M$^+$], 204 (27), 160 (25), 91 (100). HRMS (ESI) for $C_{17}H_{22}O_3N_2Na$: 325.1523 [M+Na]$^+$, found: 325.1532.

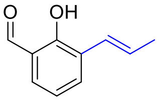

***(E)*-2-hydroxy-3-(prop-1-en-1-yl)benzaldehyde** (**36**): Prepared, following the general procedure from 3-allyl-2-hydroxybenzaldehyde. The title product was obtained after purification by column chromatography (Pentane/Et_2O 95:5) as a colourless liquid (30.1 mg, 0.18 mmol, 93%, *E:Z* 98:2, based on GC-MS). R_f = 0.74 (Hexane/EtOAc 80:20). ^{1}H NMR (400 MHz, $CDCl_3$): δ 11.43 (s, 1H), 9.87 (s, 1H), 7.63 (dd, *J* = 7.6, 1.2 Hz, 1H), 7.41 (dd, *J* = 7.7, 1.5 Hz, 1H), 6.97 (dd, *J* = 7.6, 7.6 Hz, 1H), 6.70 (dd, *J* = 15.9, 1.6 Hz, 1H), 6.35 (dq, *J* = 15.9, 6.7 Hz, 1H), 1.93 (dd, *J* = 6.6, 1.8 Hz, 3H). ^{13}C NMR (101 MHz, $CDCl_3$): δ 197.0, 158.6, 133.7, 132.3, 128.5, 127.2, 124.0, 120.7, 119.8, 19.1. MS (70 eV, EI): *m/z* (%): 162 (100) [M$^+$], 147 (18), 133 (29), 115 (57), 105 (29), 91 (37), 77 (31). These data are in agreement with those reported previously in the literature.[19]

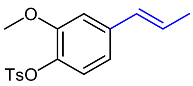

***(E)*-2-methoxy-4-(prop-1-en-1-yl)phenyl 4-methylbenzenesulfonate** (**37**): Prepared, following the general procedure from 4-allyl-2-methoxyphenyl 4-methylbenzenesulfonate (**37a**). The title product was obtained after purification by column chromatography (Hexane/EtOAc 60:40) as a colorless liquid (59 mg, 0.19 mmol, 94%, *E:Z* 96:4, based on GC-MS). R_f = 0.6 (Hexane/EtOAc 70:30). ^{1}H NMR (400 MHz, $CDCl_3$): δ 7.73 (d, *J* = 8.3 Hz, 2H), 7.28 (d, *J* = 8.1 Hz, 2H), 7.04 (d, *J* = 8.3 Hz, 1H), 6.82 (dd, *J* = 8.3, 2.0 Hz, 1H), 6.76 (d, *J* = 1.9 Hz, 1H), 6.31 (dd, *J* = 15.7, 1.7 Hz, 1H), 6.18 (dq, *J* = 15.7, 6.5 Hz, 1H), 3.53 (s, 3H), 2.43 (s, 3H), 1.86 (dd, *J* = 6.5, 1.5 Hz, 3H). ^{13}C NMR (101 MHz, $CDCl_3$): δ 151.7, 145.0, 138.1, 137.2, 133.3, 130.2, 129.4, 128.7, 127.1, 124.0, 118.2, 109.8, 55.5, 21.8, 18.5. MS (70eV, EI): *m/z* (%): 318 (13) [M$^+$], 163 (100), 107 (30), 91 (34), 77 (8).These data are in agreement with those reported previously in the literature.[8]

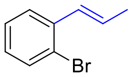

***(E)*-1-bromo-2-(prop-1-en-1-yl)benzene** (**38**): Prepared, following the general procedure from 1-allyl-2-bromobenzene. The title product was obtained after purification by column chromatography (pentane) as a colorless liquid (35.2 mg, 0.18mmol, 88%, *E:Z* 95:5, based on GC-MS). R_f = 0.6 (Hexane). ^{1}H NMR (400 MHz, $CDCl_3$): δ 7.53 (d, *J* = 8.0 Hz, 1H), 7.48 (dd, *J* = 7.8, 1.3 Hz, 1H), 7.26 – 7.22 (m, 1H), 7.08 – 7.04 (m, 1H), 6.74 (dq, *J* = 15.6, 1.9 Hz, 1H), 6.19 (dq, *J* = 15.7, 6.7 Hz, 1H), 1.93 (dd, *J* = 6.7, 1.8 Hz, 3H). ^{13}C NMR (101 MHz, $CDCl_3$): δ 137.8, 132.9, 130.0, 129.0, 128.2, 127.5, 126.9, 123.1, 18.8. MS (70 eV, EI): *m/z* (%): 198 (47) [M$^+$], 196 (48), 117 (80), 115 (100), 91 (22). These data are in agreement with those reported previously in the literature.[20]

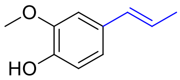

***(E)*-2-methoxy-4-(prop-1-en-1-yl)phenol** (**39**): Prepared, following the general procedure from 4-allyl-2-methoxyphenol. The title product was obtained after purification by column chromatography (Hexane/EtOAc 90:10) as a colourless liquid (27.9 mg, 0.17 mmol, 85%, *E:Z* 99:1, based on GC-MS). R_f = 0.41 (Hexane/EtOAc 80:20). ^{1}H NMR (400 MHz, $CDCl_3$): δ 6.90 – 6.81 (m, 3H), 6.33 (dq, *J* = 15.7, 1.7 Hz, 1H), 6.08 (dq, *J* = 15.7, 6.6 Hz, 1H), 5.57 (s, 1H), 3.90 (s, 3H), 1.86 (dd, *J* = 6.6, 1.7 Hz, 3H). ^{13}C NMR (101 MHz, $CDCl_3$): δ 146.7, 144.9, 130.8 (130.84), 130.8 (130.76), 123.6, 119.4, 114.5, 108.0, 56.0, 18.5. MS (70 eV, EI): *m/z* (%): 164 (100) [M$^+$], 149 (34), 131 (21), 103 (26), 91 (22), 77 (27). These data are in agreement with those reported previously in the literature.[8]

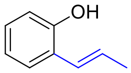

***(E)*-2-(prop-1-en-1-yl)phenol** (**40**): Prepared, following the general procedure from 2-allylphenol. The title product was obtained after purification by filtration through a small pad of silica and washing with methanol (20 mL) as a colourless liquid (37.2 mg, 0.19 mmol, 92%, *E:Z* >99:1, based on GC-MS). R_f = 0.29 (Hexane/EtOAc 90:10). ^{1}H NMR (400 MHz, CD_3OD): δ 7.30 (dd, *J* = 8.1, 1.7 Hz, 1H), 7.01 – 7.96 (m, 1H), 6.74 (dd, *J* = 7.5, 7.5 Hz, 2H), 6.66 (dq, *J* = 16.0, 1.8 Hz, 1H), 6.21 (dq, *J* = 15.9, 6.6 Hz, 1H), 4.88 (s, 1H), 1.86 (dd, *J* = 6.6, 1.6 Hz, 3H). ^{13}C NMR (101 MHz, CD_3OD): δ 155.2, 128.6, 127.4, 127.3, 126.3, 125.9, 120.6, 116.4, 19.0. MS (70 eV, EI): *m/z* (%): 135 (100), 134 (53) [M$^+$], 119 (17), 115 (11), 92 (30), 78 (24). These data are in agreement with those reported previously in the literature.[14]

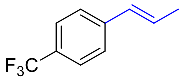

***(E)*-1-(prop-1-en-1-yl)-4-(trifluoromethyl)benzene** (**41**): Prepared, following the general procedure from 1-allyl-4-(trifluoromethyl)benzene. The title product was obtained after purification by column chromatography (pentane) as a colourless liquid (34 mg, 0.18 mmol, 85%, *E:Z* 98:2, based on GC-MS). R_f = 0.72 (Hexane). ^{1}H NMR (400 MHz, $CDCl_3$): δ 7.53 (d, *J* = 8.2 Hz, 2H), 7.41 (d, *J* = 8.1 Hz, 2H), 6.43 (d, *J* = 16.2 Hz, 1H), 6.35 (dq, *J* = 15.8, 6.1 Hz, 1H), 1.92 (dd, *J* = 6.3, 1.2 Hz, 3H). ^{13}C NMR (101 MHz, $CDCl_3$): δ 141.5, 134.2, 130.0, 128.8, 126.1, 125.6 (q, *J* = 3.7 Hz), 123.1, 18.7. ^{19}F

NMR (376 MHz, $CDCl_3$): δ −62.4 (s). MS (70 eV, EI): *m/z* (%): 186 (62) [M^+], 167 (12), 117 (100), 115 (48), 91 (10). These data are in agreement with those reported previously in the literature.[8]

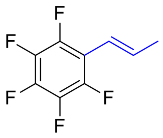

***(E)*-1,2,3,4,5-pentafluoro-6-(prop-1-en-1-yl)benzene** (**42**): Prepared, following the general procedure from 1-allyl-2,3,4,5,6-pentafluorobenzene. The title product was obtained after purification by filtration through a small pad of silica and washing with pentane (20 mL) as a colourless liquid (38.6 mg, 0.18 mmol, 82%, *E:Z* 93:7, based on GC-MS). R_f = 0.76 (Hexane). ^{1}H NMR (400 MHz, CD_3OD): δ 6.60 (dq, *J* = 16.3, 6.7 Hz, 1H), 6.33 (dq, *J* = 16.2, 1.8 Hz, 1H), 1.96 (dd, *J* = 6.6, 1.6 Hz, 3H). ^{13}C NMR (101 MHz, CD_3OD): 137.6, 116.1, 19.8. ^{19}F NMR (376 MHz, CD_3OD): δ −146.44 (dd, 2F, *J* = 20.7, 7.6 Hz), −161.35 (t, 1F, *J* = 20.0 Hz), −166.58 (td, 2F, *J* = 20.6, 7.5 Hz). MS (70 eV, EI): *m/z* (%): 208 (100) [M^+], 181 (85), 169 (20), 143 (8). *Note:* Only non-fluorinated carbons were observable in ^{13}C. These data are in agreement with those reported previously in the literature.[14]

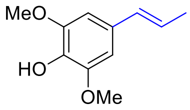

***(E)*-2,6-dimethoxy-4-(prop-1-en-1-yl)phenol** (**43**): Prepared, following the general procedure from 4-allyl-2,6-dimethoxyphenol. The title product was obtained after purification by filtration through a small pad of silica and washing with methanol (20 mL) as a colourless liquid (37.2 mg, 0.19 mmol, 98%, *E:Z* >99:1, based on GC-MS). R_f = 0.29 (Hexane/EtOAc 80:20). ^{1}H NMR (400 MHz, CD_3OD): δ 6.61 (s, 2H), 6.29 (d, *J* = 15.7 Hz, 1H), 6.10 (dq, *J* = 15.7, 6.5 Hz, 1H), 4.87 (s, 1H), 3.83 (s, 6H), 1.83 (dd, *J* = 6.6, 1.6 Hz, 3H). ^{13}C NMR (101 MHz, CD_3OD): δ 149.2, 135.8, 132.4, 130.7, 123.9, 104.2, 56.7, 18.5. MS (70 eV, EI): *m/z* (%): 194 (100) [M^+], 179 (17), 151 (11), 131 (14), 119 (17), 91 (26). These data are in agreement with those reported previously in the literature.[21]

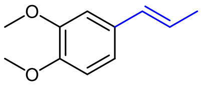

***(E)*-1,2-dimethoxy-4-(prop-1-en-1-yl)benzene** (**44**): Prepared, following the general procedure from 4-allyl-1,2-dimethoxybenzene. The title product was obtained after purification by column chromatography (Hexane/EtOAc 90:10) as a colorless liquid (33.9 mg, 0.19 mmol, 94%, *E:Z* 99:1, based on GC-MS). R_f = 0.57 (Hexane/EtOAc 80:20). ^{1}H NMR (400 MHz, $CDCl_3$): δ 6.90 – 6.84 (m, 2H), 6.79 (d, *J* = 8.2 Hz, 1H), 6.34 (dq, *J* = 15.6, 1.7 Hz, 1H), 6.11 (dq, *J* = 15.7, 6.6 Hz, 1H), 3.89 (s, 3H), 3.87 (s, 3H), 1.87 (dd, *J* = 6.6, 1.7 Hz, 3H). ^{13}C NMR (101 MHz, $CDCl_3$): 149.1, 148.2, 131.2, 130.7, 123.9, 118.7, 111.2, 108.5, 56.0, 55.9, 18.5. MS (70 eV, EI): *m/z* (%): 178 (100) [M^+], 163 (30), 147 (22), 107 (16), 91 (42), 77 (8). These data are in agreement with those reported previously in the literature.[8]

6. General procedure for site selective couplings and characterization data of products

6.1. General procedure for site selective coupling reactions

General Procedure A: C-OTf/OFs selective cross-coupling reactions[10-11]

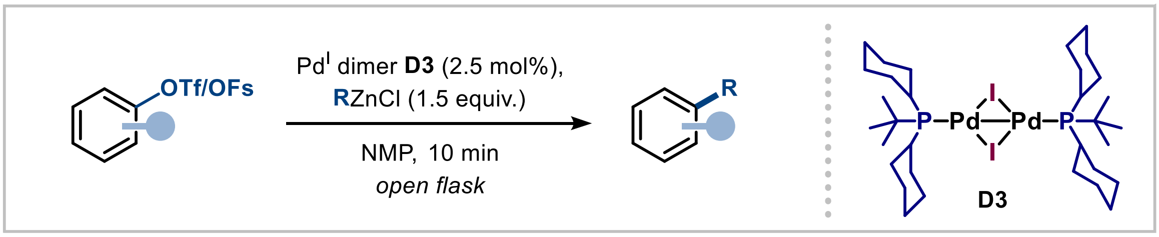

Under an argon atmosphere, a solution of Grignard reagent (0.3 mmol, 1.5 equiv.) and $ZnCl_2$ (1M in THF, 0.4 mL, 2.0 equiv.)[22] were added to a oven-dried 16 mL vial and stirred for 15 minutes. The vial was then opened to air and a solution of the aryl triflate or aryl fluorosulfate (0.2 mmol, 1 equiv.) in NMP (0.5 mL) was added, followed by NMP (2 x 0.25 mL) used to wash the vial containing the aryl triflate. Then immediately [Pd(μ-I)(PCy_2tBu)]$_2$ **D3** (4.9 mg, 0.005 mmol, 0.025 equiv.) was added in one portion. The reaction mixture was stirred for 10 minutes, and the crude reaction mixture was filtered through a small pad of silica and washed with pentane (20 mL). Volatiles were removed under reduced pressure and the obtained crude was subjected to column chromatography on silica for further purification.

General Procedure B: Sequential coupling[11]

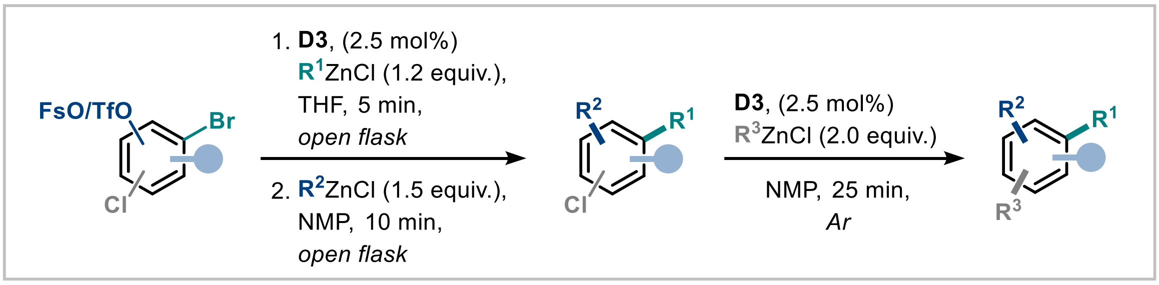

The vial containing organozinc solution (0.36 mmol, 1.2 equiv., in THF; prepared analogous to general procedure A) was opened to air and a solution of aryl fluorosulfate or triflate (0.2 mmol, 1.0 equiv.) in THF (1 mL) was added, followed by immediate addition of [Pd(μ-I)(PCy_2tBu)]$_2$ **D3** (4.9 mg, 0.005 mmol, 0.025 equiv.) in one portion. The reaction mixture was stirred for 5 minutes at ambient temperature in an open flask.

NMP (1.5 mL) was added to the second organozinc solution (0.3 mmol, 1.5 equiv., in THF; prepared analogous to general procedure A) and the mixture was then added to the reaction vial in air. The mixture was further stirred for 10 minutes and then the intermediate was filtered through a small silica pad using pentane and the volatles were removed afterwards. The compound was transferred to a vial and dissolved in NMP (1.5 mL) under an argon atmosphere and placed in an aluminium heating block at 80°C. The solution of the third organozinc reagent (0.8 mmol, 2 equiv., in THF; prepared analogous to general procedure A) and [Pd(μ-I)(PCy_2tBu)]$_2$ **D3** (4.9 mg, 0.005 mmol, 0.025 equiv.) dissolved in THF (0.5 mL) were then added simultaneously *via* separate syringe pumps to the reaction mixture over 15 minutes. Once addition was complete the reaction mixture was stirred for a further 10 minutes at 80°C. The obtained reaction mixture was subjected to column chromatography on silica for further purification.

6.2. Characterization data of cross-coupling products

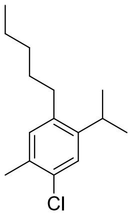

1-chloro-5-isopropyl-2-methyl-4-pentylbenzene (**45**): Prepared, following the general procedure A from 4-chloro-2-isopropyl-5-methylphenyl trifluoromethanesulfonate (**45a**). The title product was obtained after purification by column chromatography (hexane) as a colourless liquid (35 mg, 0.15 mmol, 75%). R_f = 0.75 (hexane). ^{1}H NMR (400 MHz, $CDCl_3$): δ 7.19 (s, 1H), 6.97 (s, 1H), 3.09 (hept, *J* = 6.8 Hz, 1H), 2.57 – 2.53 (m, 2H), 2.30 (s, 3H), 1.56 – 1.49 (m, 2H), 1.37 – 1.33 (m, 4H), 1.21 (d, *J* = 6.9 Hz, 6H), 0.92 – 0.89 (m, 3H). ^{13}C NMR (101 MHz, $CDCl_3$): δ 146.0, 138.4, 132.8, 132.0 (132.03), 132.0 (131.97), 126.0, 32.4, 32.1, 31.7, 28.6, 24.1, 22.7, 19.6, 14.2. IR (neat, cm^{-1}): 2930, 2866, 1465, 979, 882, 729. MS (70 eV, EI): *m/z* (%): 240 (14) [^{37}Cl-M$^+$], 238 (41) [^{35}Cl-M$^+$], 183 (20), 181 (63), 169 (32), 167 (100), 155 (16), 153 (49). HRMS (ESI) calculated for $C_{15}H_{24}Cl$: 239.1561 [M+H]$^+$, found: 239.1559.

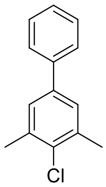

4-chloro-3,5-dimethyl-1,1'-biphenyl (**46**): Prepared, following the general procedure A from 4-chloro-3,5-dimethylphenyl trifluoromethanesulfonate (**46a**). The title product was obtained after purification by column chromatography (pentane) as a white solid (41 mg, 0.19 mmol, 95%). R_f = 0.65 (Hexane). ^{1}H NMR (600 MHz, $CDCl_3$): δ 7.58 – 7.55 (m, 2H), 7.44 (dd, *J* = 7.7, 7.7 Hz, 2H), 7.35 (dd, *J* = 7.1, 7.1 Hz, 1H), 7.31 (s, 2H), 2.45 (s, 6H). ^{13}C NMR (151 MHz, $CDCl_3$): δ 140.5, 139.2, 136.6, 134.0, 128.9, 127.5, 127.3, 127.1, 21.0. MS (70 eV, EI): *m/z* (%): 218 (33) [^{37}Cl-M$^+$], 216 (100) [^{35}Cl-M$^+$], 181 (38), 178 (18), 165 (64), 89 (8). These data are in agreement with those previously reported in the literature.[11]

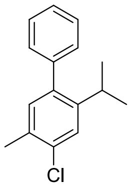

4-chloro-2-isopropyl-5-methyl-1,1'-biphenyl (**47**): Prepared, following the general procedure A from 4-chloro-2-isopropyl-5-methylphenyl trifluoromethanesulfonate (**45a**). The title product was obtained after purification by column chromatography (hexane) as a colourless liquid (39.1 mg, 0.16 mmol, 80%). R_f = 0.71 (hexane). ^{1}H NMR (400 MHz, $CDCl_3$): δ 7.46– 7.37 (m, 4H), 7.31– 7.29 (m, 2H), 7.09 (s, 1H), 3.03 (hept, *J* = 6.9 Hz, 1H), 2.40 (s, 3H), 1.18 (d, *J* = 6.9 Hz, 6H). ^{13}C NMR (101 MHz, $CDCl_3$): δ 145.8, 141.2, 139.7, 133.8, 132.9, 132.5, 129.3, 128.2, 127.1, 126.3, 29.4, 24.3, 19.6. IR (neat, cm^{-1}): 2962, 2872, 2328, 1477, 1061, 886, 768, 702. MS (70 eV, EI): *m/z* (%): 246 (32) [^{37}Cl-M$^+$], 244 (96) [^{35}Cl-M$^+$], 229 (100), 194 (61), 179(65). HRMS (ESI) calculated for $C_{16}H_{18}Cl$: 245.1095 [M+H]$^+$, found: 245.1095.

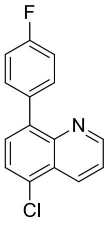

5-chloro-8-(4-fluorophenyl)quinoline (**48**): Prepared, following the general procedure A from 5-chloroquinolin-8-yl trifluoromethanesulfonate (**48a**). The title product was obtained after purification by column chromatography (Hexane/EtOAc 95:5) as a white solid (46.1 mg, 0.18 mmol, 90%). R_f = 0.46 (Hexane/EtOAc 90:10). M.p. = 125 – 127°C. ^{1}H NMR (600 MHz, $CDCl_3$): δ 9.00 – 8.99 (m, 1H), 8.66 (dd, *J* = 8.5, 1.4 Hz, 1H), 7.69 (d, *J* = 7.8 Hz, 1H), 7.69 – 7.60 (m, 3H), 7.55 (dd, *J* = 8.5, 4.1 Hz, 1H), 7.23 – 7.18 (m, 2H). ^{13}C NMR (151 MHz, $CDCl_3$): δ 163.5, 161.8, 150.8, 146.6, 139.3, 134.9 (d, *J* = 3.1 Hz), 132.3 (d, *J* = 8.0 Hz), 130.8, 130.0, 126.8, 126.5, 122.0, 115.2 (d, *J* = 21.3 Hz). ^{19}F NMR (564 MHz, $CDCl_3$): δ −115.1 (s). MS (70 eV, EI): *m/z* (%): 259 (13), [^{37}Cl-M$^+$], 258 (17), 257 (39) [^{35}Cl-M$^+$], 256 (100), 222 (29), 221 (16), 128 (7), 111 (15). These data are in agreement with those reported previously in the literature.[11]

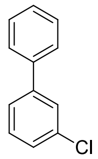

3-chloro-1,1'-biphenyl (**49**): Prepared, following the general procedure A from 3-chlorophenyl fluorosulfate (**49a**). The title product was obtained after purification by column chromatography (pentane) as a colourless liquid (34.7 mg, 0.18 mmol, 92%). R_f = 0.6 (hexane). ^{1}H NMR (600 MHz, $CDCl_3$): δ 7.60 – 7.58 (m, 3H), 7.49 – 7.46 (m, 3H), 7.41 – 7.37 (m, 2H), 7.35 – 7.33 (m, 1H). ^{13}C NMR (151 MHz, $CDCl_3$): δ 143.2, 139.9, 134.8, 130.1, 129.0, 128.0, 127.4 (127.42), 127.4 (127.38), 127.2, 125.4. MS (70eV, EI): *m/z* (%): 190 (33) [^{37}Cl-M$^+$], 189 (13), 188 (100) [^{35}Cl-M$^+$], 153 (25), 152 (55), 151 (8). These data are in agreement with those reported previously in the literature.[11]

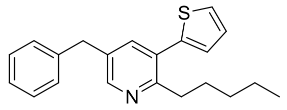

5-Benzyl-2-pentyl-3-(thiophen-2-yl)pyridine (**50**): Prepared, following the general procedure B from 3-bromo-5-chloropyridin-2-yl trifluoromethane-sulfonate (**50a**). The title product was obtained after purification by column chromatography (pentane) as a yellow liquid (38.5 mg, 0.12 mmol, 60%). R_f = 0.67 (Hexane/EtOAc 90:10). ^{1}H NMR (600 MHz, $CDCl_3$): δ 8.43 (d, *J* = 2.1 Hz, 1H), 7.45 – 7.43 (m, 1H), 7.37 (dd, *J* = 5.1, 0.9 Hz, 1H), 7.34 – 7.31 (m, 2H), 7.27 – 7.20 (m, 3H), 7.10 (dd, *J* = 5.1, 3.6 Hz, 1H), 7.04 (dd, *J* = 3.5, 1.0 Hz, 1H), 3.98 (s, 2H), 2.89 – 2.86 (m, 2H), 1.71 – 1.66 (m, 2H), 1.33 – 1.29 (m, 4H), 0.86 (t, *J* = 7.1 Hz, 3H). ^{13}C NMR (151 MHz, $CDCl_3$): δ 158.4, 149.0, 140.9, 139.8, 138.7, 133.4, 129.3, 128.9, 128.7, 127.2, 127.0, 126.5, 125.9, 38.6, 35.6, 31.9, 29.8, 22.7, 14.2. MS (70 eV, EI): *m/z* 321 (5) [M$^+$], 278 (21), 265 (100), 91 (18). These data are in agreement with those reported previously in the literature.[11]

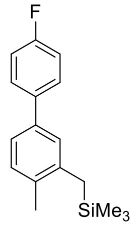

((4'-Fluoro-4-methyl-[1,1'-biphenyl]-3-yl)methyl)trimethylsilane (**51**): Prepared, following the general procedure B from 4-bromo-2-chlorophenyl fluorosulfate (**51a**). The title product was obtained after purification by column chromatography (pentane) as a colourless liquid (38.1 mg, 0.14 mmol, 70%). R_f = 0.70 (Pentane/Et_2O 90:10). ^{1}H NMR (600 MHz, $CDCl_3$): δ 7.55 – 7.51 (m, 2H), 7.19-7.18 (m, 2H), 7.16 (s, 1H), 7.13 – 7.11 (m, 2H), 2.29 (s, 3H), 2.19 (s, 2H), 0.07 (s, 9H). ^{13}C NMR (151 MHz, $CDCl_3$): δ 162.3 (d, *J* = 245.4 Hz), 139.6, 137.7 (137.71), 137.7 (137.70), 133.9, 130.7, 128.5 (d, *J* = 8.0 Hz), 127.4, 122.9, 115.6 (d, *J* = 21.2 Hz), 24.1, 20.2, −1.2. ^{19}F NMR (564 MHz, $CDCl_3$): δ −116.9 (s). MS (70 eV, EI): m/z (%): 272 (21) [M+], 199 (14), 184 (13), 183 (26), 180 (55), 165 (10), 73 (100). These data are in agreement with those reported previously in the literature.[11]

7. Mechanistic Studies

7.1. Reaction Progress

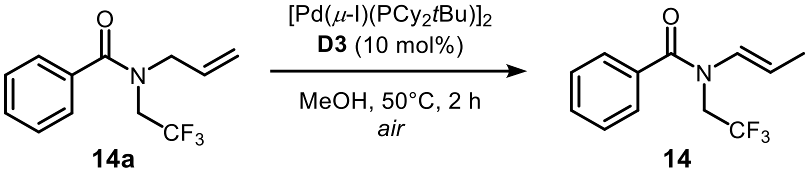

In air, $[Pd(\mu\text{-}I)(PCy_2tBu)]_2$ **D3** (58.6 mg, 0.06 mmol, 0.1 equiv.) was weighed into a 15 mL vial and *N*-allyl-*N*-(2,2,2-trifluoroethyl)benzamide (**14a**, 145.9 mg, 0.6 mmol, 1.0 equiv.) dissolved in MeOH (3.0 mL) was added. α,α,α-Trifluorotoluene (44.1 mg, 0.3 mmol, 0.5 equiv.) was added as an internal standard and the resulting mixture was stirred at 50°C. Aliquots (0.5 mL) were taken after 15 min, 30 min, 45 min, 60 min and 90 min and analysed *via* ^{19}F NMR and ^{31}P NMR.

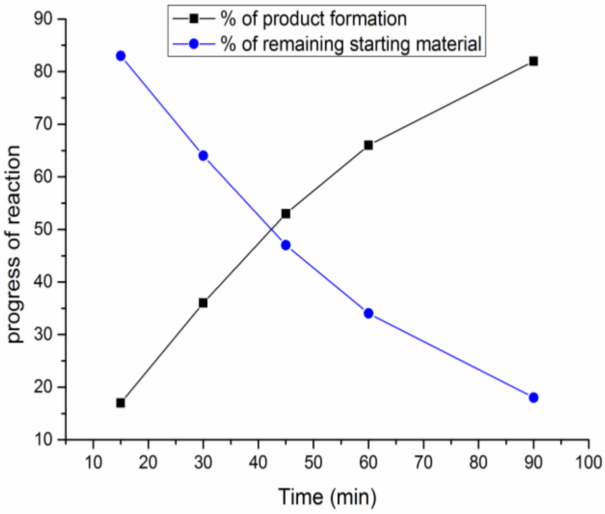

Figure S1 | Study of reaction progress via ^{19}F NMR.

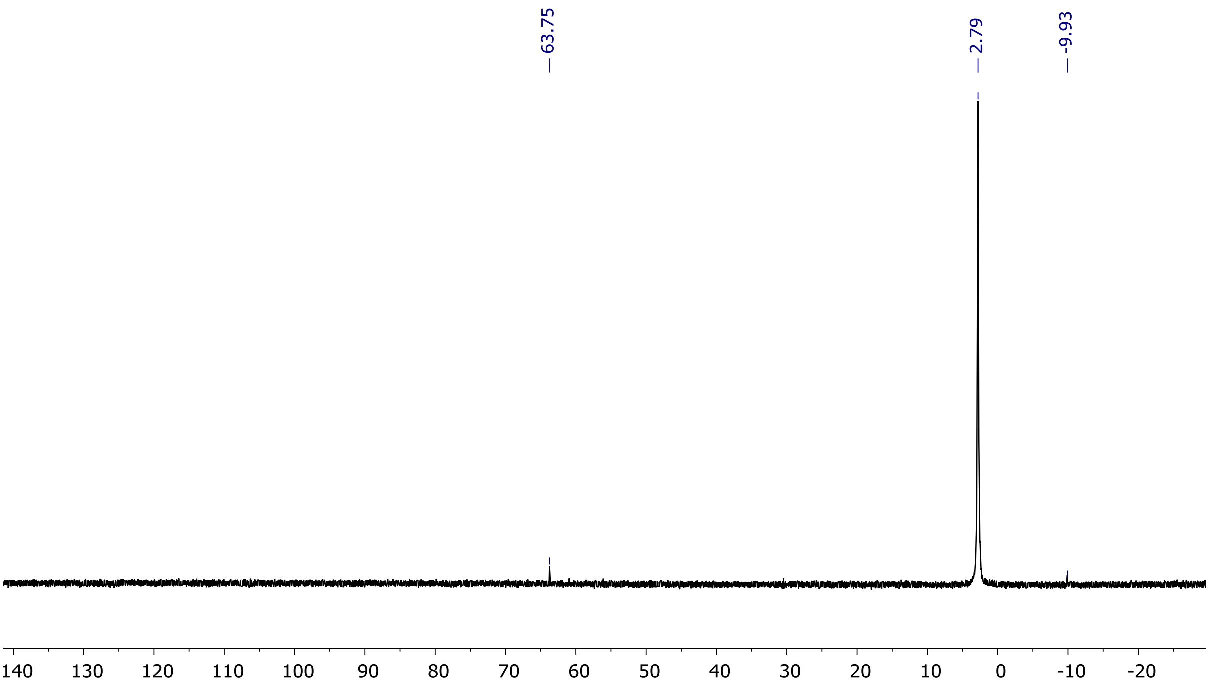

Figure S2 | ^{31}P NMR of the reaction mixture after 1h.

7.2. Control experiment with Pd[II]H in air

[(PCy$_2$*t*Bu)$_2$Pd(H)(Cl)] (C3)[23]

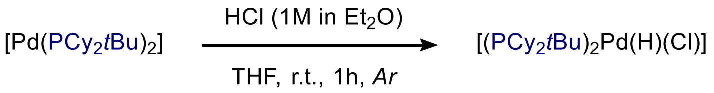

Inside an Argon-filled glovebox, HCl (0.11 mL, 1M in diethyl ether, 0.11 mmol, 1.1 equiv.) was added dropwise to a solution of [Pd(PCy$_2$*t*Bu)$_2$] in THF (0.1M). After 1 hour of stirring at ambient temperature the formation of [(PCy$_2$*t*Bu)$_2$Pd(H)(Cl)] was confirmed by ^{1}H NMR (see Figure S3 and Figure S4). All volatiles were removed *in vacuo* and the obtained white solid was used without further purification. ^{1}H NMR (400 MHz, THF-*d*$_8$): δ 2.35 – 2.19 (m, 8H), 2.04 (d, *J* = 13.0 Hz, 4H), 1.83 – 1.57 (m, 20H, overlap with solvent), 1.44 – 1.17 (m, 30H), -14.90 (t, *J* = 5.4 Hz, 1H). ^{31}P NMR (121 MHz, THF-*d*$_8$): δ 55.7 (s). The data are in agreement with those previously reported in the literature.[24]

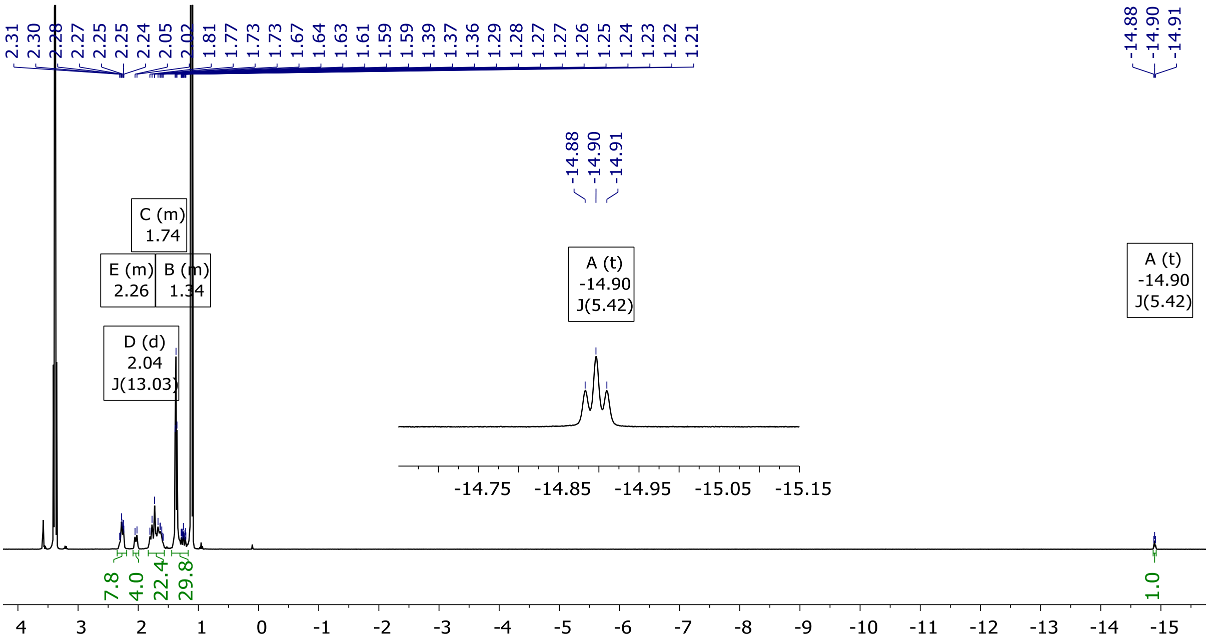

Figure S3 | ^{1}H NMR of [Pd(H)(Cl)(PCy$_2$*t*Bu)$_2$].

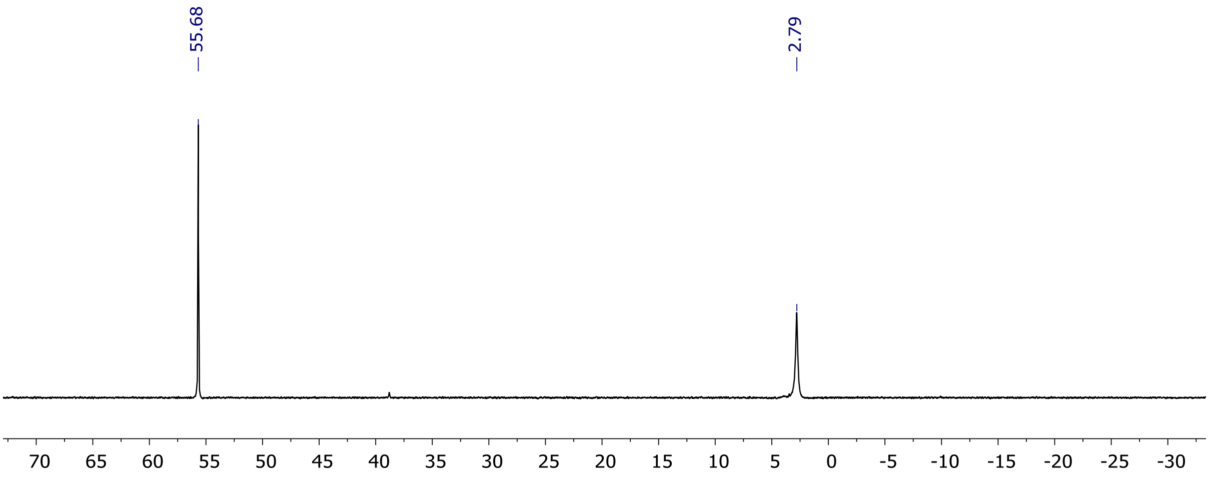

Figure S4 | ^{31}P NMR of [Pd(H)(Cl)(PCy$_2$*t*Bu)$_2$].

Olefin isomerization using Pd(II)H in air

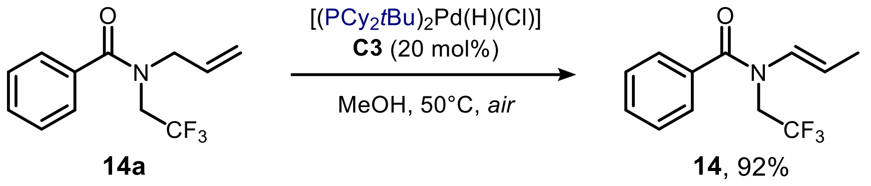

The obtained [(PCy_2tBu)$_2$Pd(H)(Cl)] **C3** was subjected to air and was weighed into a 5 mL vial (35.2 mg, 0.04 mmol, 0.2 equiv.). A solution of *N*-allyl-*N*-(2,2,2-trifluoroethyl)benzamide (48.6 mg, 0.2 mmol, 1.0 equiv) in MeOH (1.0 mL) was added and the resulting mixture stirred at 50°C in air overnight. ^{19}F NMR analysis of the crude reaction mixture showed the formation of (*E*)-*N*-(prop-1-en-1-yl)-*N*-(2,2,2-trifluoroethyl)benzamide in 92% yield.

7.3. Cross-over experiment

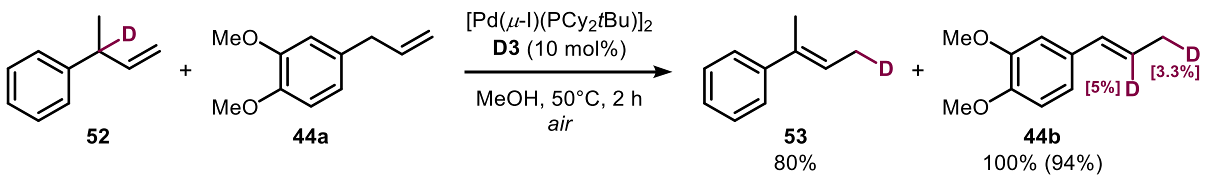

The cross-over experiment was performed under standard isomerization reaction conditions using 5 mol% Pd(I) dimer **D3** (4.9 mg, 0.005 mmol), 4-allyl-1,2-dimethoxybenzene (**44a**) (17.8 mg, 0.1 mmol) and but-3-en-2-yl-2-d_1-benzene (**52**)[8] (13.3 mg 0.1 mmol). After 2 hours an aliquot of the reaction mixture was analysed by GC-MS (see Figure S5) and the crude was purified *via* column chromatography: Compound **53** (R_f = 0.6 (hexane), 80% conversion to product as determined by GC-MS of the crude), was eluted first using pentane as eluent before using pentane/ether (90:10) to elute compound **44b** (full conversion to product as determined by GC-MS of the crude; 94% isolated yield; *E:Z* 99:1, based on GC-MS; R_f = 0.57 (Hexane/EtOAc 80:20)). The isolated product **44b** was analyzed *via* ^{1}H and ^{2}H NMR to determine the extent of deuteration.

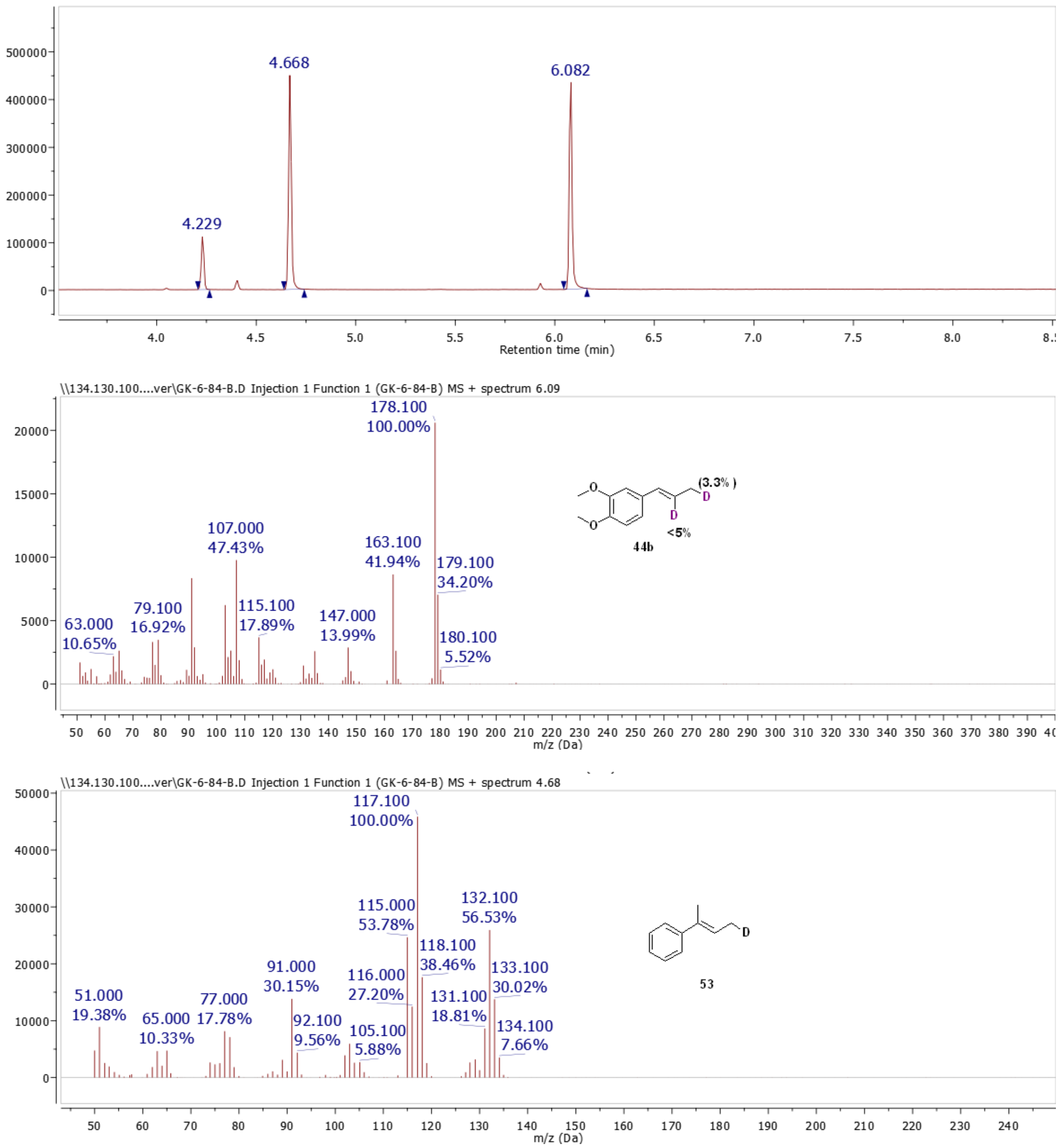

Figure S5 | GC-MS of the crude reaction mixture containing **44b** (t_r = 6.08 min), **53** (t_r = 4.67 min) and unreacted **52** (t_r = 4.23 min).

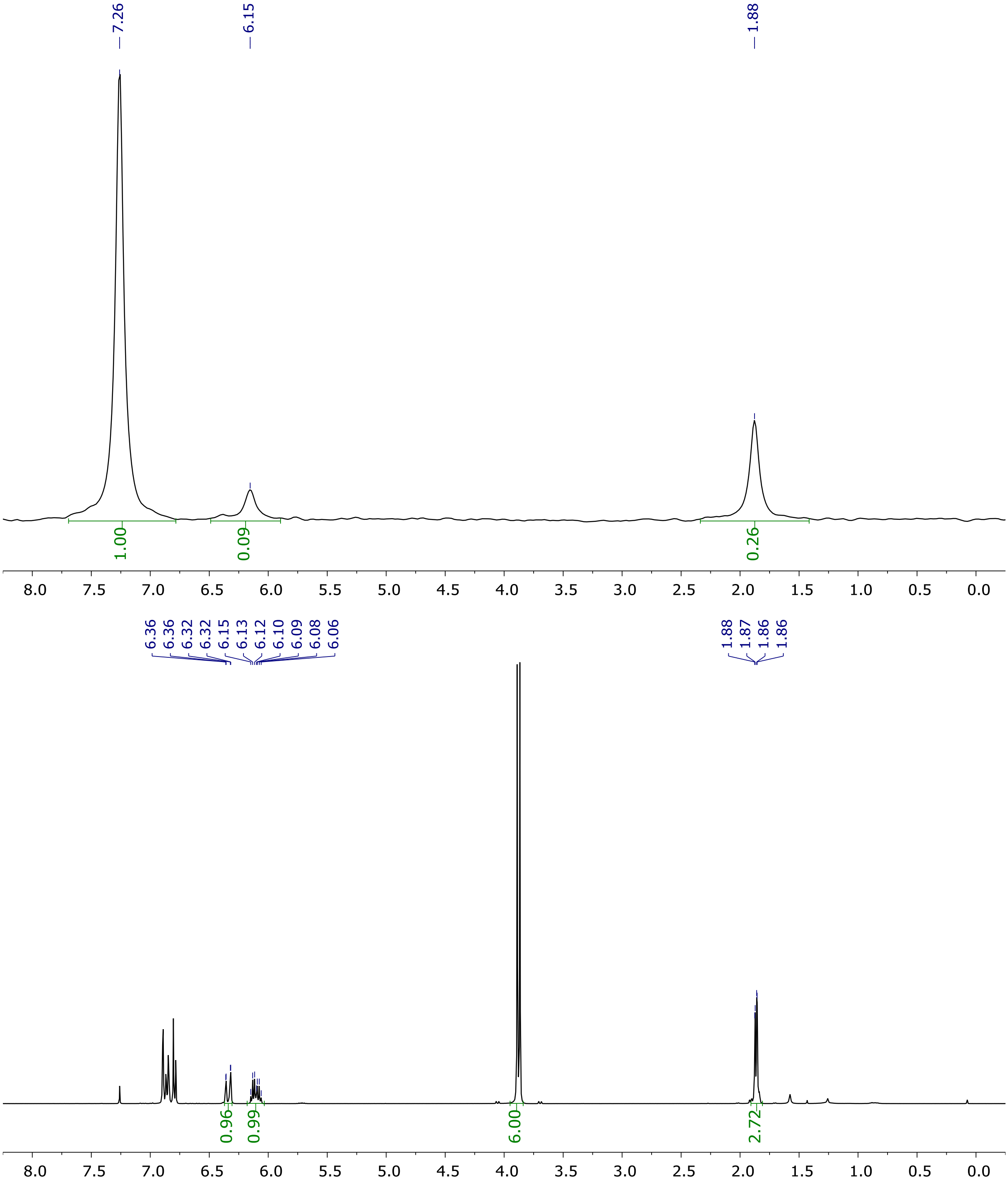

Figure S6 | ^{2}H NMR (top) and ^{1}H NMR (bottom) of **44b**.

7.4. Deuteration experiments

Reaction with Pd(I) iodo dimer D3

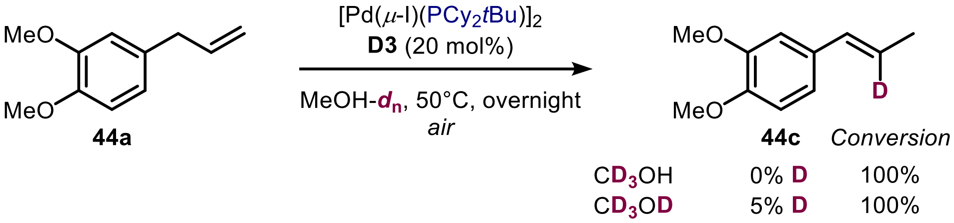

In air Pd(I) iodo dimer **D3** (20.0 mg, 0.02 mmol, 0.2 equiv.) was weighed into a 5 mL vial. A solution of 4-allyl-1,2-dimethoxybenzene (17.9 mg, 0.1 mmol, 1.0 equiv) in 0.5 mL MeOH-d_n was added and the reaction mixture stirred at 50°C overnight. The obtained crude was purified by column chromatography (Hexane/EtOAc 90:10) to give the product as a colorless liquid (70% (CD_3OH), 89% (CD_3OD); *E:Z* 99:1, based on GC-MS; R_f = 0.57 (Hexane/EtOAc 80:20)). The isolated products were analysed by ^2H NMR to determine the deuterium content as 0% and 5% in CD_3OH and CD_3OD, respectively.

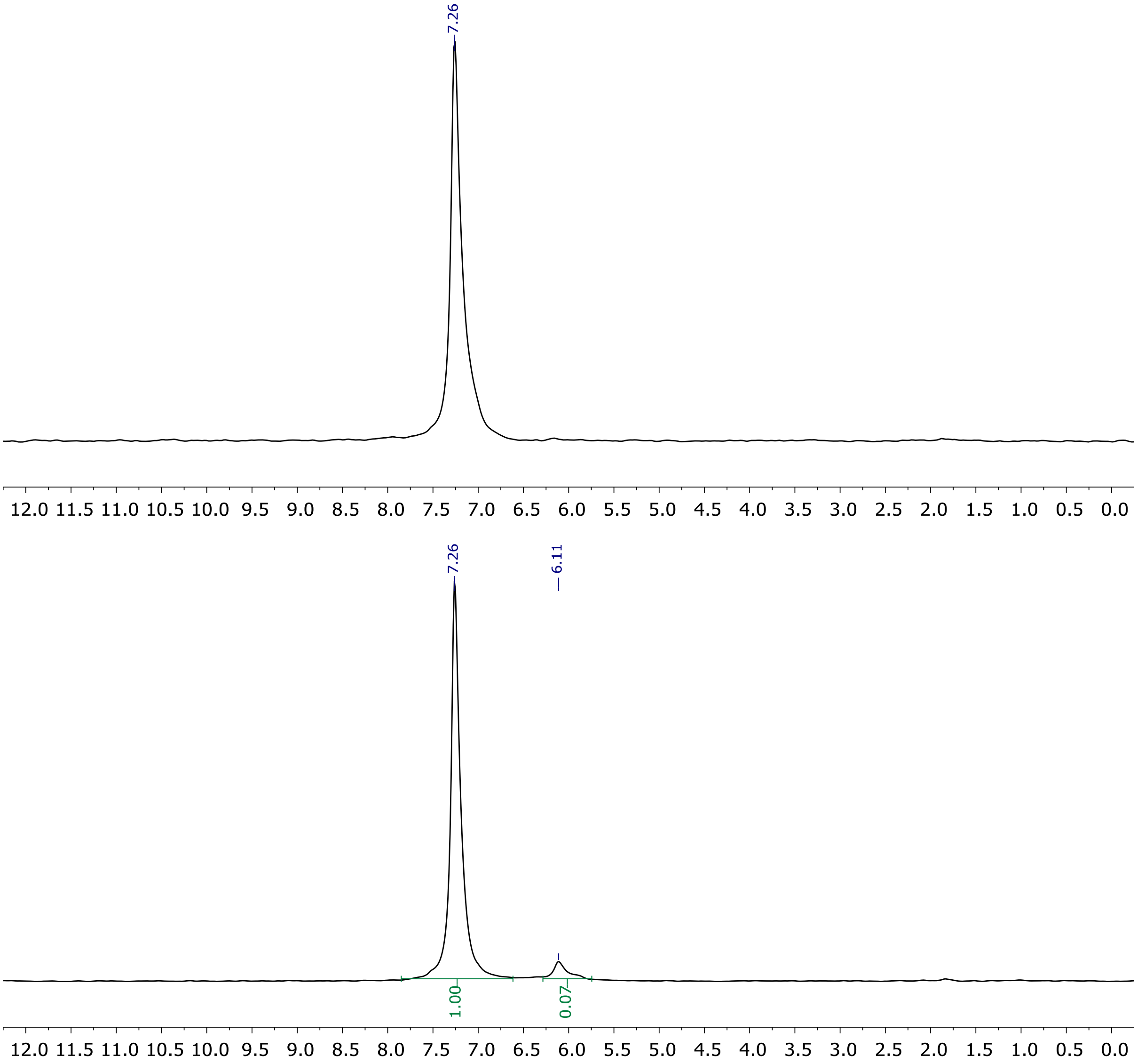

Figure S7 | ^2H NMR of the isolated products obtained from isomerization in CD_3OH (top) and CD_3OD (bottom).

Reaction with [(P*t*Bu$_3$)$_2$Pd(H)(Cl)] C2

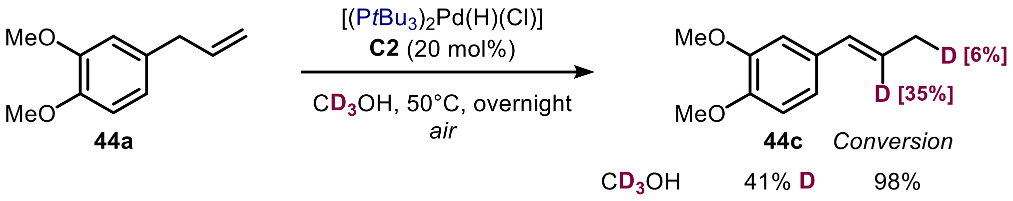

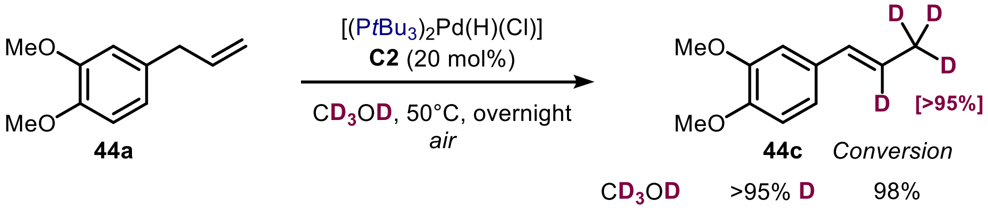

Inside an Ar filled glovebox, [(P*t*Bu$_3$)$_2$Pd(H)(Cl)] **C2**[23] (22.0 mg, 0.04 mmol, 0.2 equiv.) was weighed into a 5 mL vial. A solution of 4-allyl-1,2-dimethoxybenzene (36.1 mg, 0.2 mmol, 1.0 equiv) in MeOH-d_n (1.0 mL) was added and the reaction mixture stirred at 50°C overnight. The obtained crude was purified by column chromatography (Hexane/EtOAc 90:10) to give the product as a colorless liquid (75% (CD_3OH), 82% (CD_3OD); *E*:*Z* 99:1, based on GC-MS; R_f = 0.57 (Hexane/EtOAc 80:20)). The isolated products were analysed by ^{1}H-NMR and ^{2}H-NMR to determine the extent of deuteration (see Figure S8 and Figure S9).

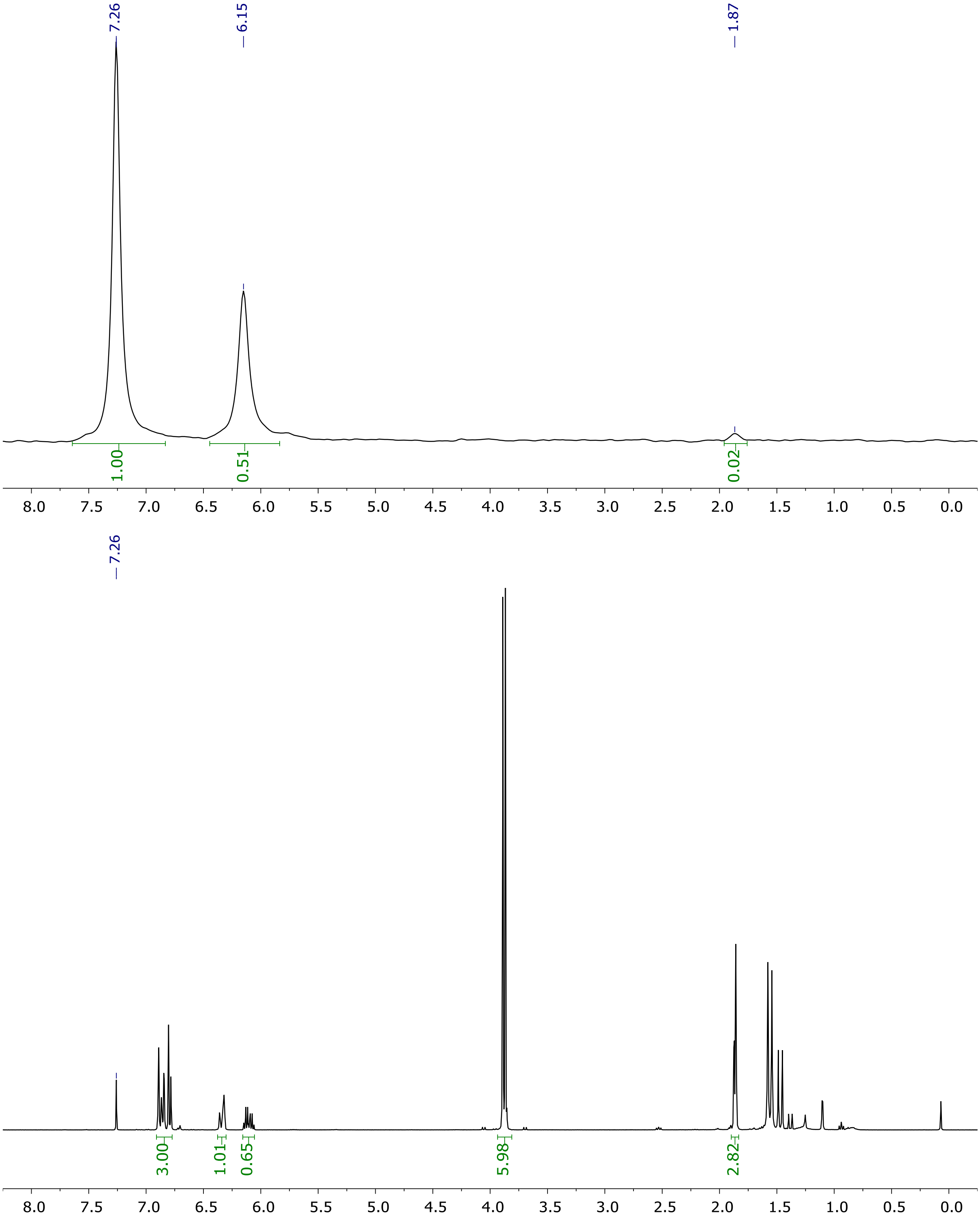

Figure S8 | ^{2}H NMR (top) and ^{1}H NMR (bottom) analysis of product **44c** obtained from the reaction in CD_3OH.

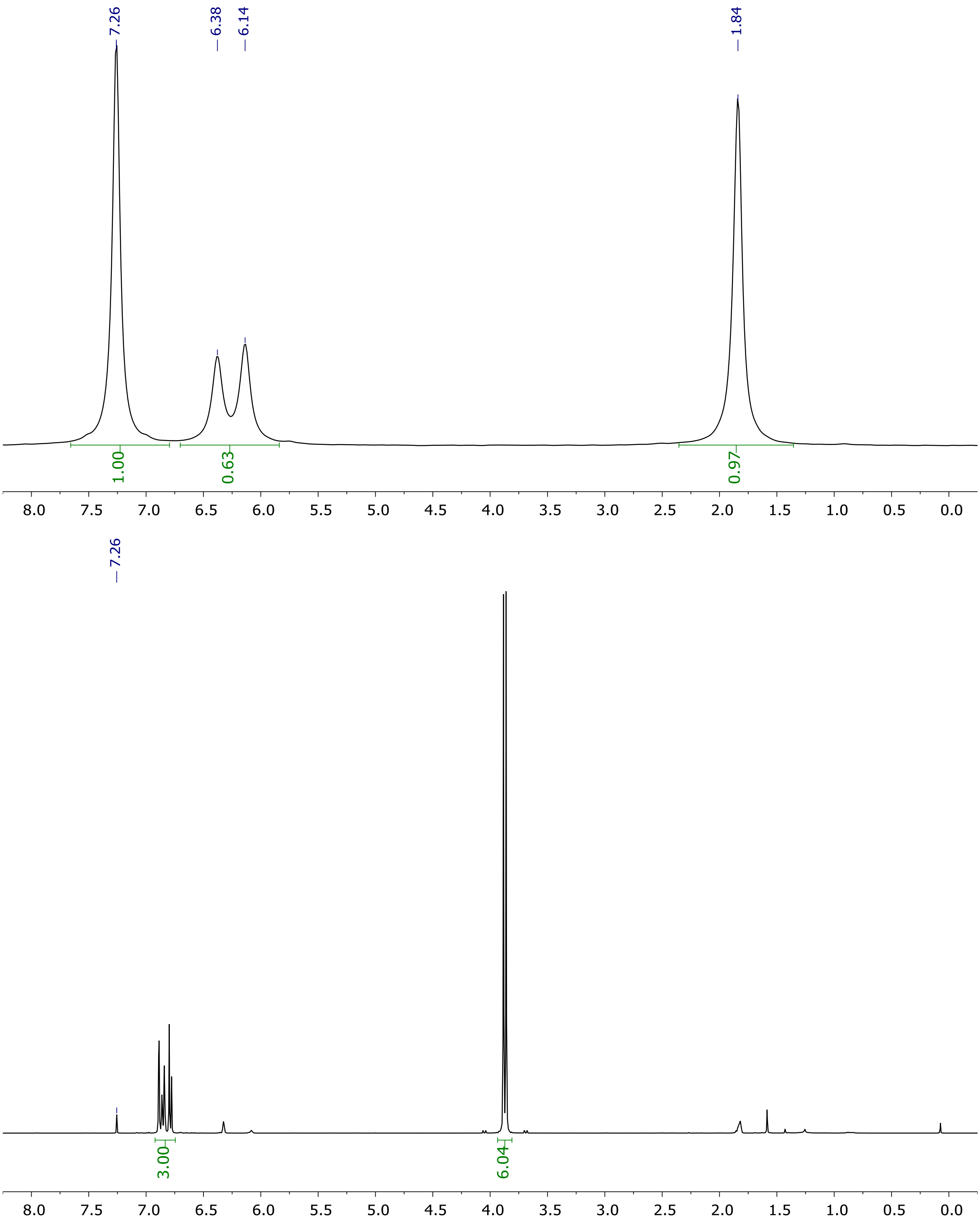

Figure S9 | ^{2}H NMR (top) and ^{1}H NMR (bottom) analysis of product **44c** obtained from the reaction in CD_3OD.

Reaction with [(PCy$_2$*t*Bu)$_2$Pd(H)(Cl)] C3

[(PCy$_2$*t*Bu)$_2$Pd(H)(Cl)] **C3** was prepared as stated in 7.2.

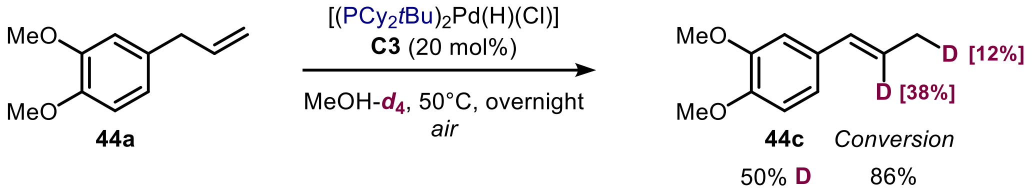

Inside an Ar filled glovebox, [(PCy$_2$*t*Bu)$_2$Pd(H)(Cl)] **C3** (26.1 mg, 0.04 mmol, 0.2 equiv.) was weighed into a 5 mL vial. A solution of 4-allyl-1,2-dimethoxybenzene (35.9 mg, 0.2 mmol, 1.0 equiv) in MeOH-*d*$_4$ (1.0 mL) was added and the reaction mixture stirred at 50°C overnight. The obtained crude was purified by column chromatography (Hexane/EtOAc 90:10) to give the product (65%; *E:Z* 99:1, based on GC-MS; R_f = 0.57 (Hexane/EtOAc 80:20)) along with 10% of an inseperable reduced side-product. The isolated product was analysed by ^{1}H-NMR and ^{2}H-NMR to determine the extent of deuteration (see Figure S10).

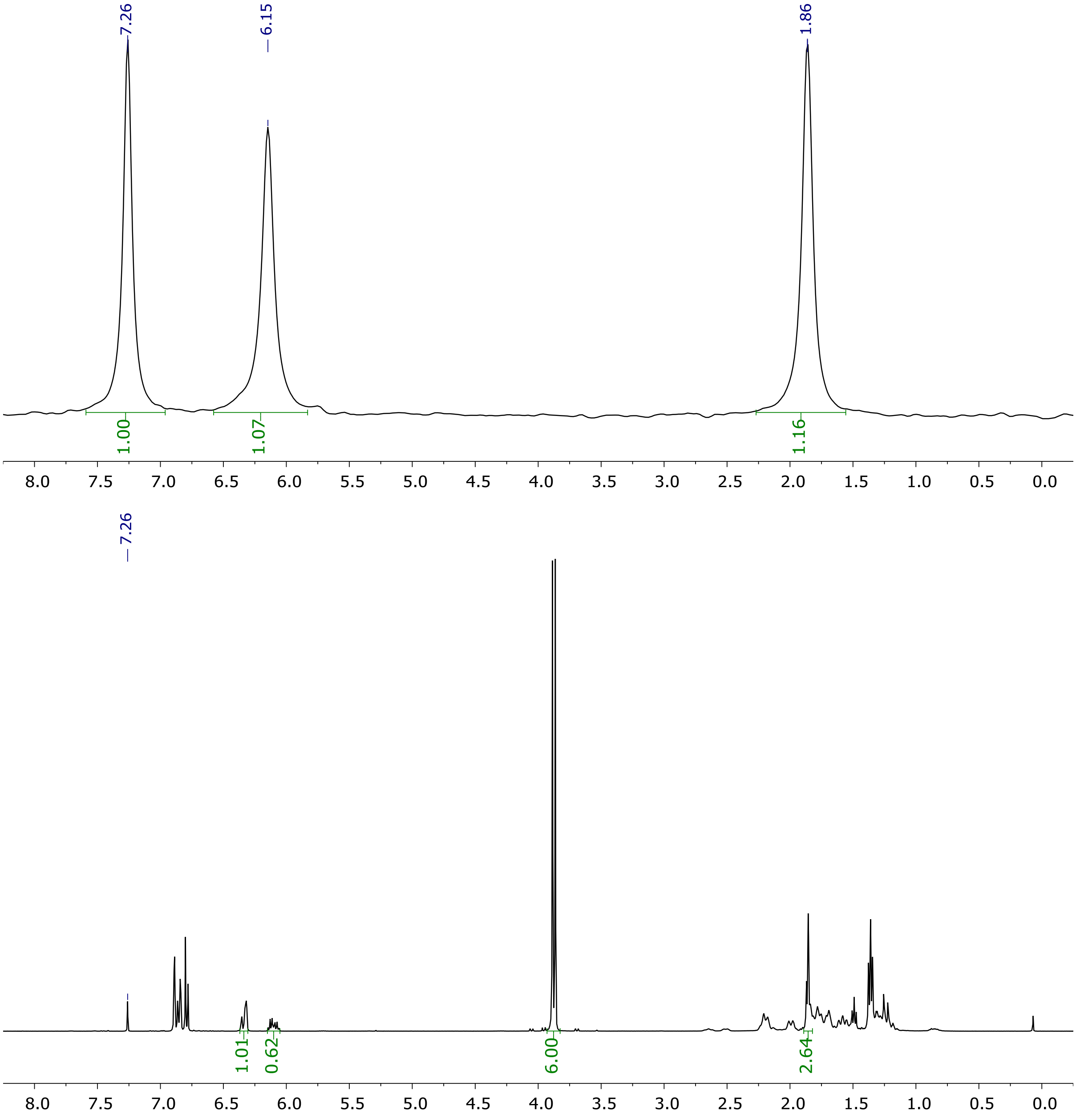

Figure S10 | ^{2}H NMR (top) and ^{1}H NMR (bottom) analysis of product **44c** obtained from the reaction in CD$_3$OD.

8. Crystallographic Data

Purple needles of $[Pd(\mu\text{-}I)(PCy_2\textit{t}Bu)]_2$ (**D3**) were obtained by diffusion from a mixture of acetone and toluene. A suitable crystal was selected and mounted on a glass fibre with grease on a Bruker APEX-II CCD diffractometer. The crystals were kept at T = 296 K during data collection. The structures were solved with the ShelXT[25] structure solution program using the direct solution method and by using Olex2[26] as the graphical interface. The model was refined with ShelXL[27] using least squares minimization.

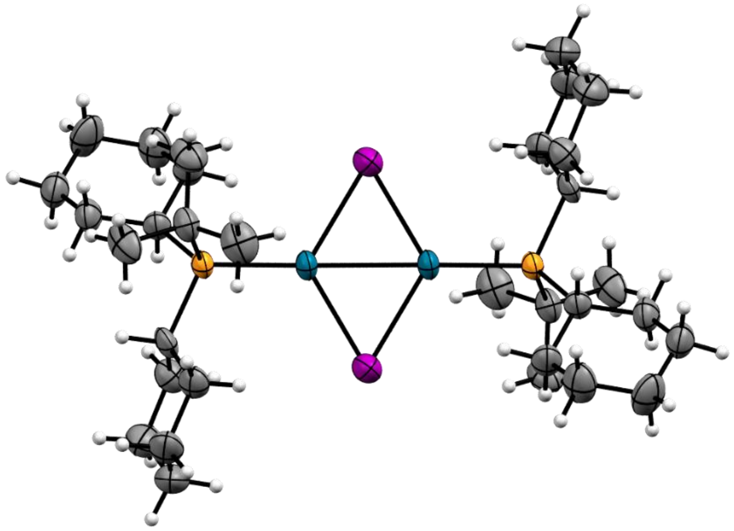

Table S1 | Crystal data and structure refinement for **D3**.

CCDC	2008108
Empirical formula	$C_{32}H_{62}I_2P_2Pd_2$
Formula weight	975.35
Temperature/K	296(2)
Crystal system	triclinic
Space group	*P*-1
a/Å	8.0594(4)
b/Å	10.6219(6)
c/Å	11.7927(5)
α/°	77.493(4)
β/°	86.775(4)
γ/°	68.103(5)
Volume/Å^3	914.13(9)
Z	1
ρ_{calc}g/cm^3	1.772
μ/mm^{-1}	2.779
F(000)	482.0
Crystal size/mm^3	0.470 × 0.171 × 0.145
Radiation	MoKα (λ = 0.71073)
2Θ range for data collection/°	3.538 to 49.992
Index ranges	-9 ≤ h ≤ 9, -12 ≤ k ≤ 12, -14 ≤ l ≤ 14
Reflections collected	29077
Independent reflections	3216 [R_{int} = 0.1274, R_{sigma} = 0.0457]
Data/restraints/parameters	3216/0/175
Goodness-of-fit on F^2	1.203
Final R indexes [I>=2σ (I)]	R_1 = 0.0708, wR_2 = 0.1565
Final R indexes [all data]	R_1 = 0.0803, wR_2 = 0.1632
Largest diff. peak/hole / e Å^{-3}	1.53/-0.95

9. HPLC traces of chiral compounds

AK Schoenebeck - Analytische HPLC

Sample Name:	GK 5-189 rac
Data file:	D:\GONZO\GK\189R1IC.D
Sample Info:	Mobile phase: n-Hexane/iPrOH 9:1;<br>The sample is solved in MP
Methode file:	IC.M
Column-info:	Chiralpak IC (150x4,6)mm
Operator:	Analytical Lab 4.03 - 4.04
Injektion Time:	09:19:01
Injektion Date:	26.03.2019

Instrument Conditions:	At Start	At Stop
Temperature in°C:	30.0	30.0
Pressure in bar:	24.6	24.3
Flow in ml/min:	1.00	1.00

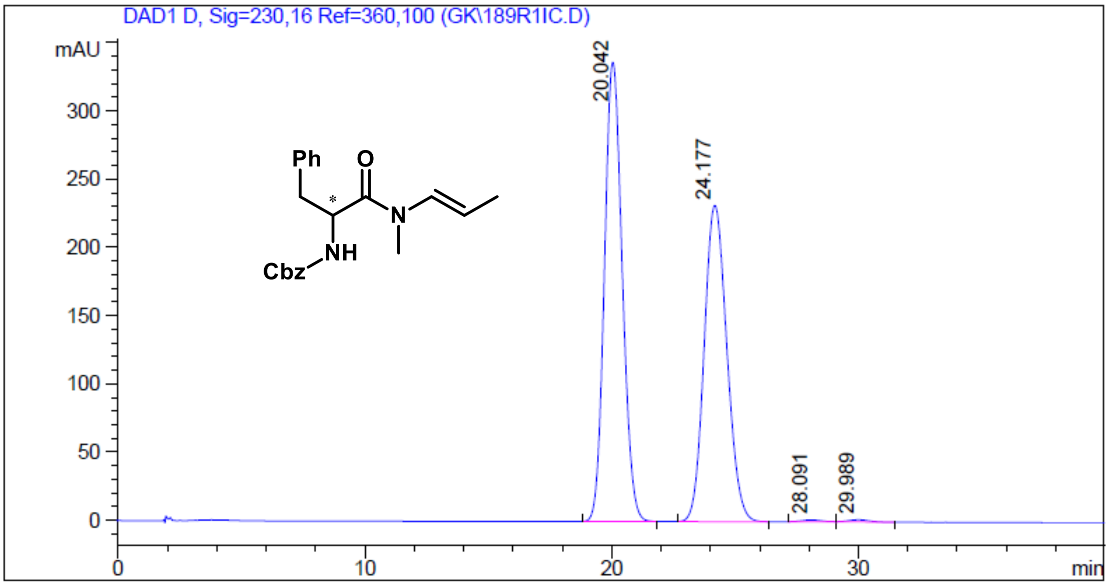

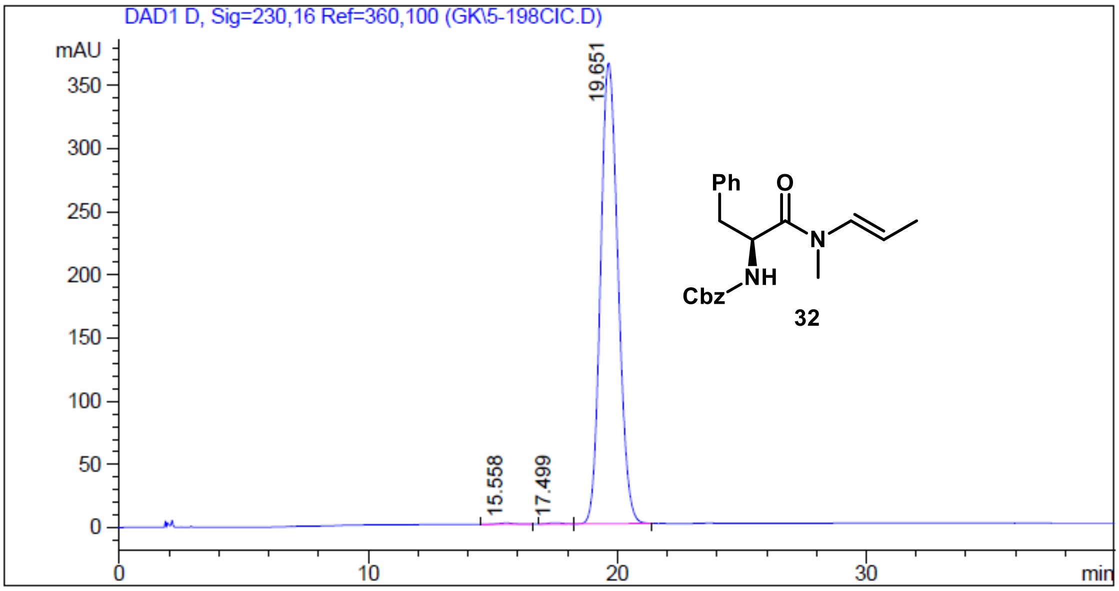

Sample Name:	GK 5-190 rac
Data file:	D:\GONZO\GK\190RIC.D
Sample Info:	Mobile phase: n-Hexane/EtOH 8:2; The sample is solved in MP
Methode file:	IC.M
Column-info:	Chiralpak IC (150x4,6)mm
Operator:	Analytical Lab 4.03 - 4.04
Injektion Time:	15:04:11
Injektion Date:	25.03.2019

Instrument Conditions:	At Start	At Stop
Temperature in°C:	30.0	30.0
Pressure in bar:	28.4	28.6
Flow in ml/min:	1.00	1.00

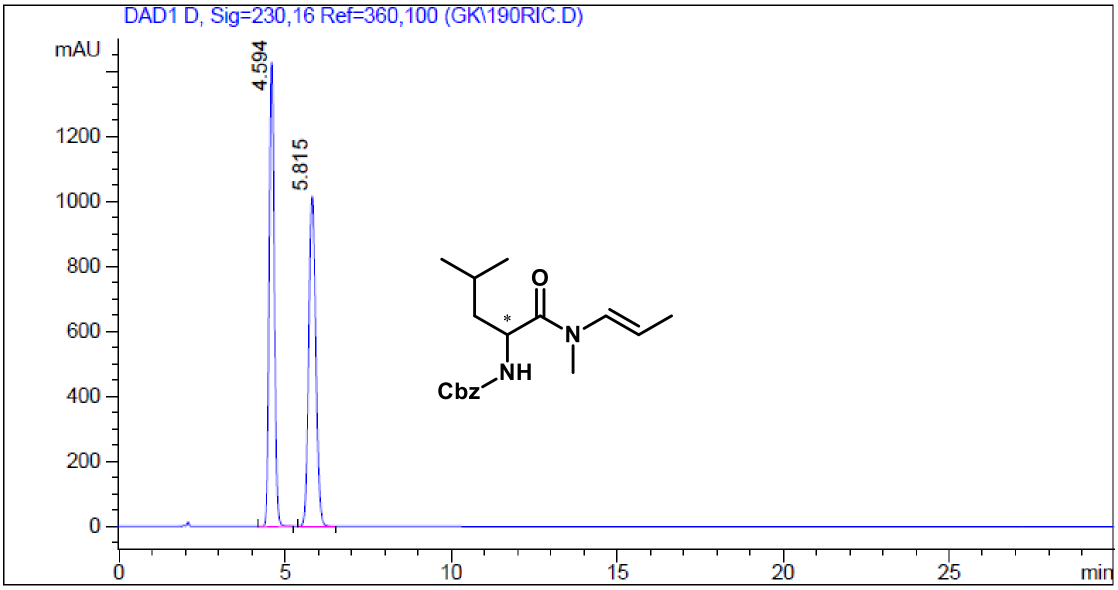

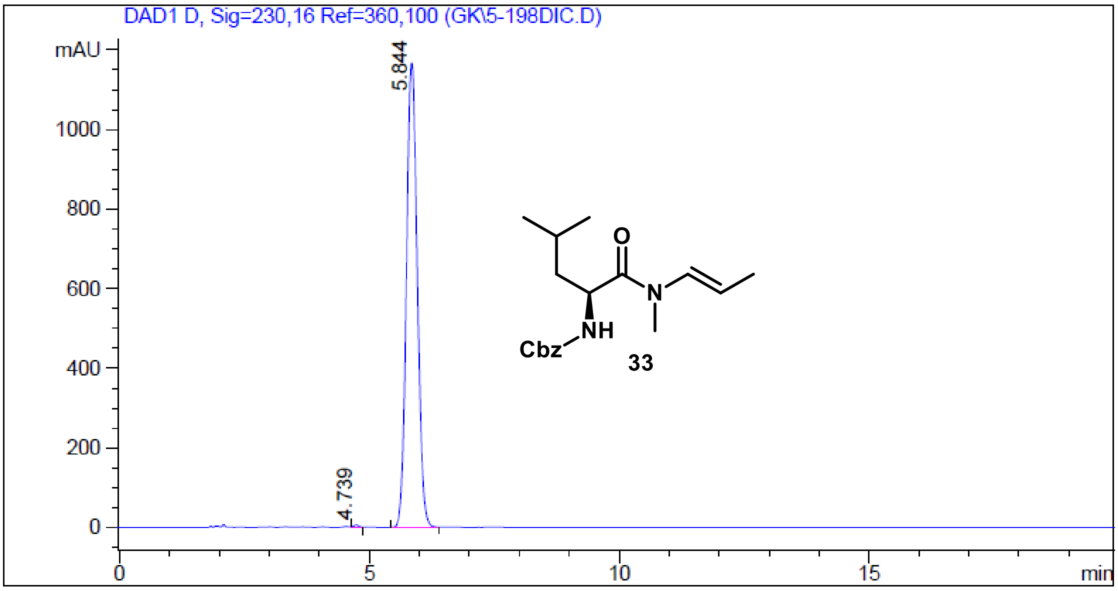

33

Sample Name:	GK 5-187 rac
Data file:	D:\GONZO\GK\187RIC.D
Sample Info:	Mobile phase: n-Hexane/EtOH 8:2; The sample is solved in MP
Methode file:	IC.M
Column-info:	Chiralpak IC (150x4,6)mm
Operator:	Analytical Lab 4.03 - 4.04
Injektion Time:	13:39:23
Injektion Date:	25.03.2019

Instrument Conditions:	At Start	At Stop
Temperature in°C:	30.0	30.0
Pressure in bar:	27.5	28.3
Flow in ml/min:	1.00	1.00

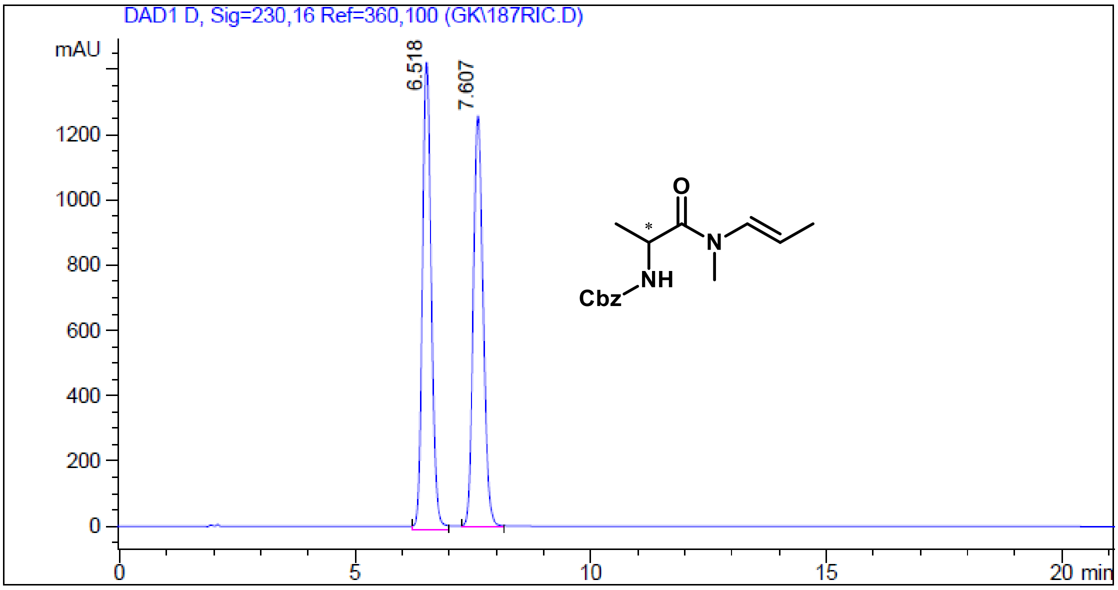

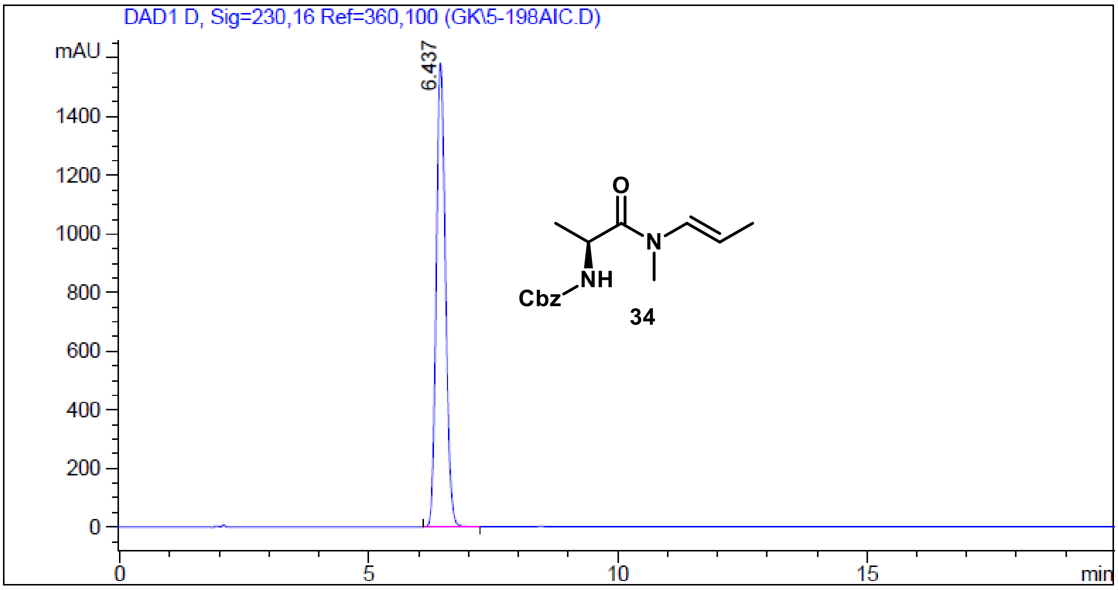

Sample Name:	GK 5-188 rac
Data file:	D:\GONZO\GK\188RIC.D
Sample Info:	Mobile phase: n-Hexane/EtOH 8:2; The sample is solved in MP
Methode file:	IC.M
Column-info:	Chiralpak IC (150x4,6)mm
Operator:	Analytical Lab 4.03 - 4.04
Injektion Time:	14:01:49
Injektion Date:	25.03.2019

Instrument Conditions:	At Start	At Stop
Temperature in°C:	30.0	30.0
Pressure in bar:	27.5	28.8
Flow in ml/min:	1.00	1.00

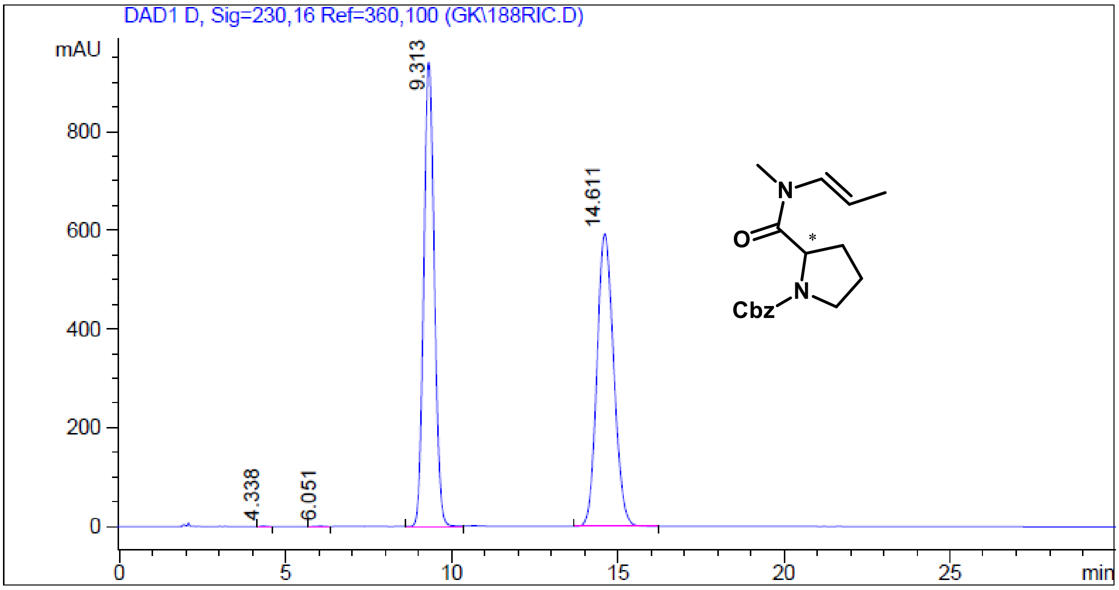

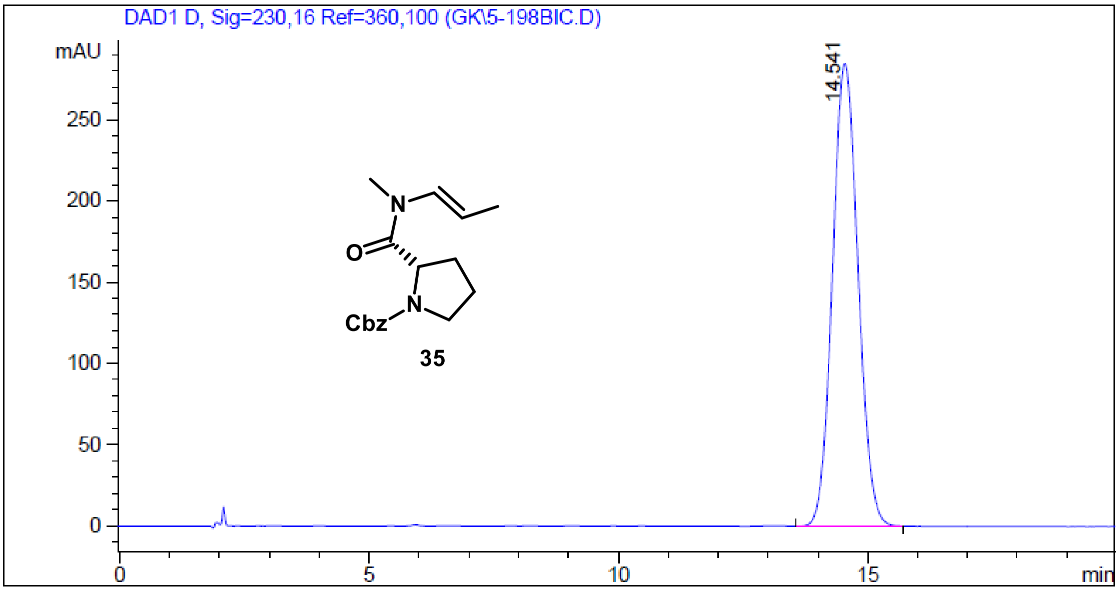

10. NMR Spectra

^{1}H (400 MHz, Benzene-d_6)

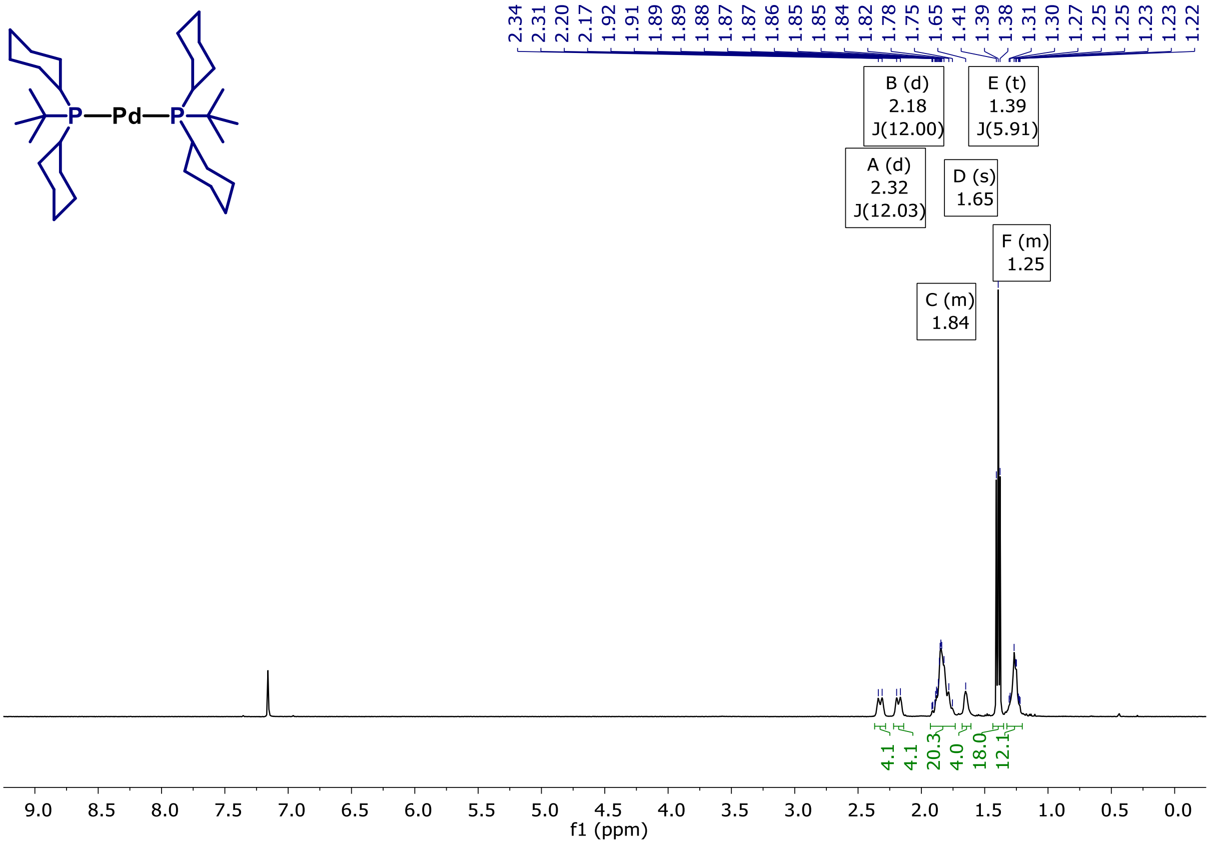

^{13}C (101 MHz, Benzene-d_6)

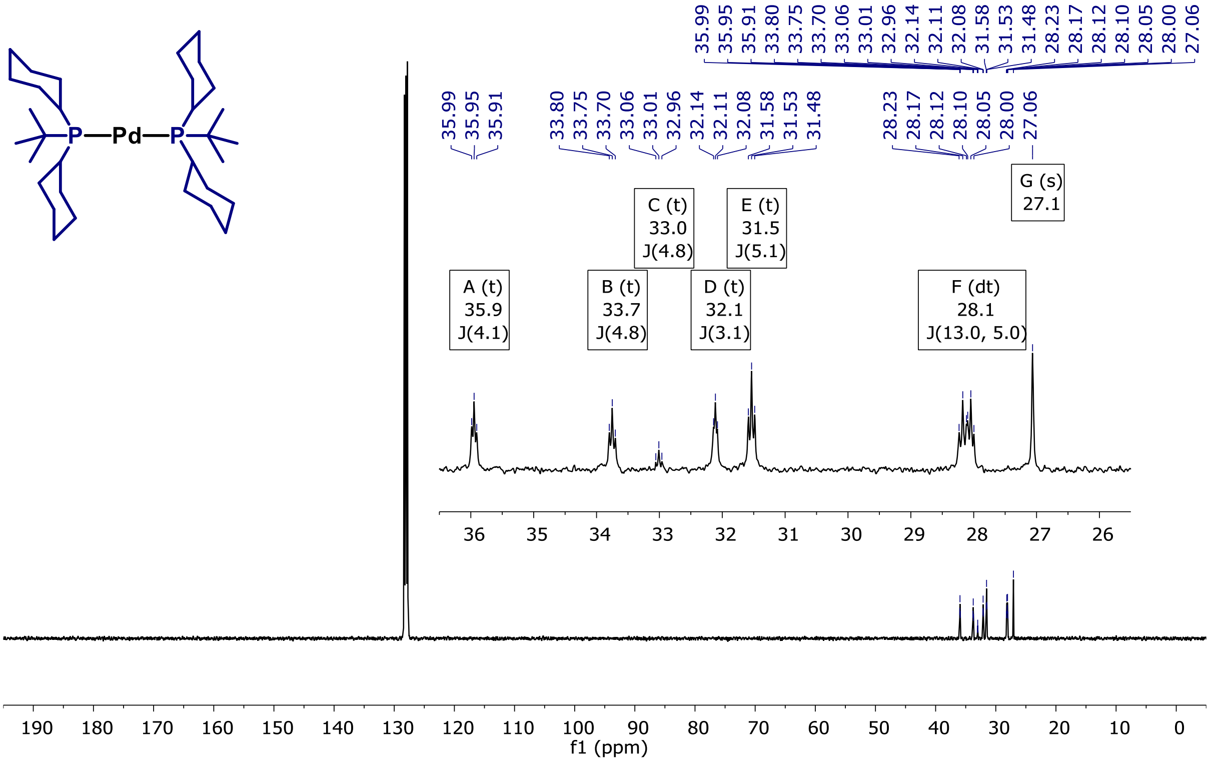

^{31}P (121 MHz, Toluene-d_8)

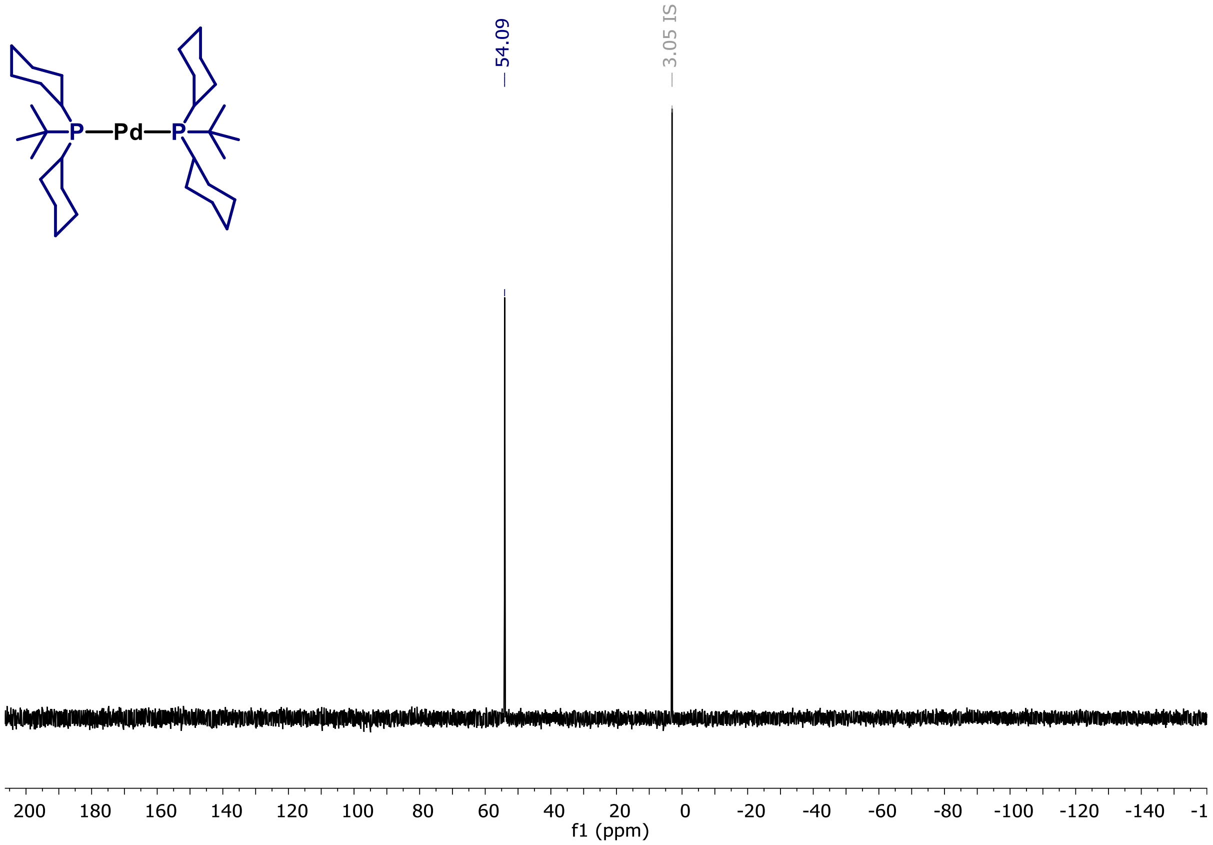

^{1}H (600 MHz, Benzene-d_6)

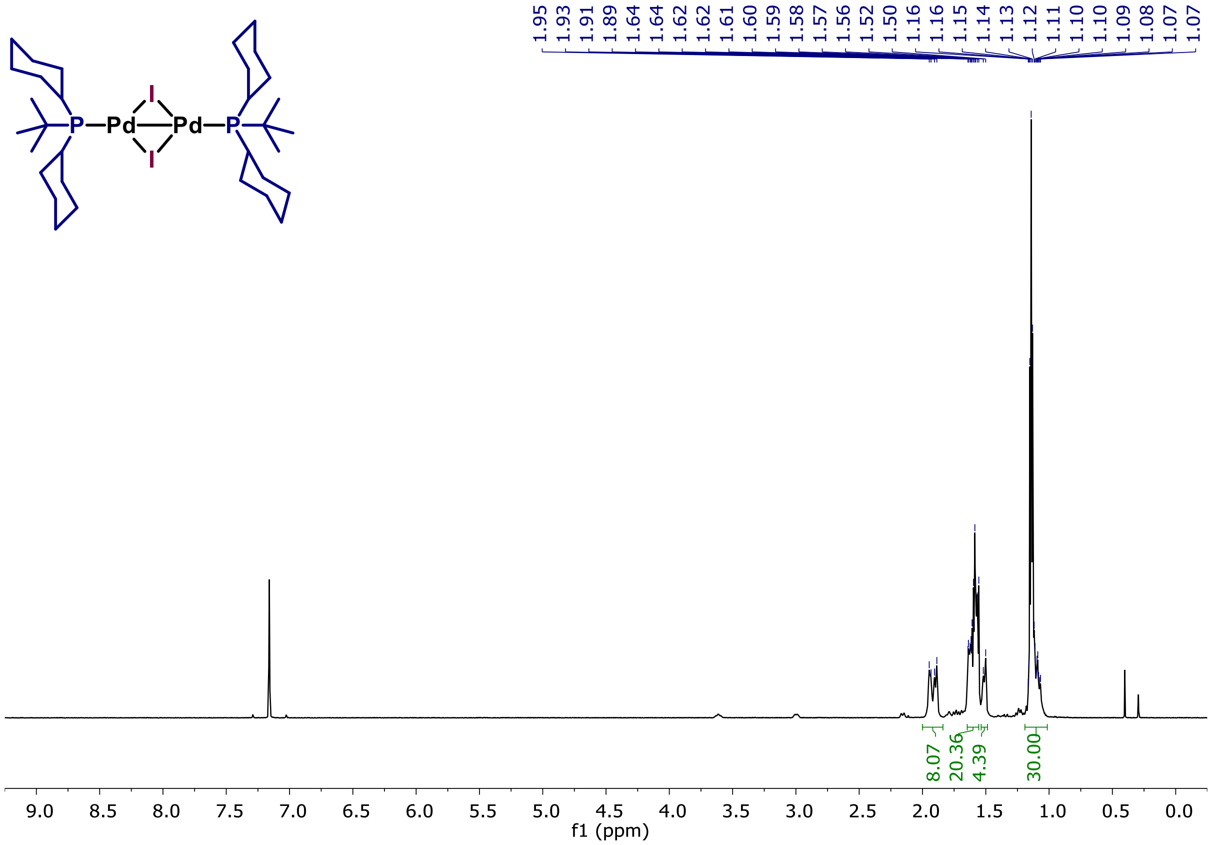

^{13}C (151 MHz, Benzene-d_6)

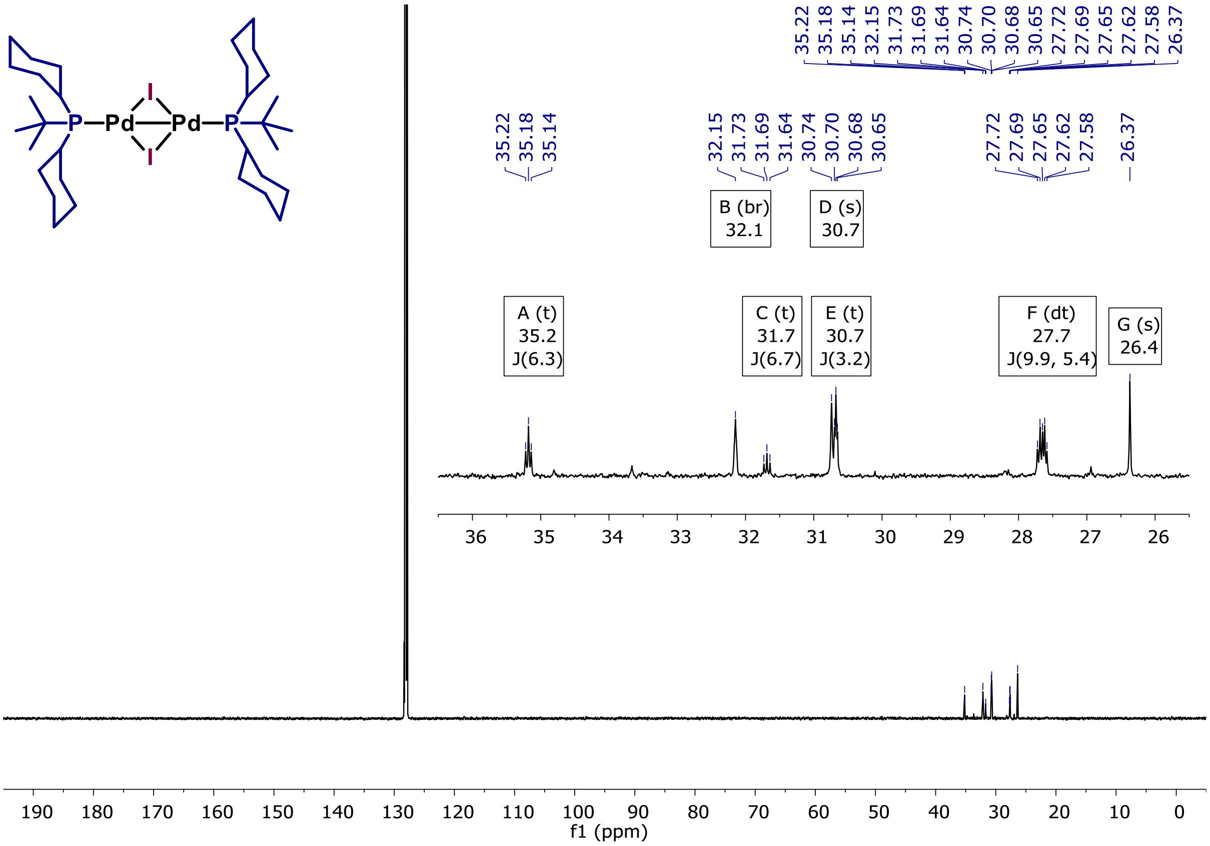

$^{31}P\{^1H\}$ (243 MHz, Benzene-d_6)

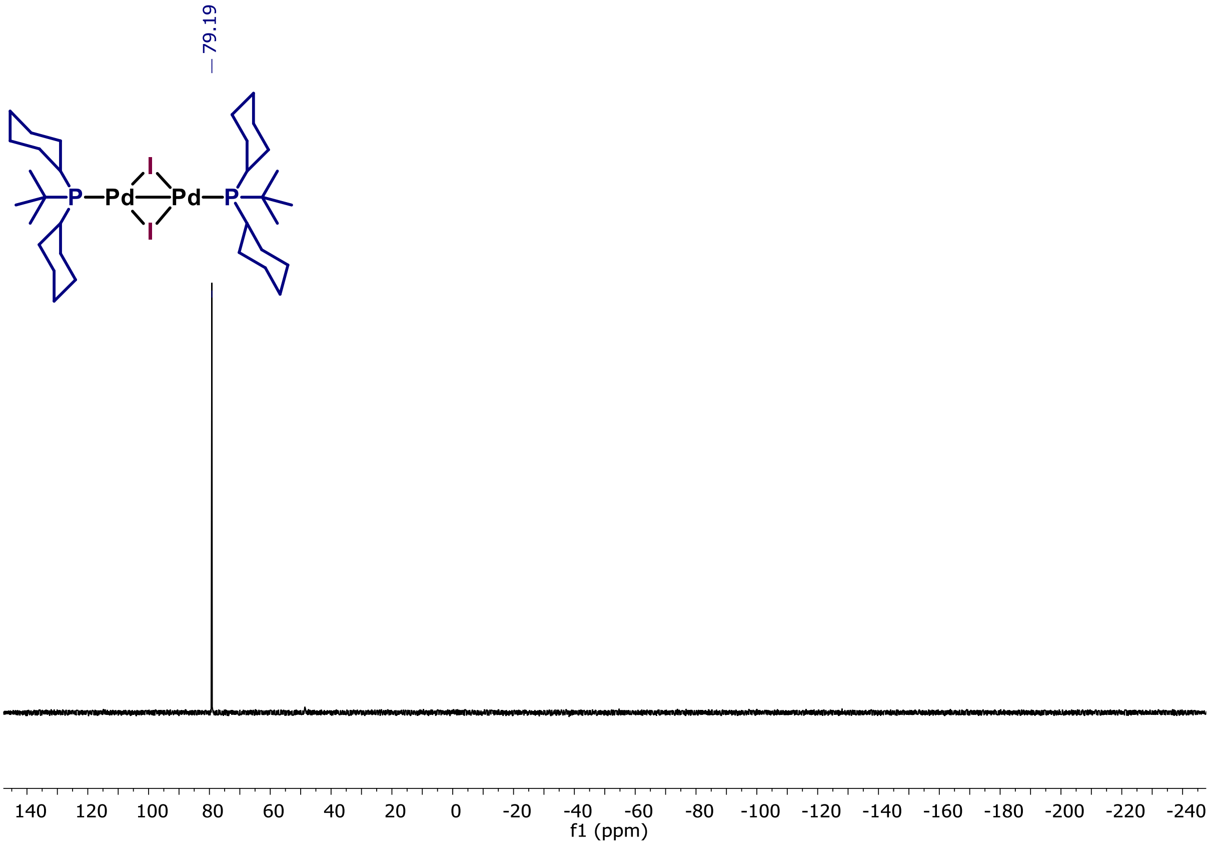

^1H (400 MHz, $CDCl_3$)

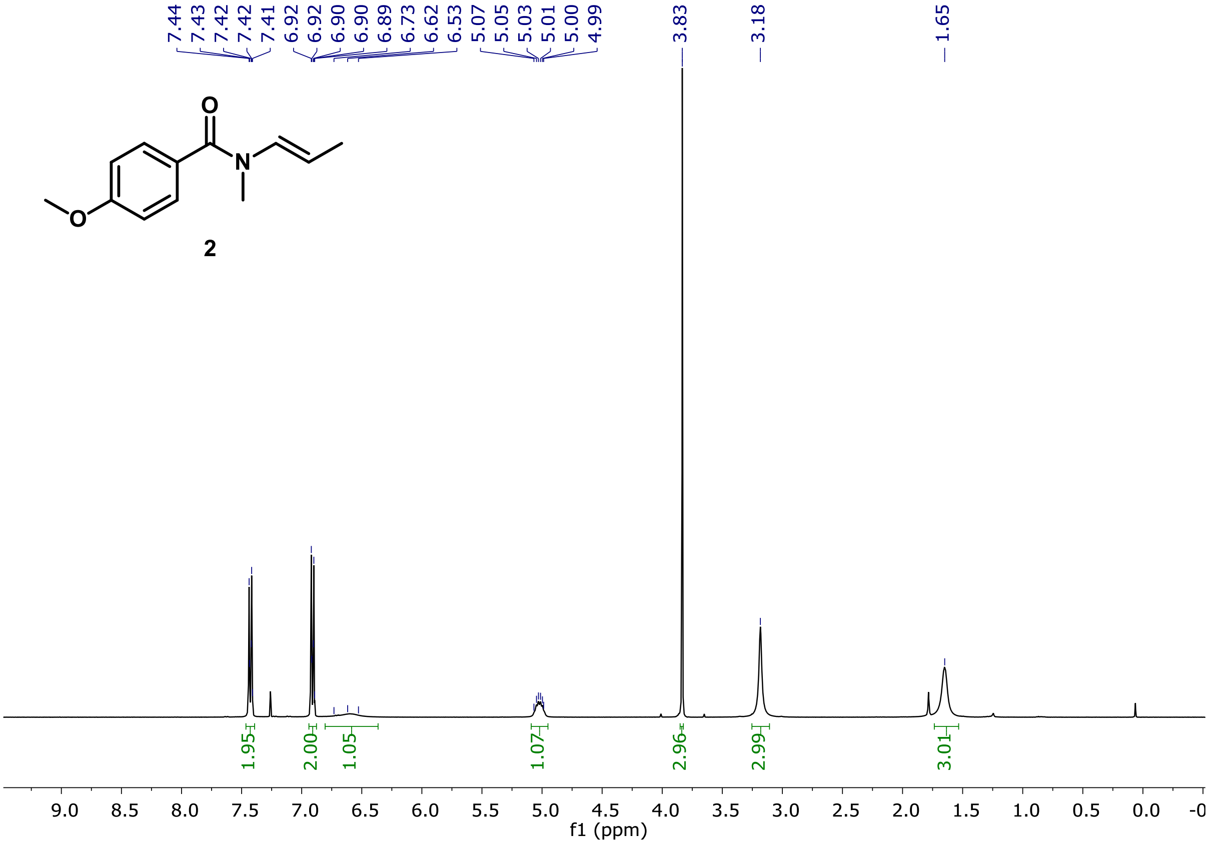

^{13}C (101 MHz, $CDCl_3$)

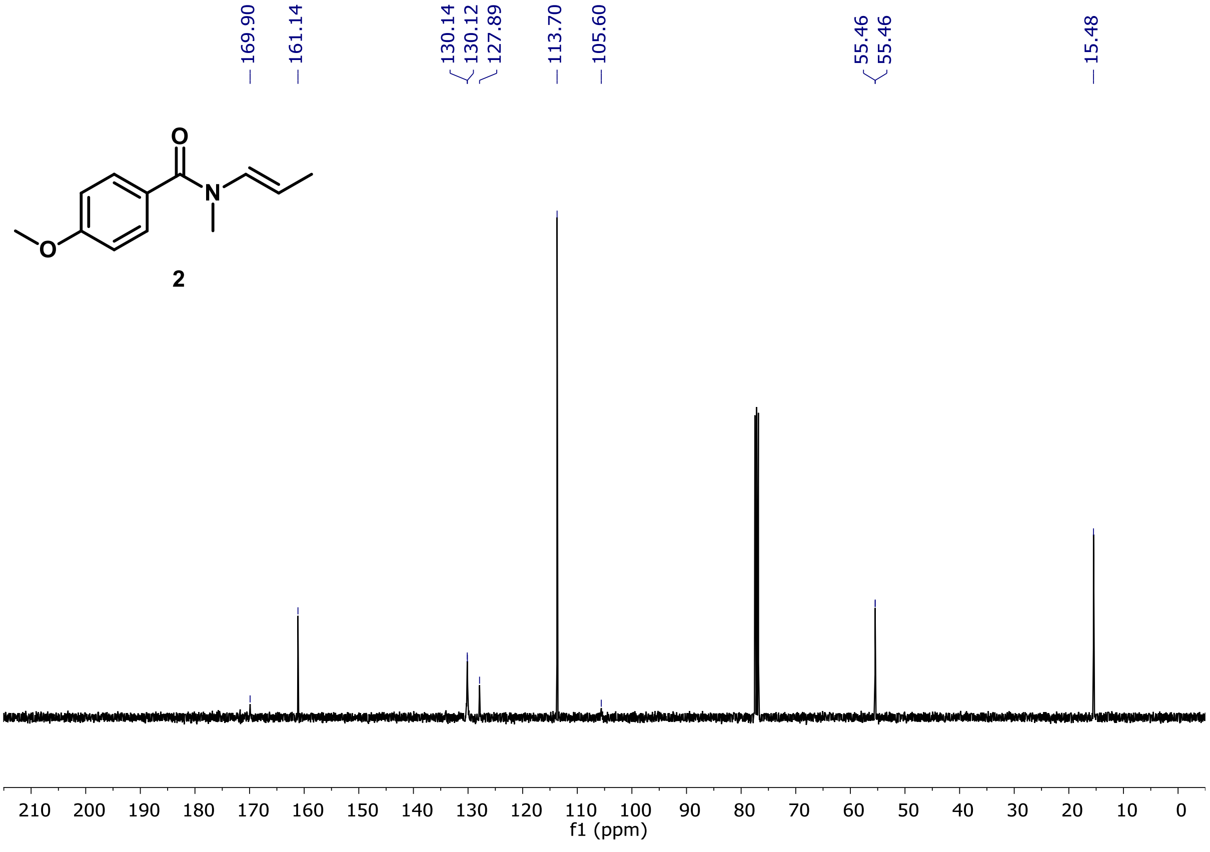

^{1}H (400 MHz, $CDCl_3$)

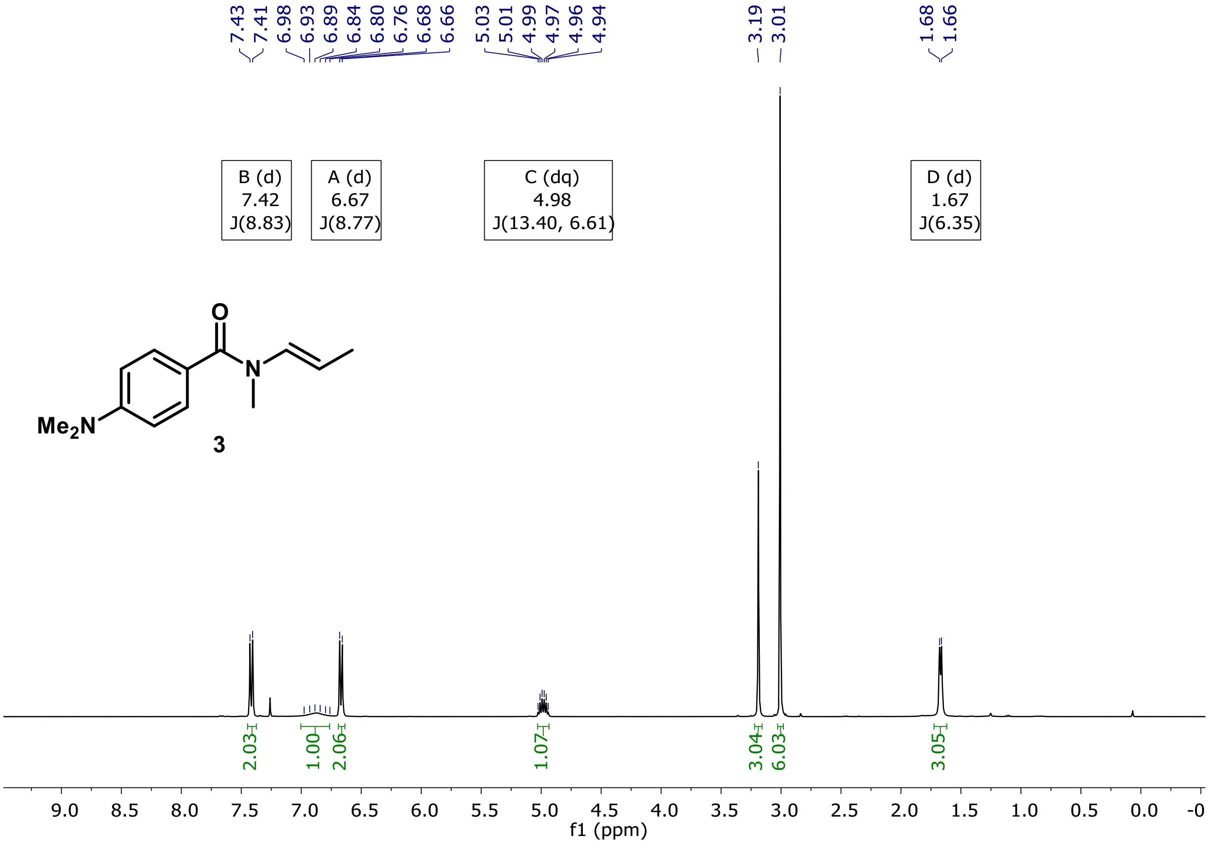

^{13}C (101 MHz, $CDCl_3$)

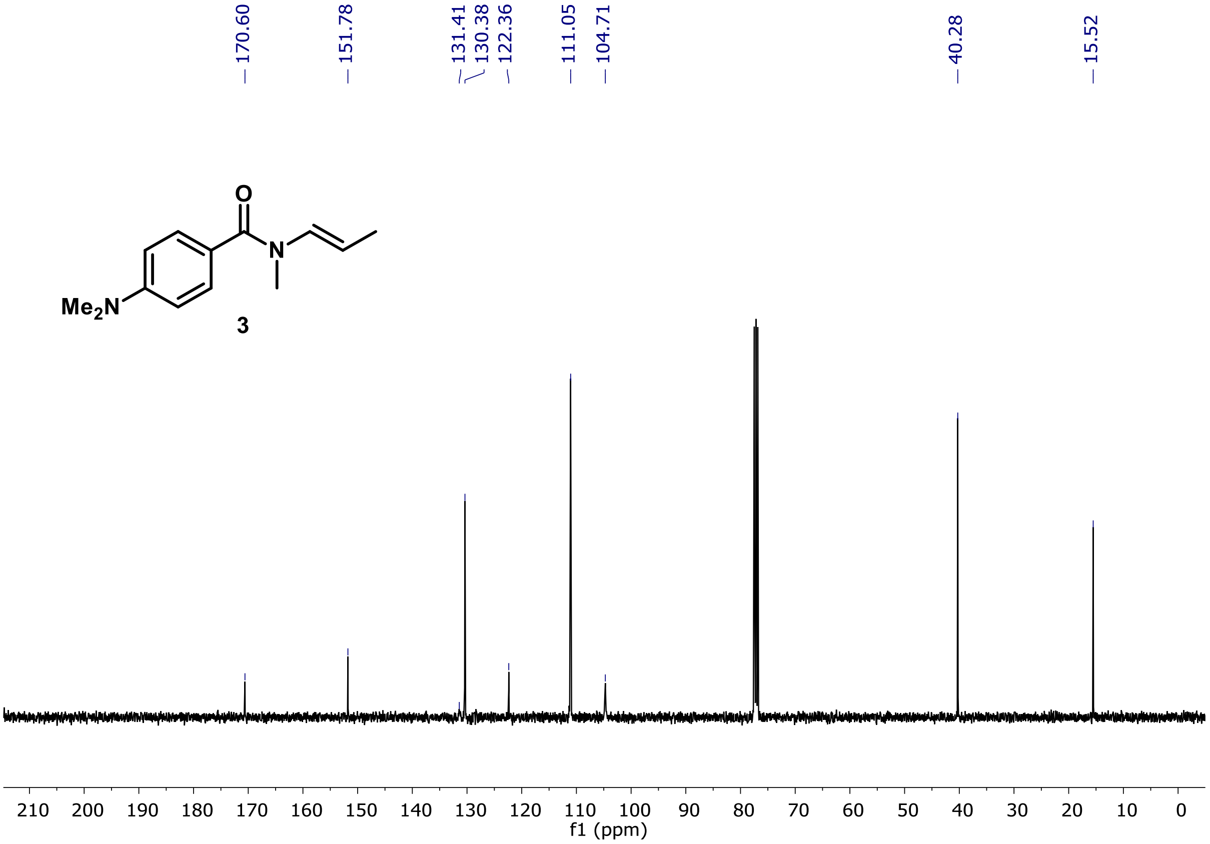

^{1}H (400 MHz, $CDCl_3$)

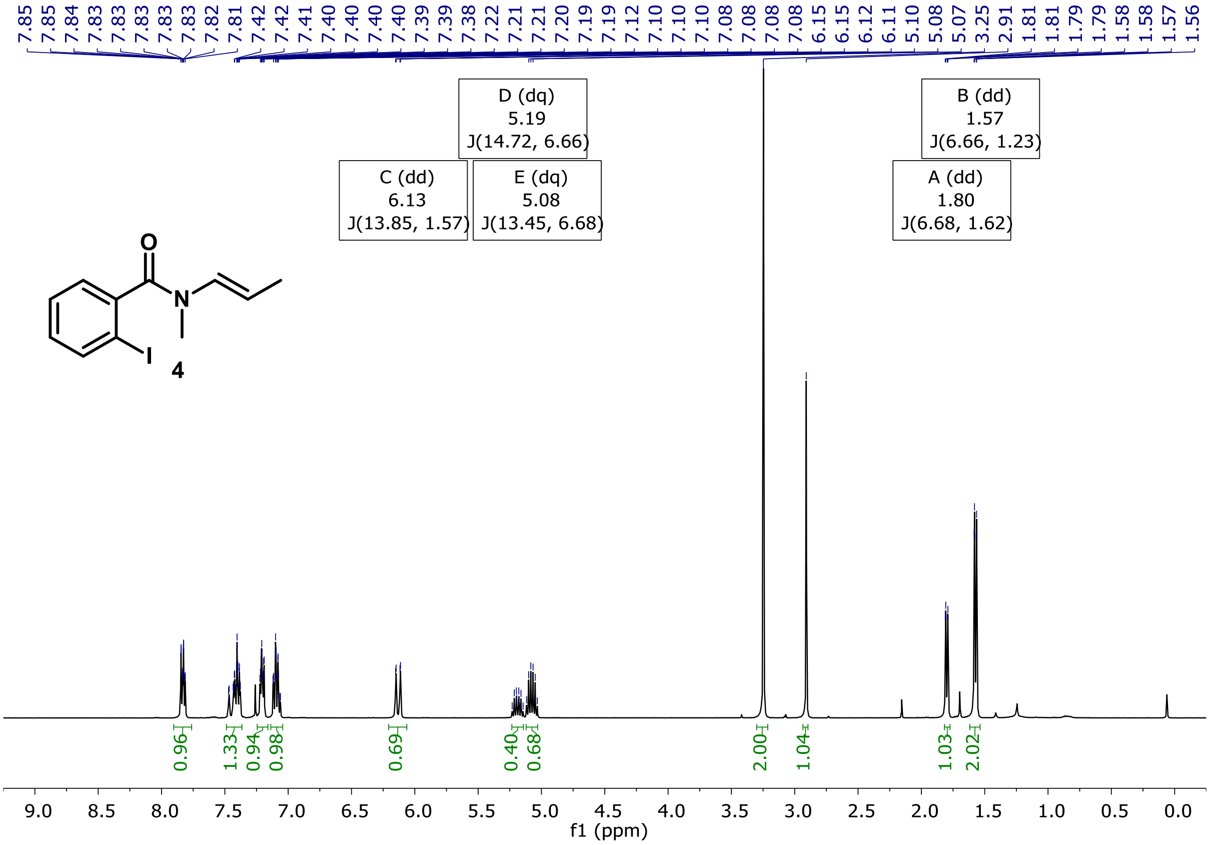

^{13}C (101 MHz, $CDCl_3$)

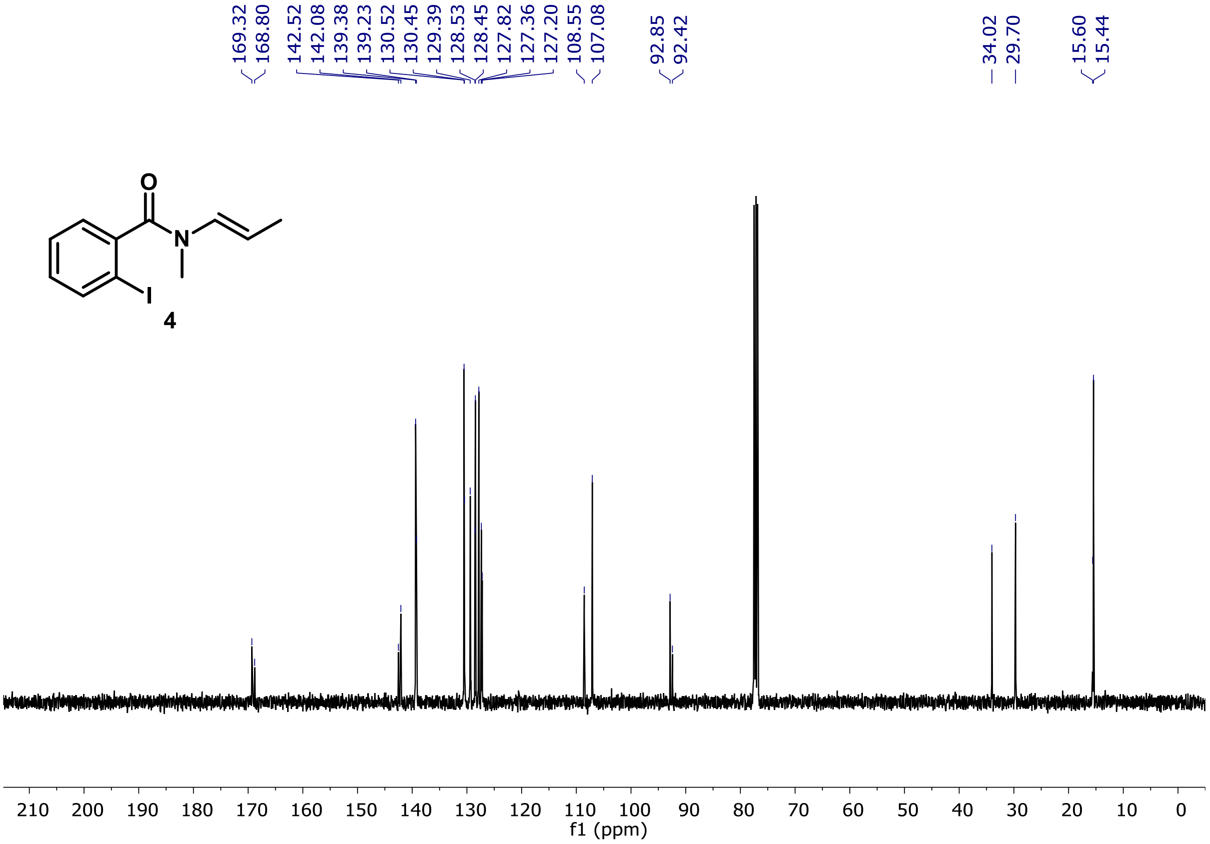

^{1}H (400 MHz, $CDCl_3$)

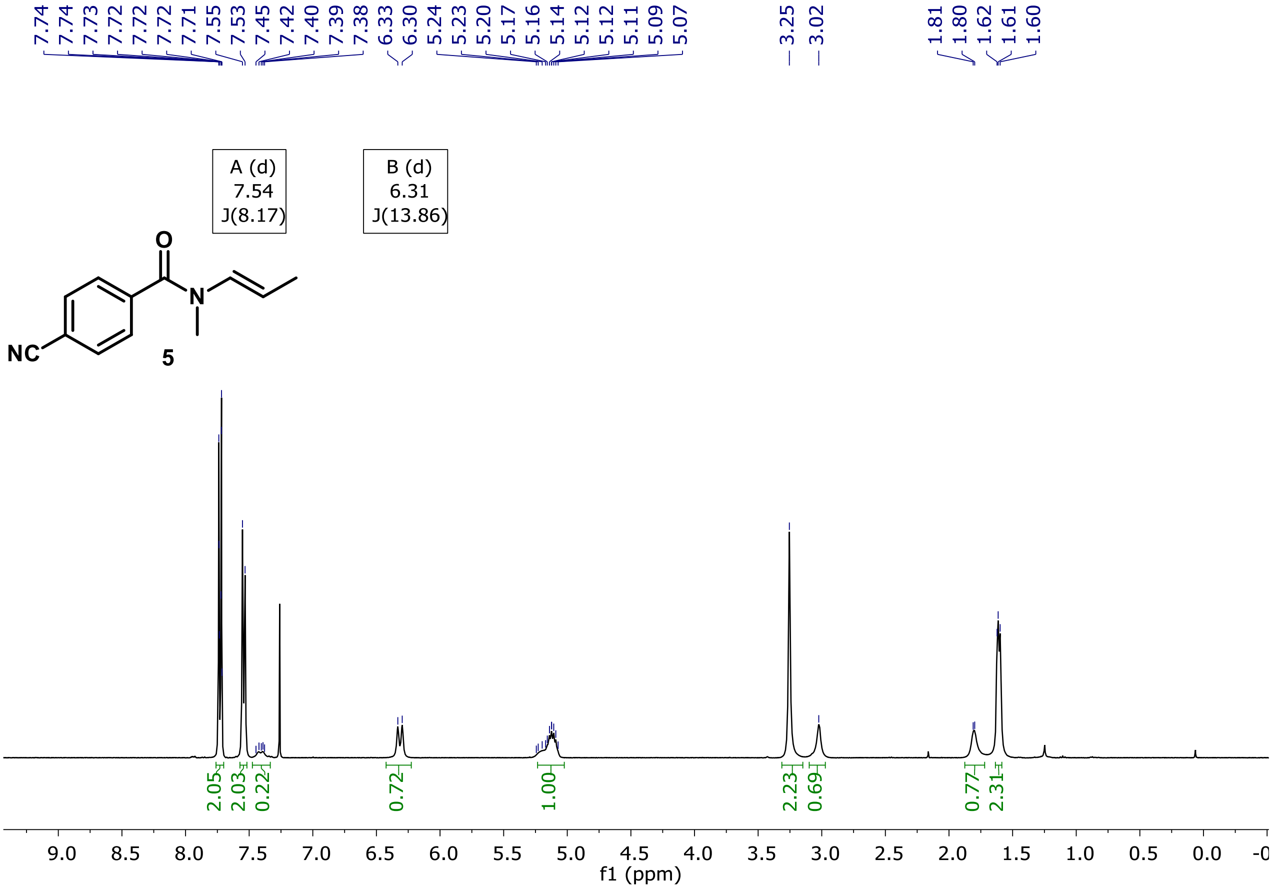

^{13}C (101 MHz, $CDCl_3$)

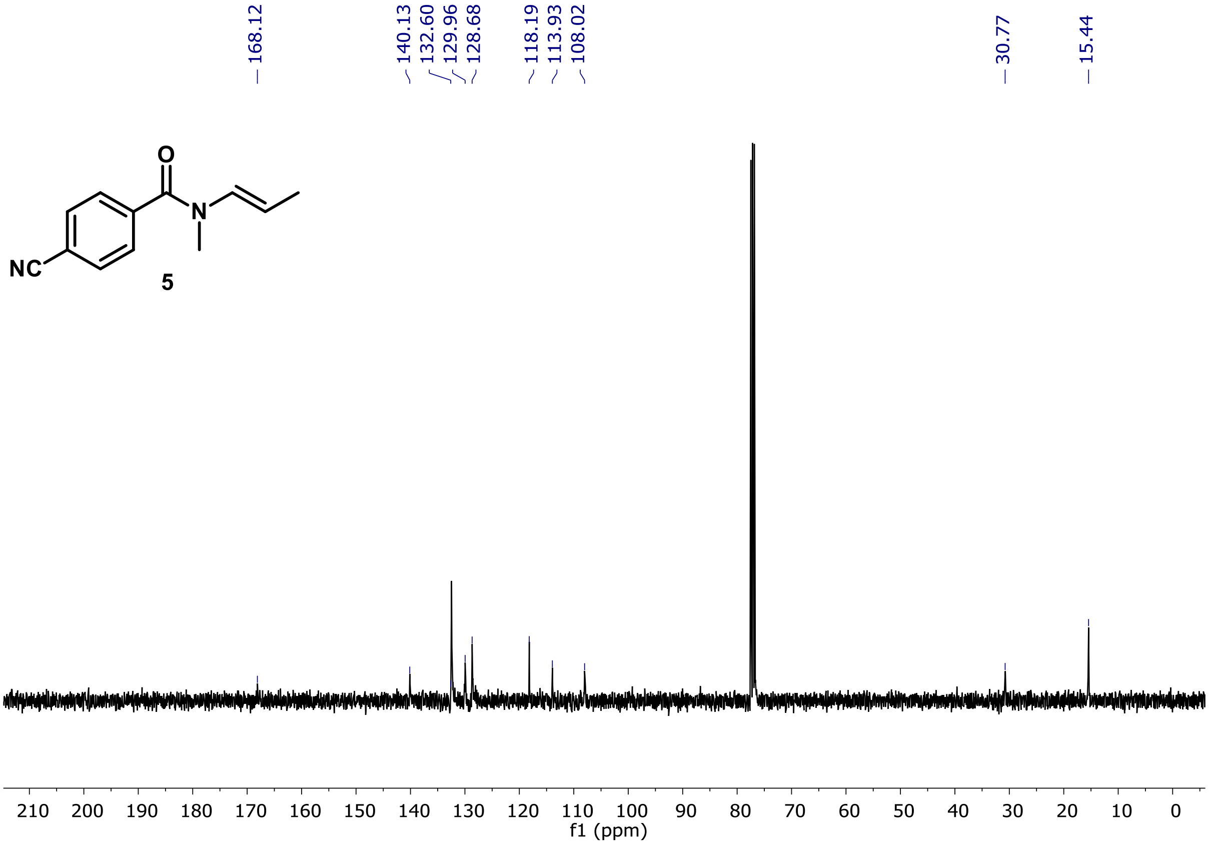

^{1}H (400 MHz, $CDCl_3$)

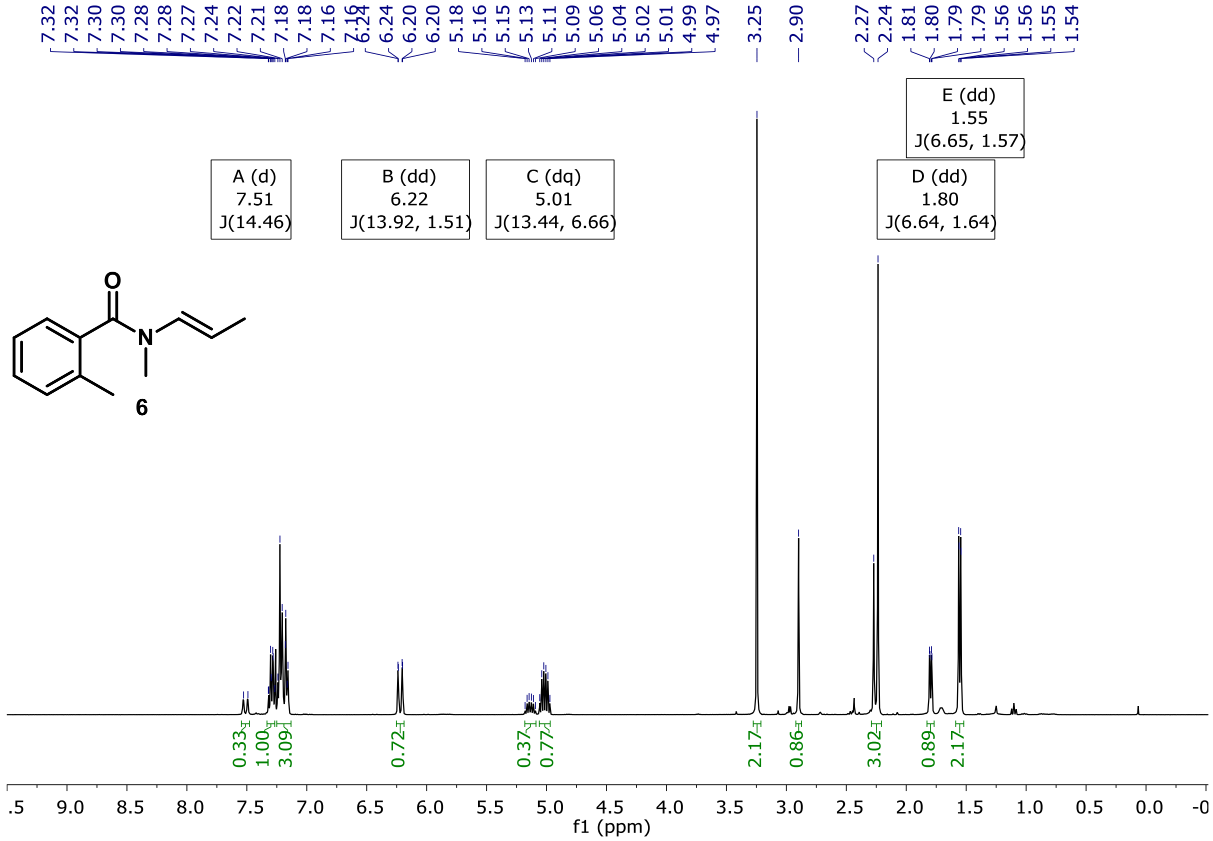

^{13}C (101 MHz, $CDCl_3$)

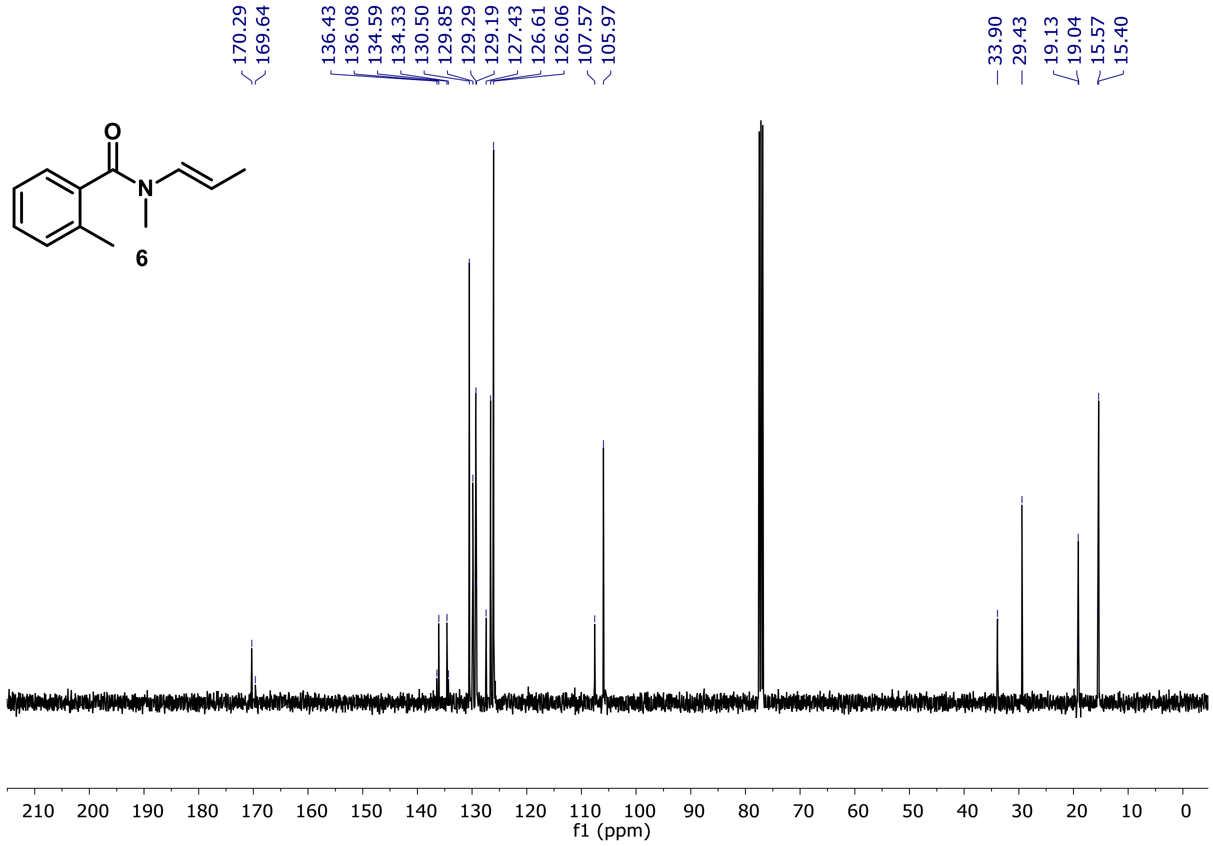

^1H (400 MHz, $CDCl_3$)

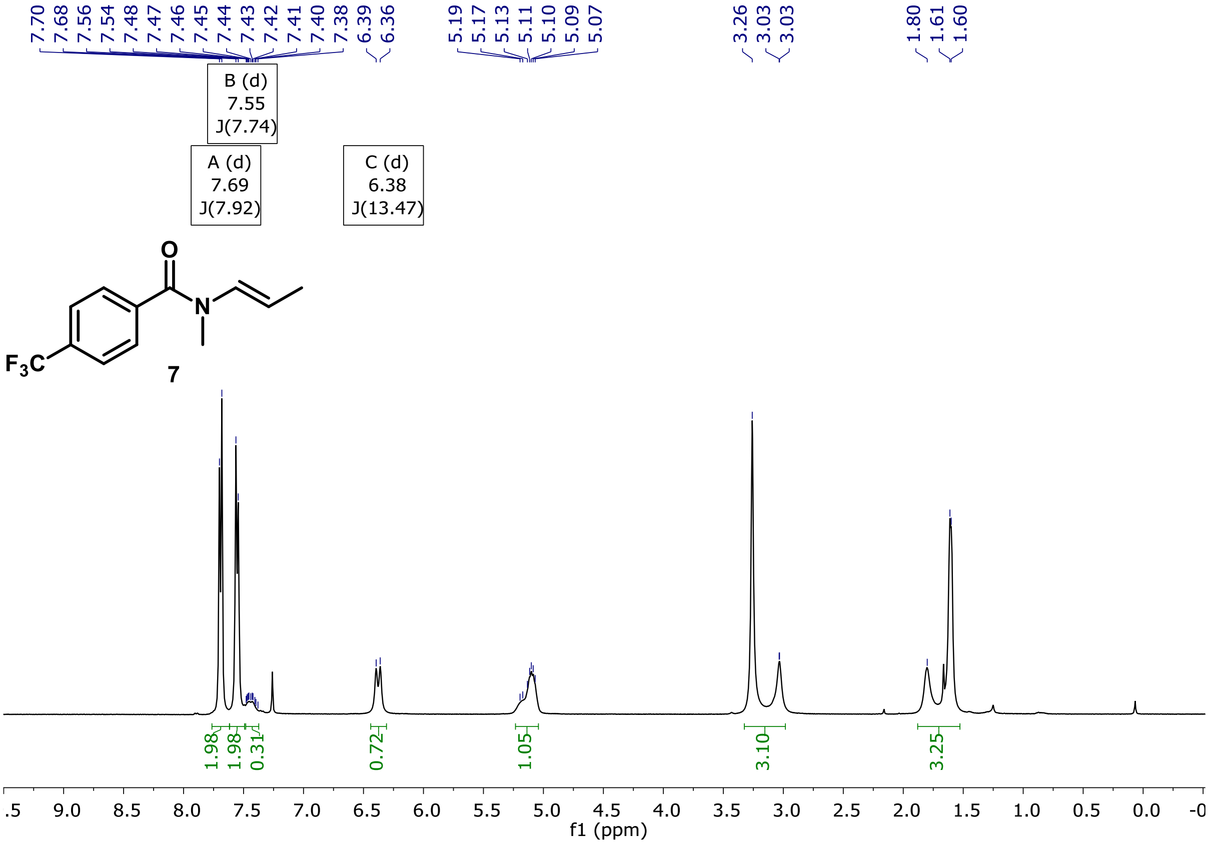

^{13}C (101 MHz, $CDCl_3$)

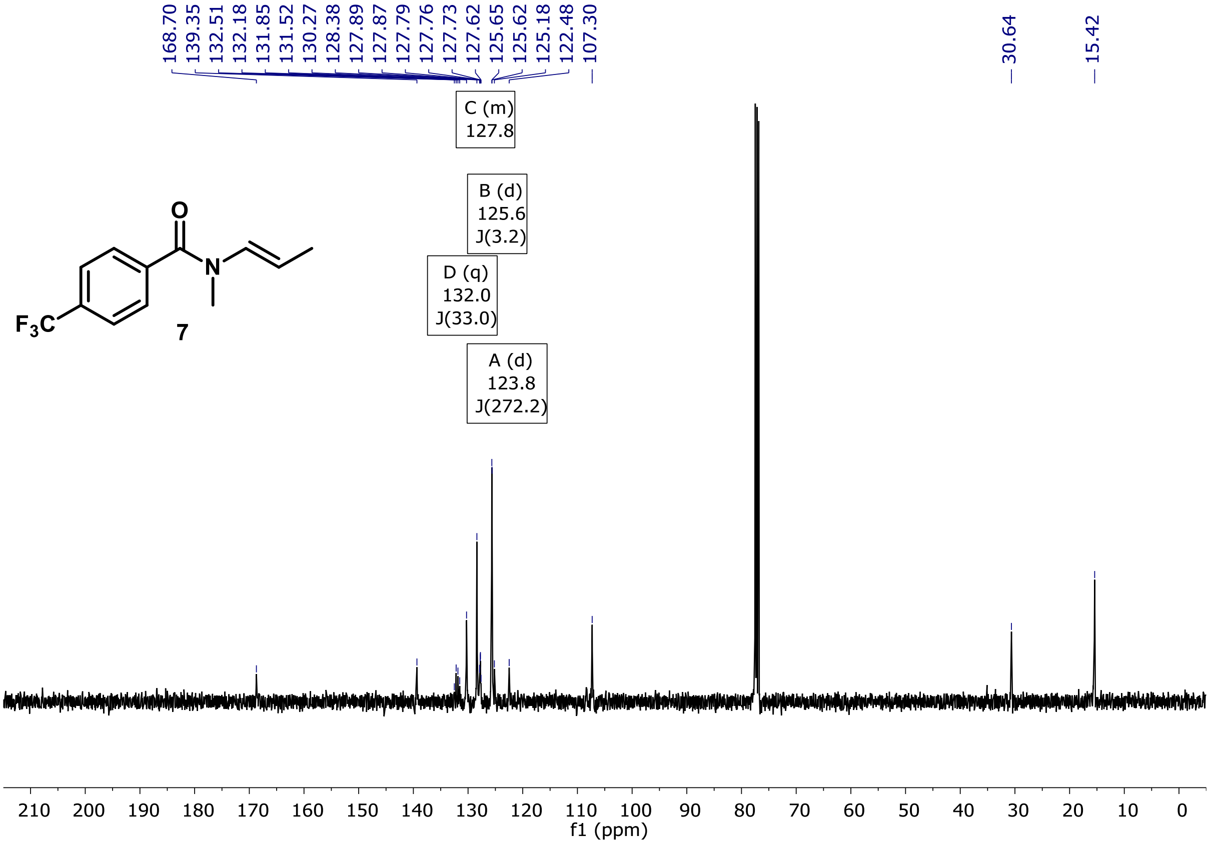

^{19}F (376 MHz, $CDCl_3$)

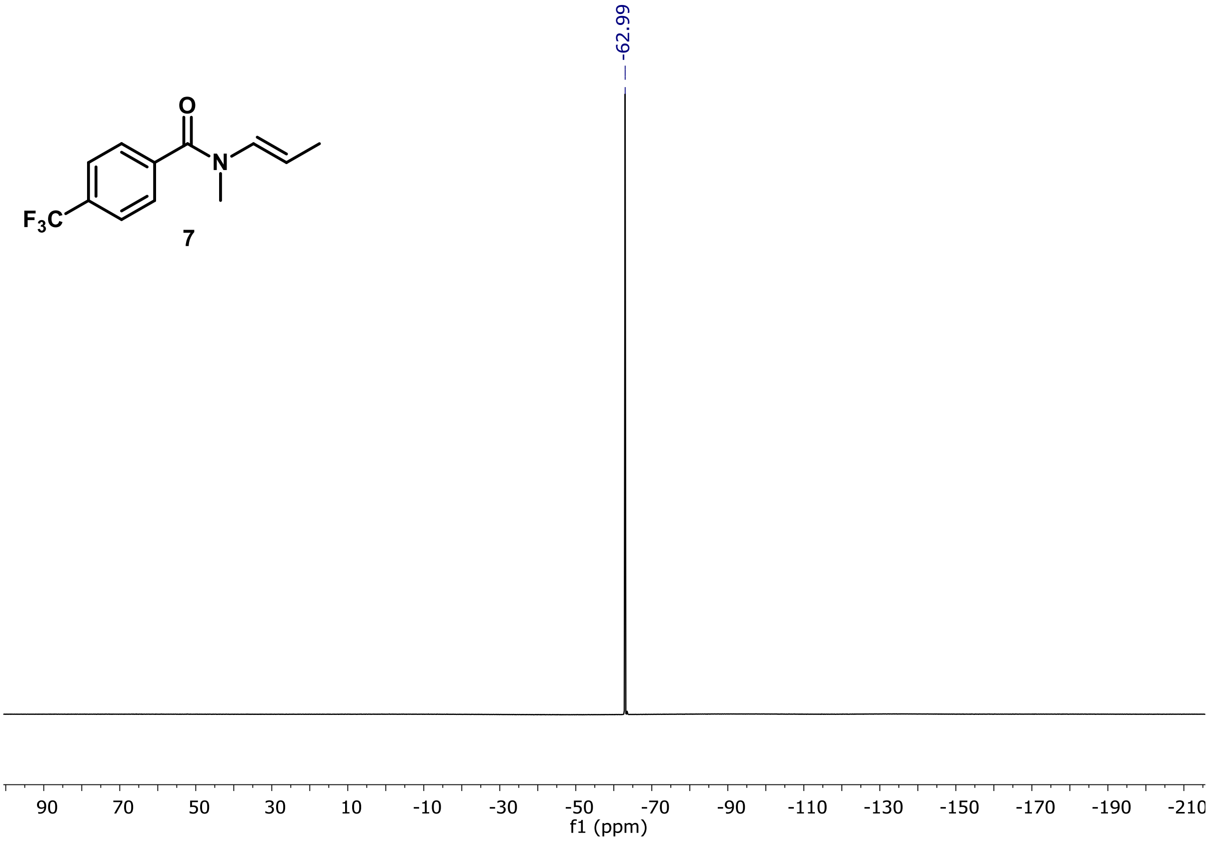

^{1}H (400 MHz, $CDCl_3$)

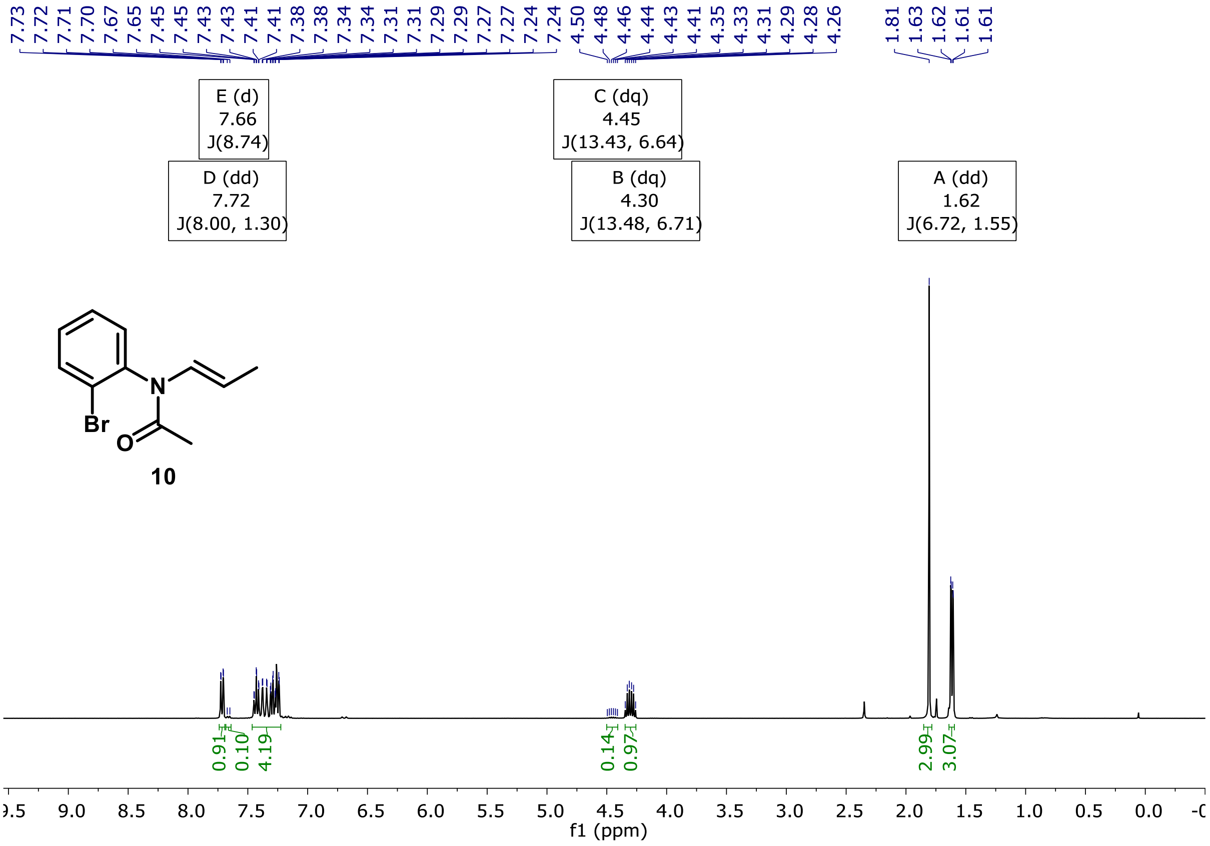

^{13}C (101 MHz, $CDCl_3$)

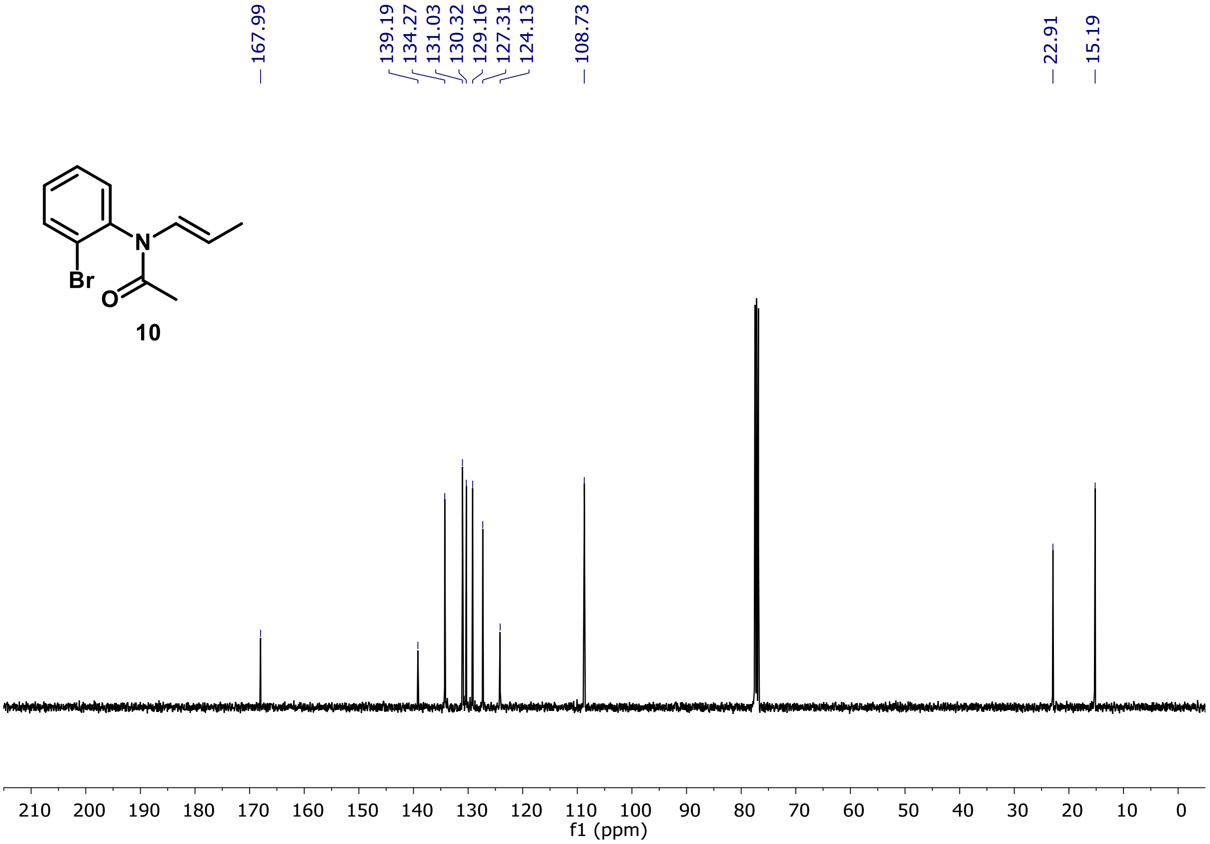

^{1}H (400 MHz, $CDCl_3$)

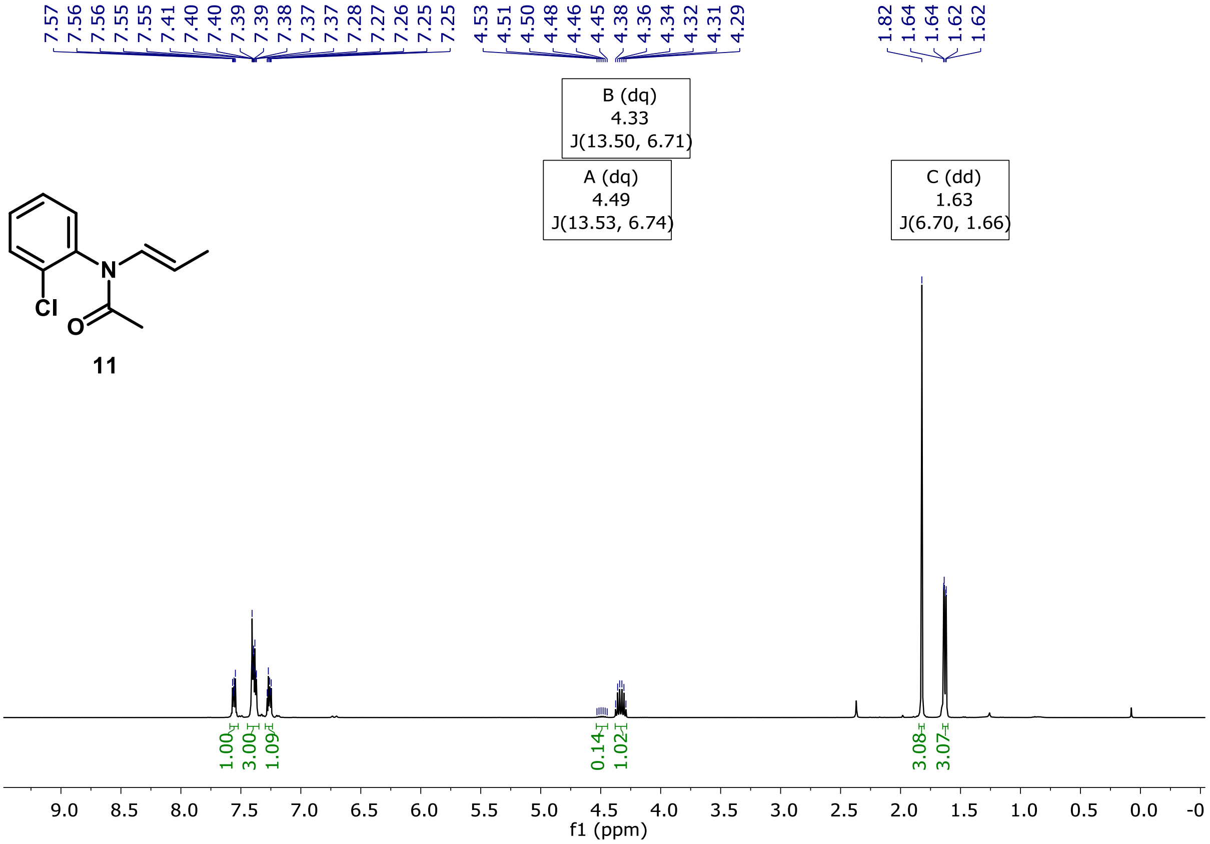

^{13}C (101 MHz, $CDCl_3$)

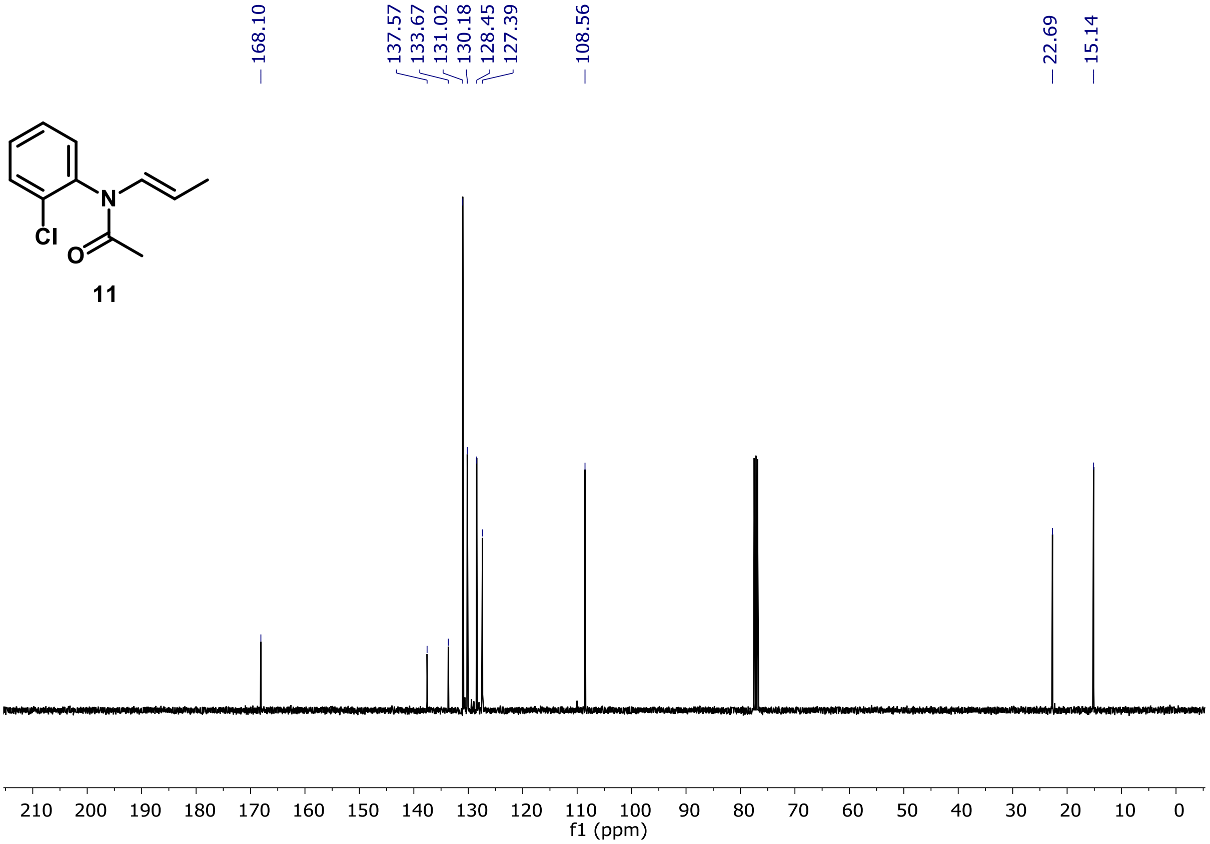

^{1}H (400 MHz, $CDCl_3$)

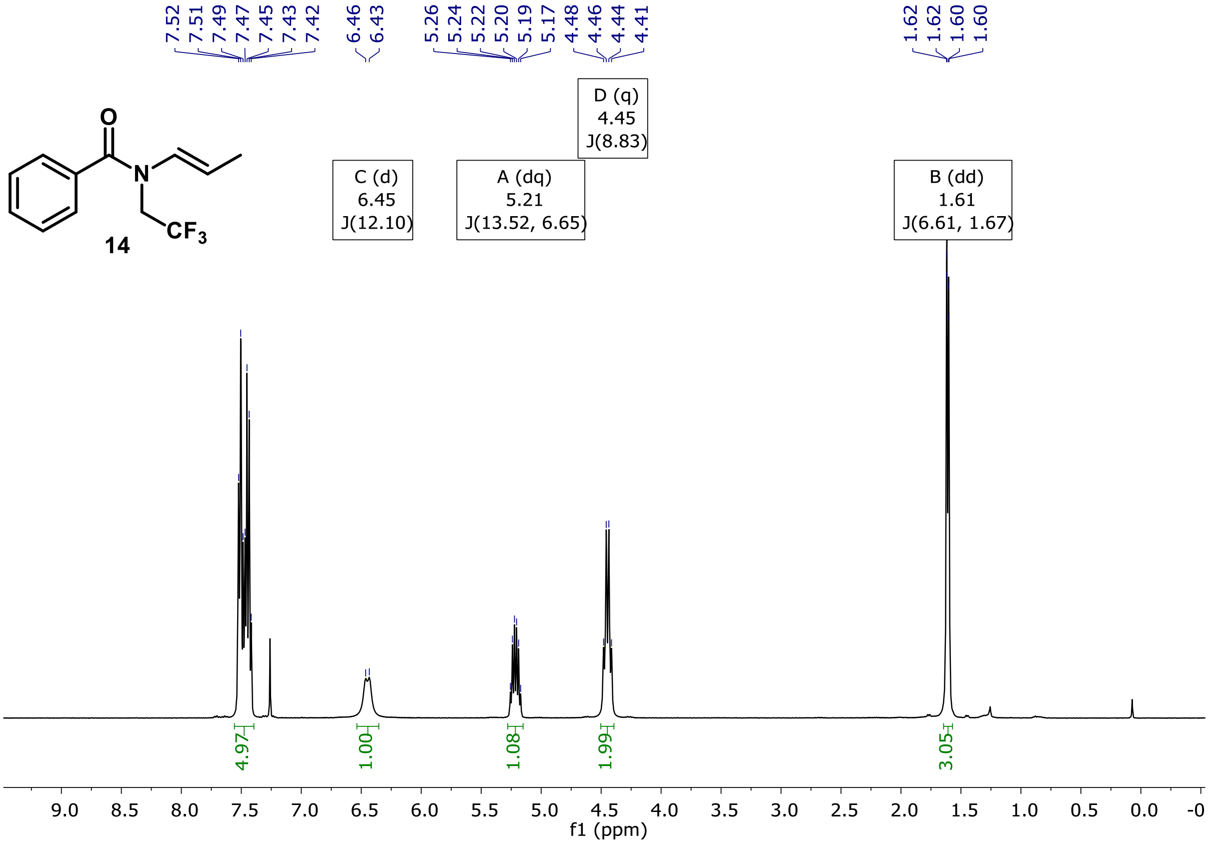

^{13}C (101 MHz, $CDCl_3$)

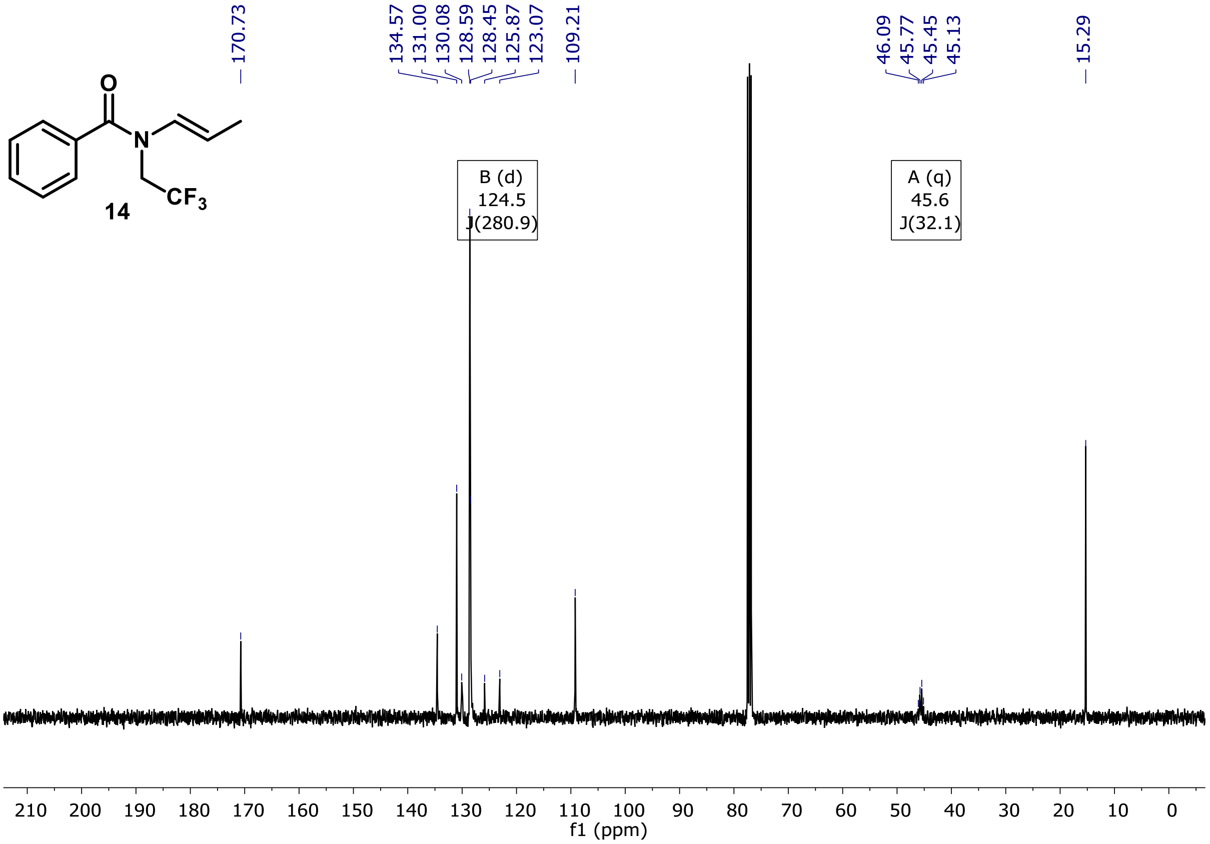

^{19}F (376 MHz, $CDCl_3$)

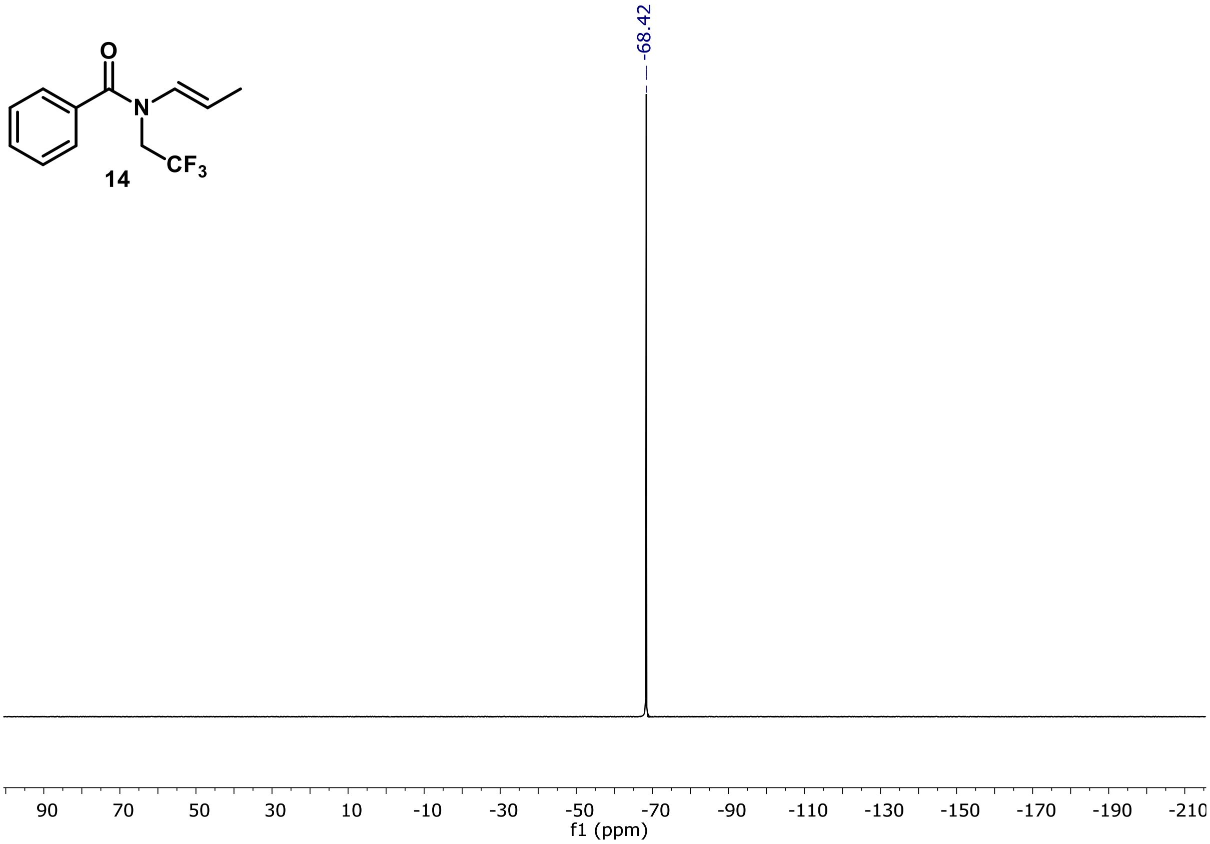

^{1}H (400 MHz, $CDCl_3$)

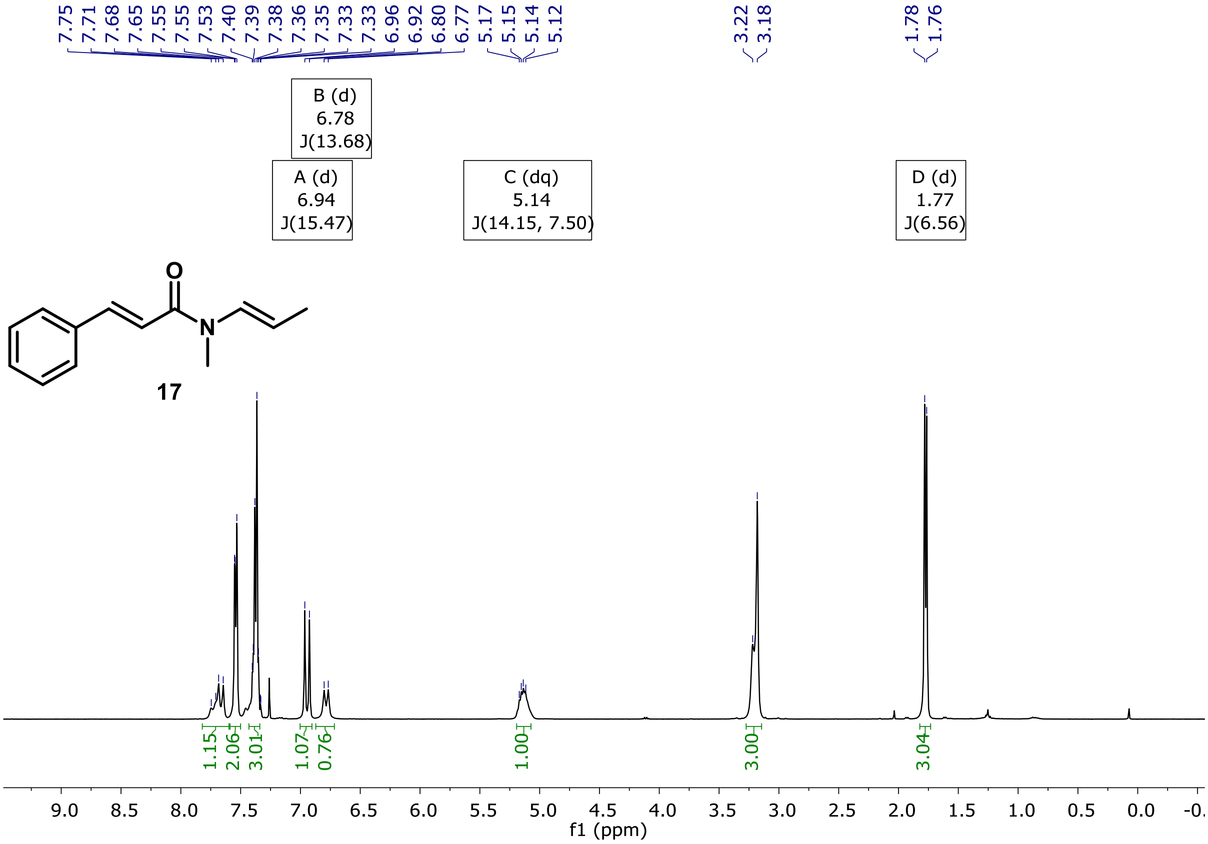

^{13}C (101 MHz, $CDCl_3$)

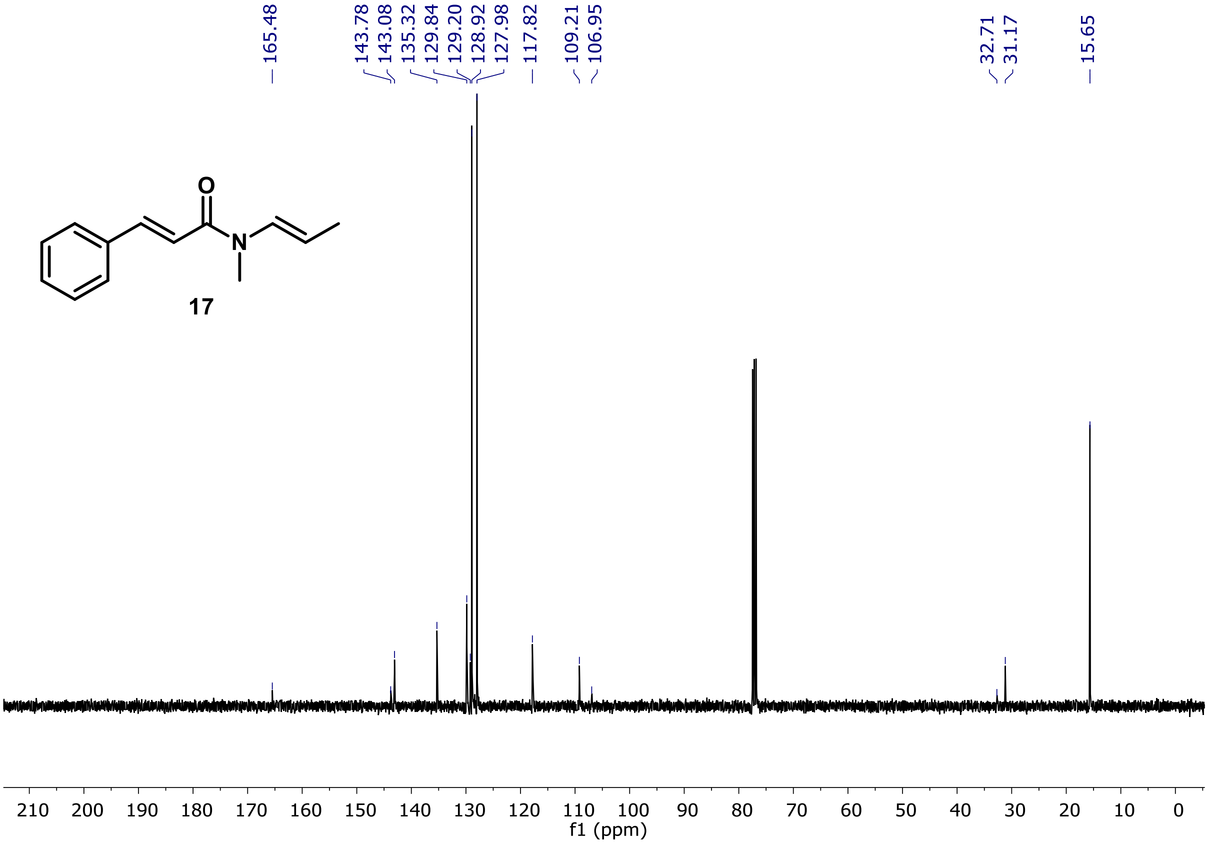

^1H (400 MHz, $CDCl_3$)

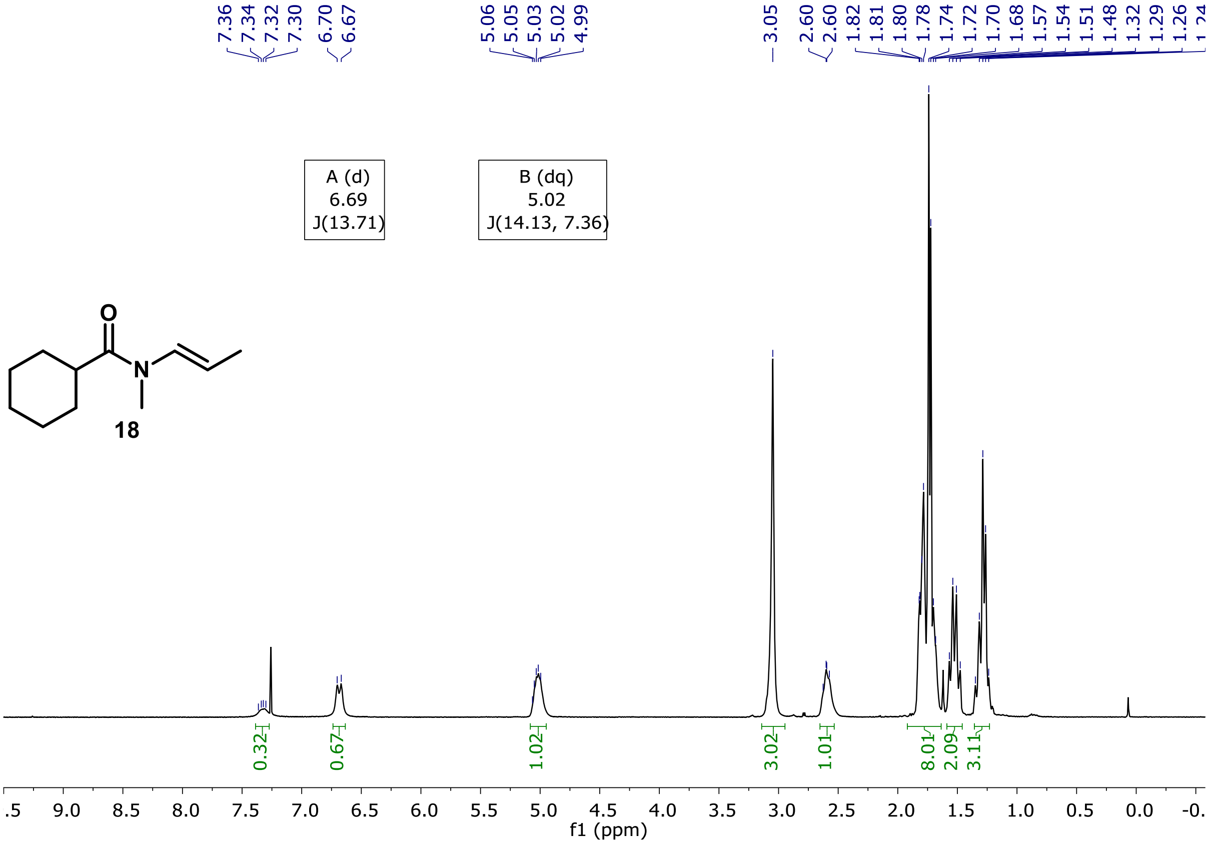

^{13}C (101 MHz, $CDCl_3$)

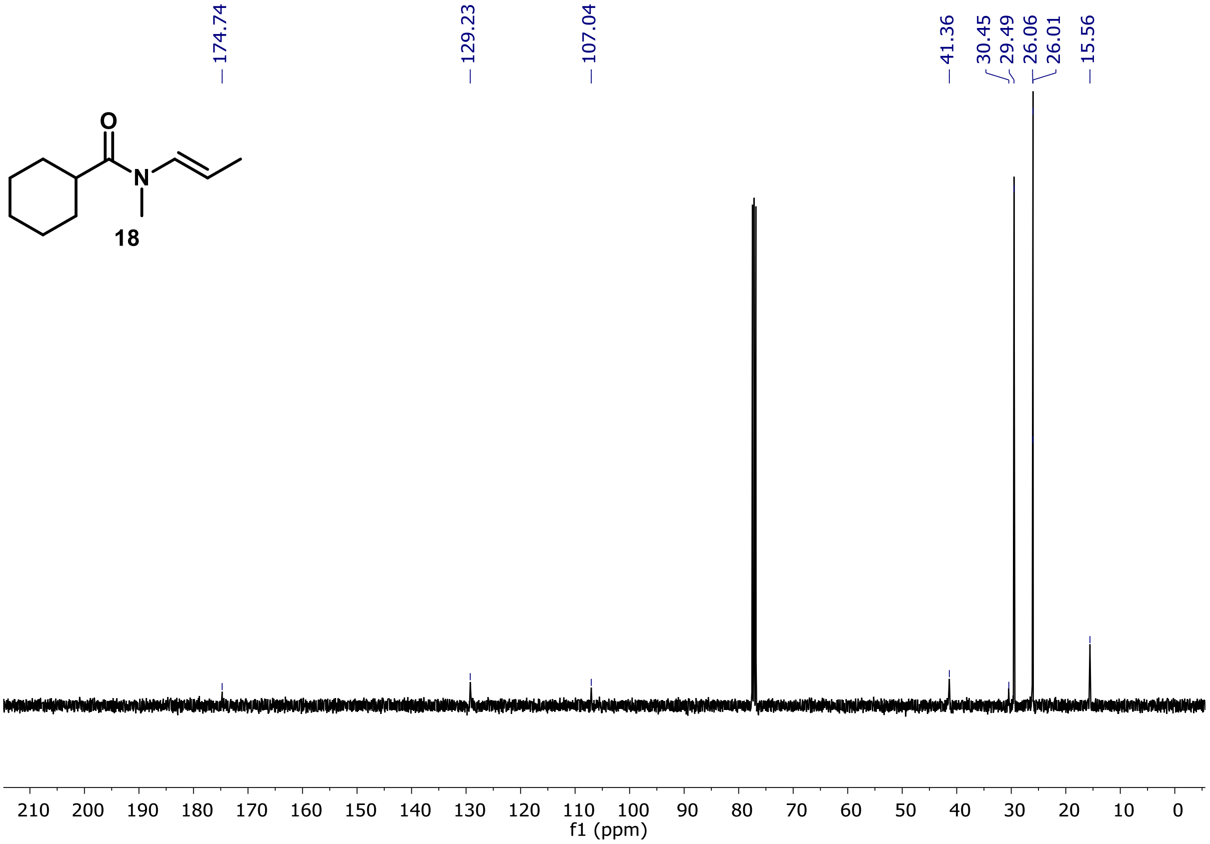

^{1}H (600 MHz, $CDCl_3$)

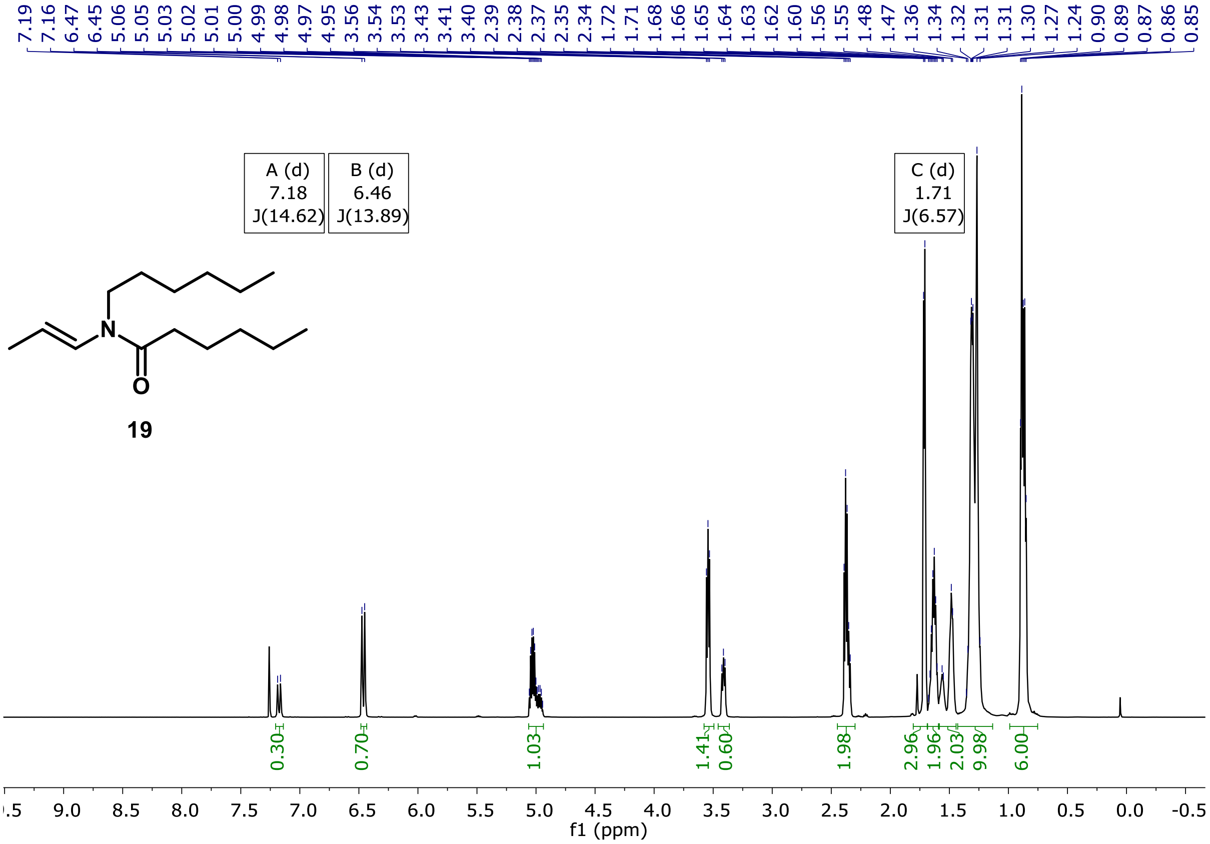

^{13}C (151 MHz, $CDCl_3$)

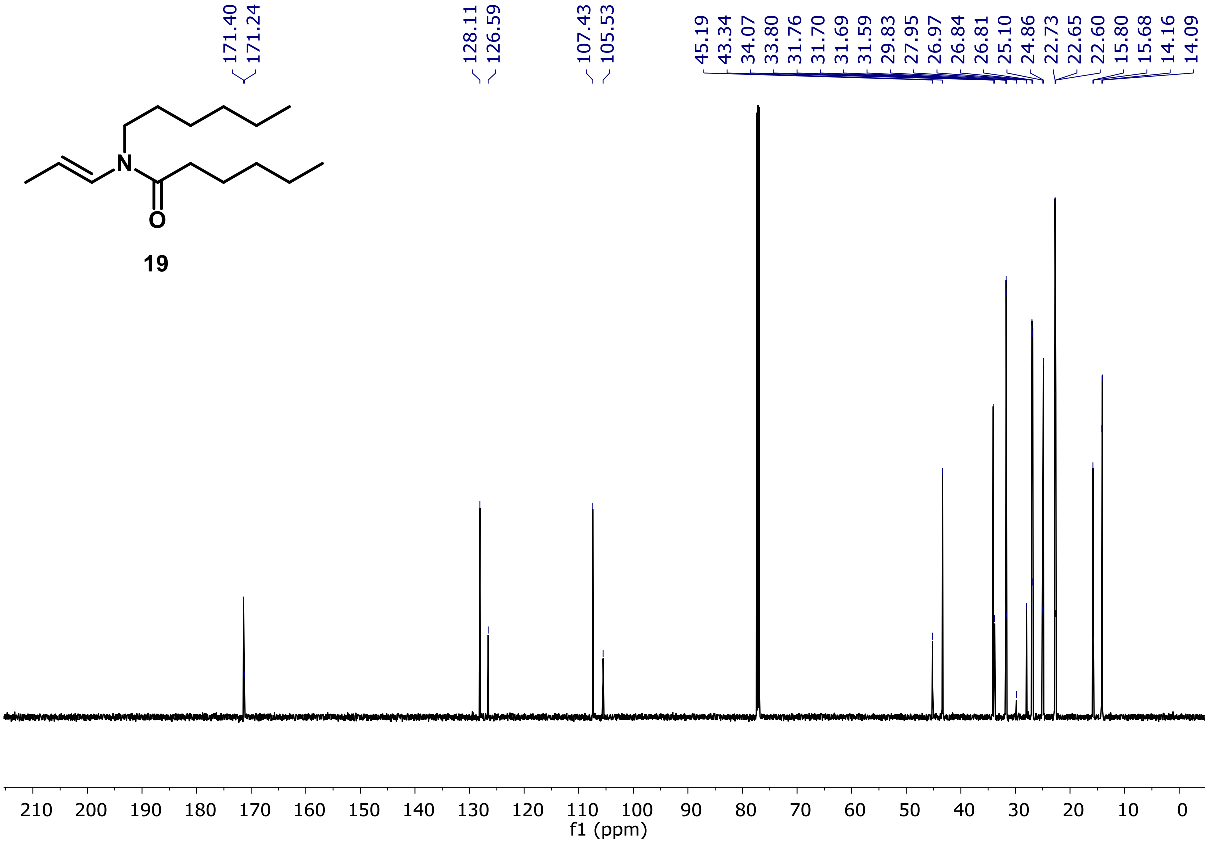

^{1}H (400 MHz, $CDCl_3$)

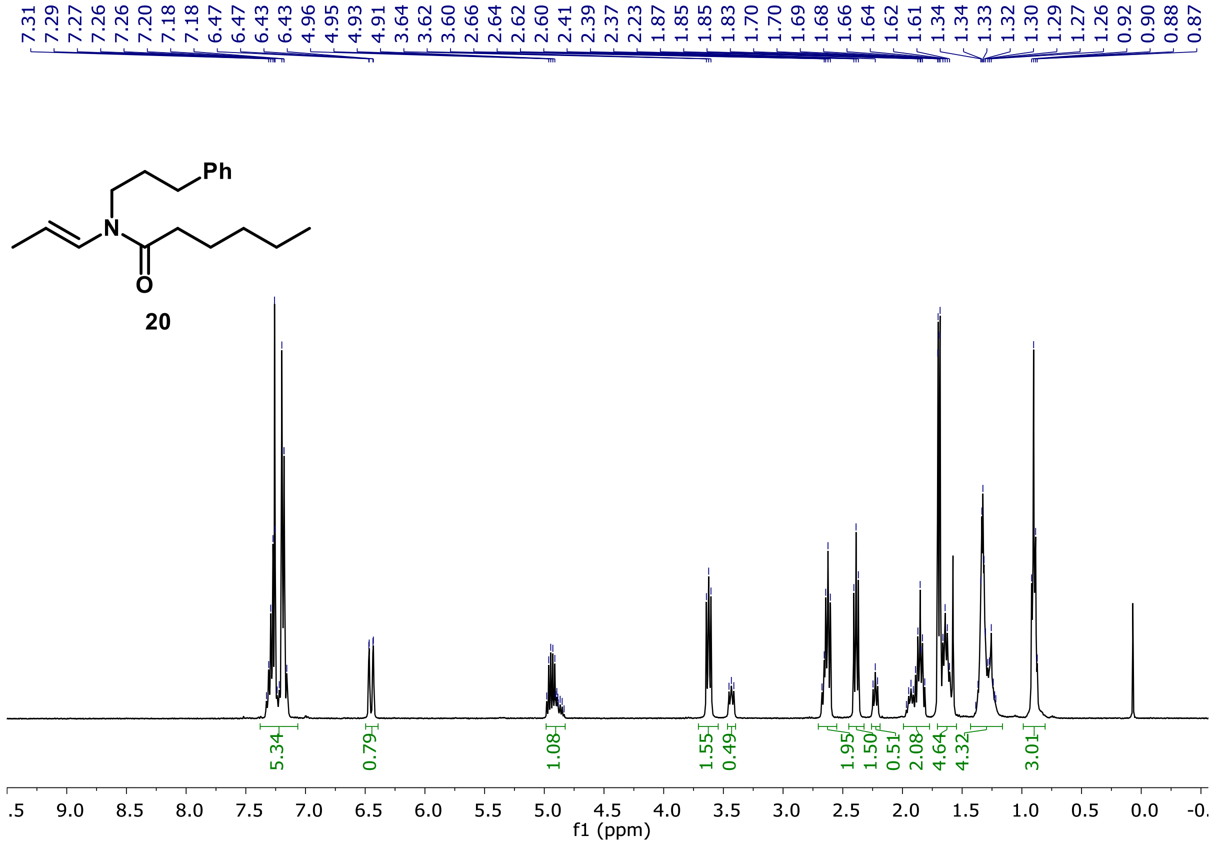

^{13}C (101 MHz, $CDCl_3$)

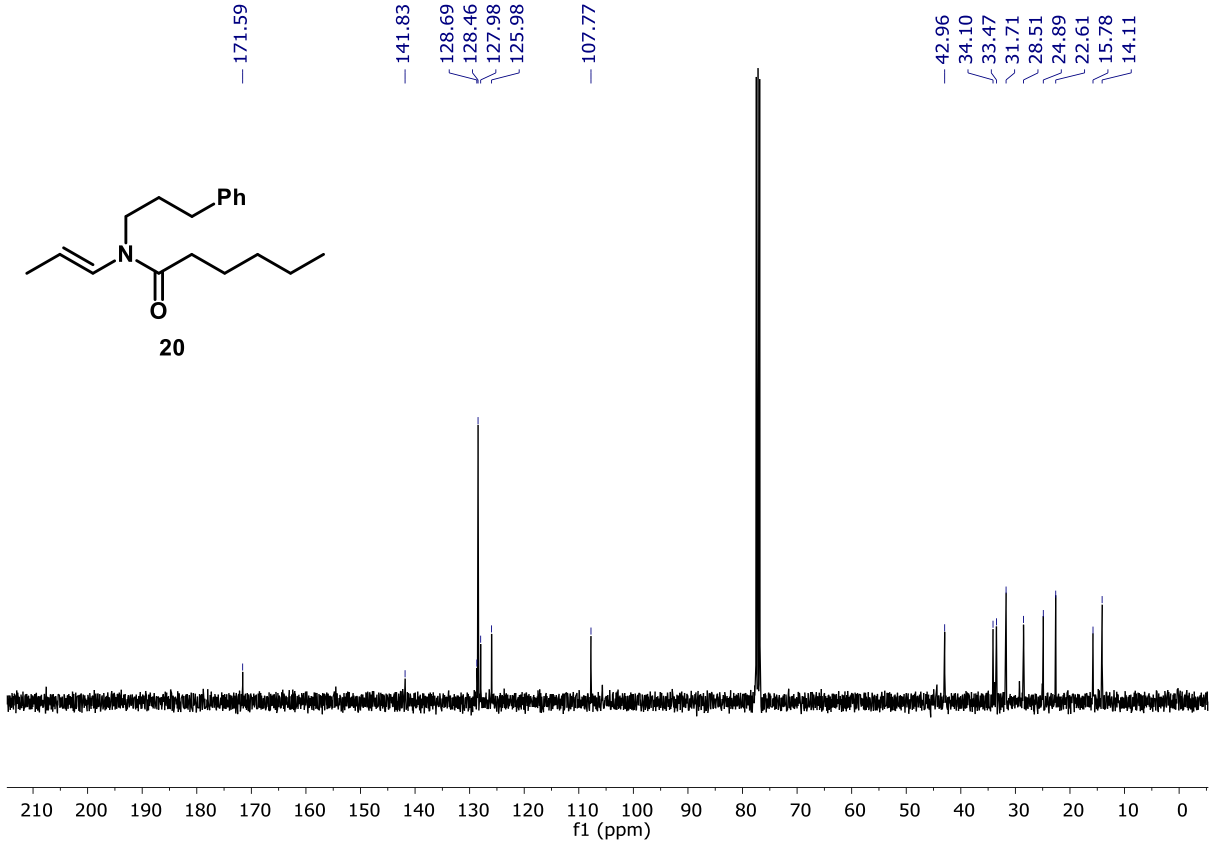

^{1}H (600 MHz, CDCl₃)

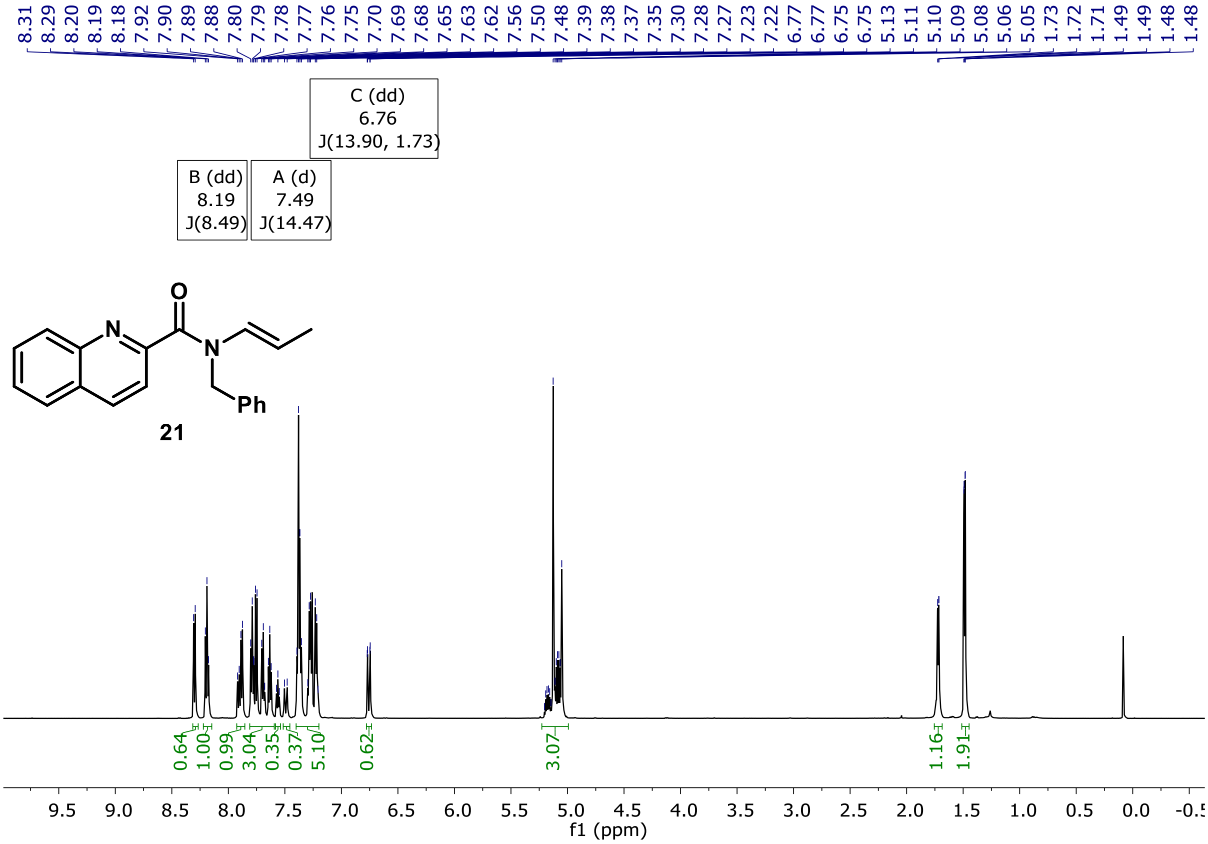

^{13}C (151 MHz, CDCl₃)

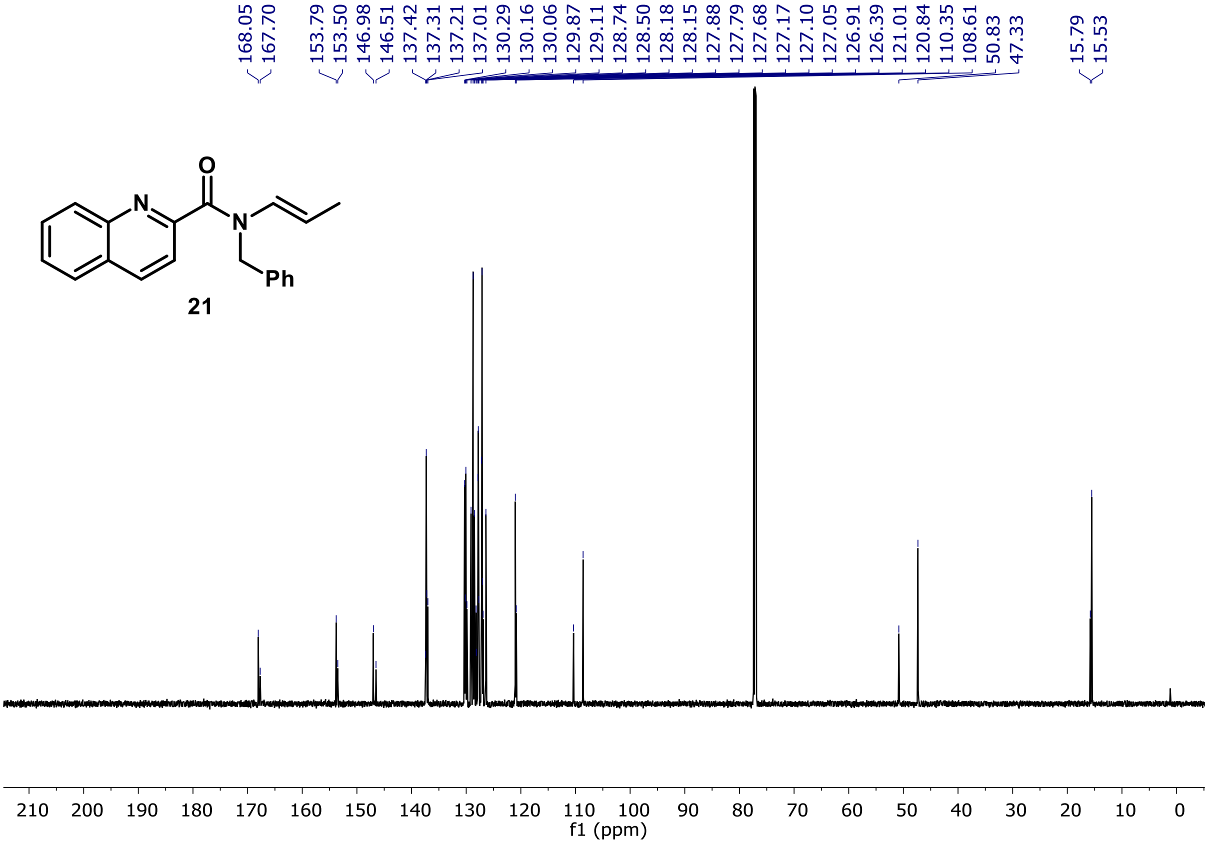

^{1}H (400 MHz, $CDCl_3$)

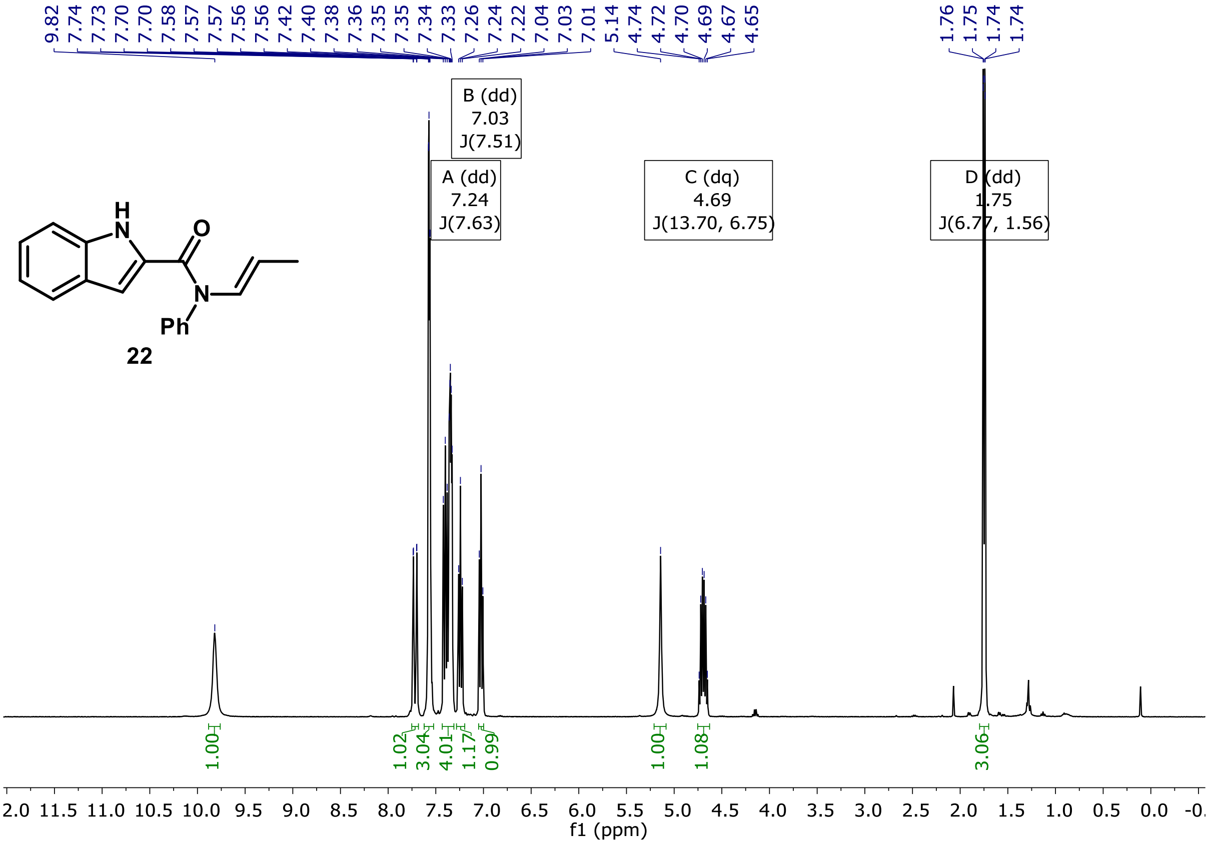

^{13}C (101 MHz, $CDCl_3$)

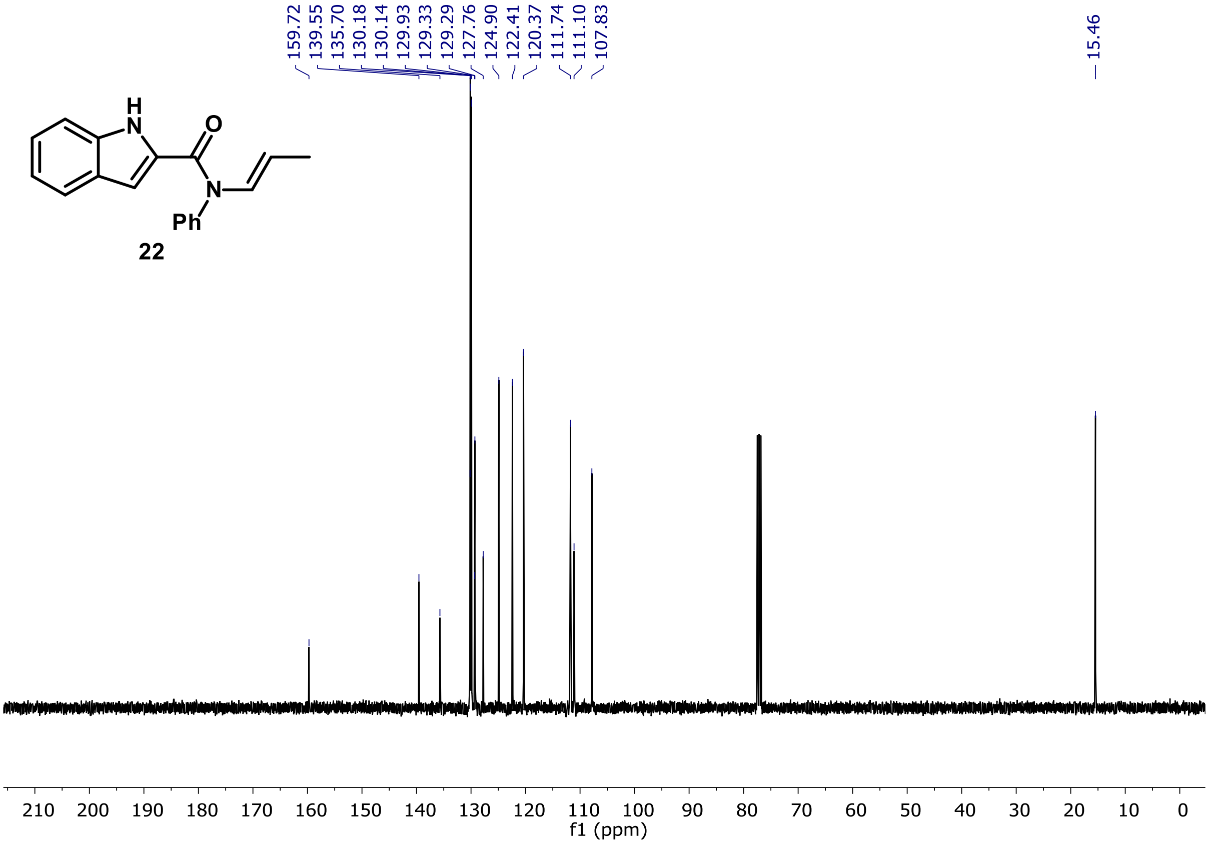

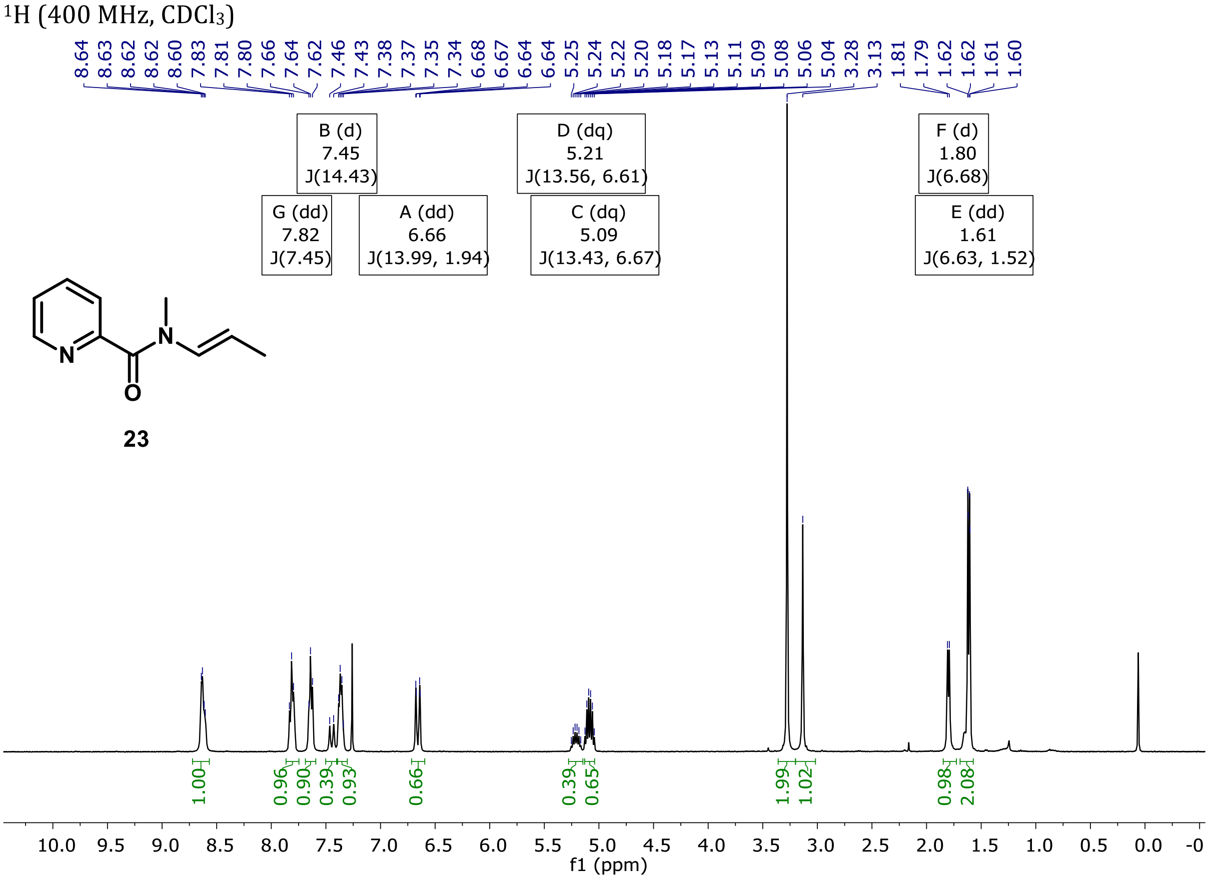

1H (400 MHz, CDCl3)
8.64
8.63
8.62
8.62
8.60
7.83
7.81
7.80
7.66
7.64
7.62
7.46
7.43
7.38
7.37
7.35
7.34
6.68
6.67
6.64
6.64
5.25
5.24
5.22
5.20
5.18
5.17
5.13
5.11
5.09
5.08
5.06
5.04
3.28
3.13
1.81
1.79
1.62
1.62
1.61
1.60
B (d) 7.45 J(14.43)
G (dd) 7.82 J(7.45)
A (dd) 6.66 J(13.99, 1.94)
D (dq) 5.21 J(13.56, 6.61)
C (dq) 5.09 J(13.43, 6.67)
F (d) 1.80 J(6.68)
E (dd) 1.61 J(6.63, 1.52)
N
O
23
1.00
0.96
0.90
0.39
0.93
0.66
0.39
0.65
1.99
1.02
0.98
2.08
10.0 9.5 9.0 8.5 8.0 7.5 7.0 6.5 6.0 5.5 5.0 4.5 4.0 3.5 3.0 2.5 2.0 1.5 1.0 0.5 0.0 -0
f1 (ppm)

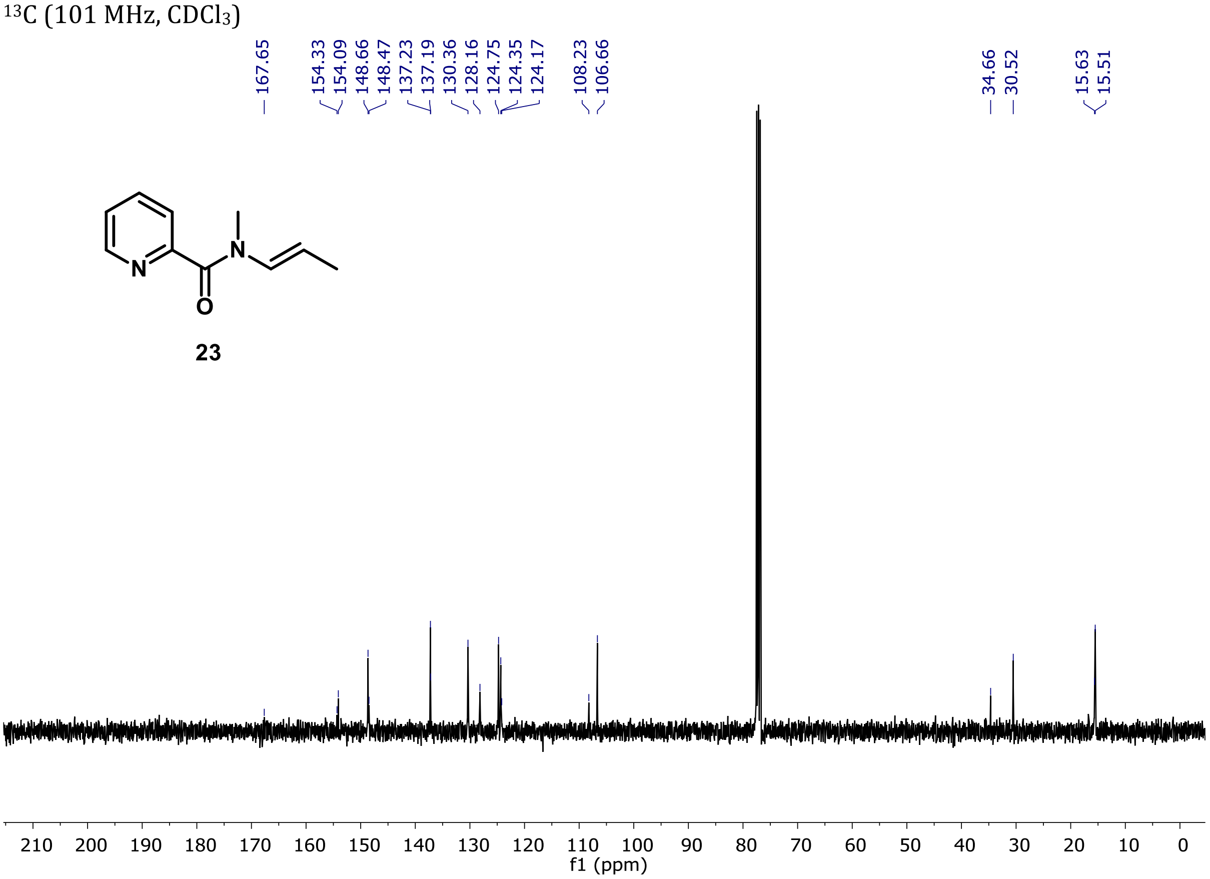

13C (101 MHz, CDCl3)
167.65
154.33
154.09
148.66
148.47
137.23
137.19
130.36
128.16
124.75
124.35
124.17
108.23
106.66
34.66
30.52
15.63
15.51
N
O
23
210 200 190 180 170 160 150 140 130 120 110 100 90 80 70 60 50 40 30 20 10 0
f1 (ppm)

^{1}H (400 MHz, $CDCl_3$)

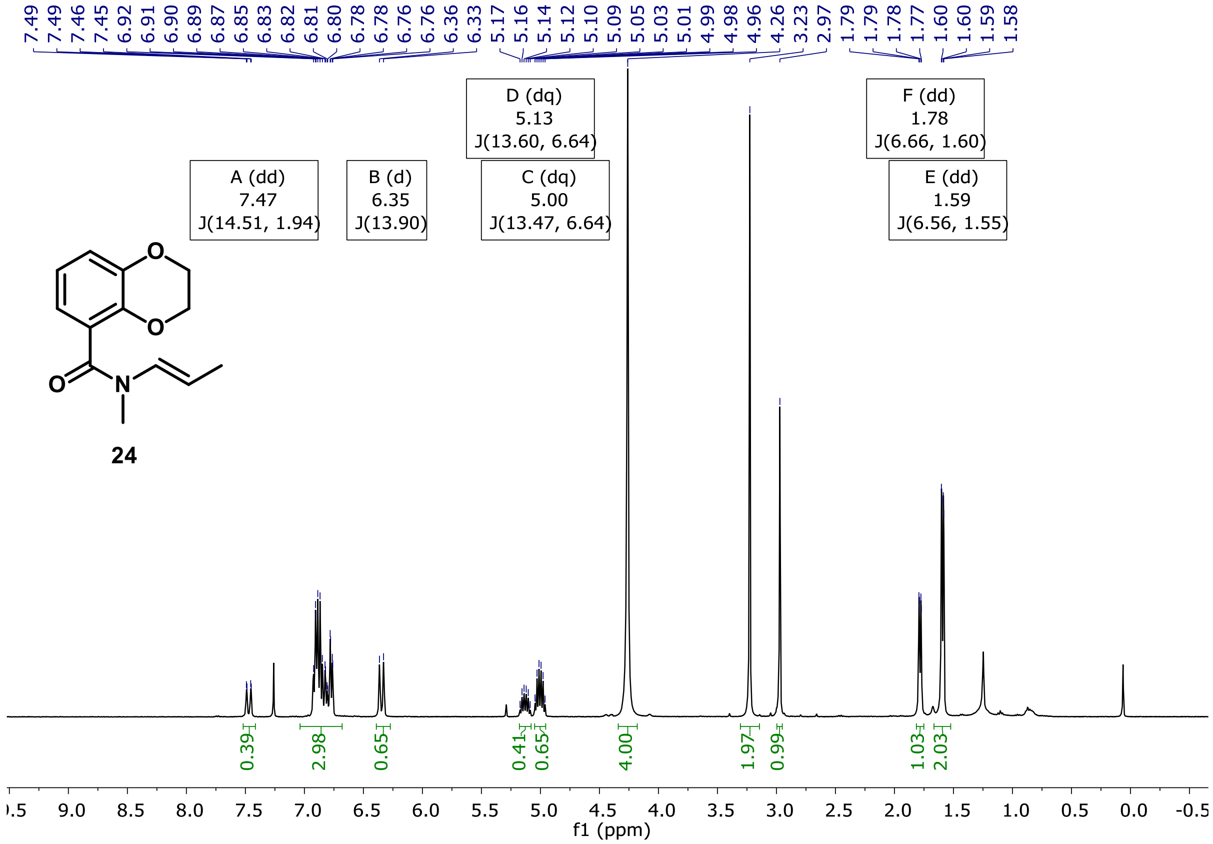

^{13}C (101 MHz, $CDCl_3$)

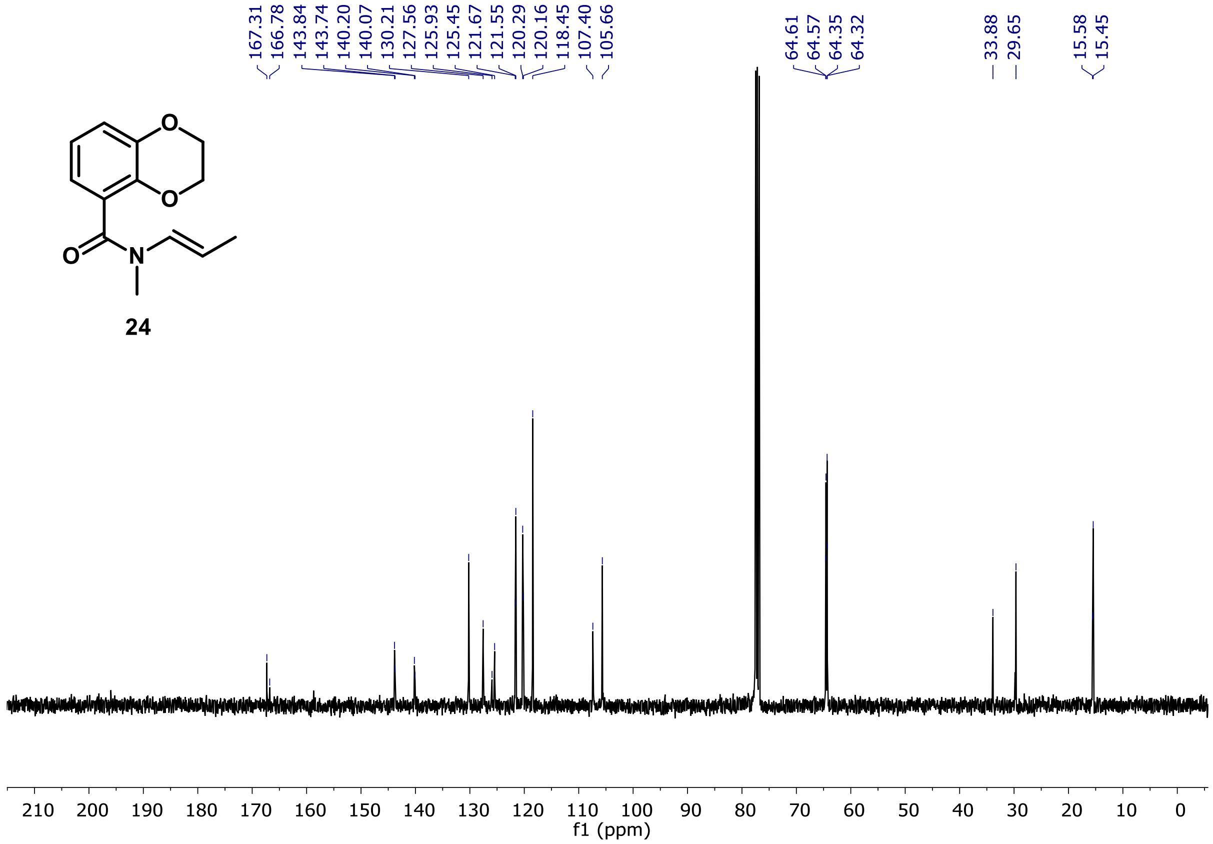

^{1}H (400 MHz, $CDCl_3$)

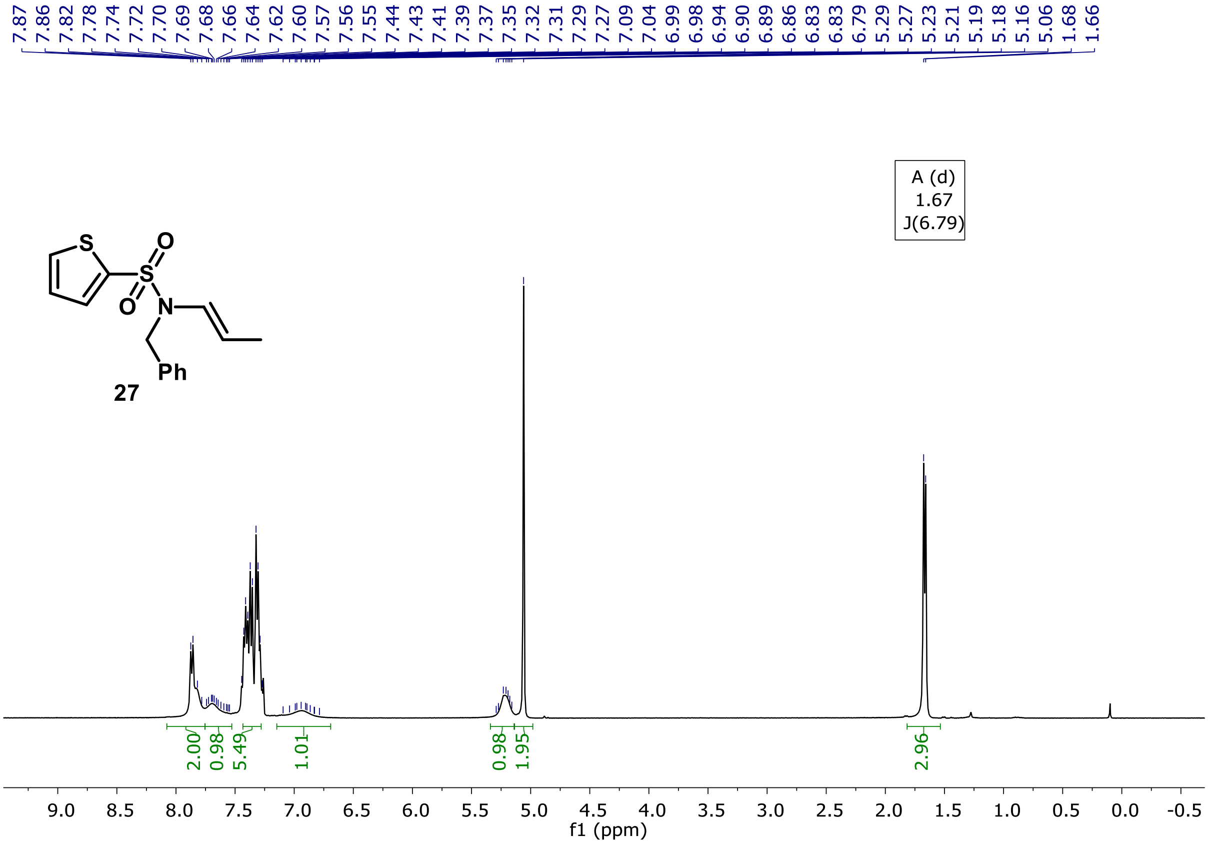

^{13}C (101 MHz, $CDCl_3$)

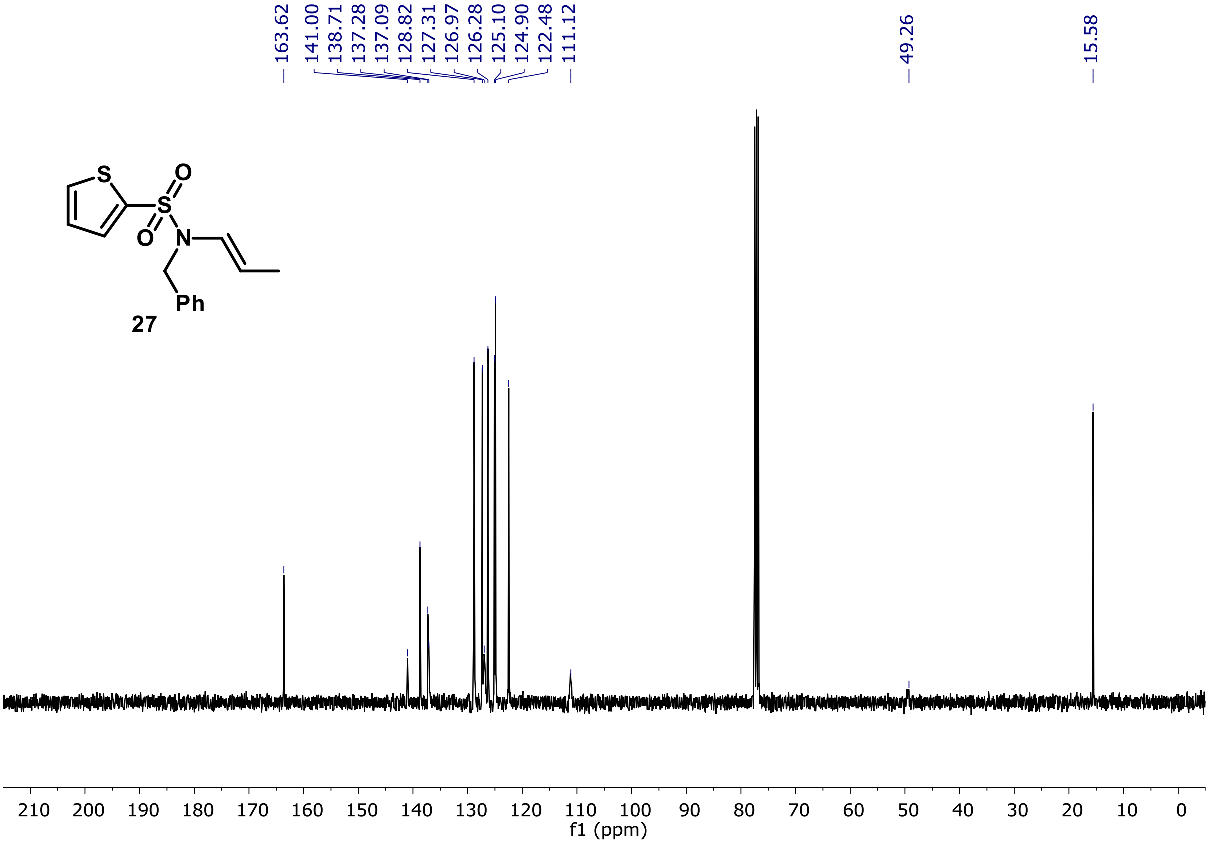

^{1}H (400 MHz, CDCl$_3$)

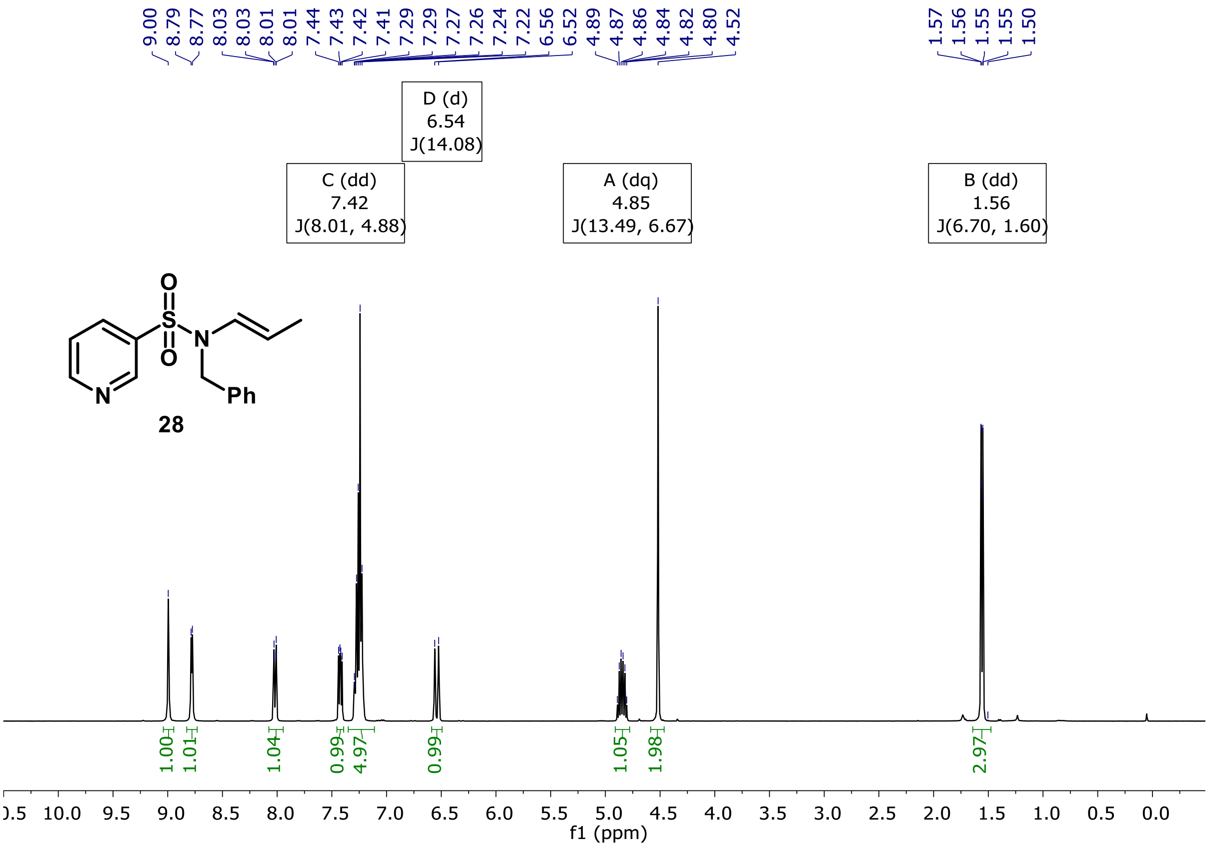

^{13}C (101 MHz, CDCl$_3$)

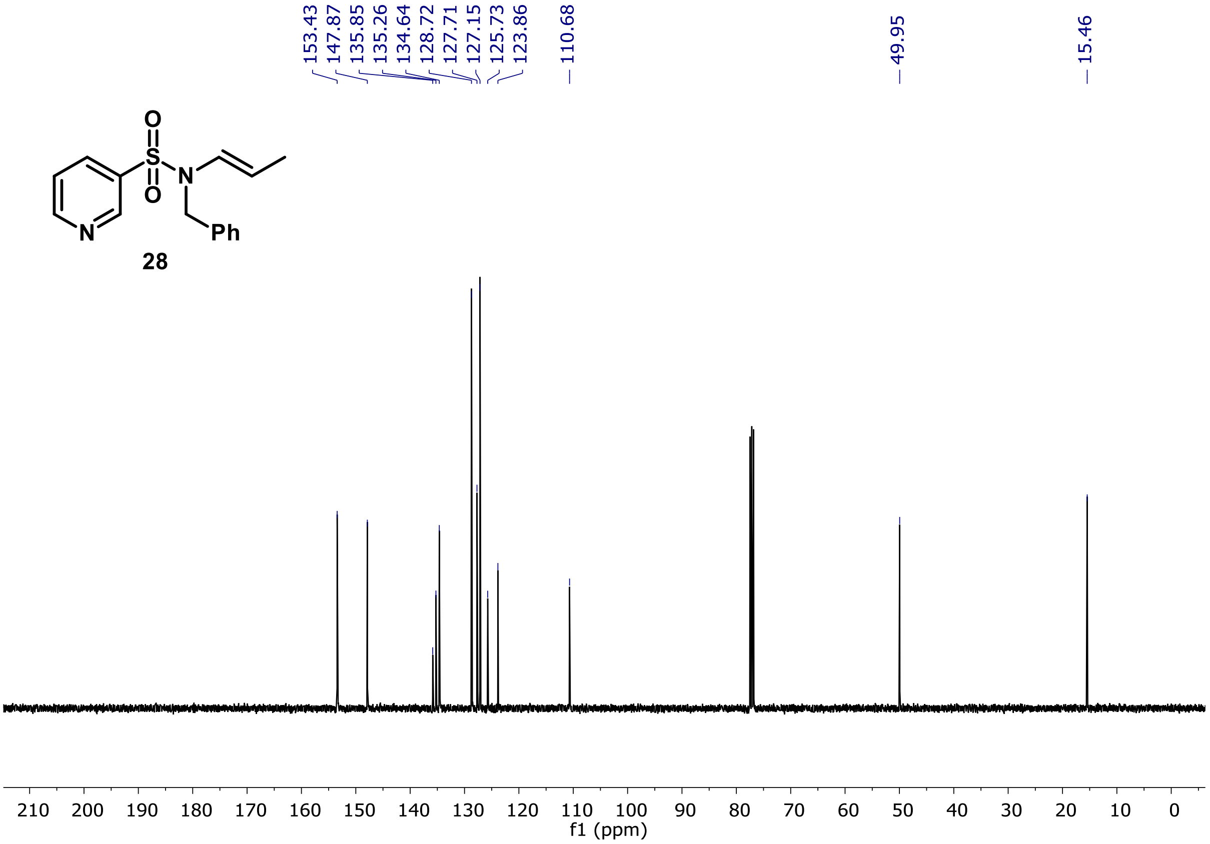

^{1}H (400 MHz, $CDCl_3$)

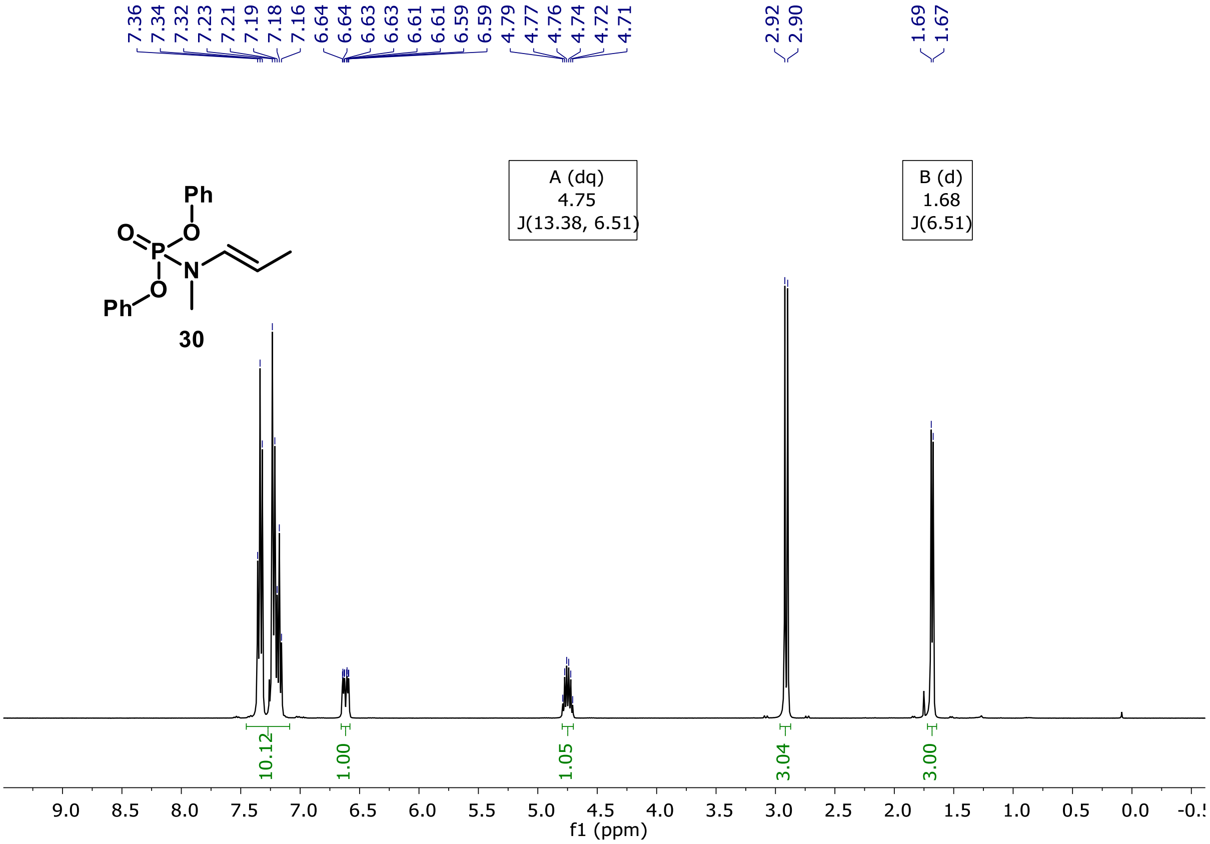

^{13}C (101 MHz, $CDCl_3$)

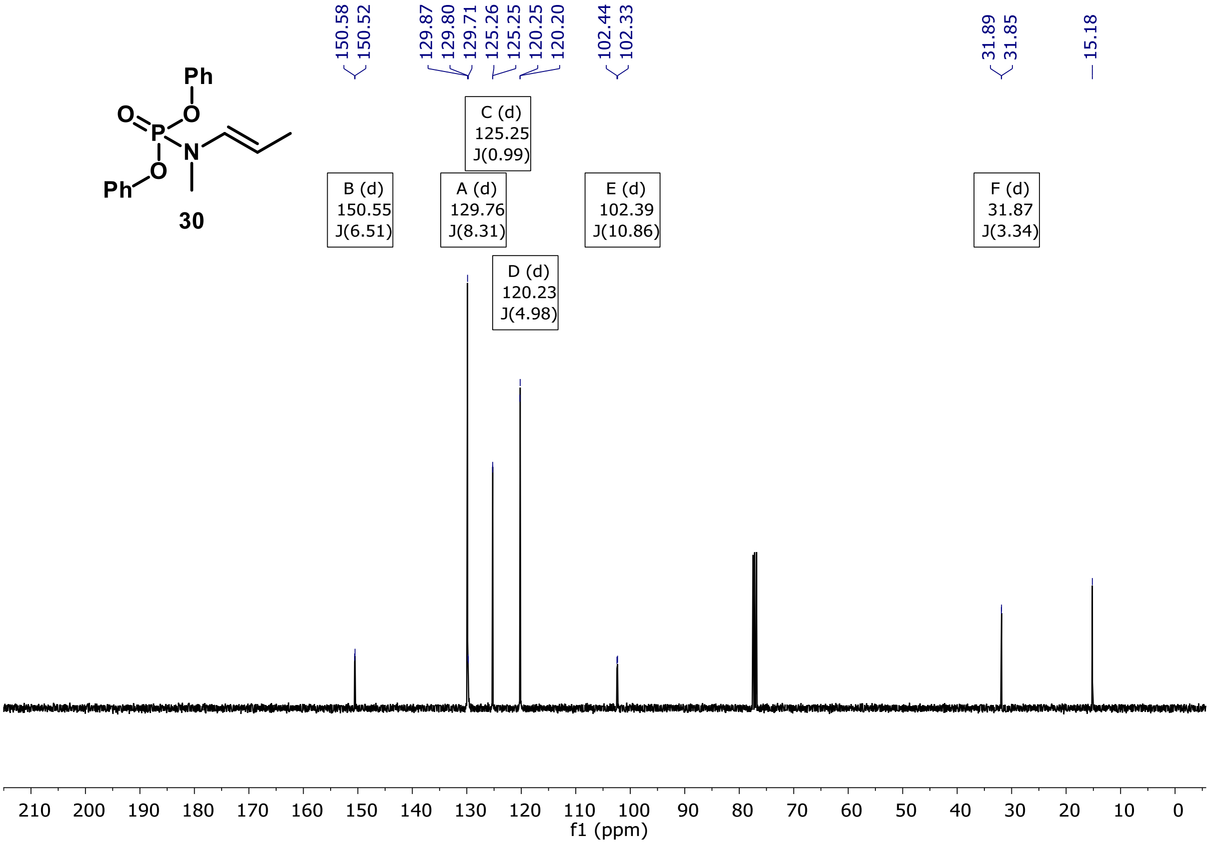

^{1}H (400 MHz, $CDCl_3$)

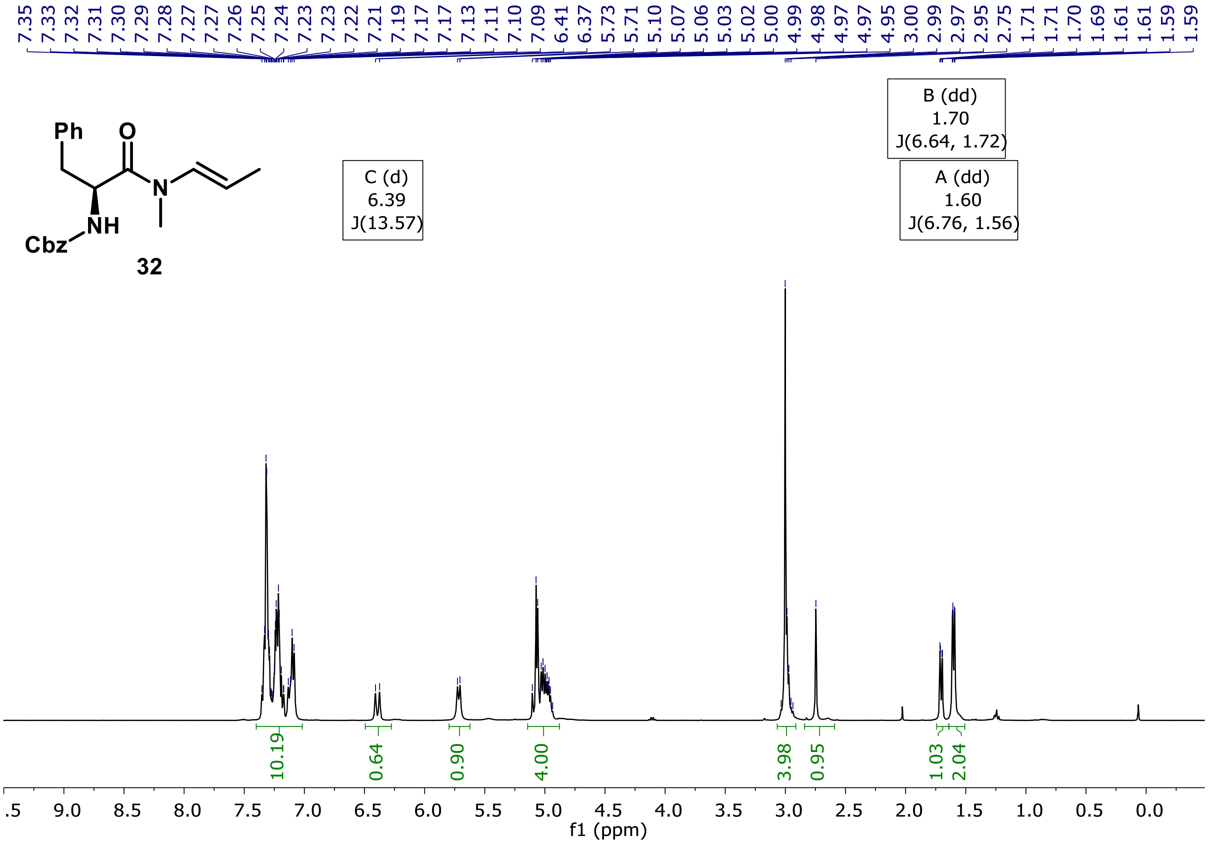

^{13}C (400 MHz, $CDCl_3$)

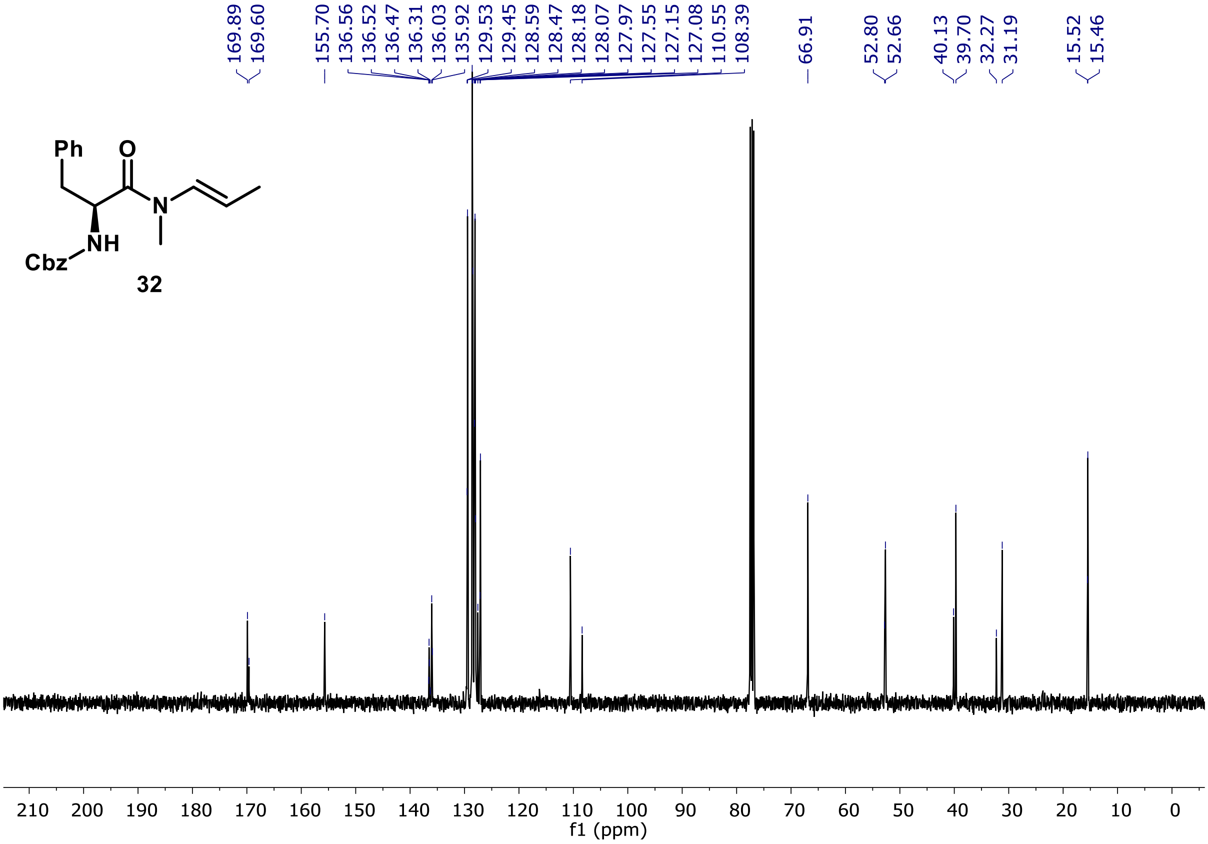

^{1}H (400 MHz, $CDCl_3$)

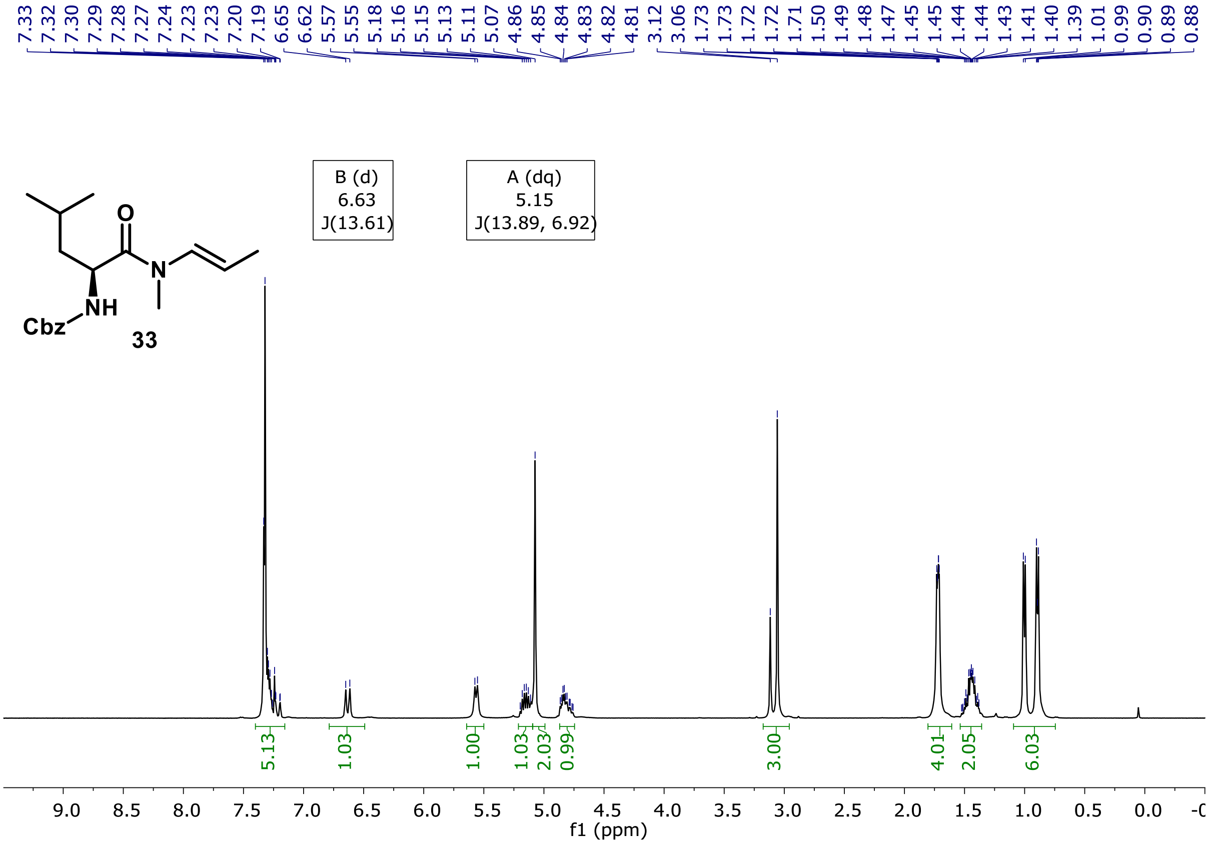

^{13}C (101 MHz, $CDCl_3$)

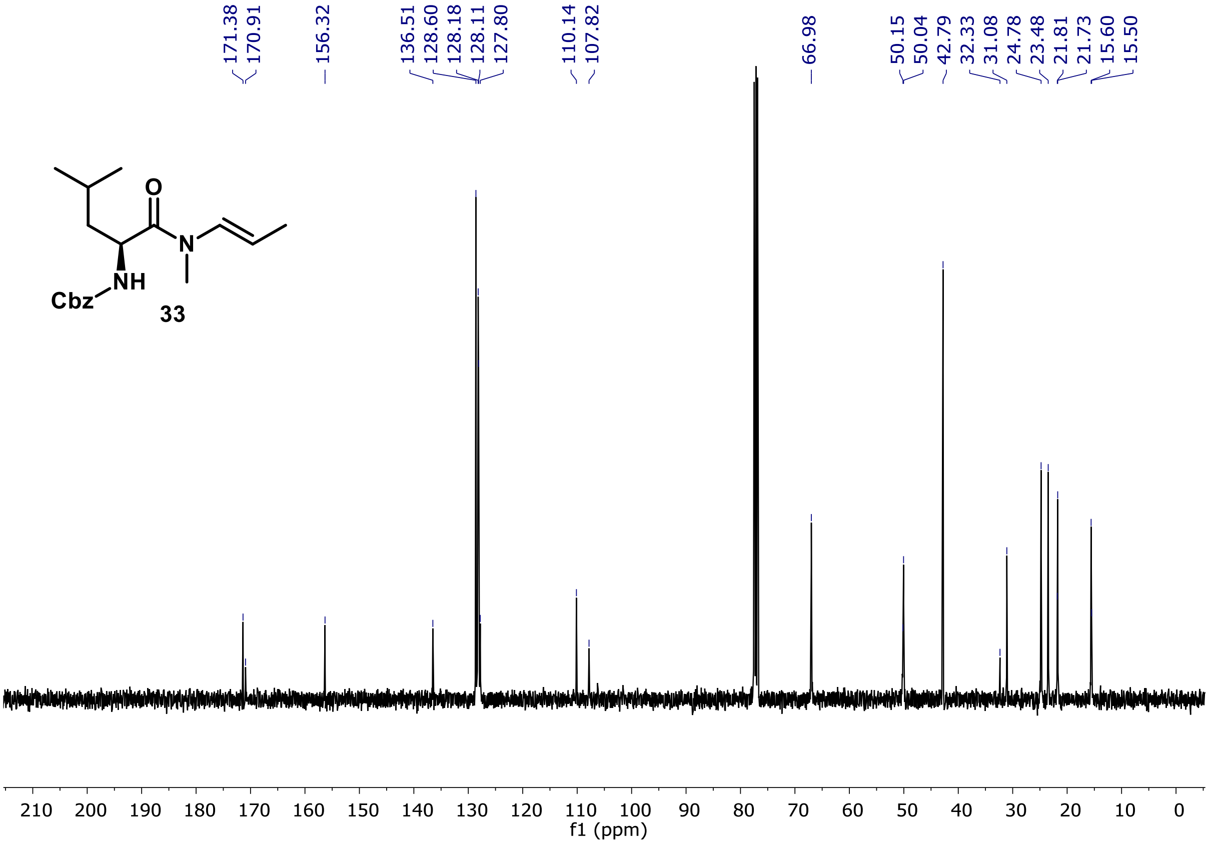

^{1}H (400 MHz, $CDCl_3$)

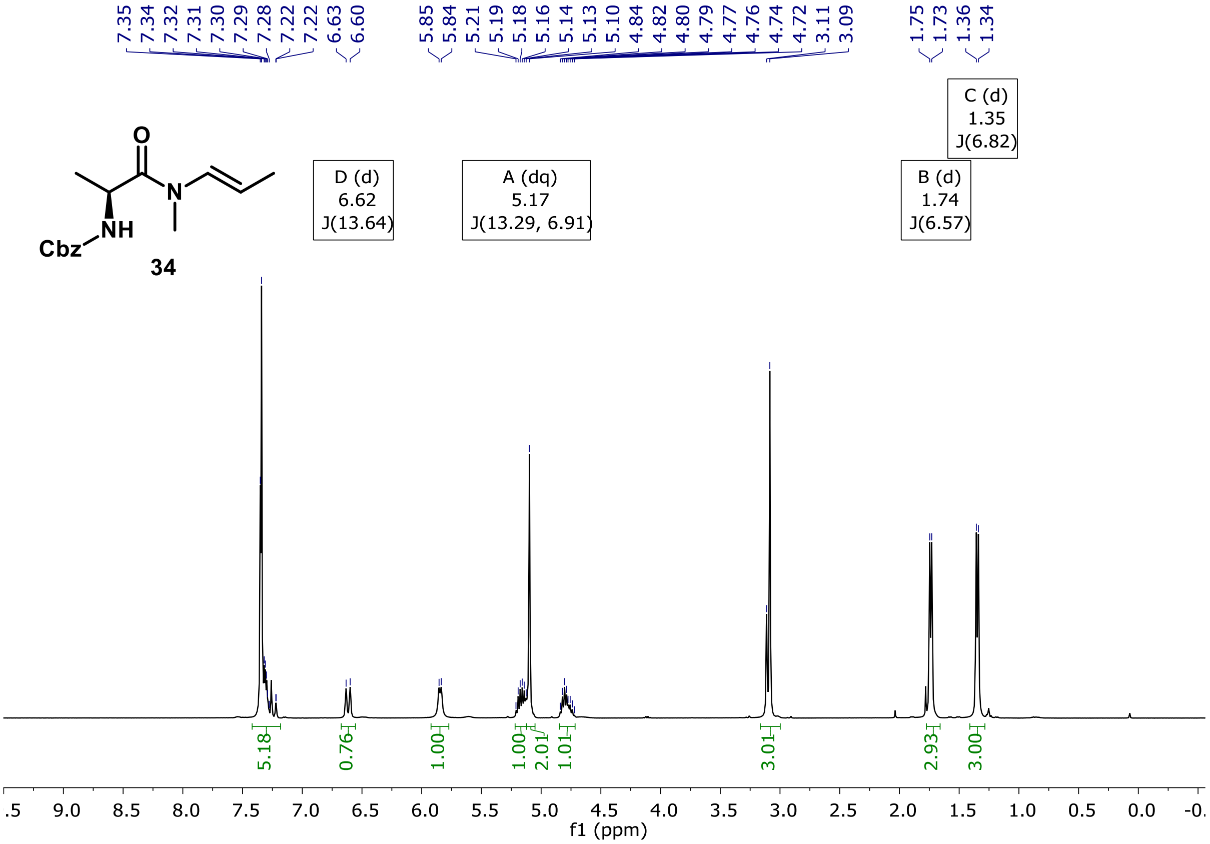

^{13}C (101 MHz, $CDCl_3$)

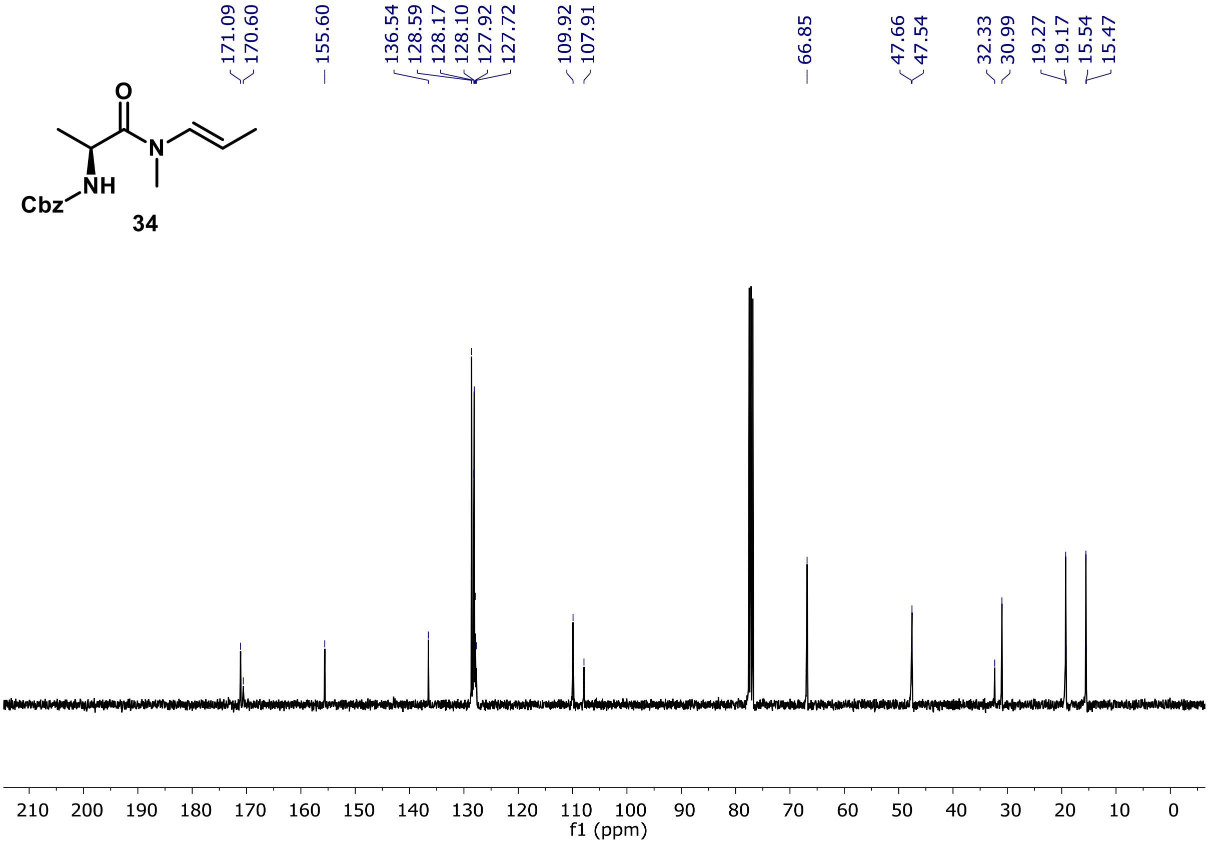

^{1}H (400 MHz, $CDCl_3$)

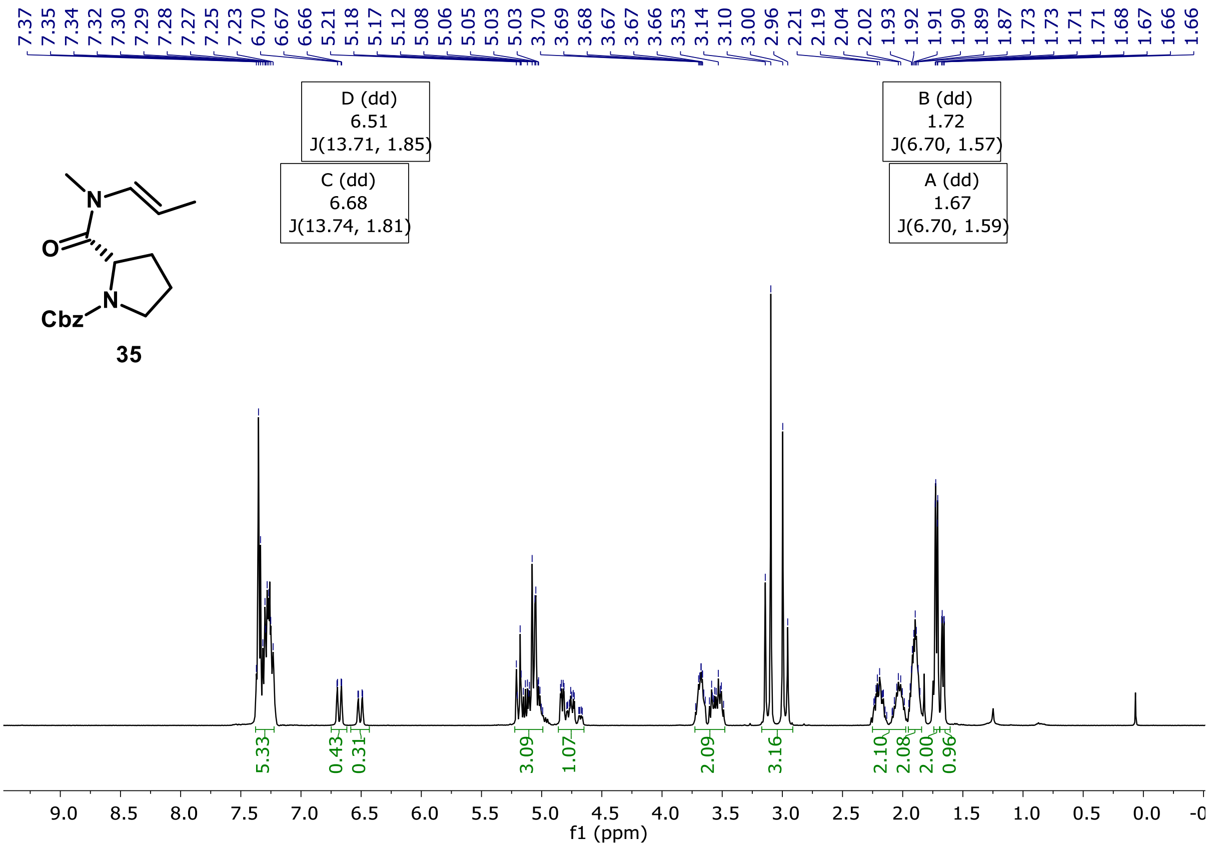

^{13}C (101 MHz, $CDCl_3$)

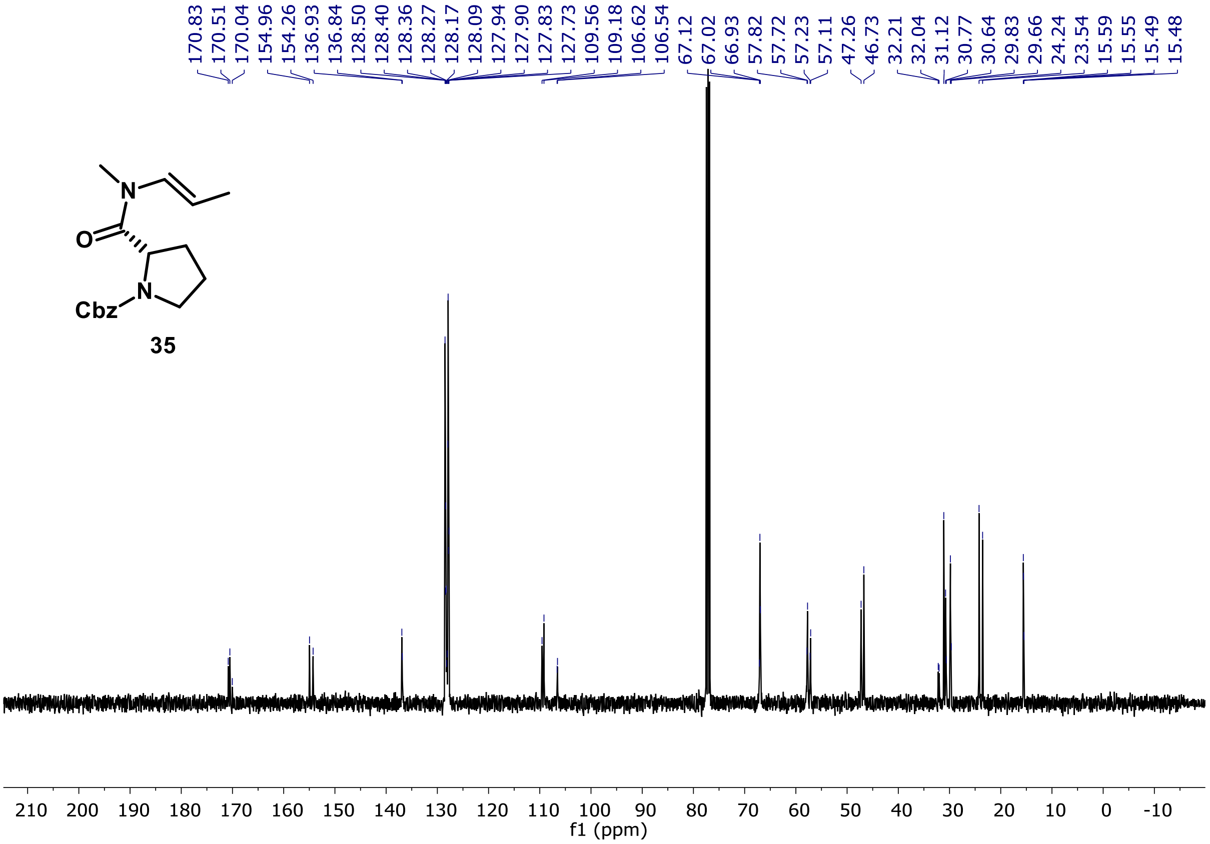

^{1}H (400 MHz, $CDCl_3$)

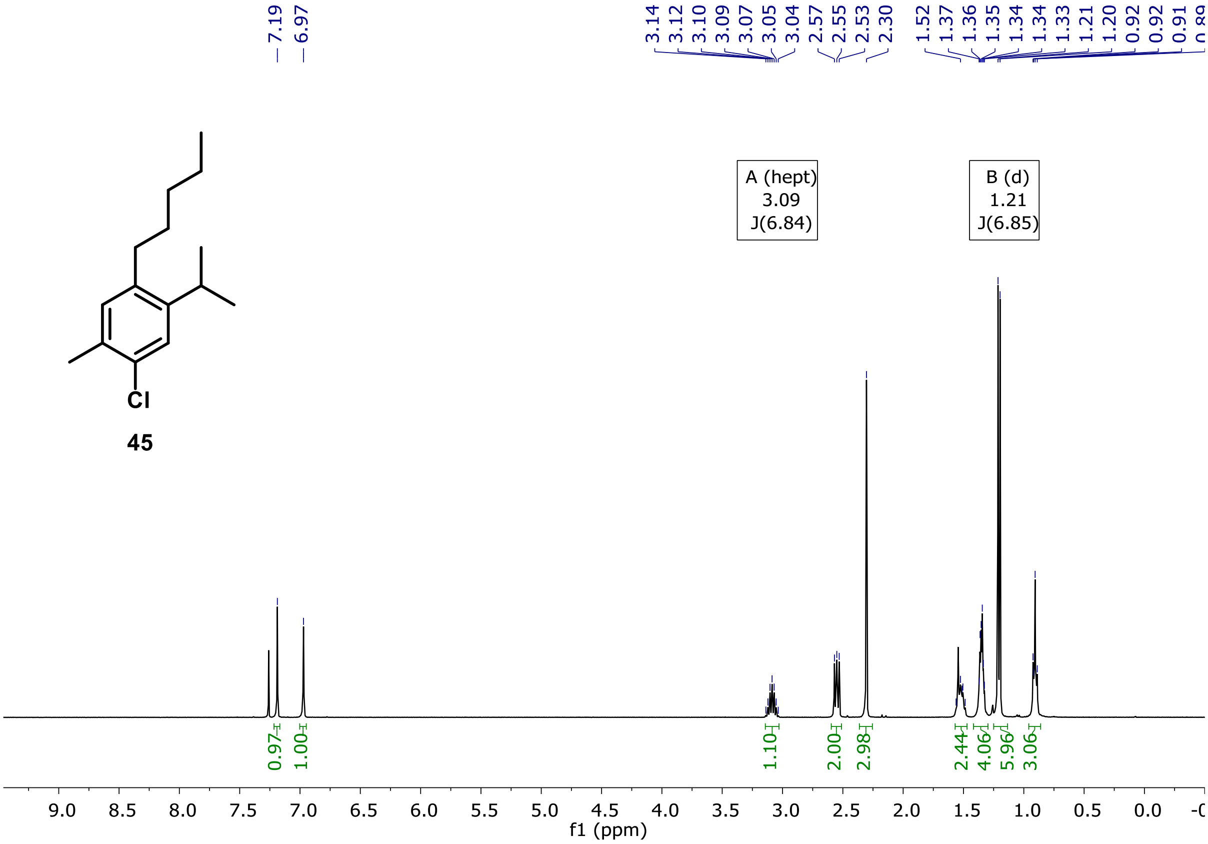

^{13}C (101 MHz, $CDCl_3$)

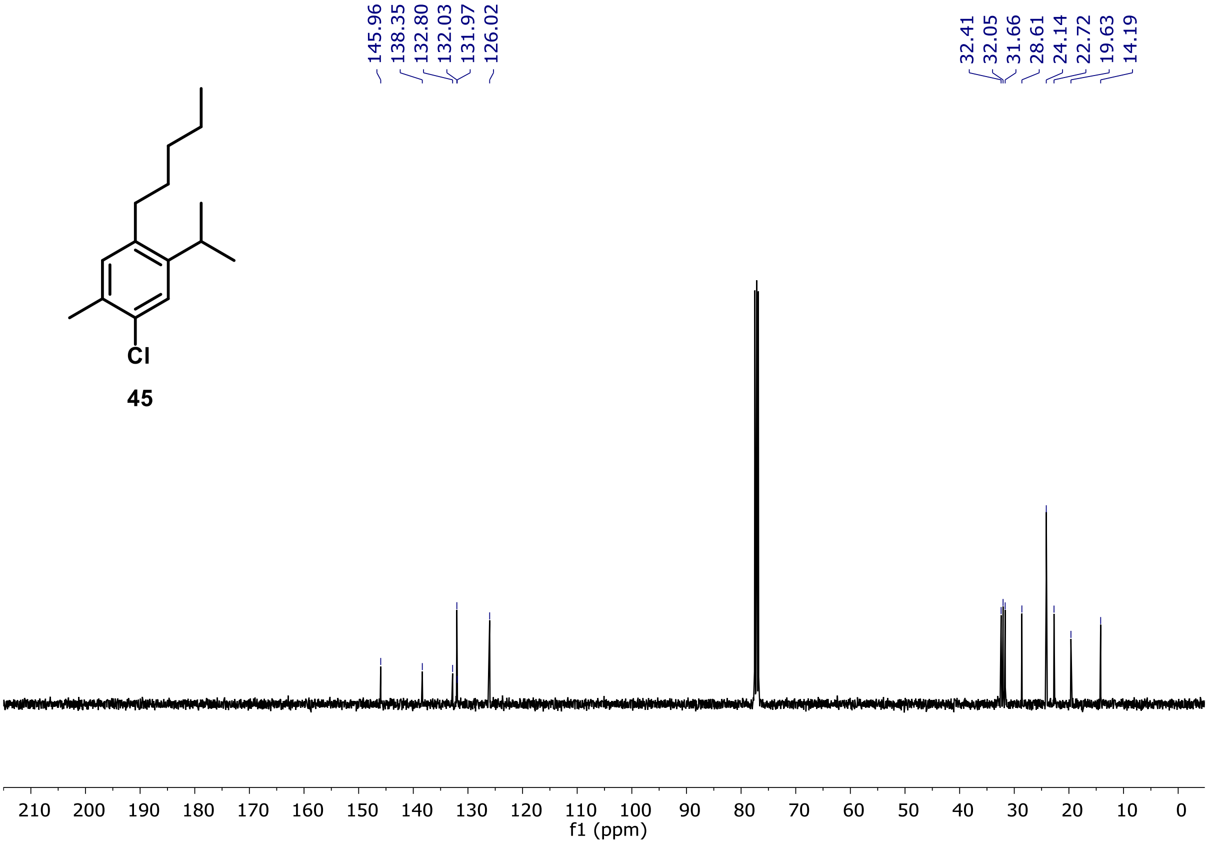

^{1}H (400 MHz, CDCl3)

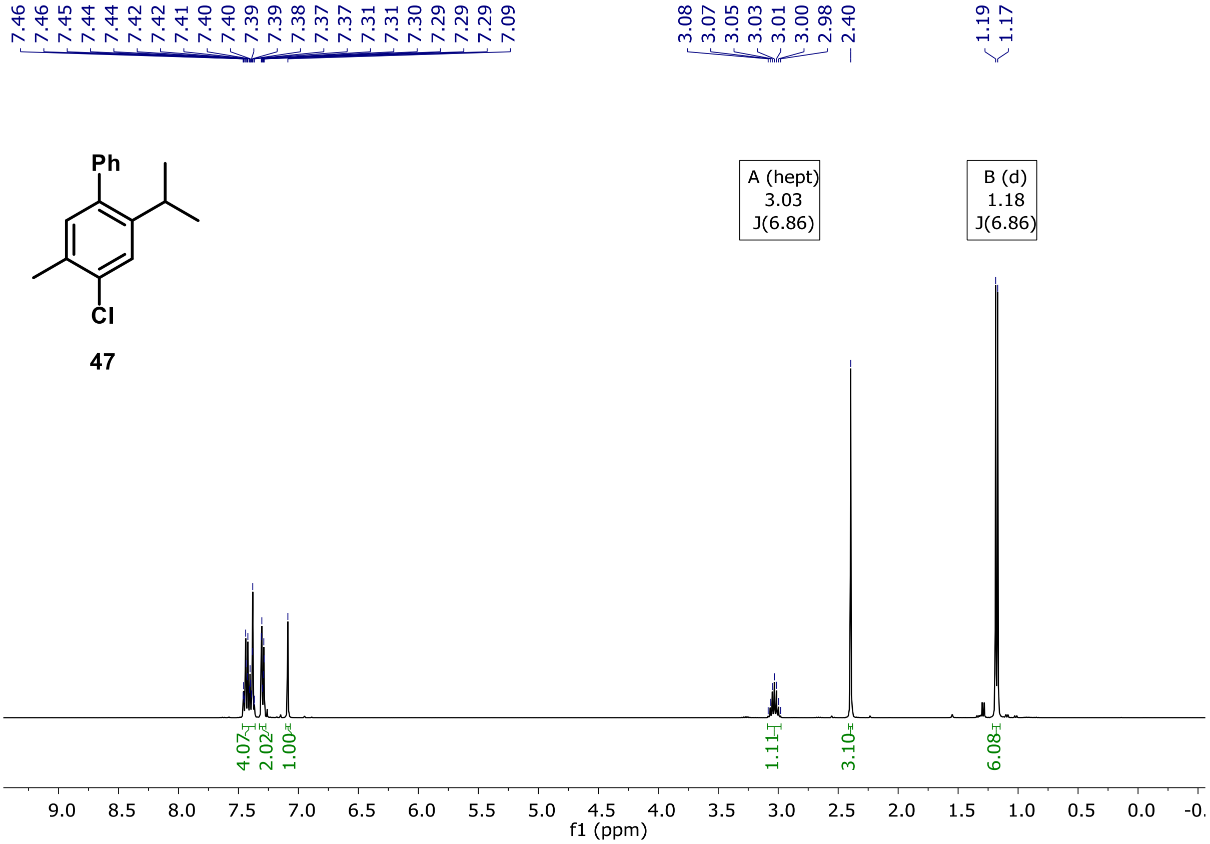

^{13}C (101 MHz, CDCl3)

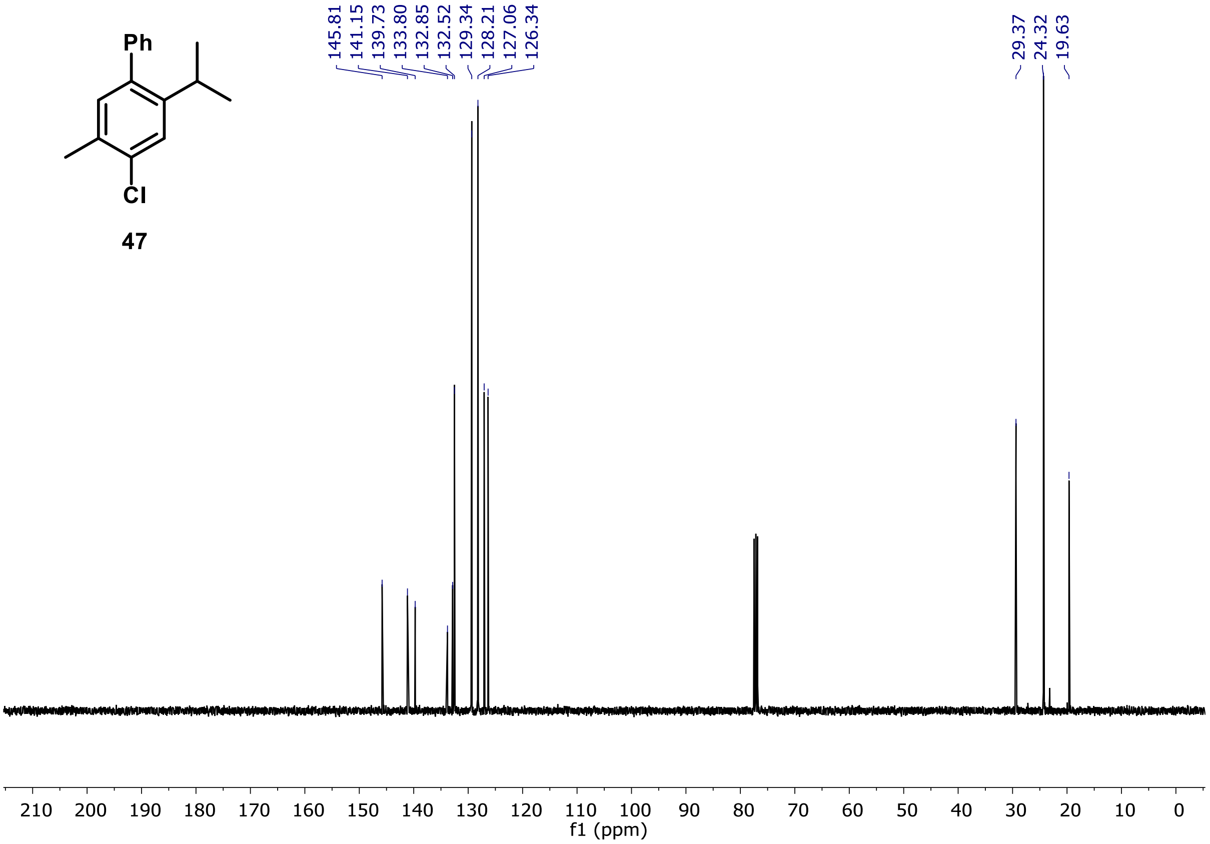

^{1}H (600 MHz, $CDCl_3$, 50°C)

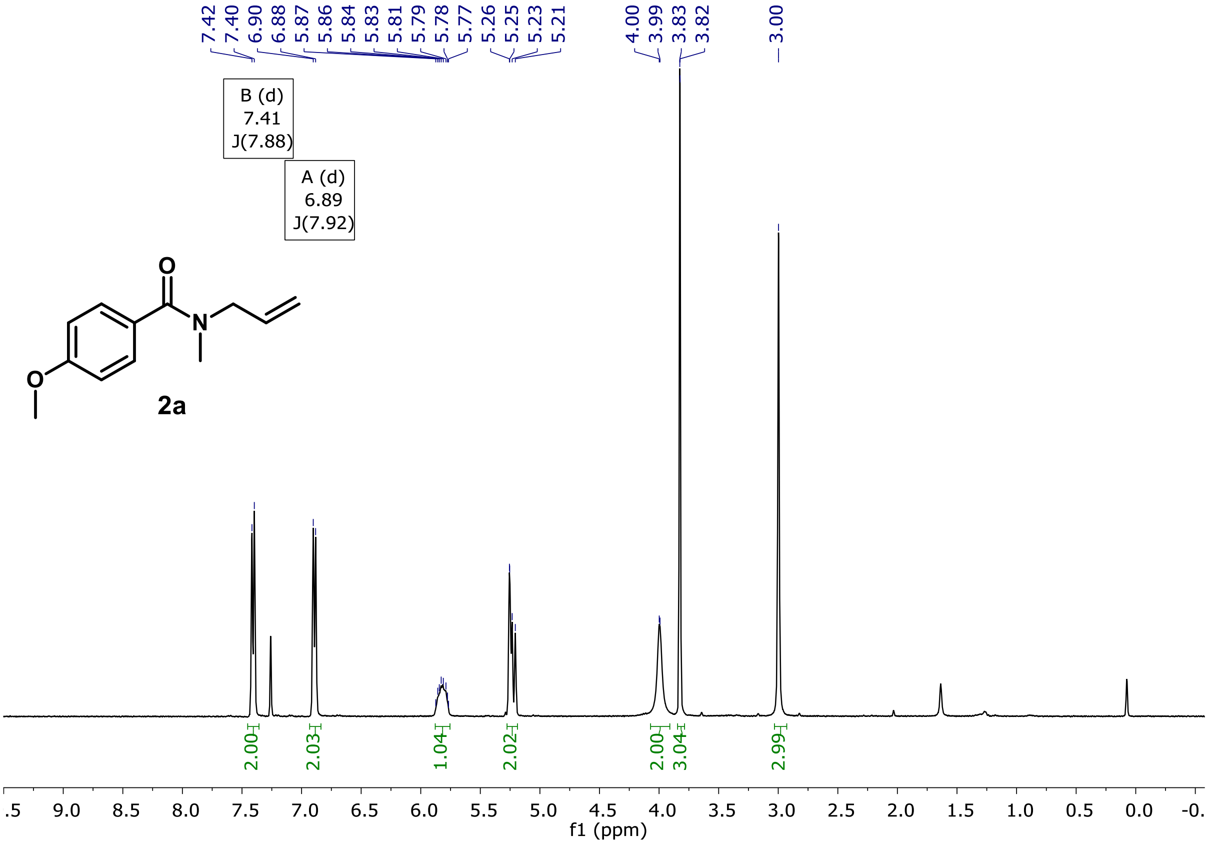

^{13}C (151 MHz, $CDCl_3$)

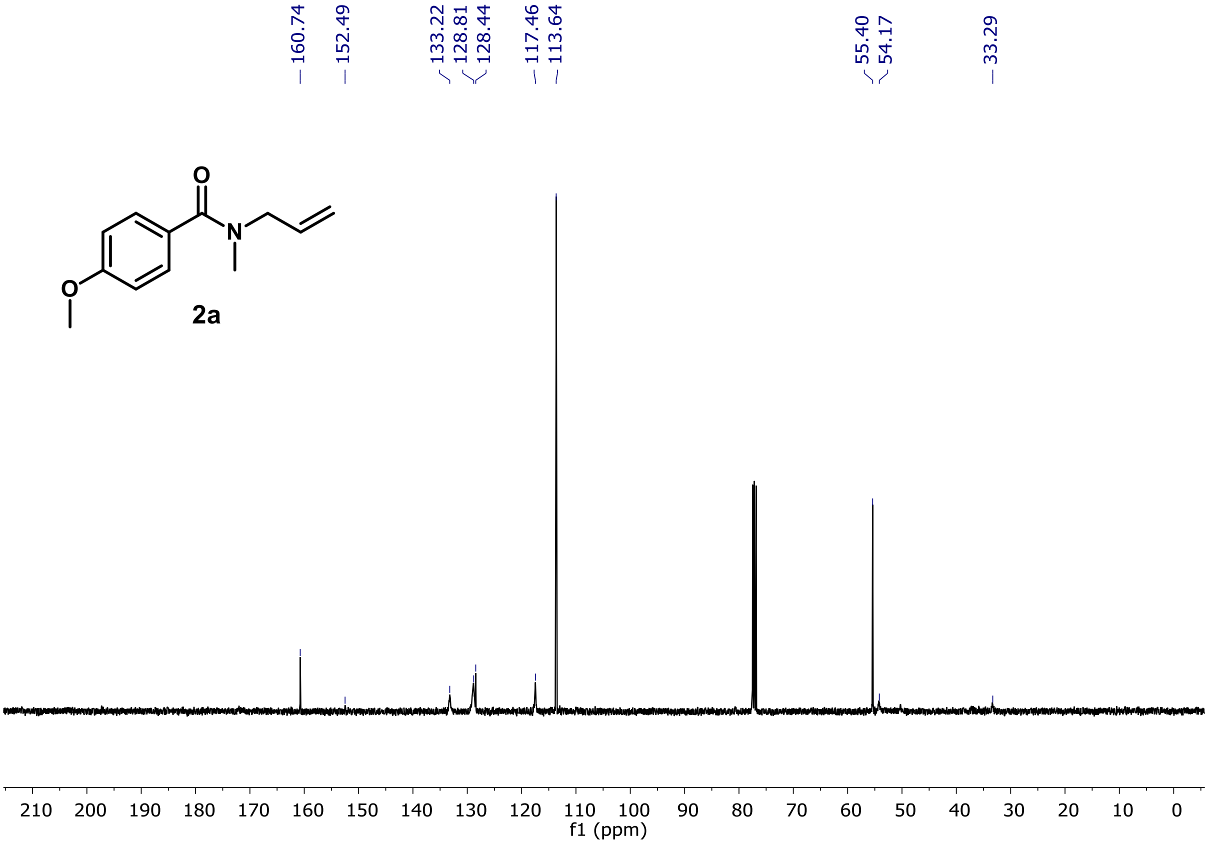

^{1}H (400 MHz, $CDCl_3$)

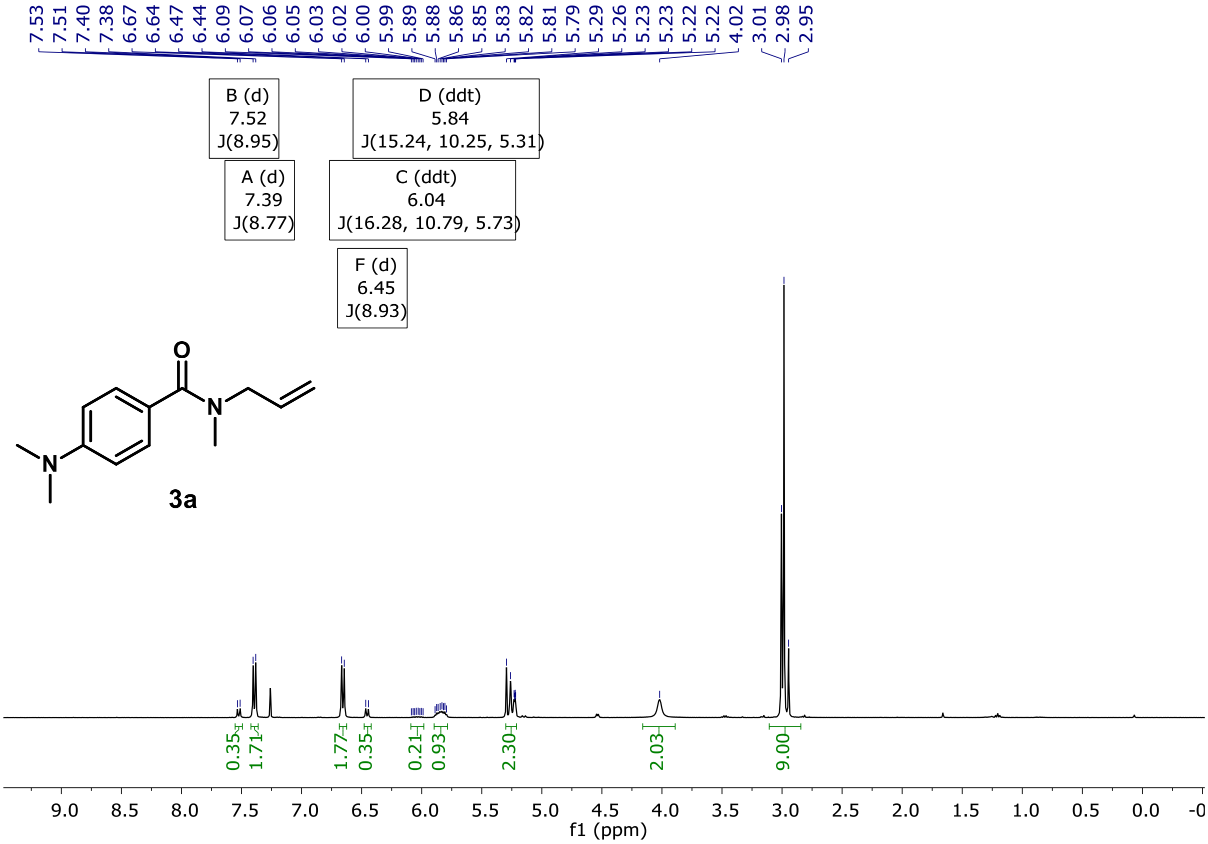

^{13}C (101 MHz, $CDCl_3$)

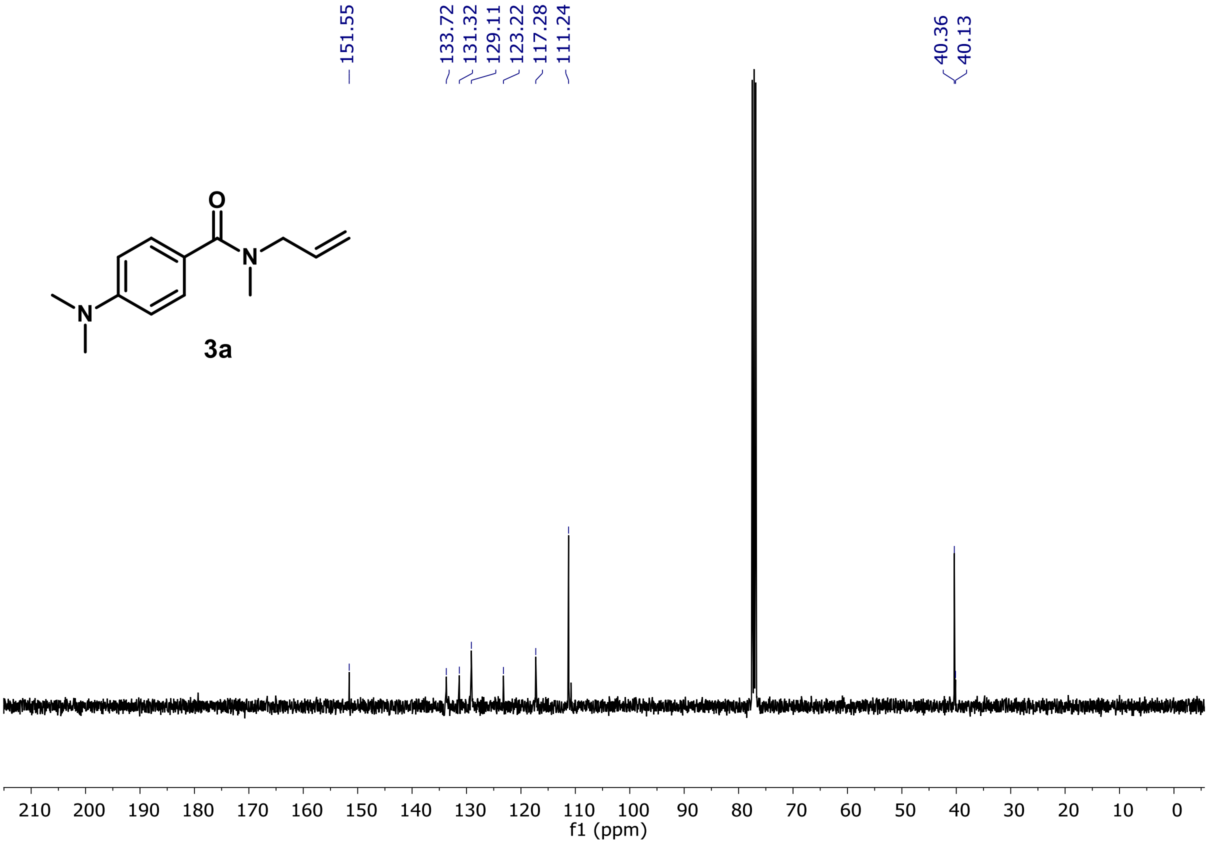

^{1}H (400 MHz, $CDCl_3$)

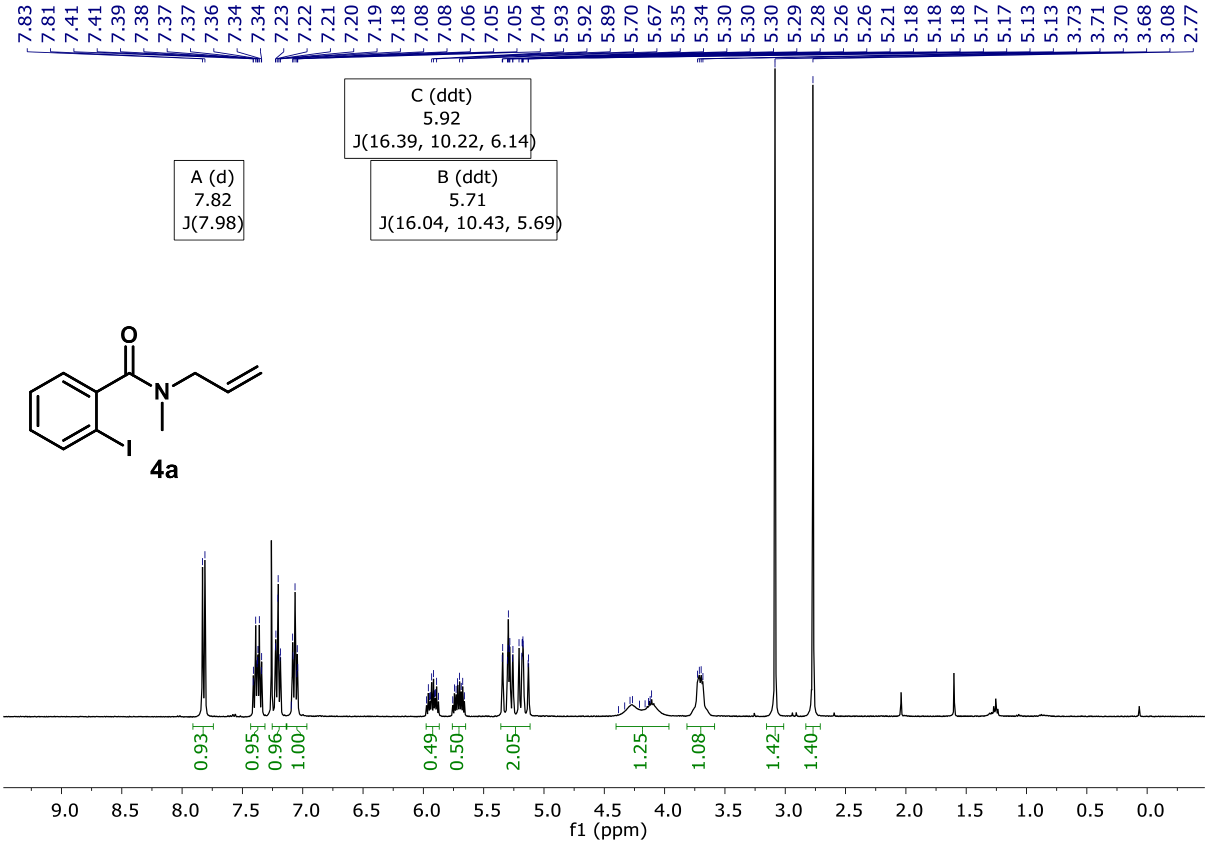

^{13}C (101 MHz, $CDCl_3$)

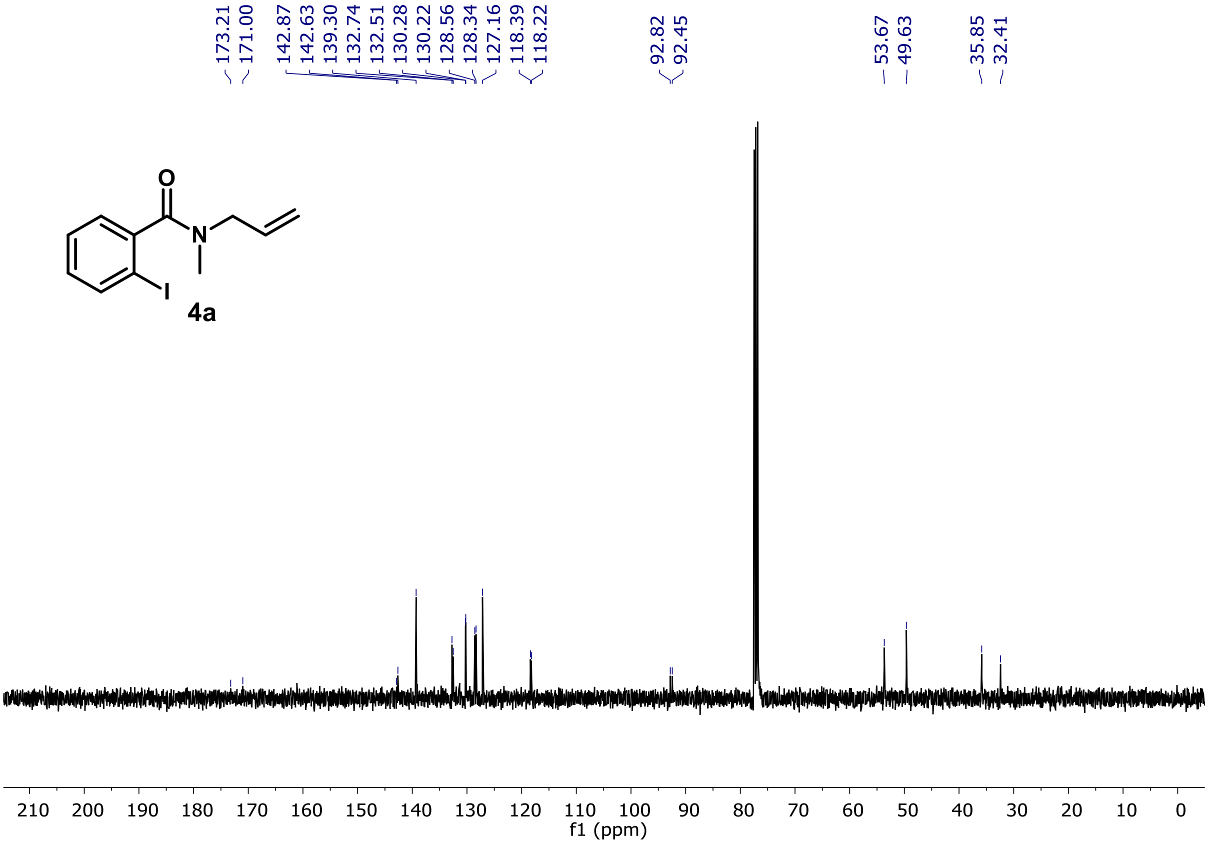

^1H (600 MHz, $CDCl_3$)

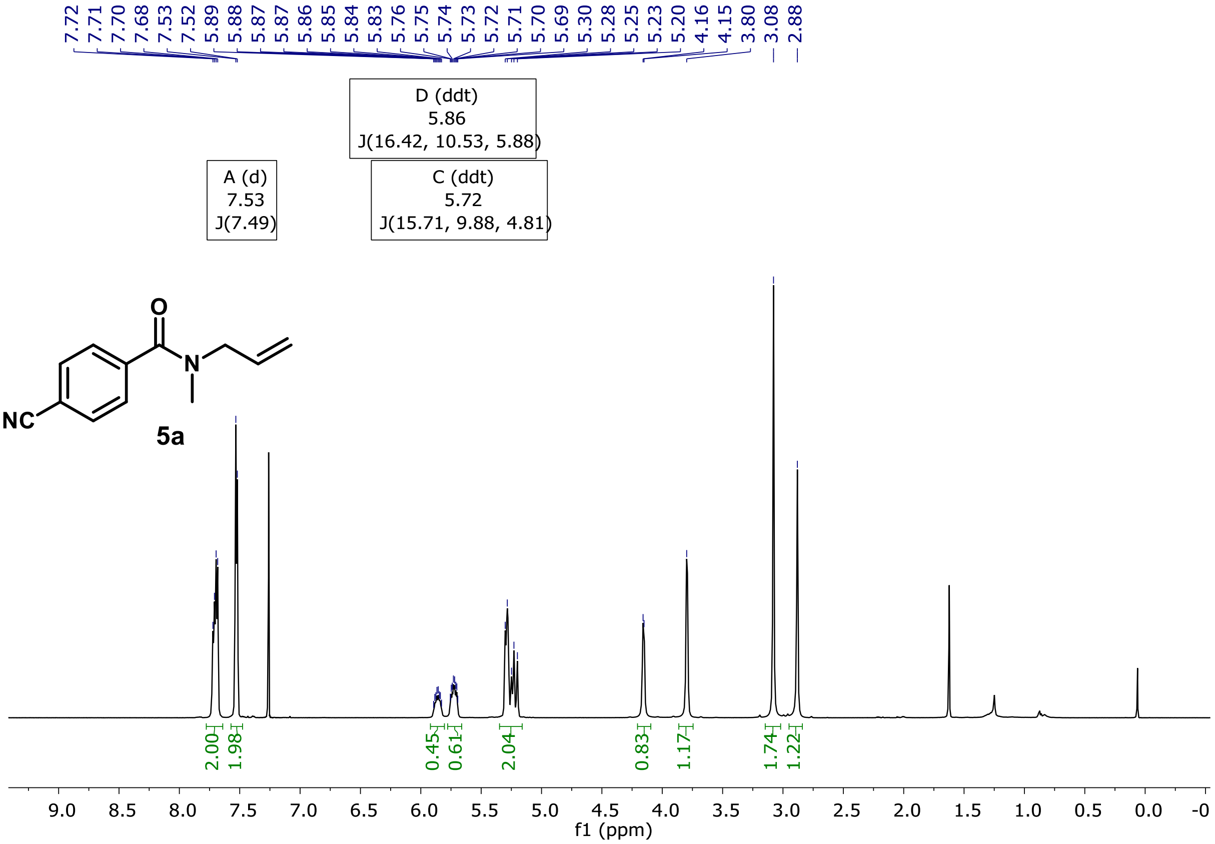

^{13}C (151 MHz, $CDCl_3$)

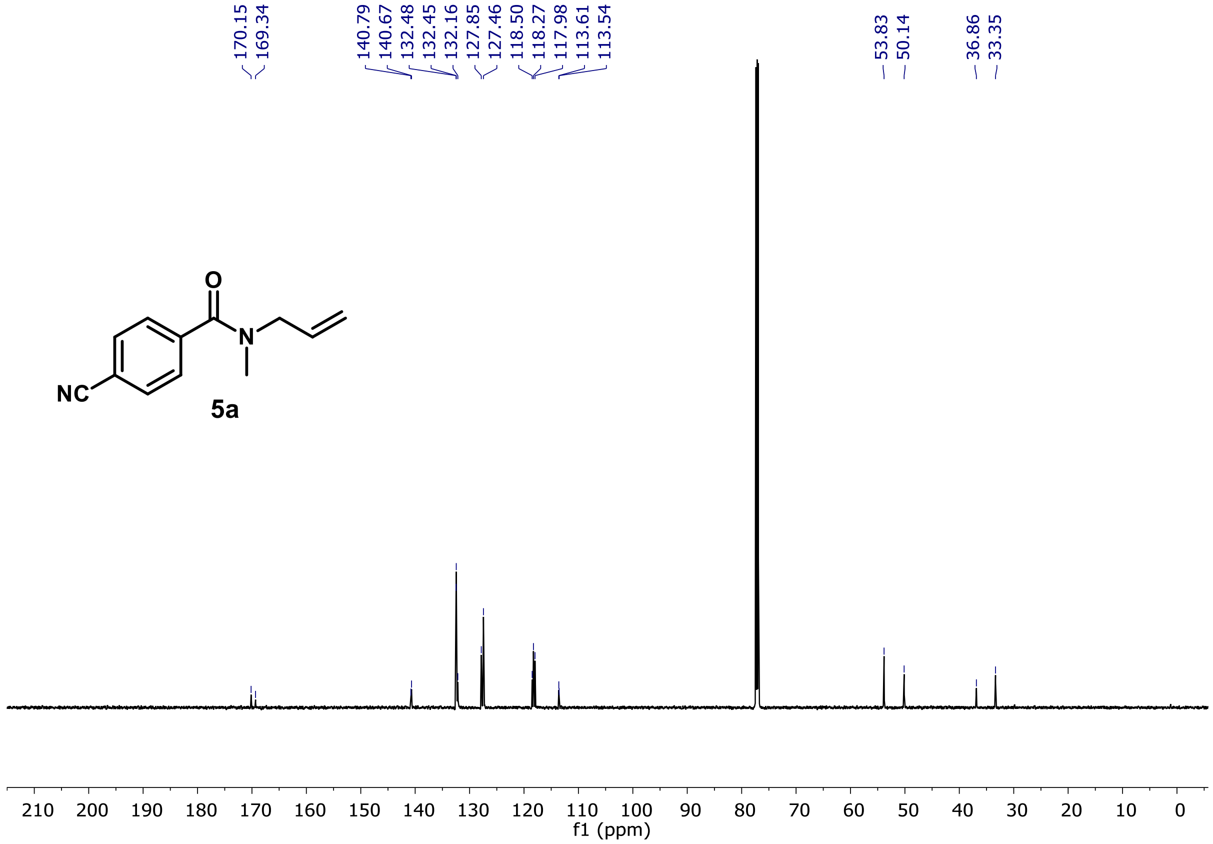

^{1}H (600 MHz, $CDCl_3$)

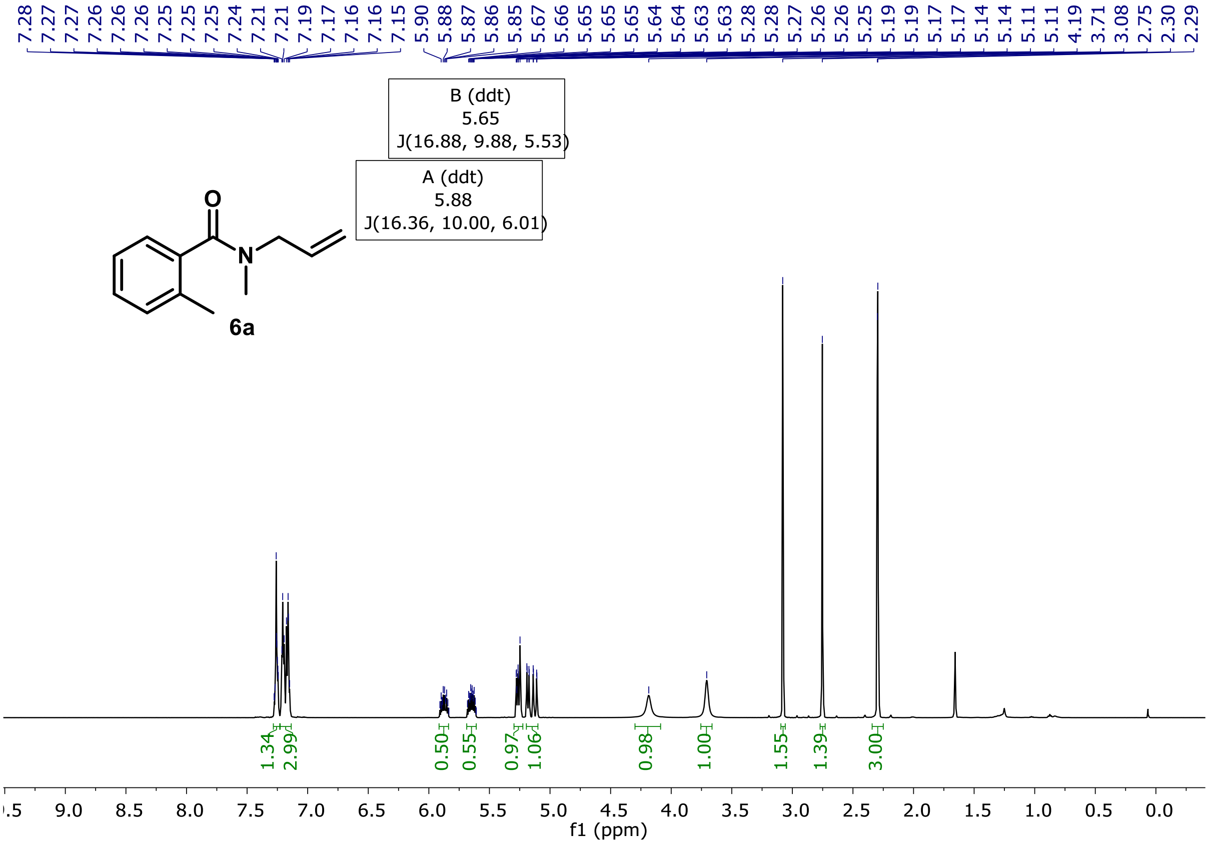

^{13}C (151 MHz, $CDCl_3$)

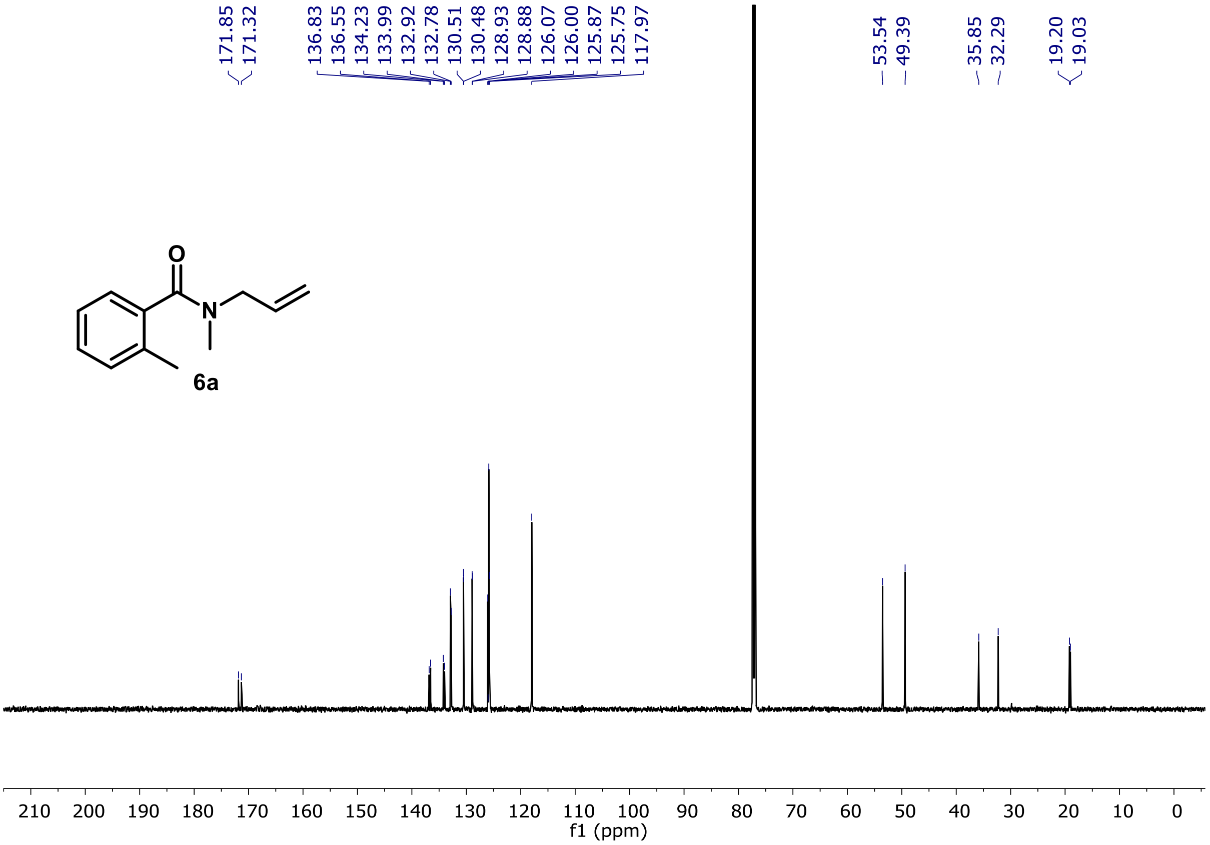

^{1}H (600 MHz, $CDCl_3$)

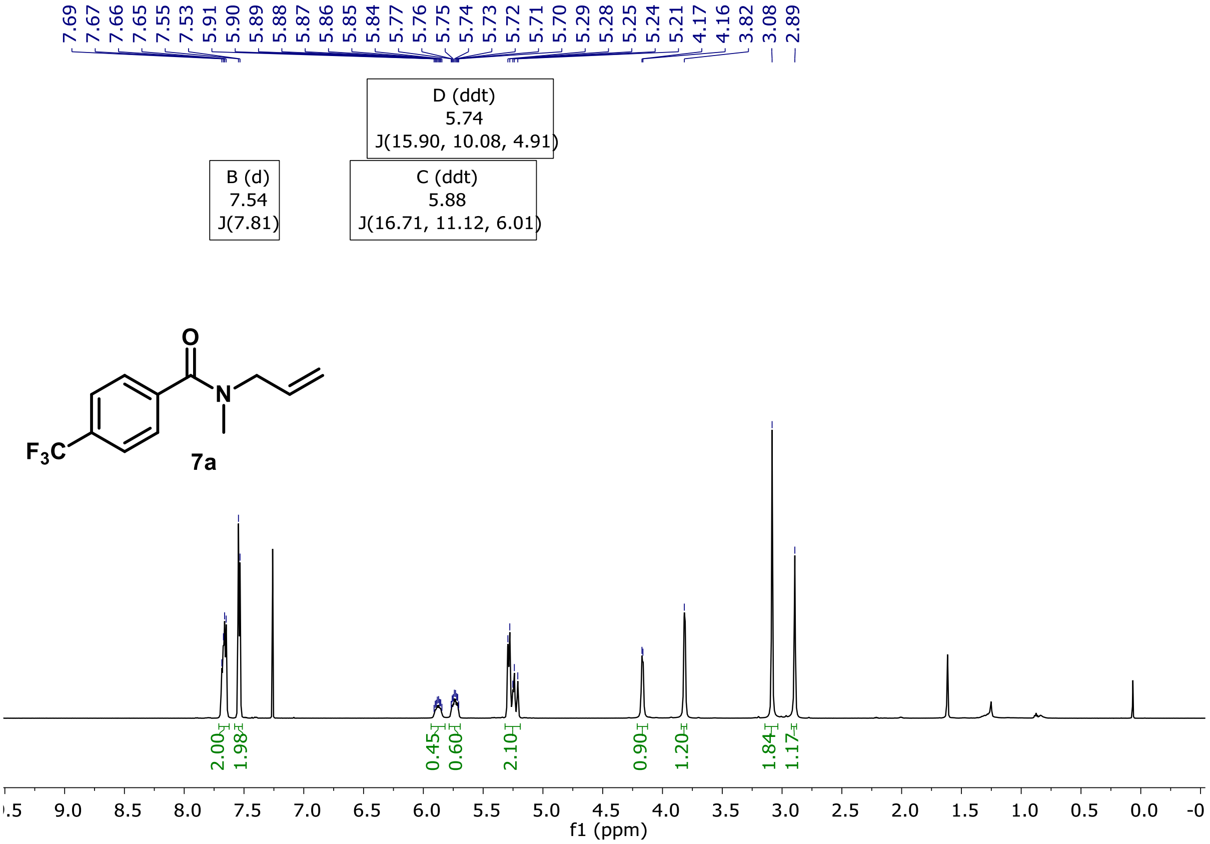

^{13}C (151 MHz, $CDCl_3$)

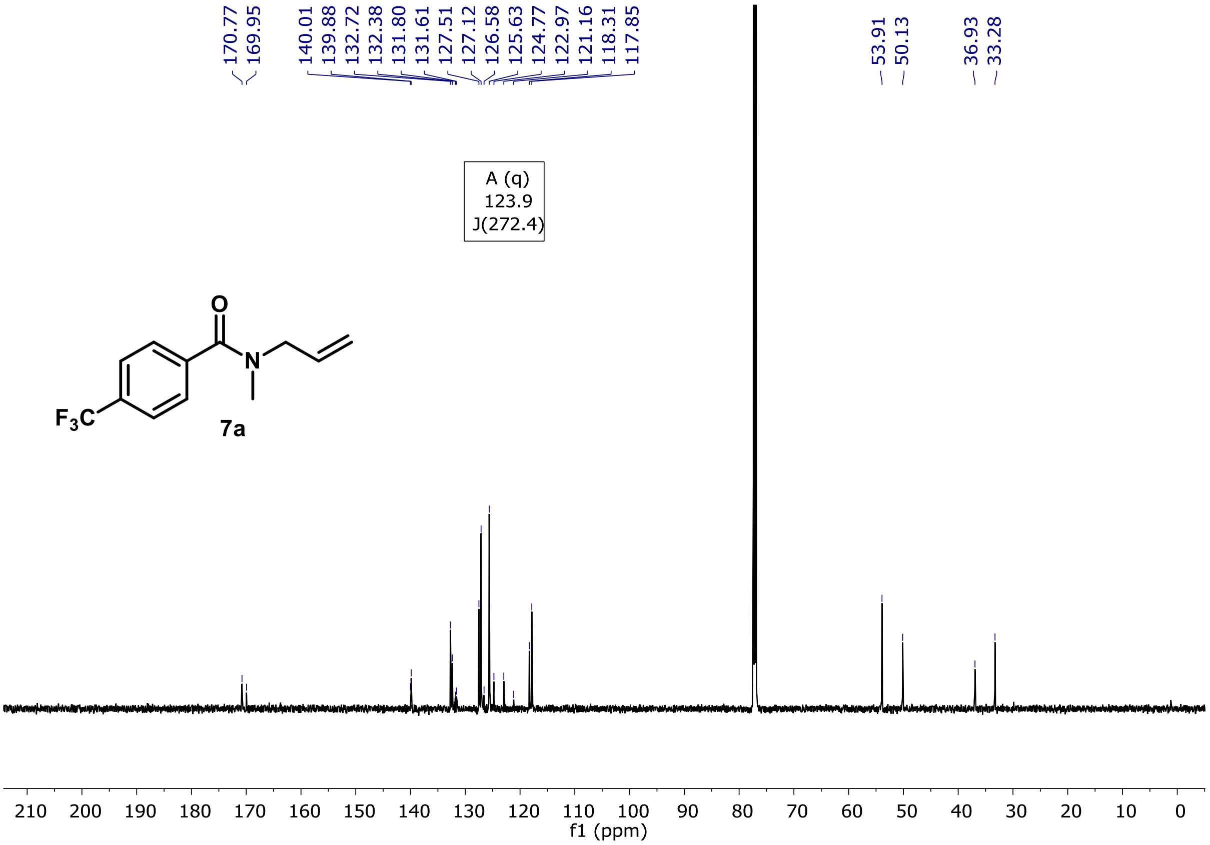

^{19}F (564 MHz, $CDCl_3$)

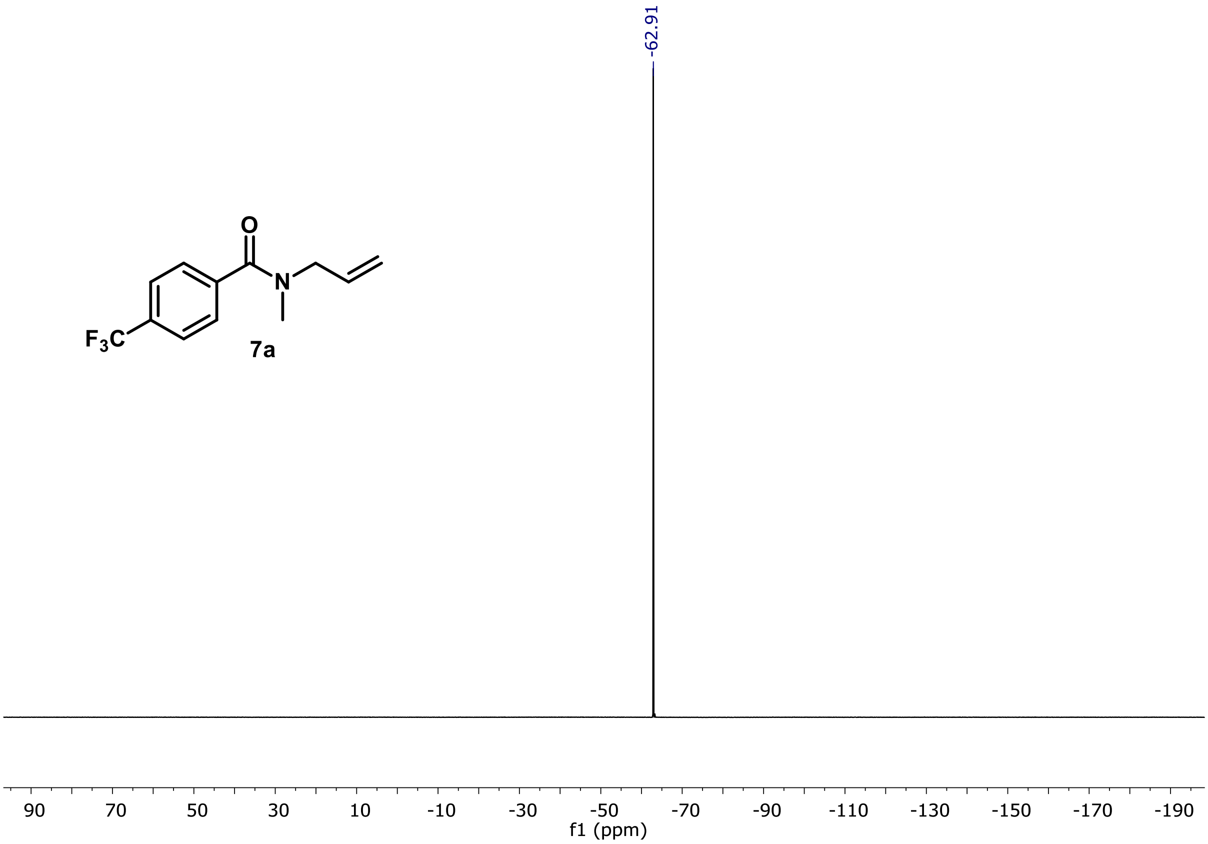

^{1}H (400 MHz, $CDCl_3$)

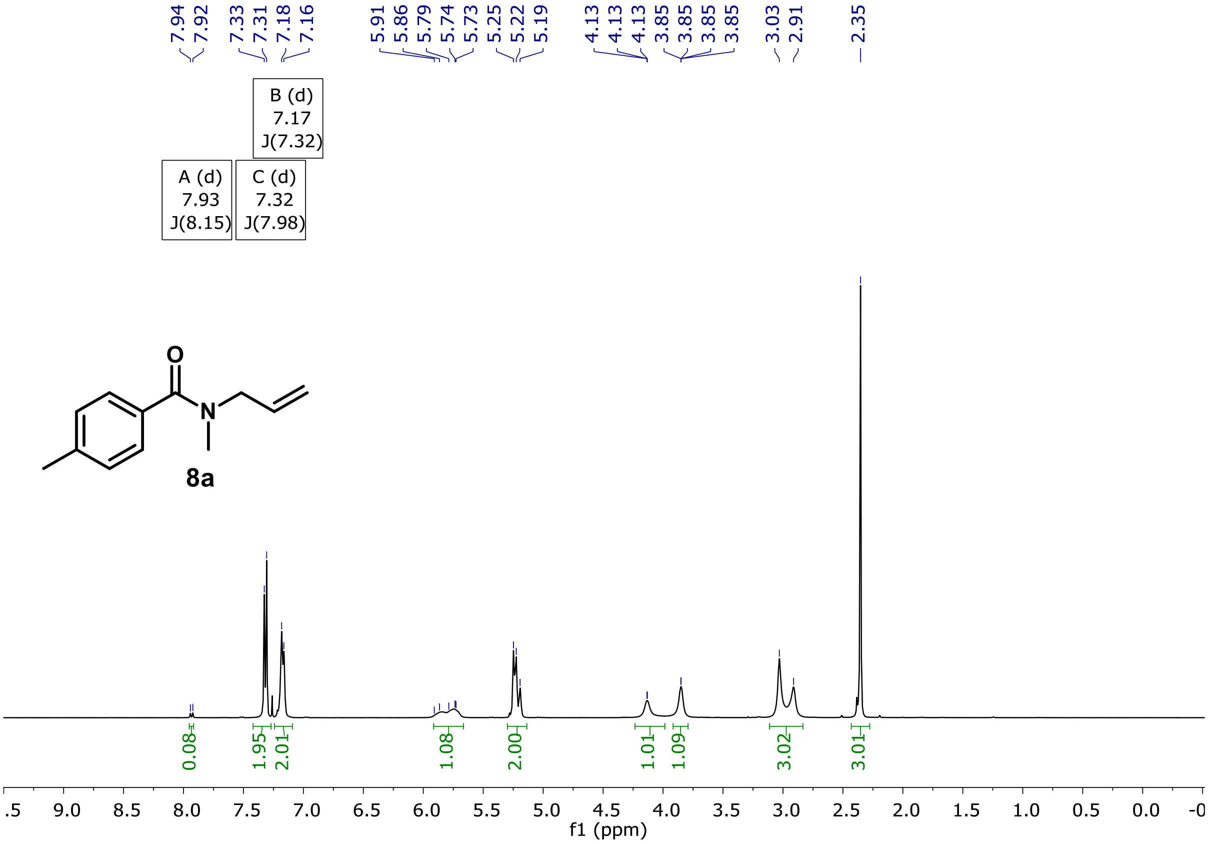

^{13}C (101 MHz, $CDCl_3$)

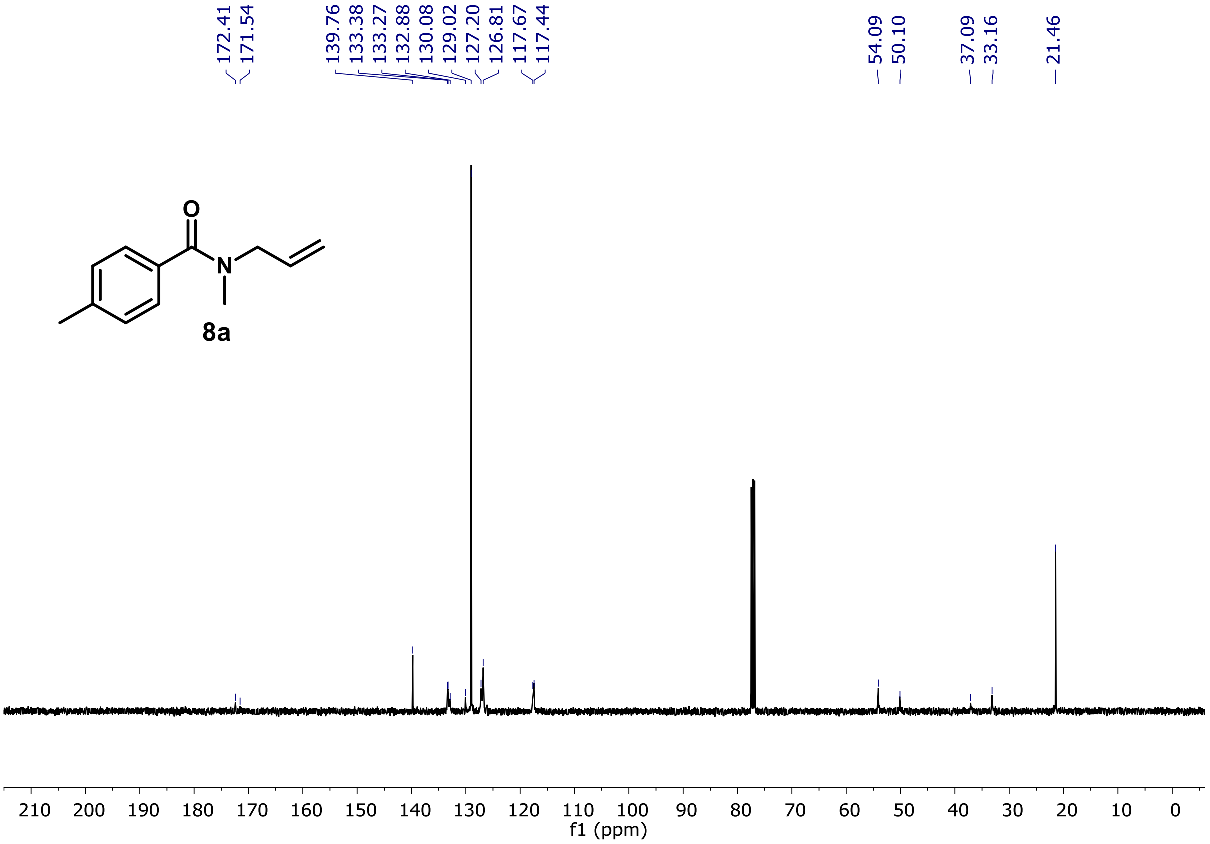

^{1}H (600 MHz, $CDCl_3$)

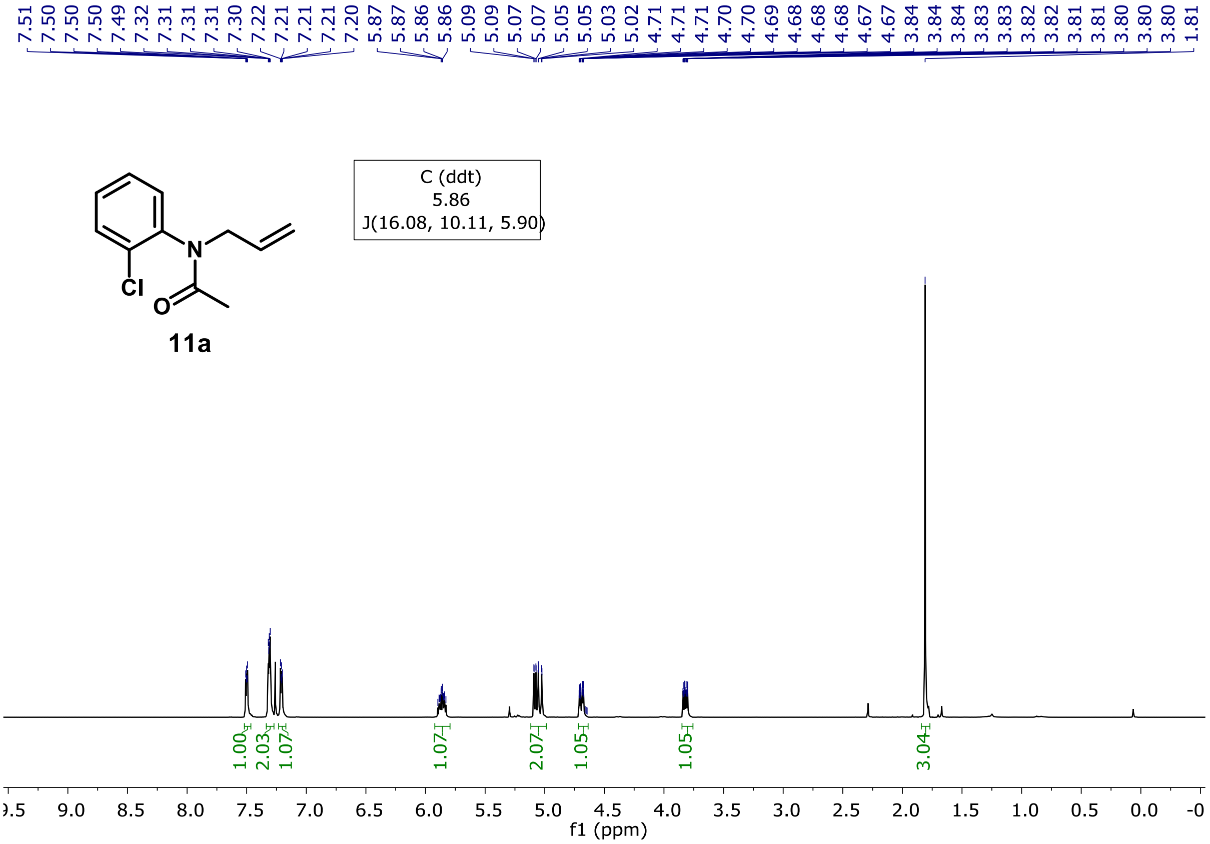

^{13}C (151 MHz, $CDCl_3$)

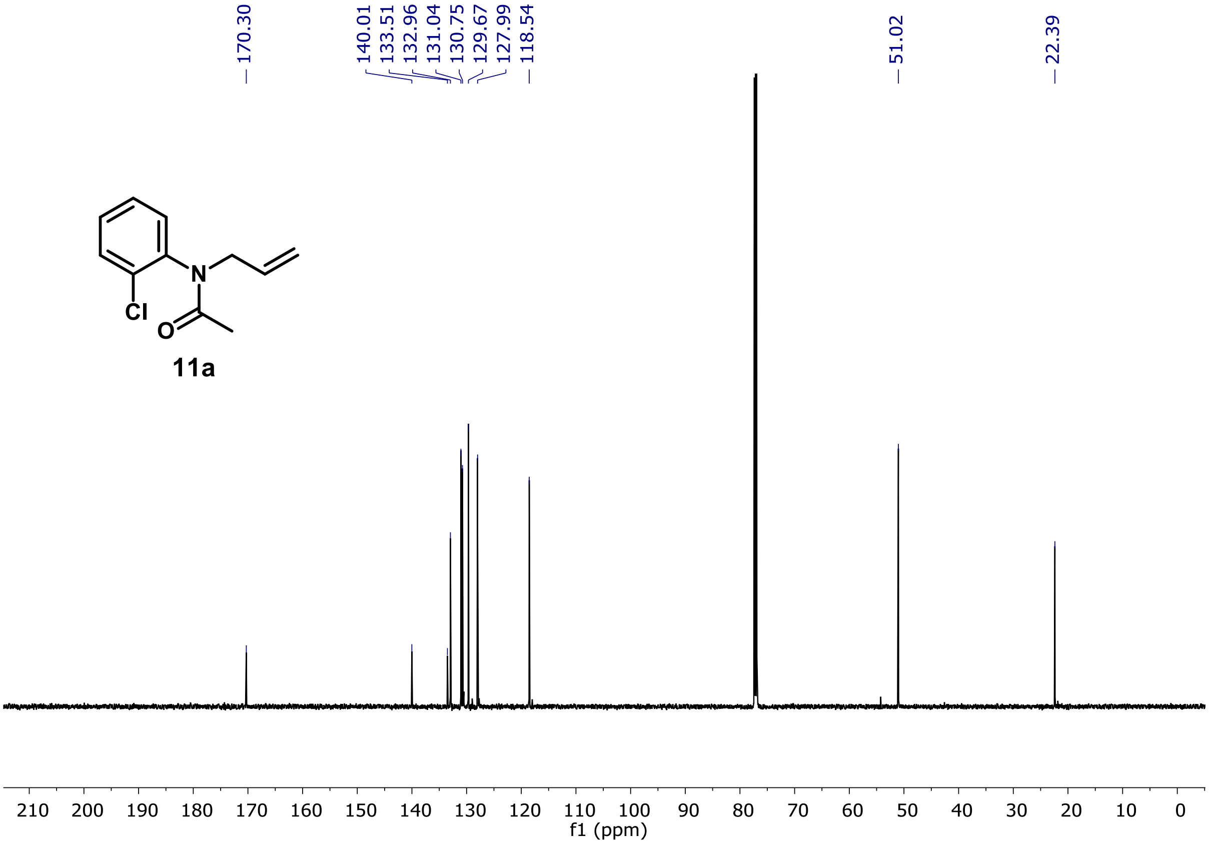

11a

^{1}H (400 MHz, $CDCl_3$)

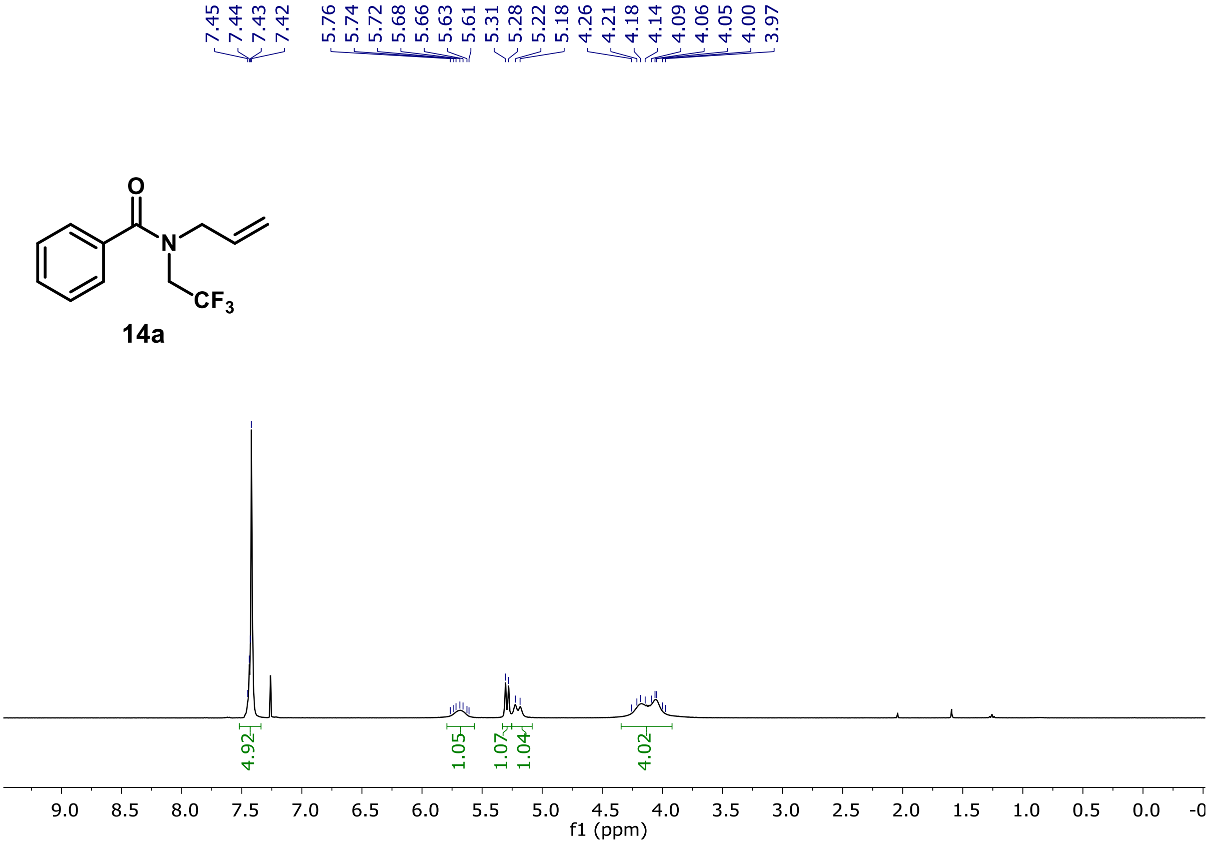

14a

^{13}C (101 MHz, $CDCl_3$)

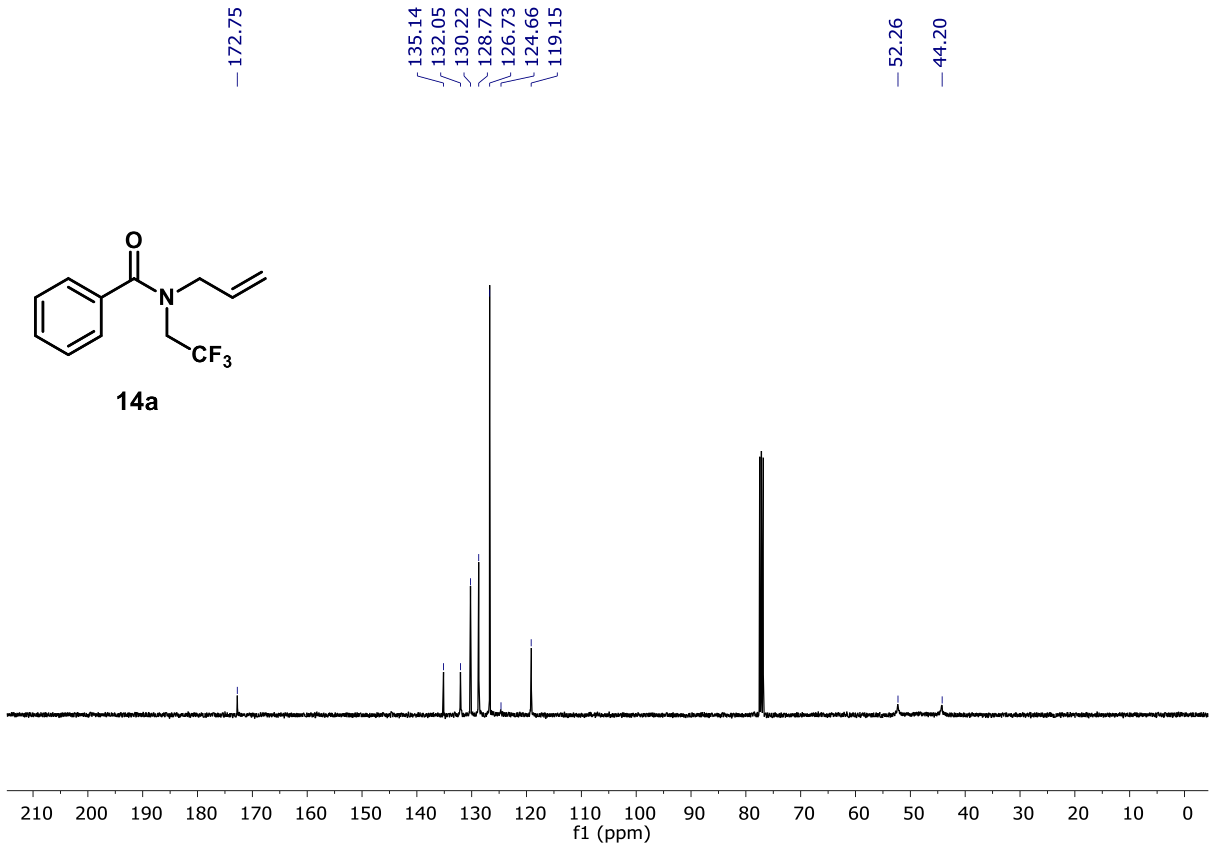

^{19}F (376 MHz, $CDCl_3$)

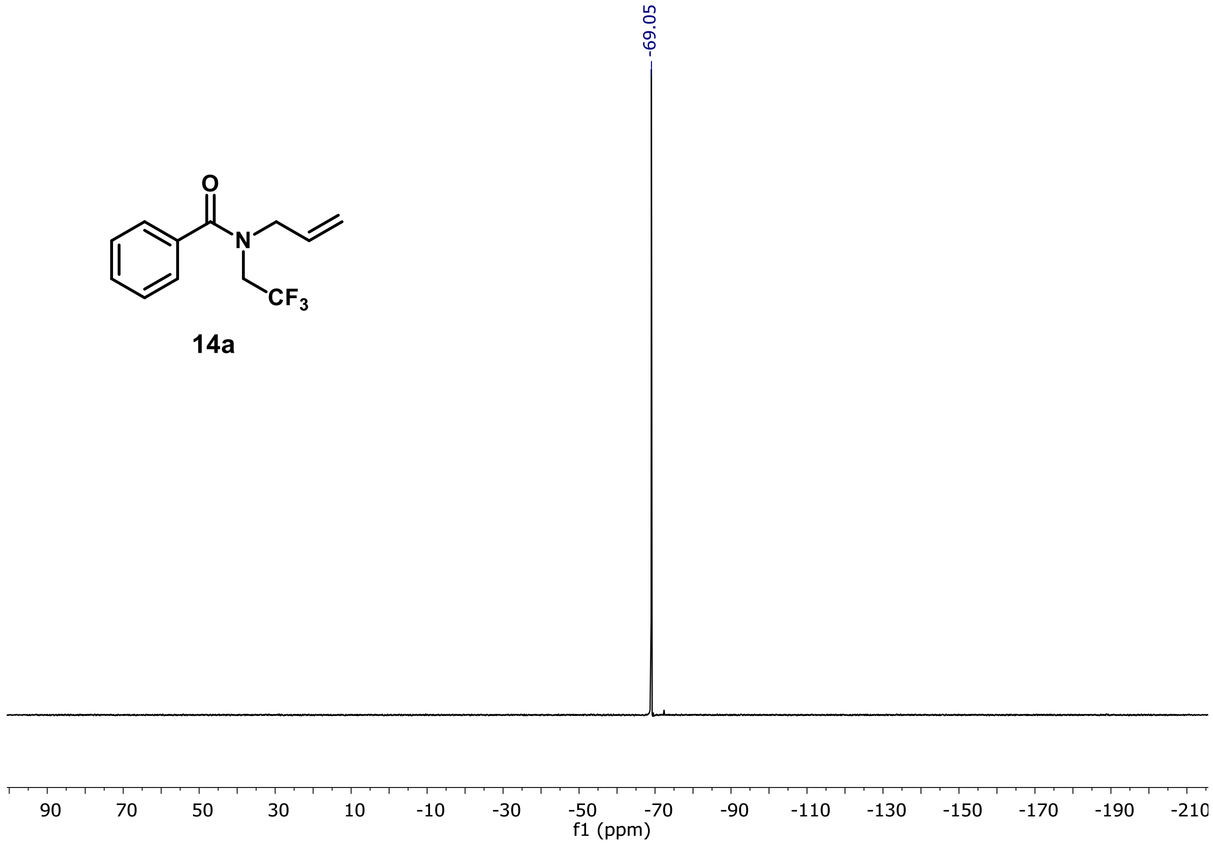

^{1}H (600 MHz, $CDCl_3$)

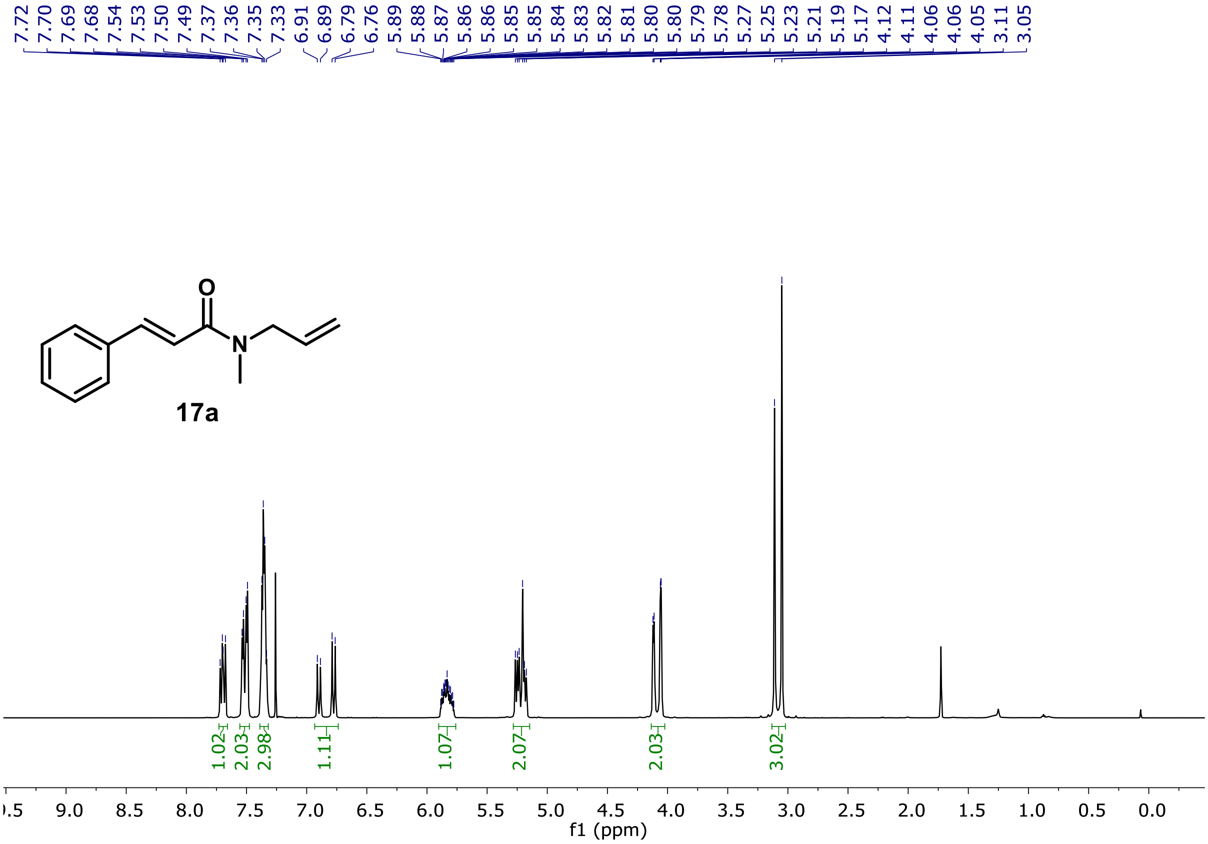

^{13}C (151 MHz, $CDCl_3$)

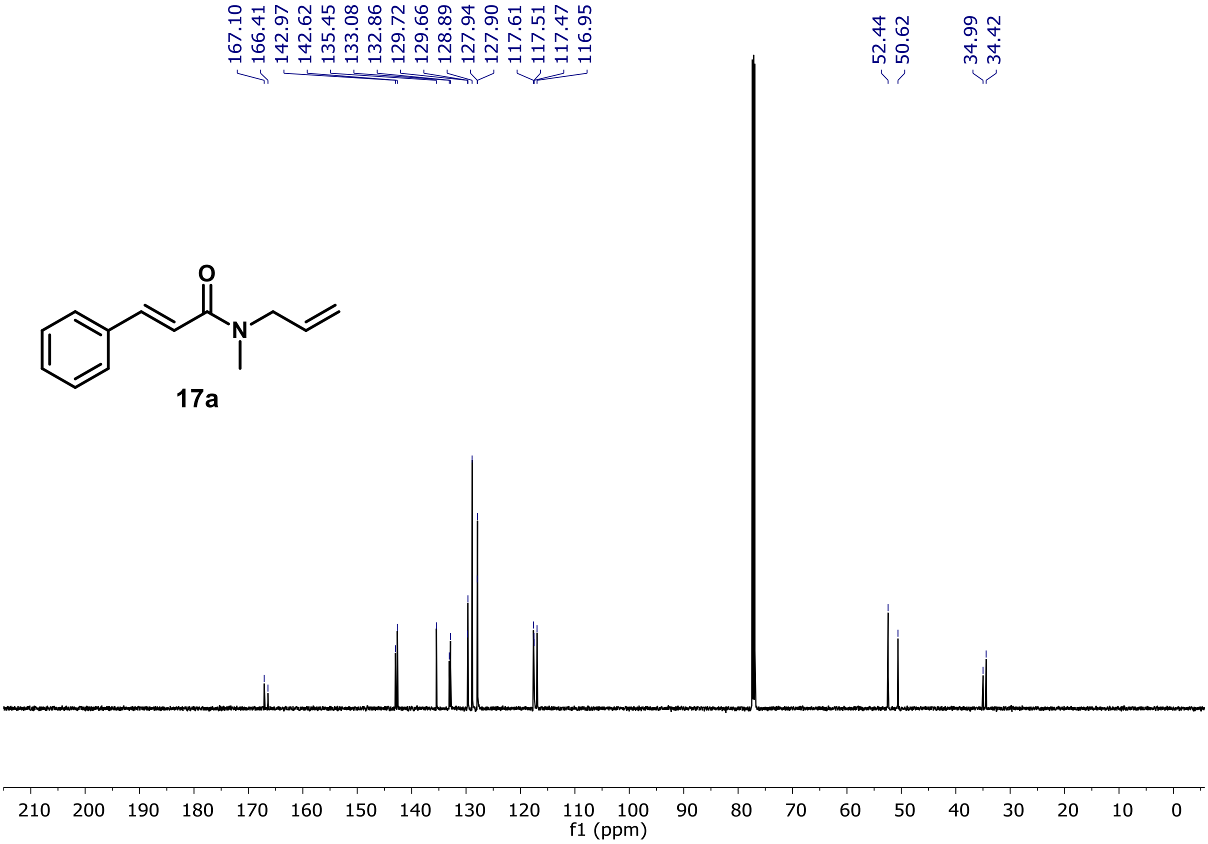

^{1}H (600 MHz, $CDCl_3$)

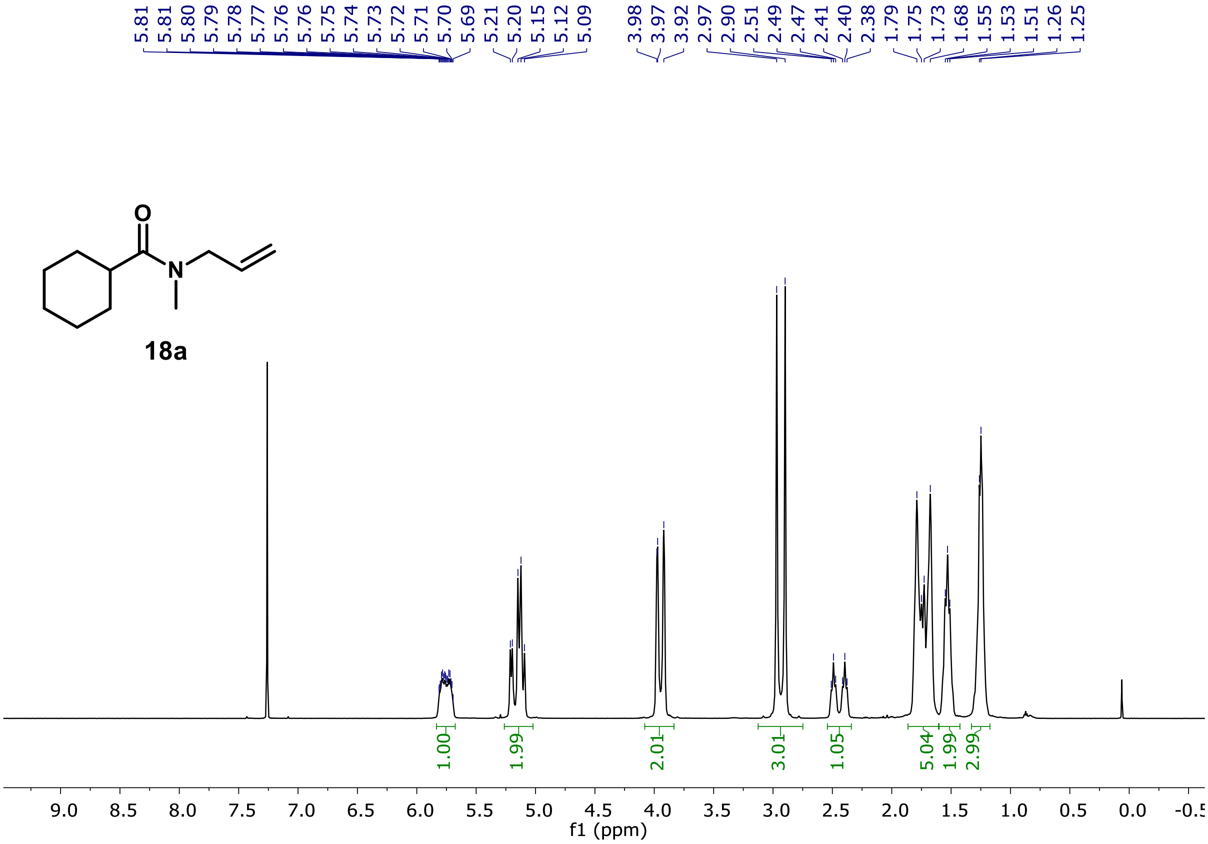

^{13}C (151 MHz, $CDCl_3$)

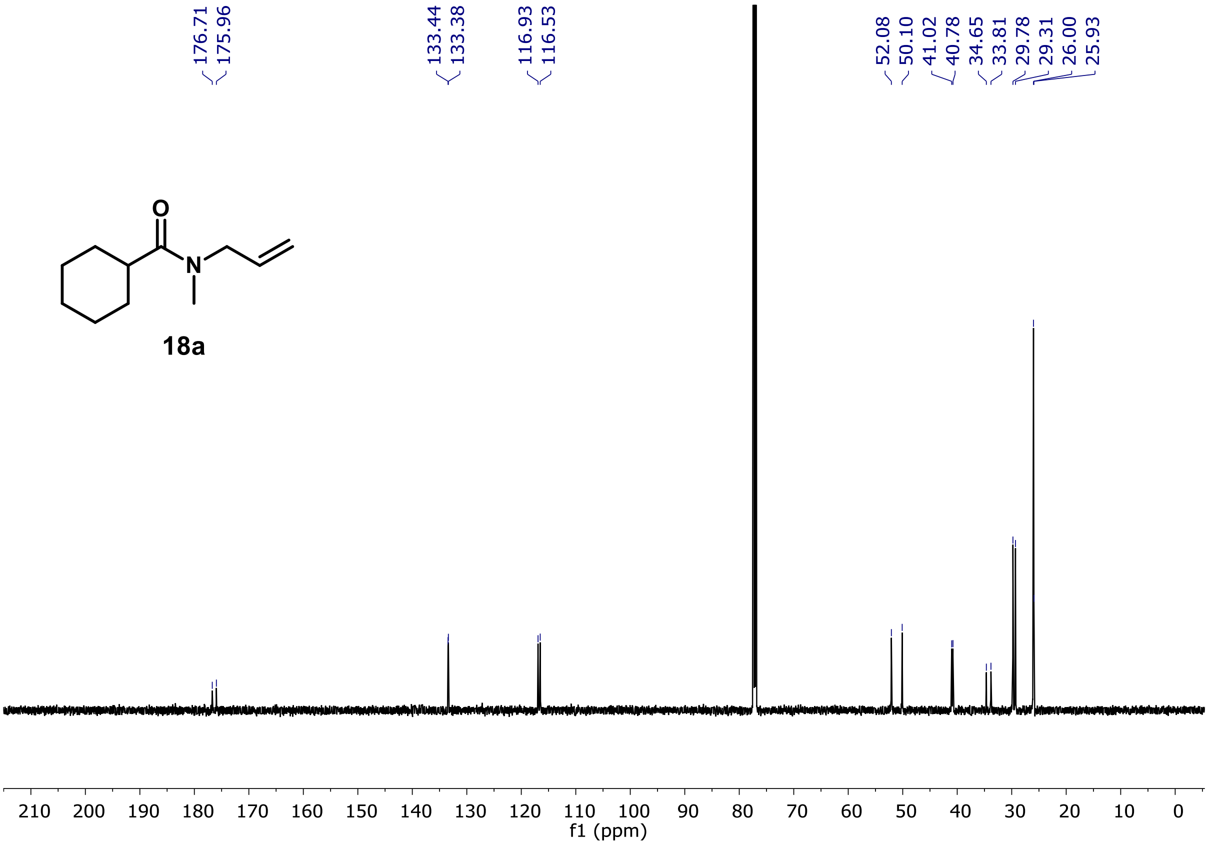

^{1}H (600 MHz, $CDCl_3$)

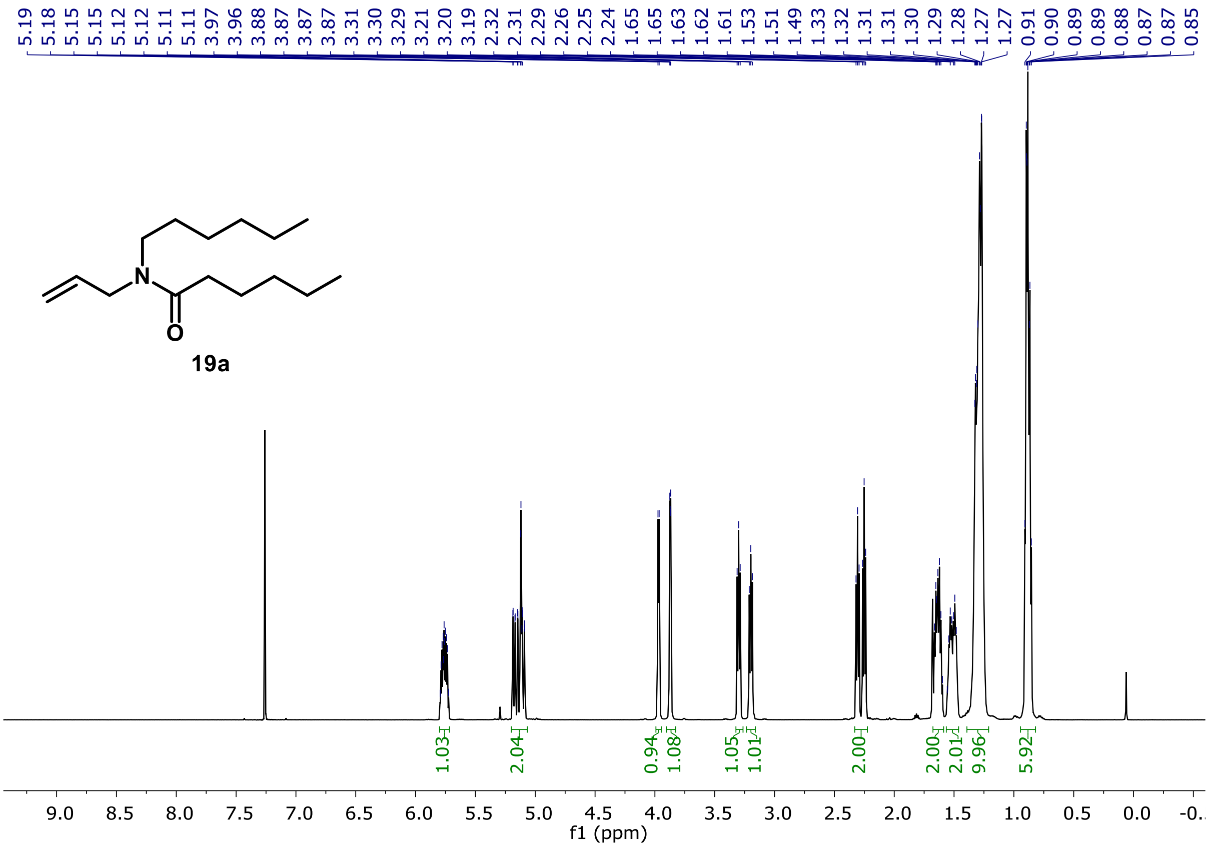

^{13}C (151 MHz, $CDCl_3$)

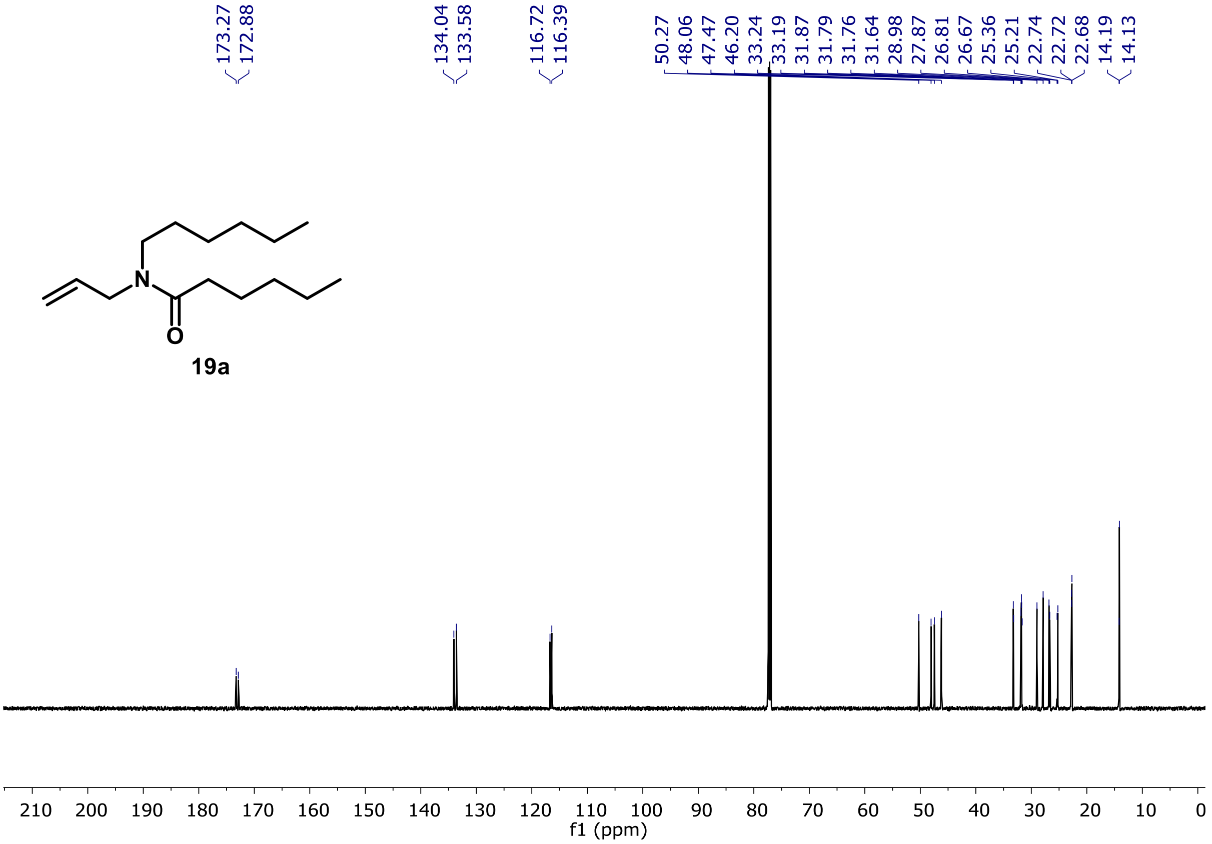

^{1}H (400 MHz, $CDCl_3$)

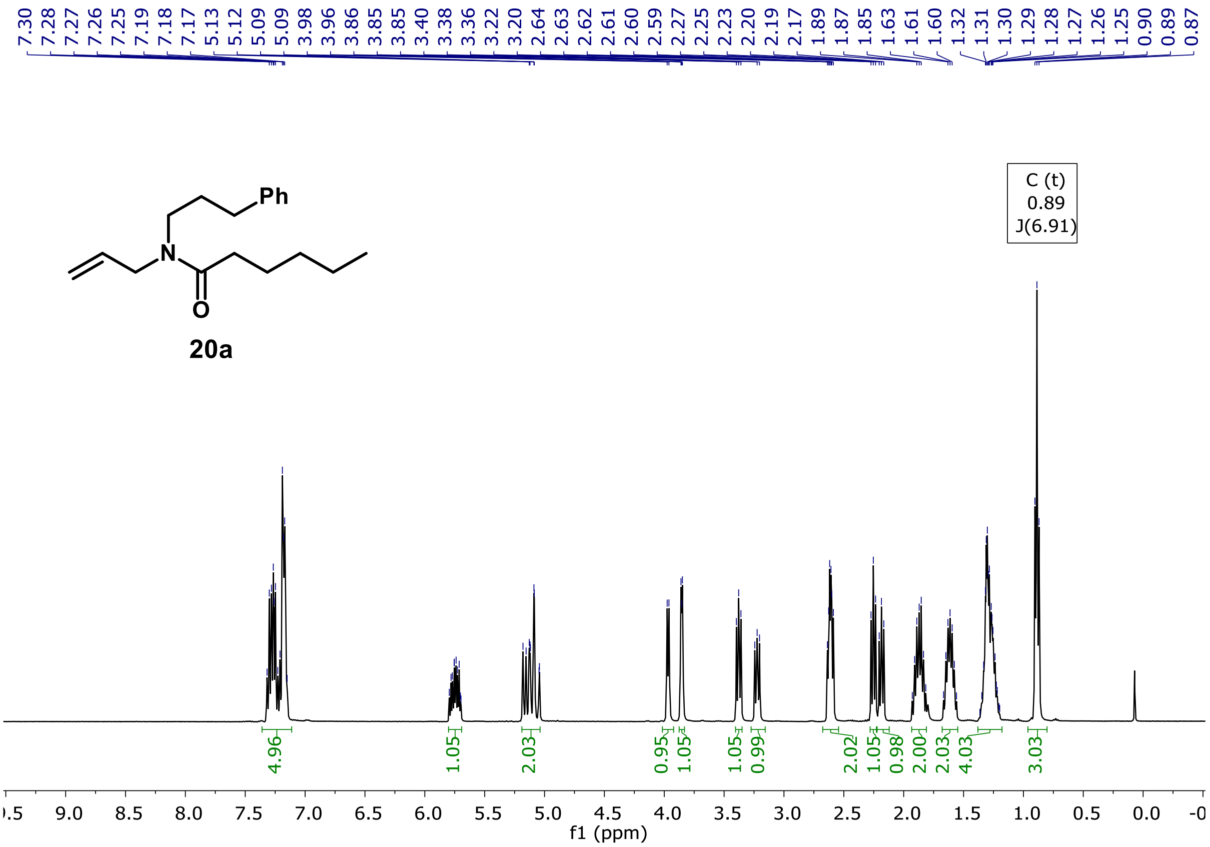

^{13}C (121 MHz, $CDCl_3$)

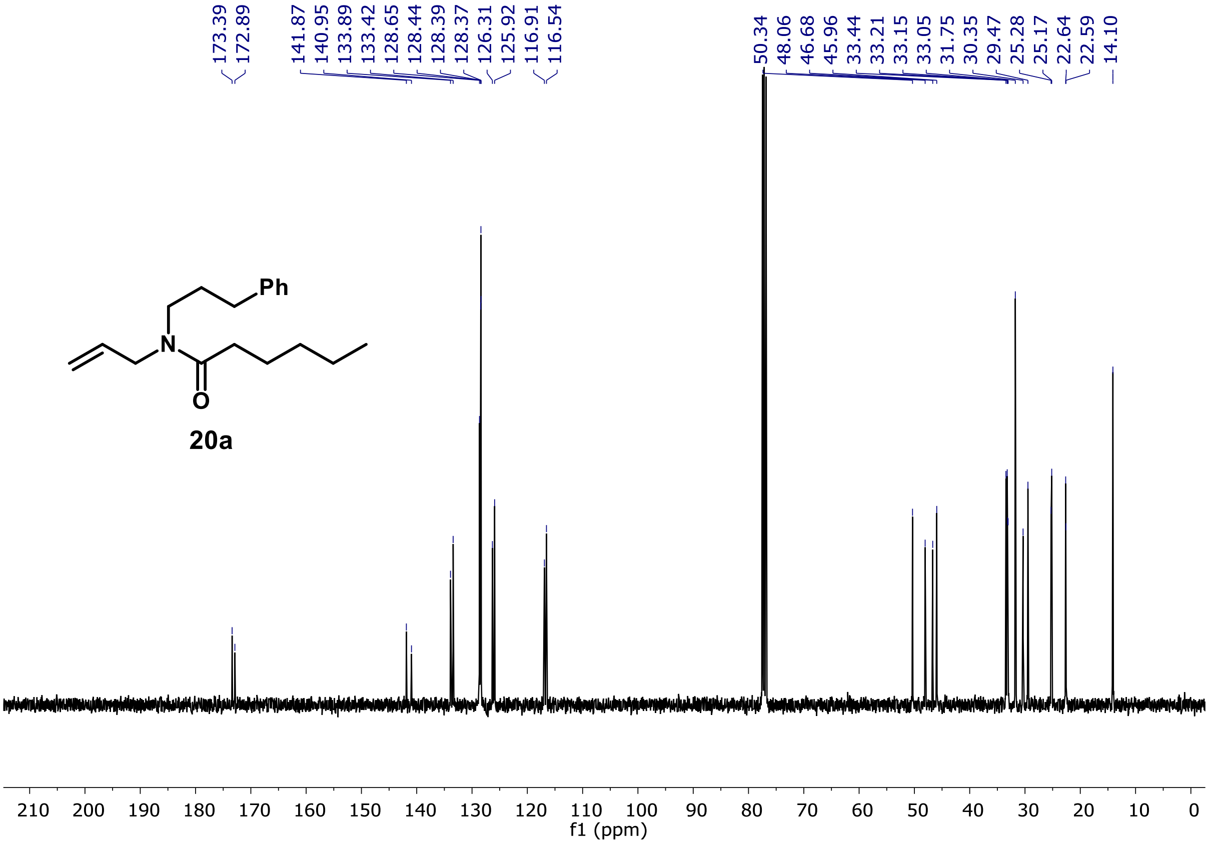

^{1}H (400 MHz, $CDCl_3$)

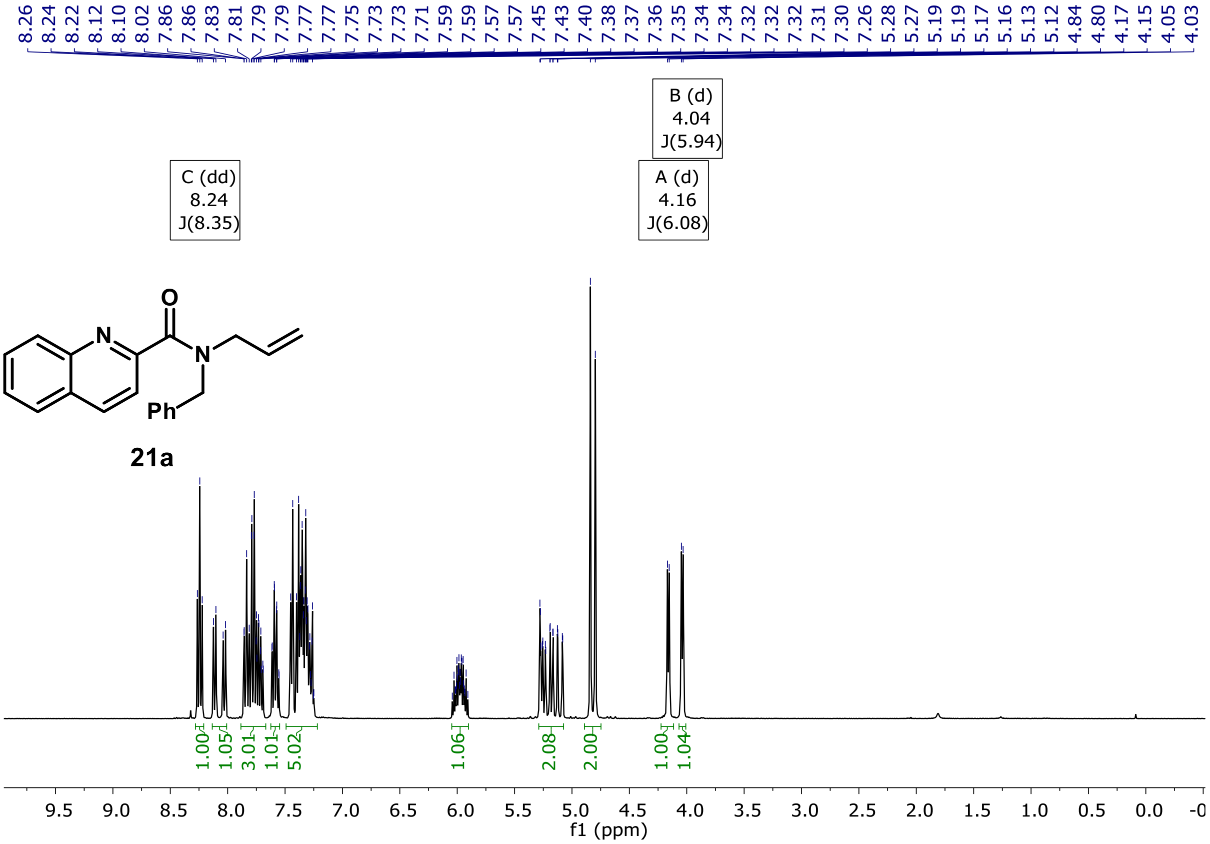

^{13}C (101 MHz, $CDCl_3$)

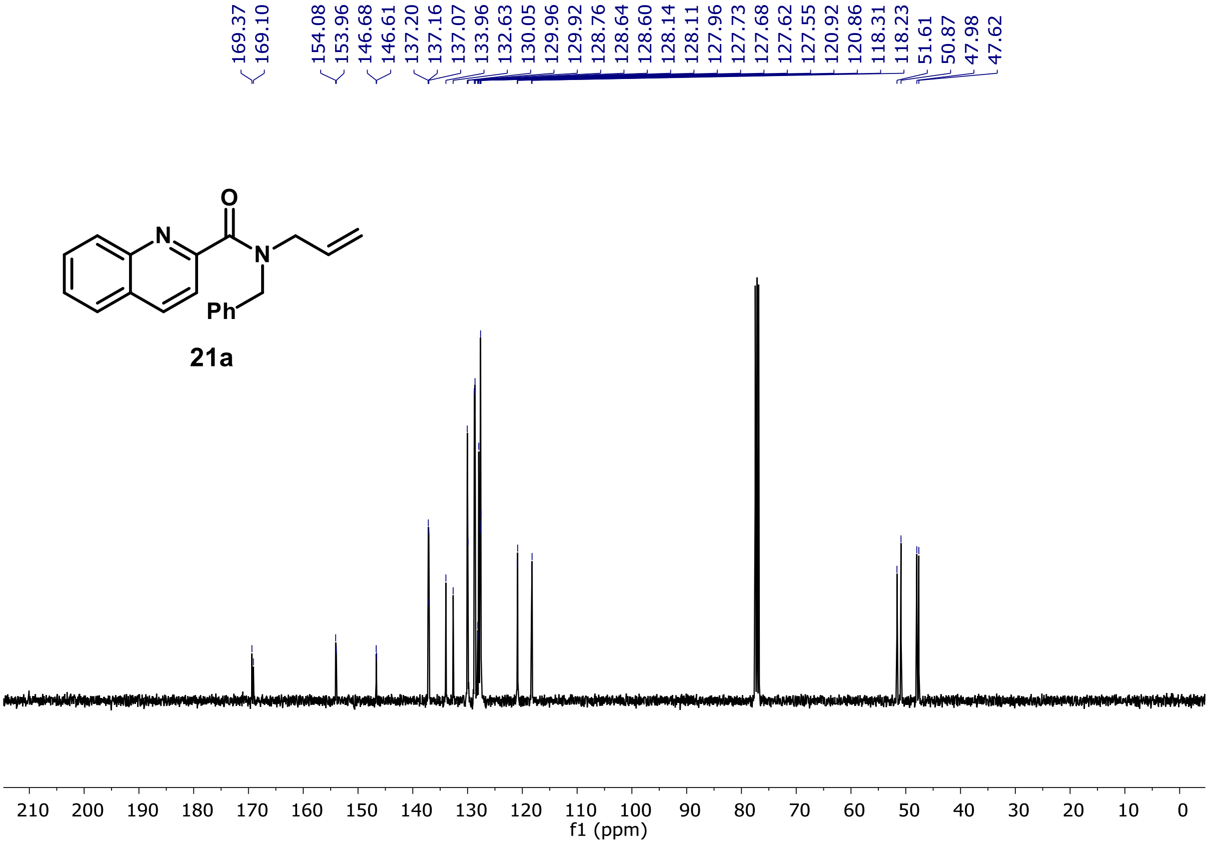

^{1}H (400 MHz, $CDCl_3$)

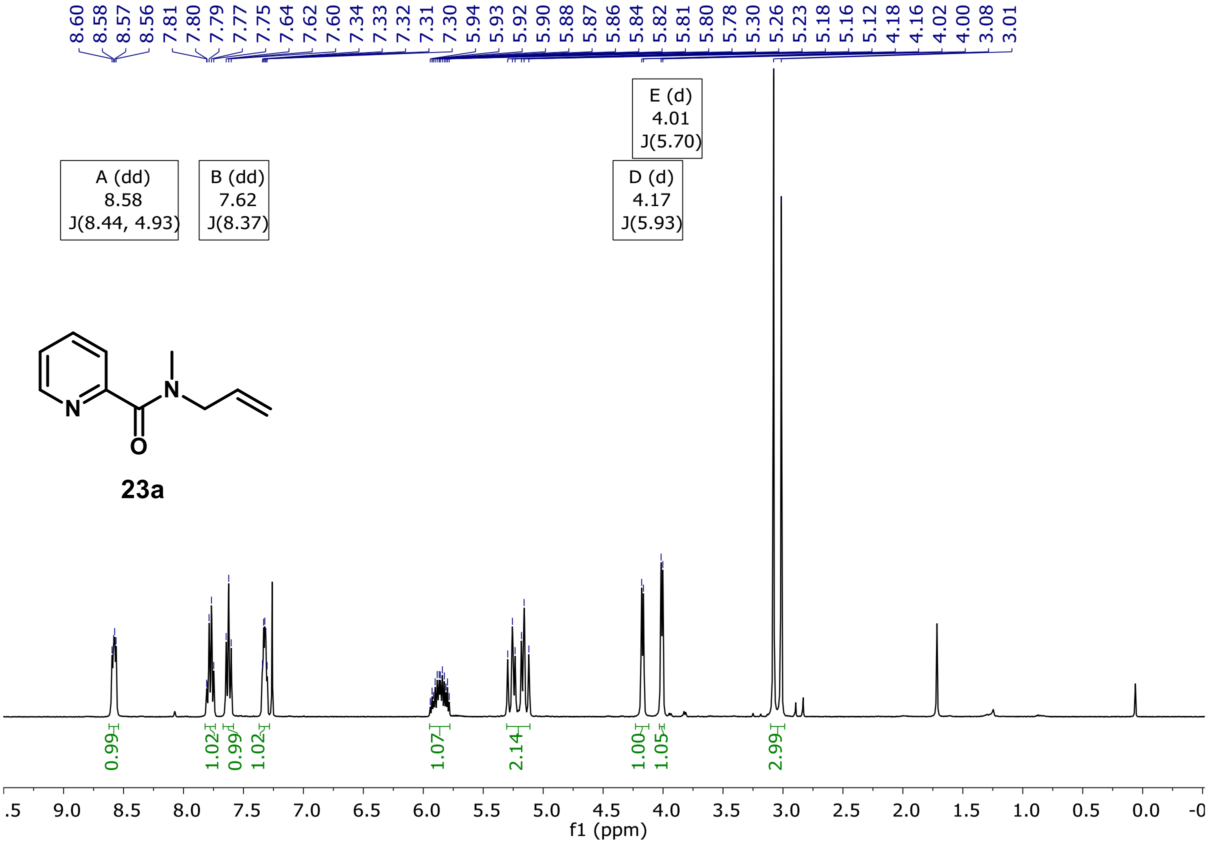

^{13}C (101 MHz, $CDCl_3$)

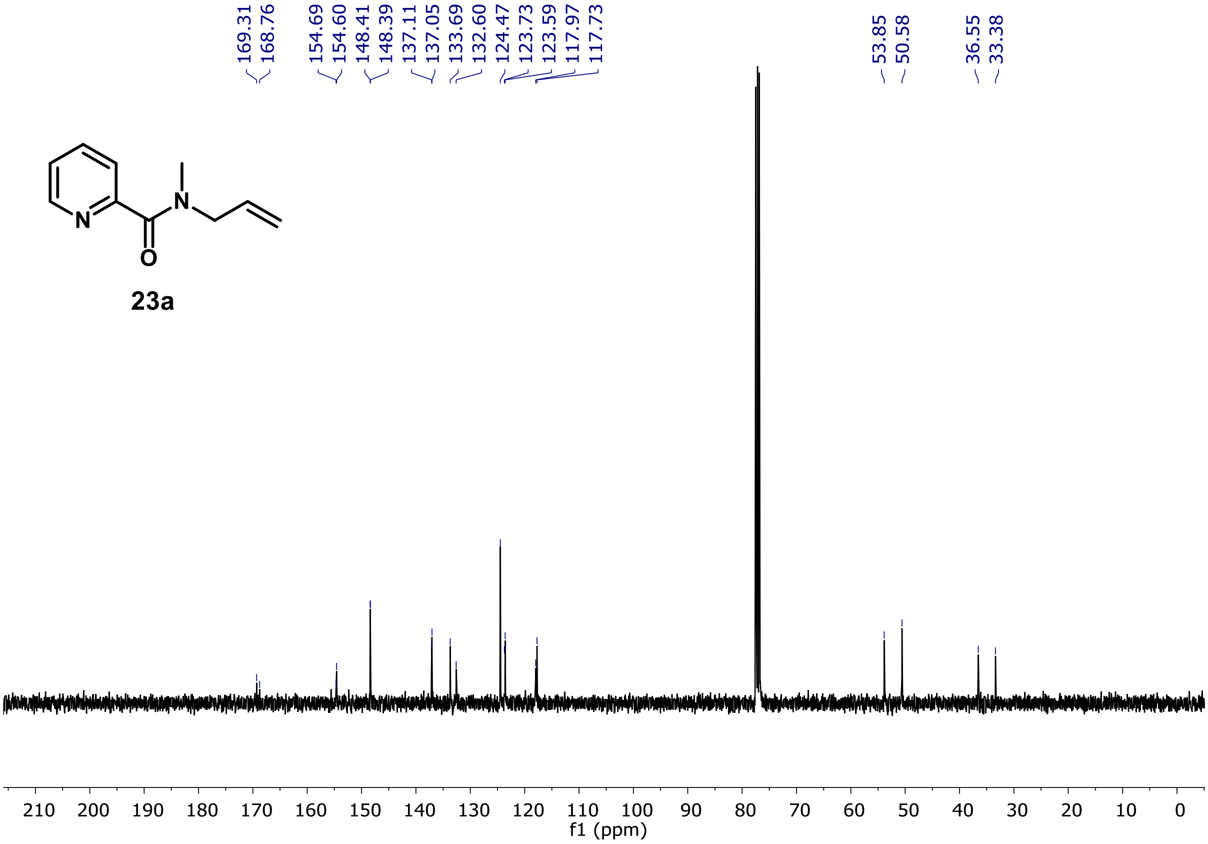

^{1}H (400 MHz, $CDCl_3$)

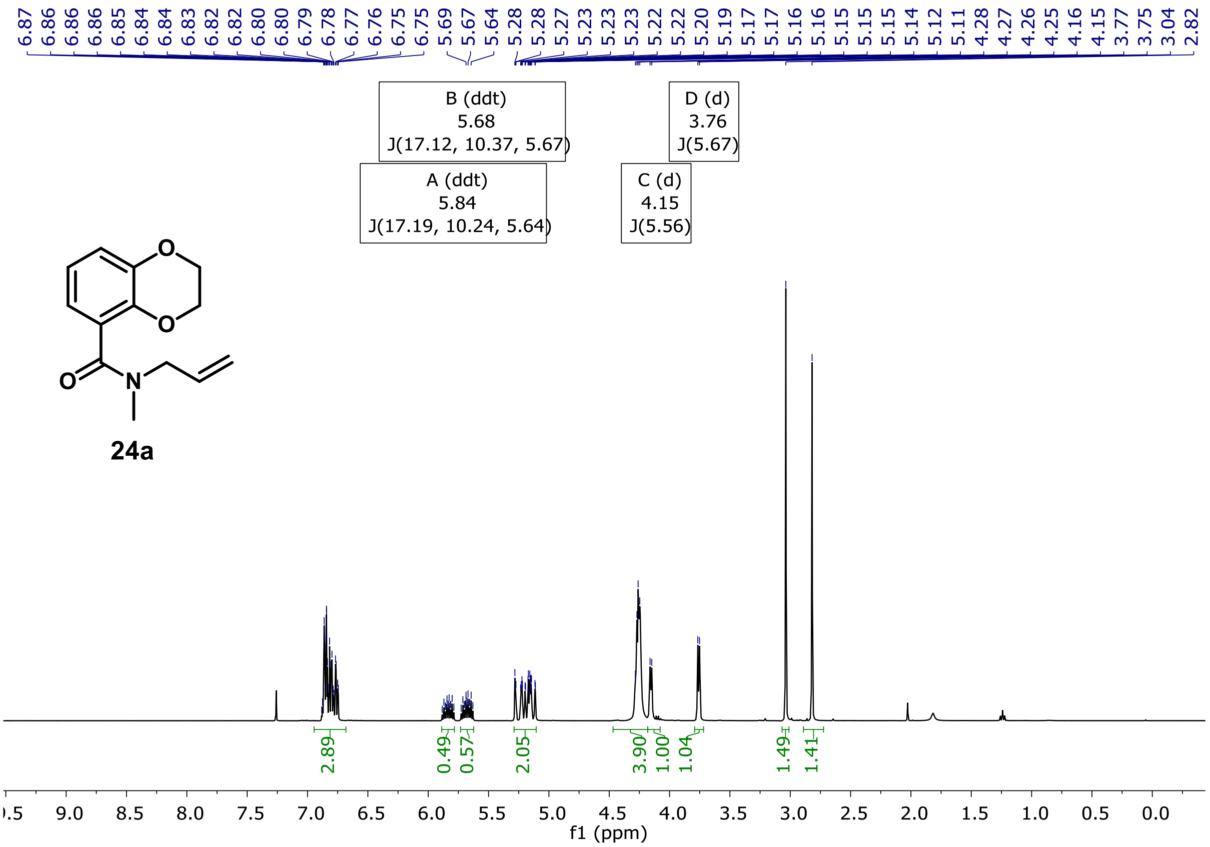

^{13}C (101 MHz, $CDCl_3$)

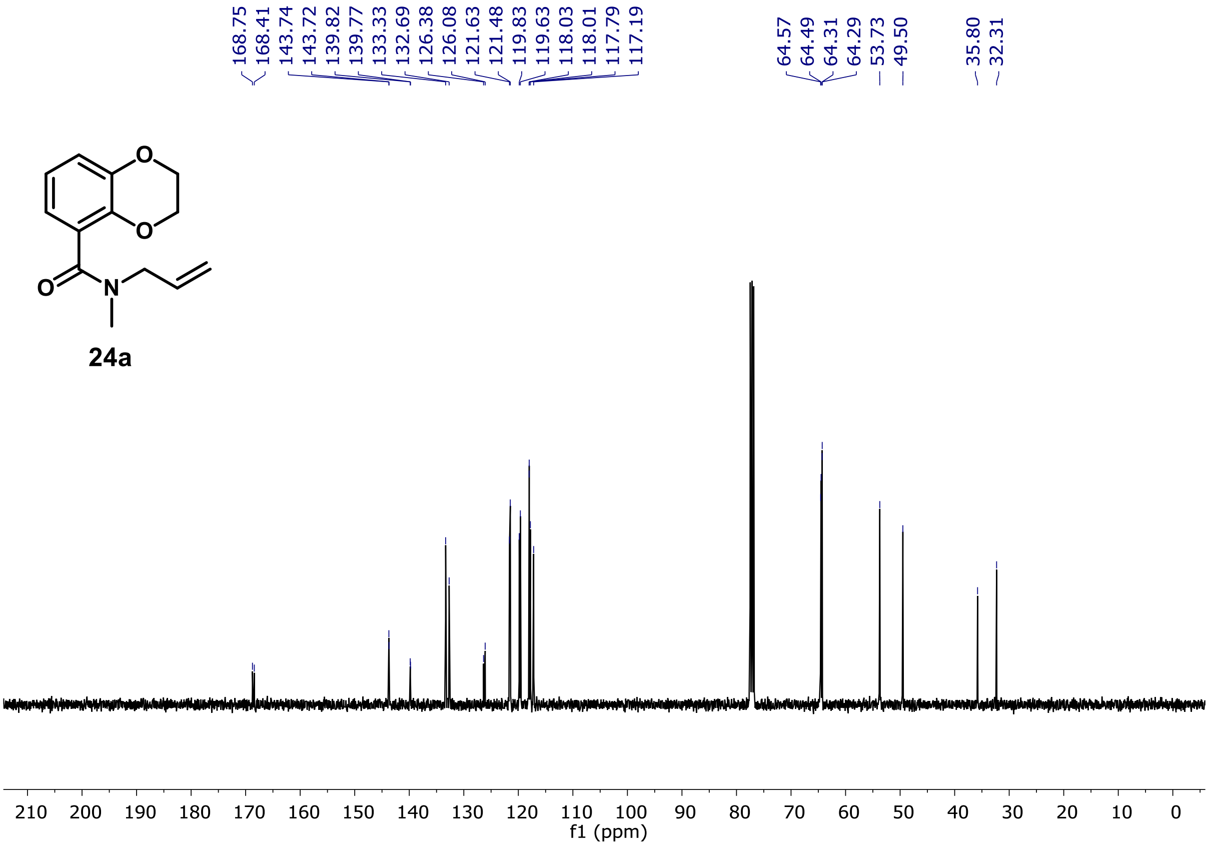

^1H (400 MHz, $CDCl_3$)

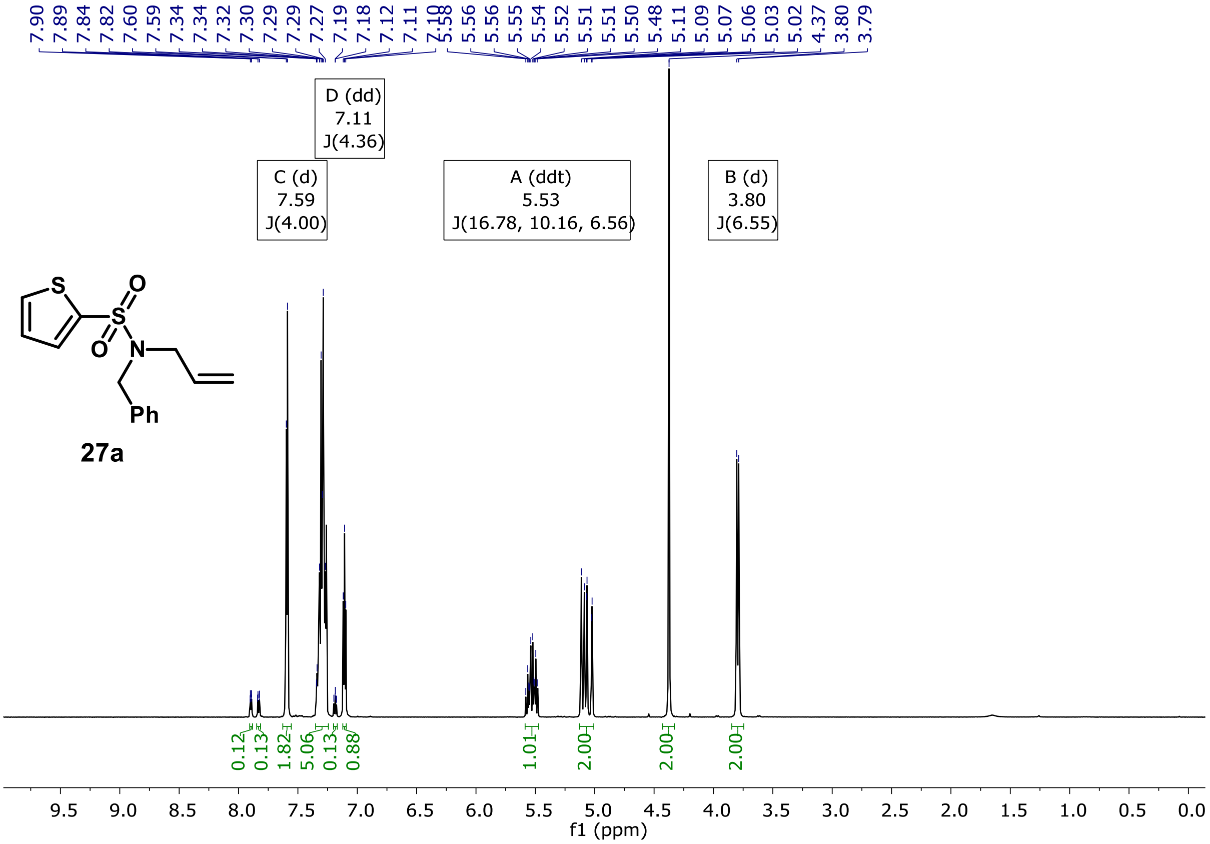

^{13}C (101 MHz, $CDCl_3$)

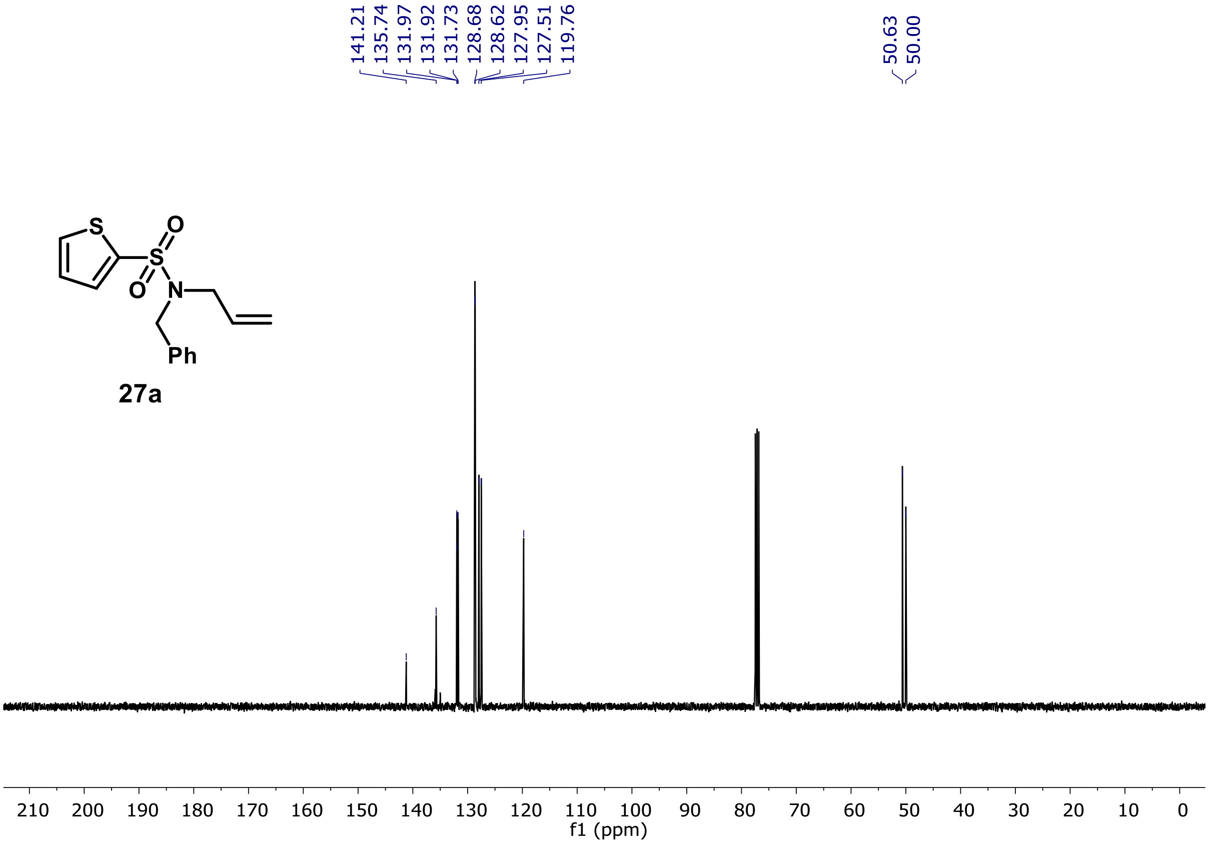

^{1}H (400 MHz, $CDCl_3$)

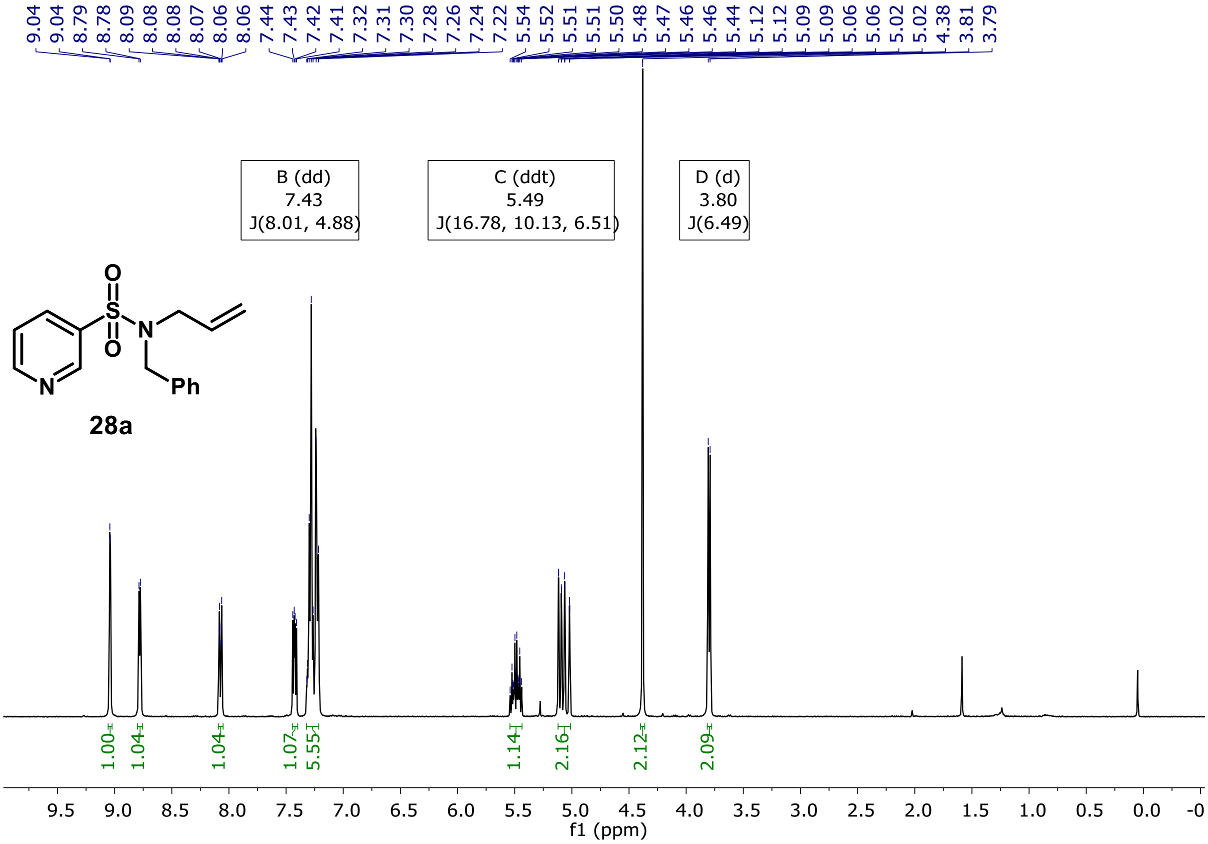

^{13}C (101 MHz, $CDCl_3$)

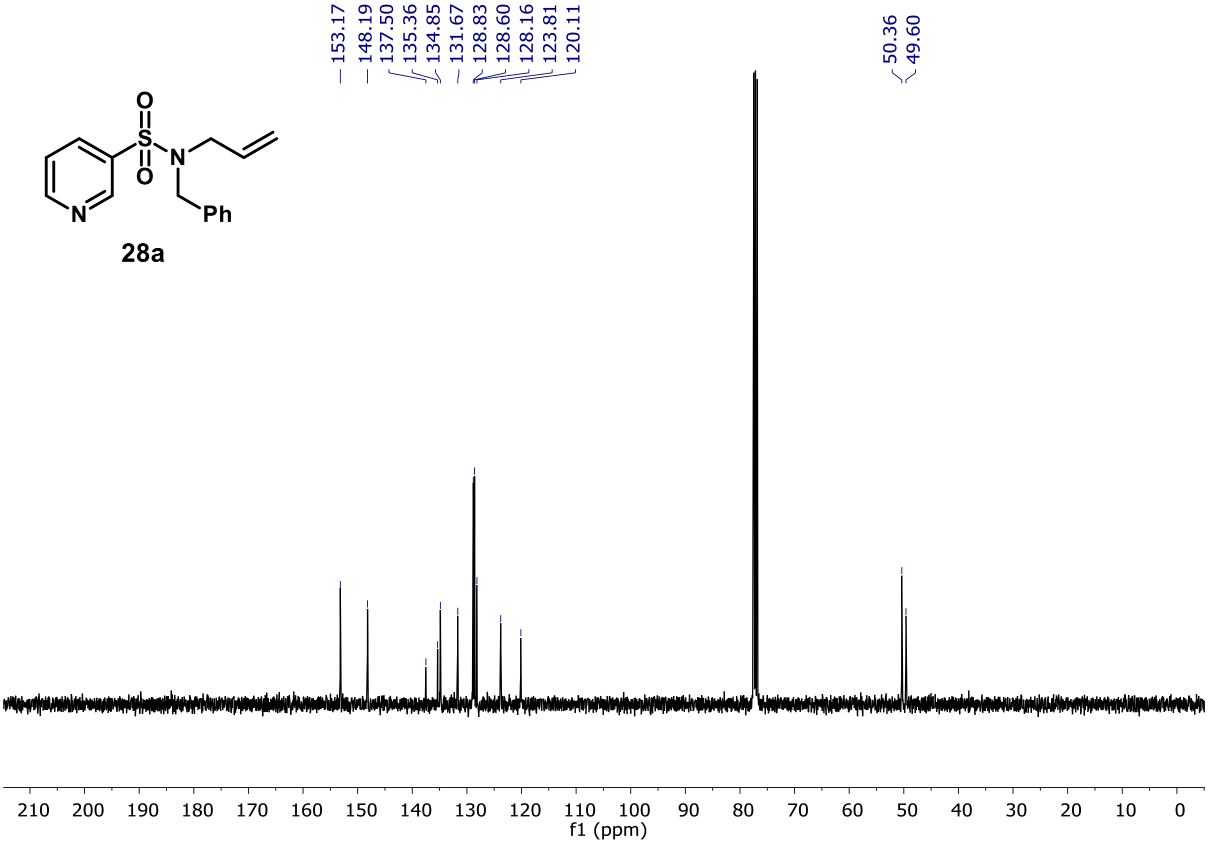

^{1}H (400 MHz, $CDCl_3$)

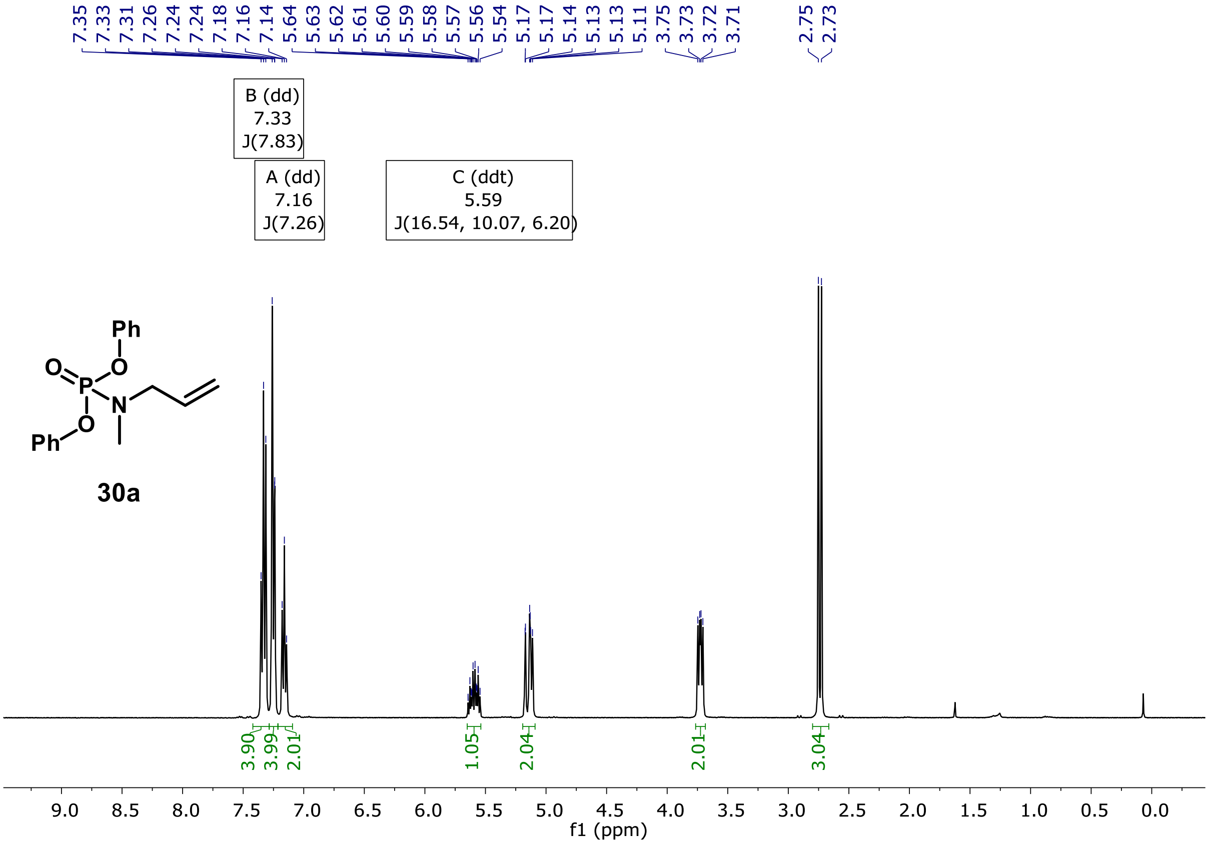

^{13}C (101 MHz, $CDCl_3$)

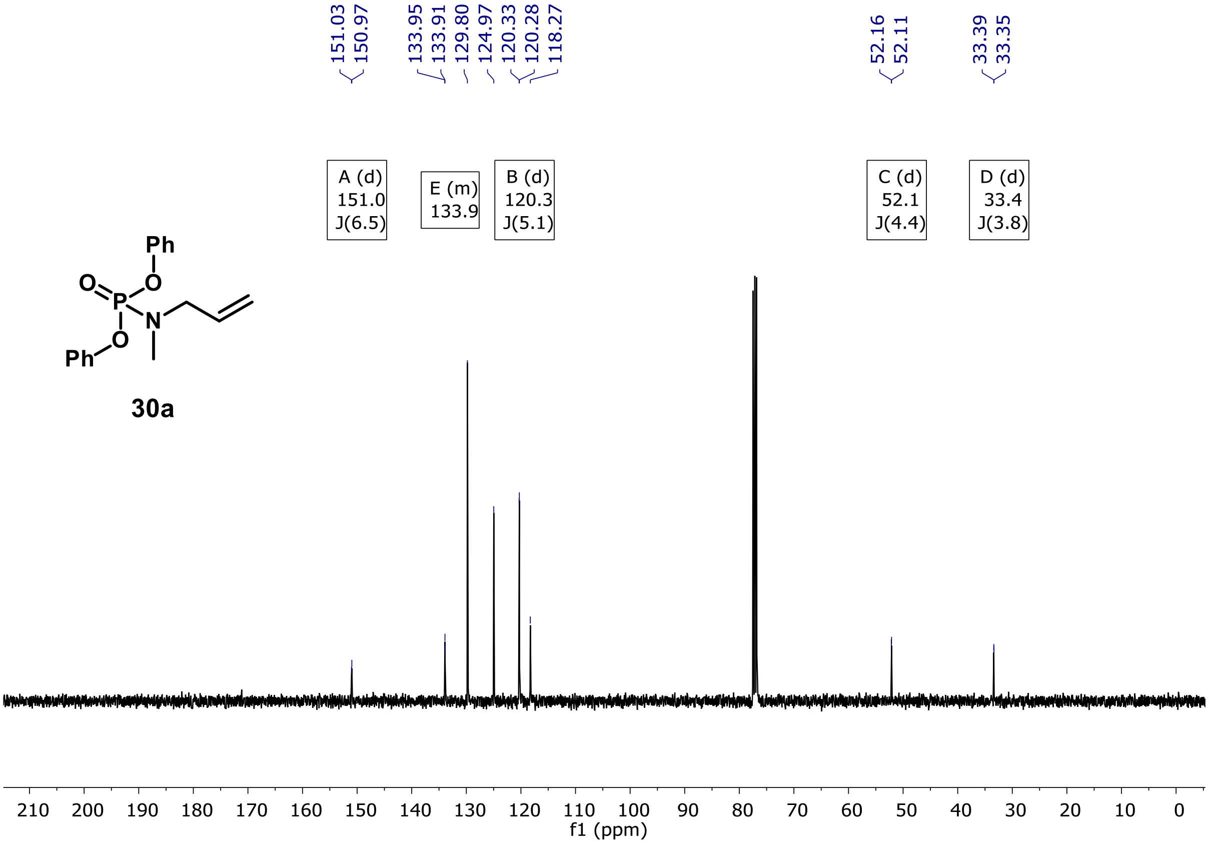

^{1}H (400 MHz, $CDCl_3$)

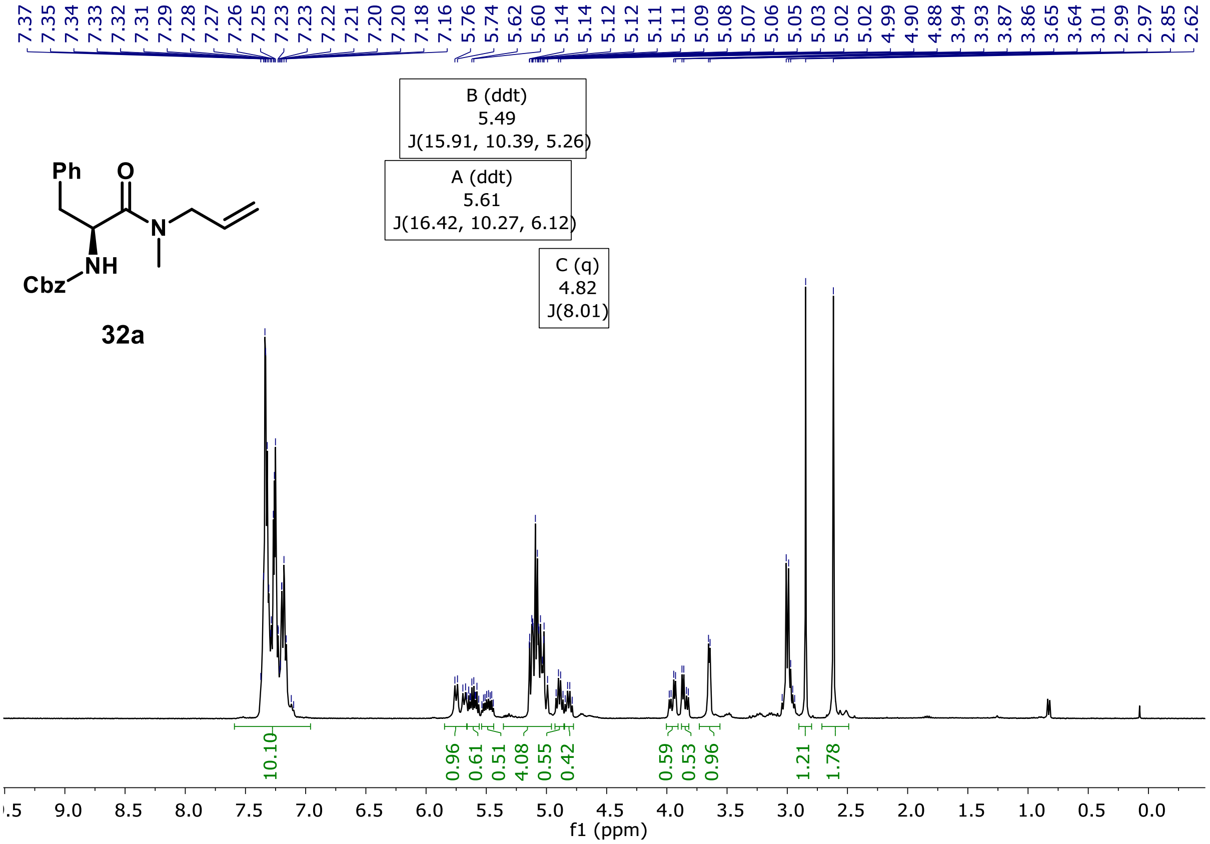

^{13}C (101 MHz, $CDCl_3$)

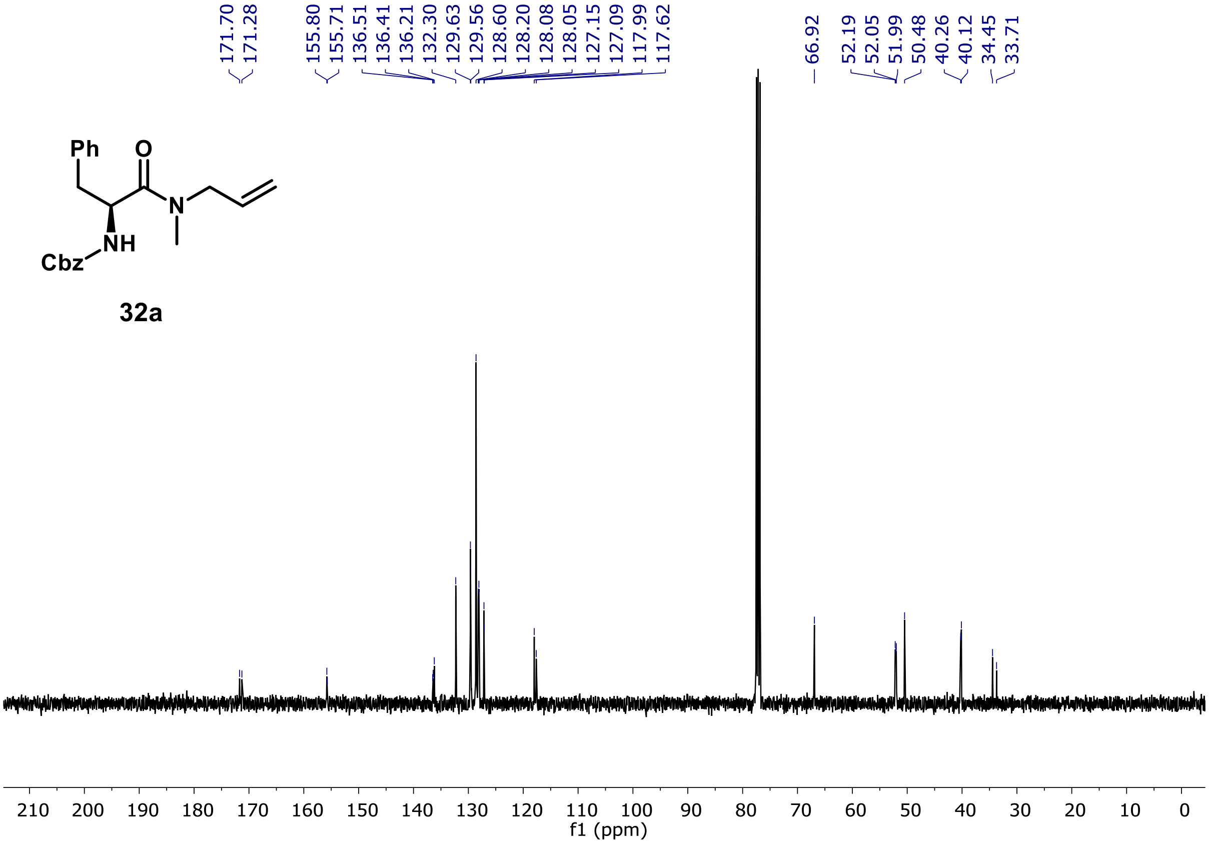

^{1}H (400 MHz, $CDCl_3$)

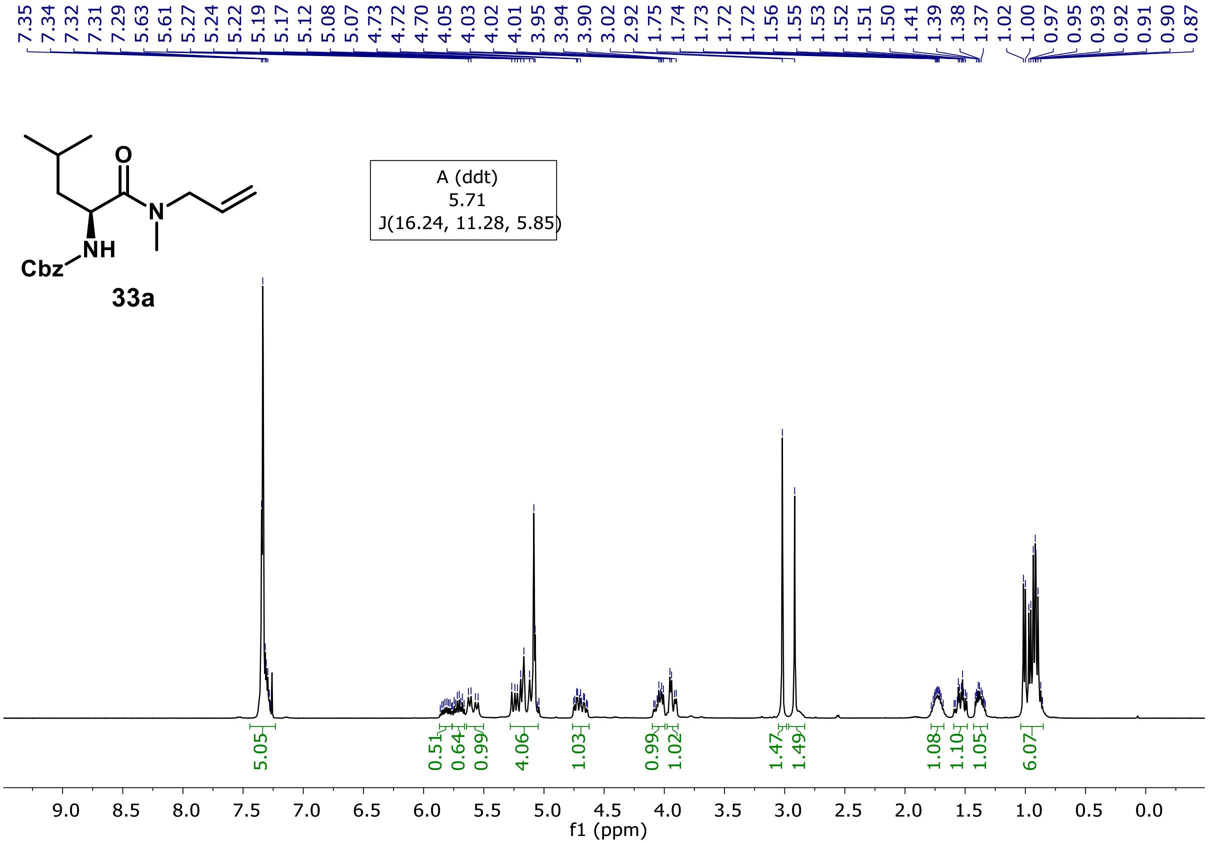

^{13}C (101 MHz, $CDCl_3$)

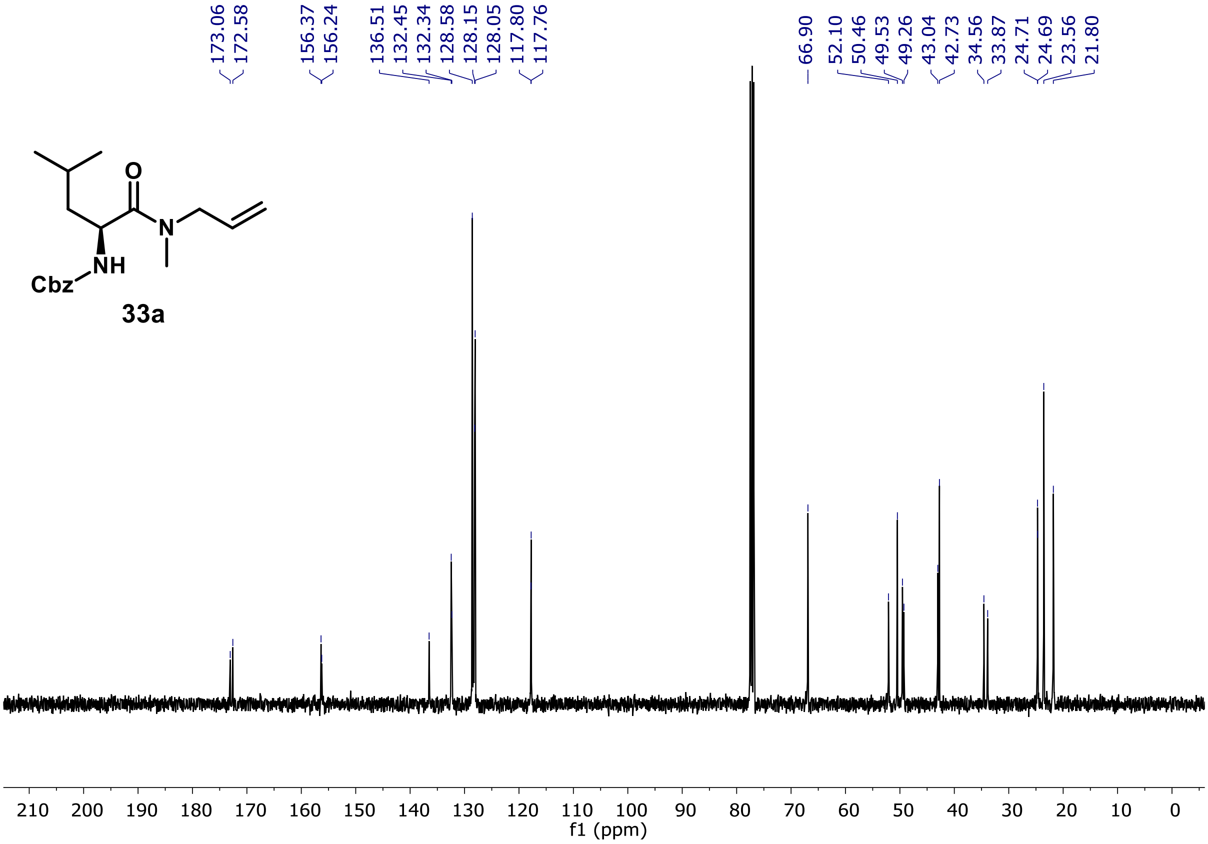

^{1}H (400 MHz, $CDCl_3$)

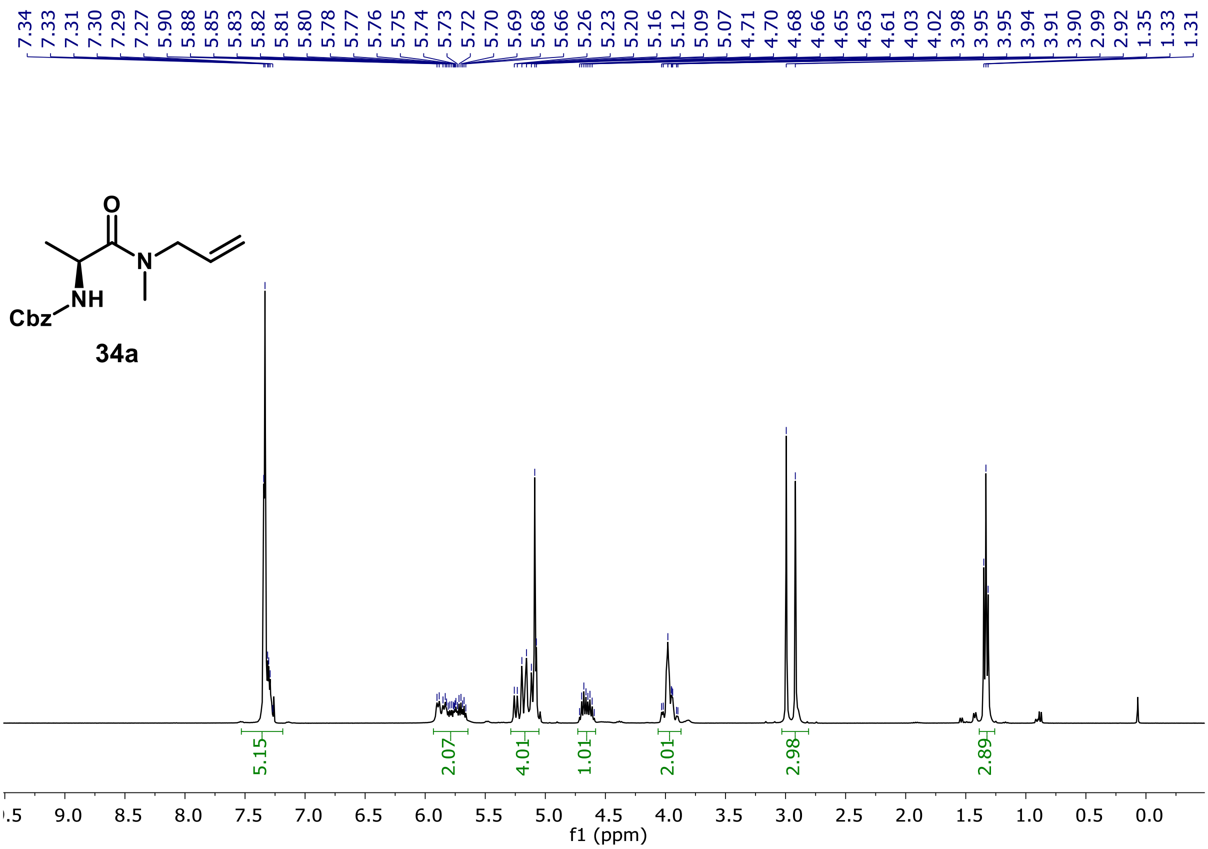

^{13}C (101 MHz, $CDCl_3$)

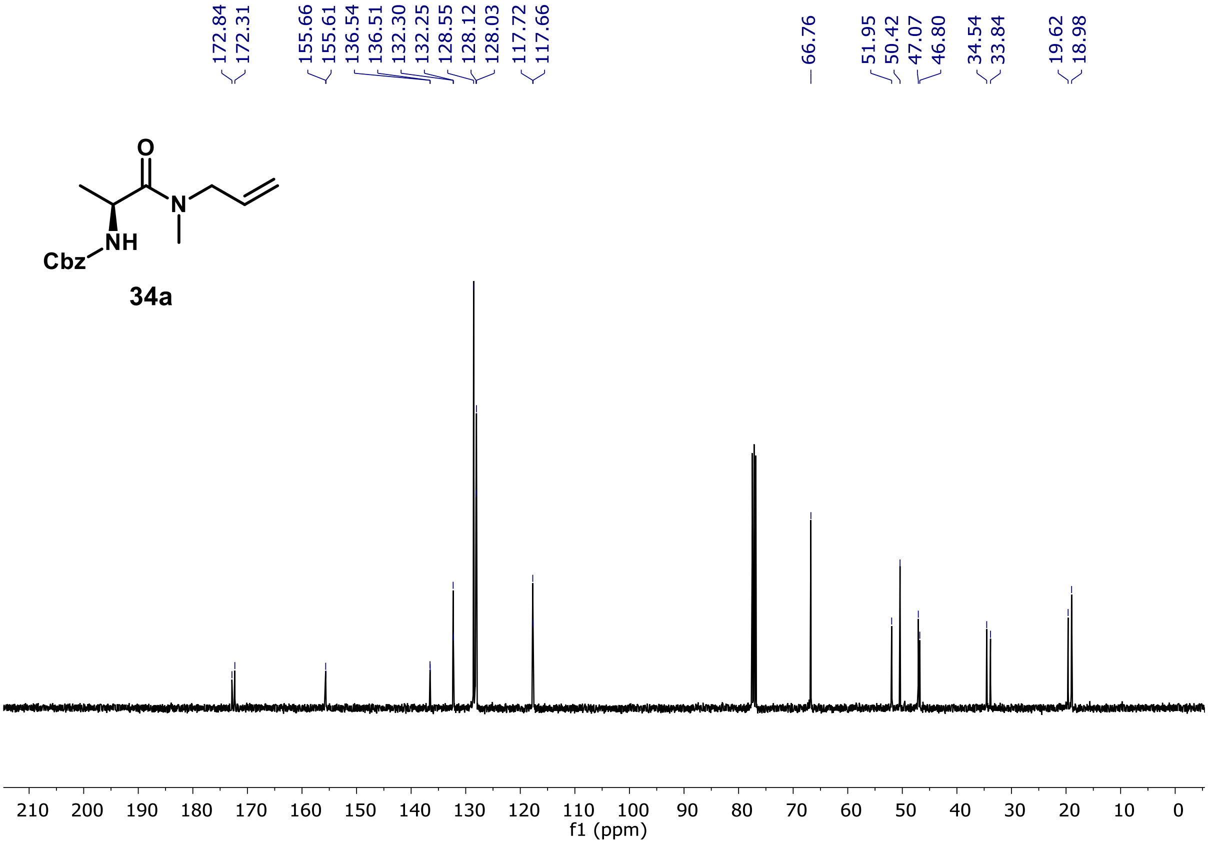

^{1}H (400 MHz, $CDCl_3$)

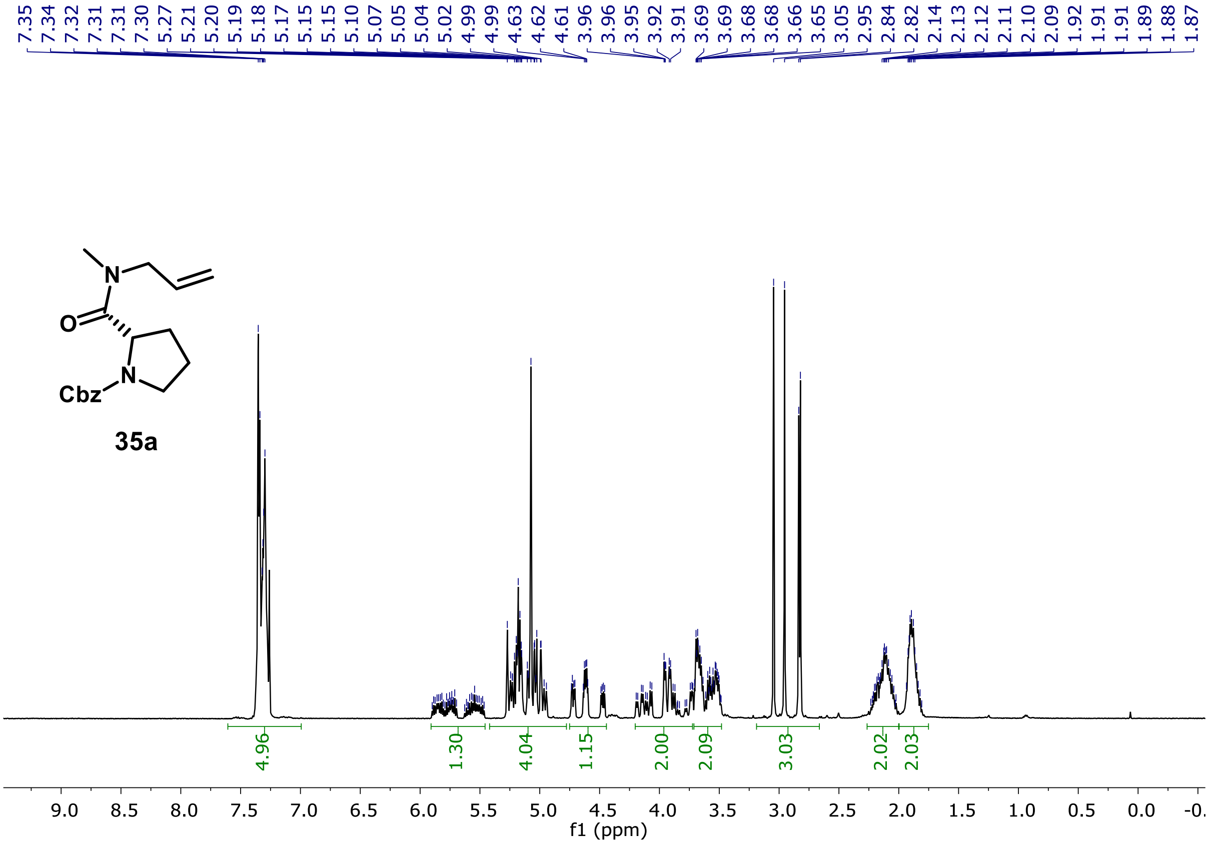

^{13}C (101 MHz, $CDCl_3$)

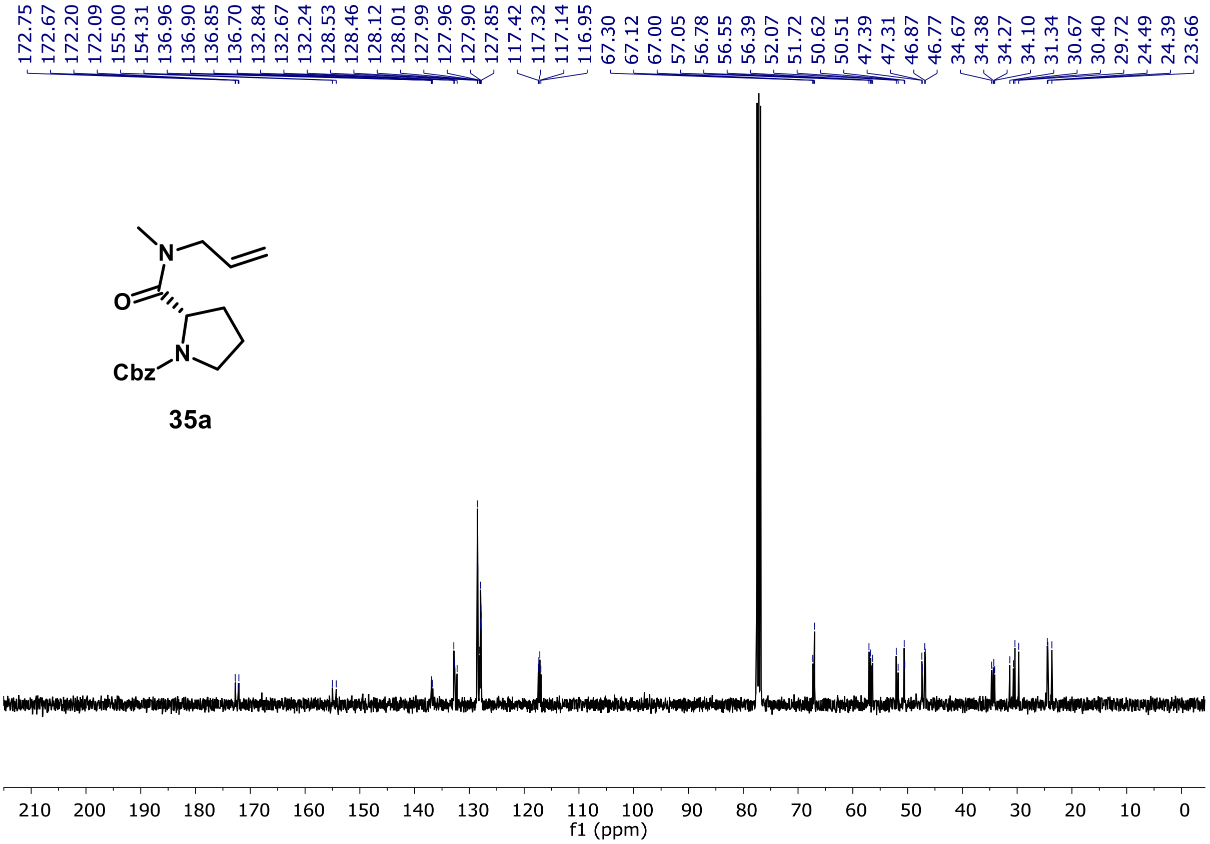

^{1}H (400 MHz, $CDCl_3$)

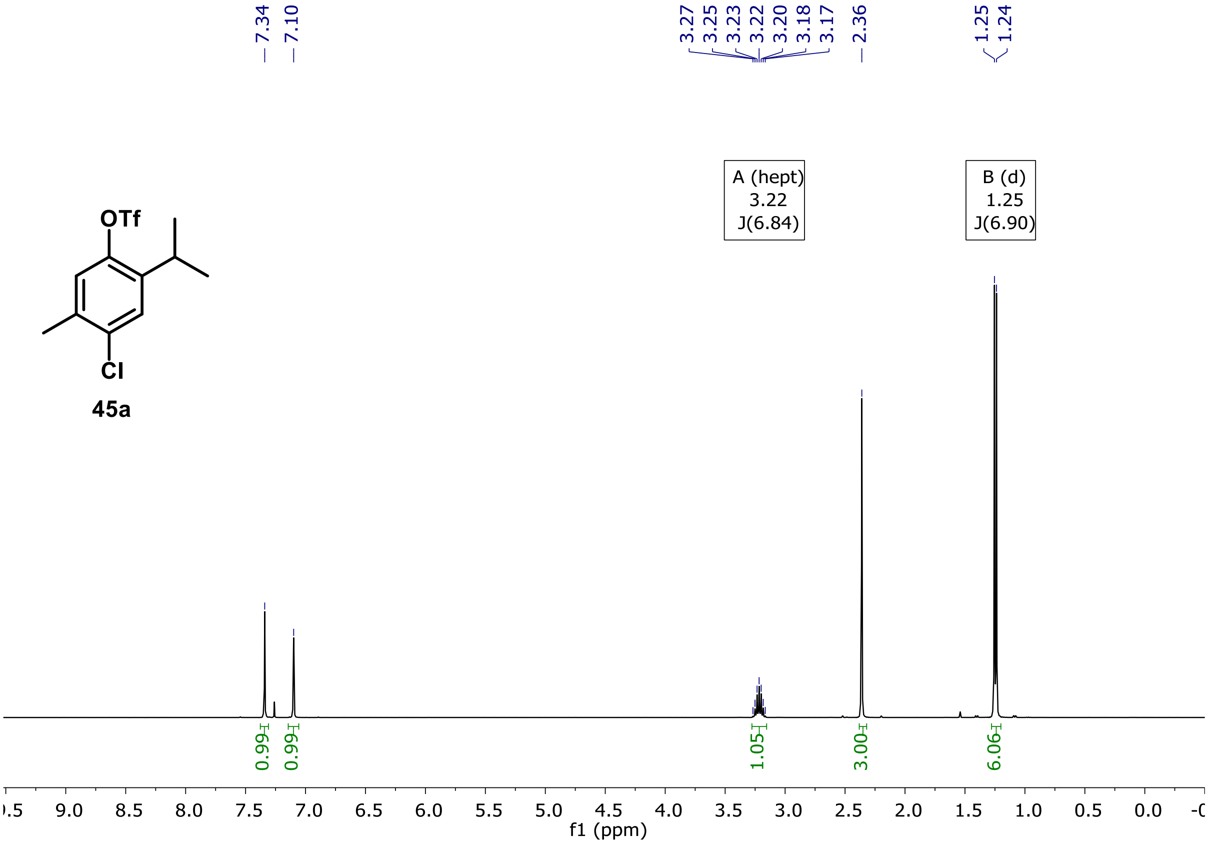

^{13}C (101 MHz, $CDCl_3$)

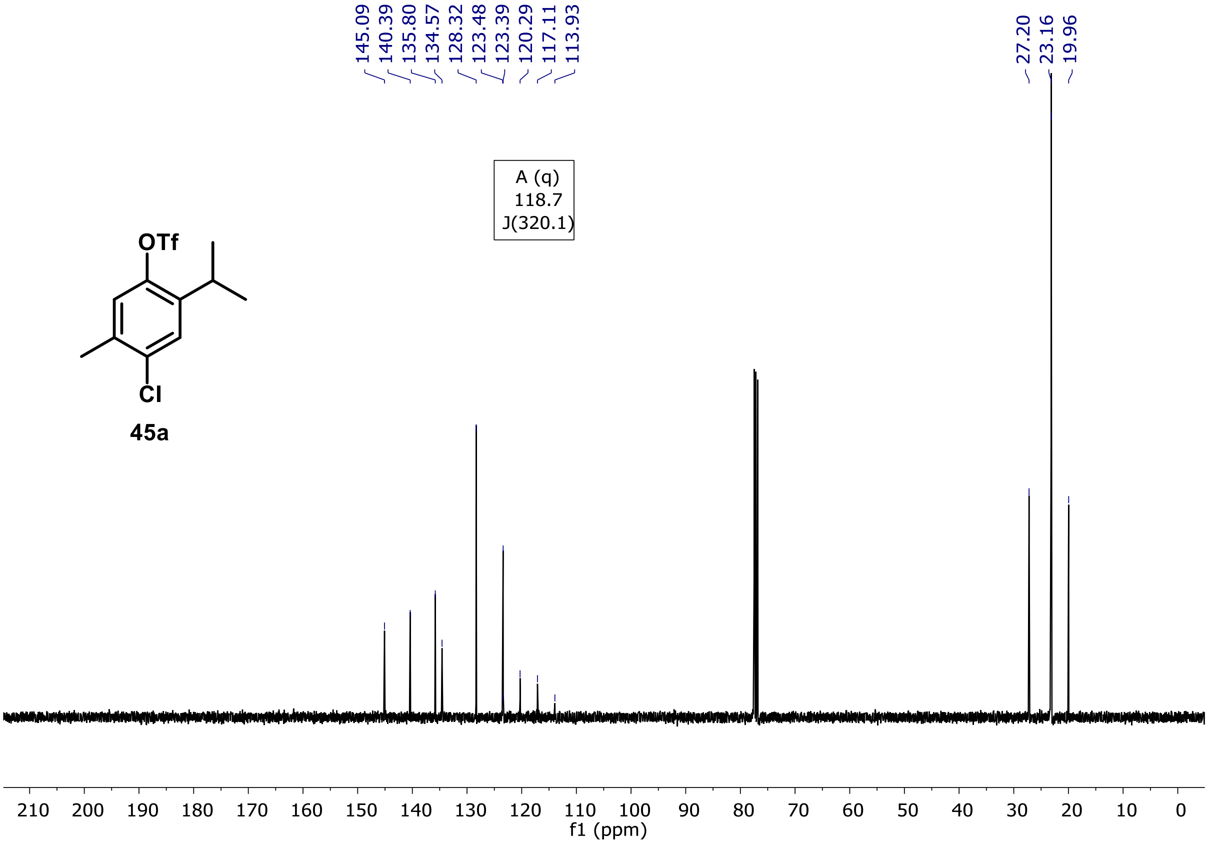

^{19}F (376 MHz, $CDCl_3$)

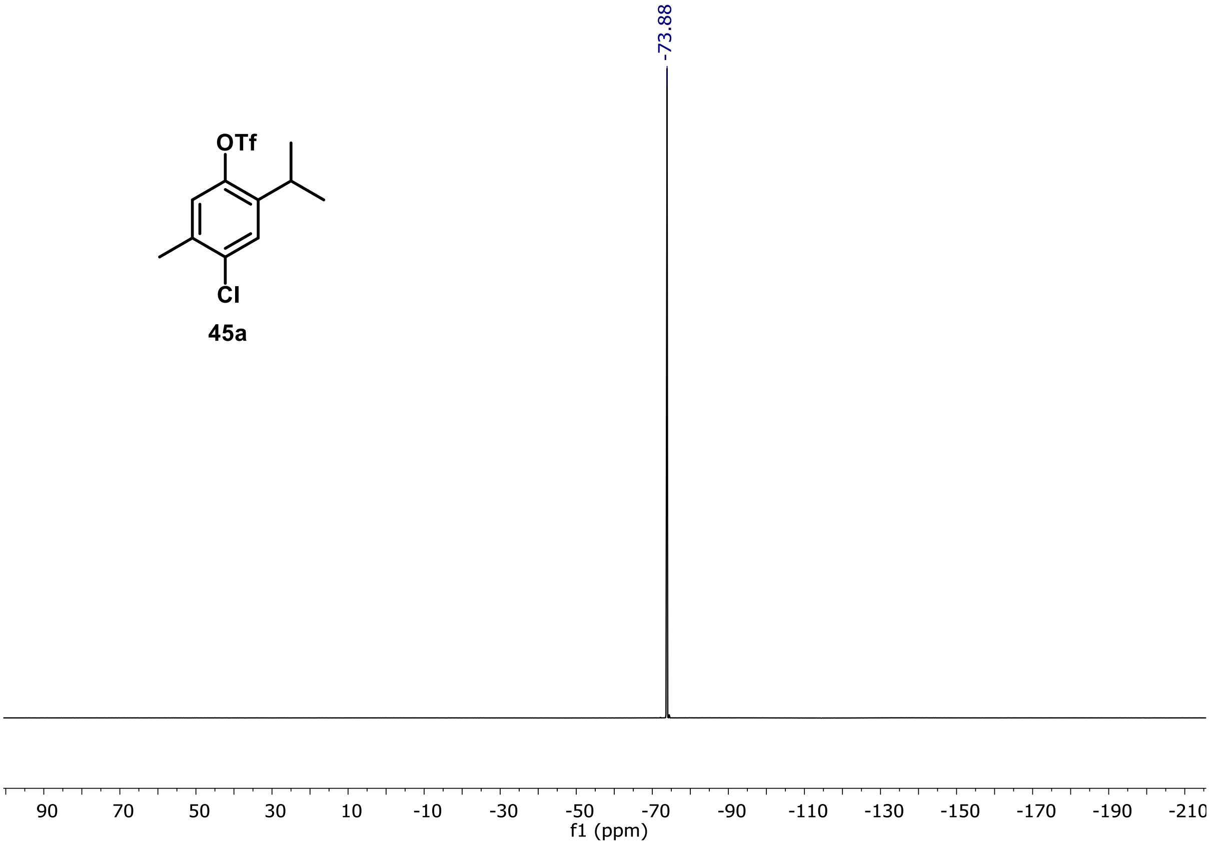

11. References

[1] F. Proutiere, E. Lyngvi, M. Aufiero, I. A. Sanhueza, F. Schoenebeck, *Organometallics* **2014**, *33*, 6879-6884.

[2] N. Ohmura, A. Nakamura, A. Hamasaki, M. Tokunaga, *European Journal of Organic Chemistry* **2008**, *2008*, 5042-5045.

[3] M. S. Azizi, Y. Edder, A. Karim, M. Sauthier, *European Journal of Organic Chemistry* **2016**, *2016*, 3796-3803.

[4] D. Xia, Y. Li, T. Miao, P. Li, L. Wang, *Chem. Commun.* **2016**, *52*, 11559-11562.

[5] R. Shintani, K. Moriya, T. Hayashi, *Chem. Commun.* **2011**, *47*, 3057-3059.

[6] G. Abbiati, E. M. Beccalli, G. Broggini, C. Zoni, *The Journal of Organic Chemistry* **2003**, *68*, 7625-7628.

[7] X.-S. Ning, M.-M. Wang, C.-Z. Yao, X.-M. Chen, Y.-B. Kang, *Organic Letters* **2016**, *18*, 2700-2703.

[8] A. Kapat, T. Sperger, S. Guven, F. Schoenebeck, *Science* **2019**, *363*, 391.

[9] J. Dhineshkumar, K. R. Prabhu, *Organic Letters* **2013**, *15*, 6062-6065.

[10] M. Mendel, I. Kalvet, D. Hupperich, G. Magnin, F. Schoenebeck, *Angew. Chem. Int. Ed.* **2020**, *59*, 2115-2119.

[11] S. T. Keaveney, G. Kundu, F. Schoenebeck, *Angew. Chem. Int. Ed.* **2018**, *57*, 12573-12577.

[12] Y. Xu, X.-Y. Liu, Z.-Q. Wang, L.-F. Tang, *Tetrahedron Letters* **2017**, *58*, 1788-1791.

[13] B. Neugnot, J.-C. Cintrat, B. Rousseau, *Tetrahedron* **2004**, *60*, 3575-3579.

[14] D. Gauthier, A. T. Lindhardt, E. P. K. Olsen, J. Overgaard, T. Skrydstrup, *J. Am. Chem. Soc.* **2010**, *132*, 7998-8009.

[15] B. Alcaide, P. Almendros, J. M. Alonso, *Chem. Eur. J.* **2006**, *12*, 2874-2879.

[16] S. Krompiec, M. Pigulla, W. Szczepankiewicz, T. Bieg, N. Kuznik, K. Leszczynska-Sejda, M. Kubicki, T. Borowiak, *Tetrahedron Lett.* **2001**, *42*, 7095-7098.

[17] M. H. Shaw, W. G. Whittingham, J. F. Bower, *Tetrahedron* **2016**, *72*, 2731-2741.

[18] S. Igueld, M. Baboulène, A. Dicko, M. Montury, *Synthesis* **1989**, *1989*, 200-202.

[19] W. A. L. van Otterlo, E. L. Ngidi, S. Kuzvidza, G. L. Morgans, S. S. Moleele, C. B. de Koning, *Tetrahedron* **2005**, *61*, 9996-10006.

[20] I. R. Hazelden, X. Ma, T. Langer, J. F. Bower, *Angew. Chem. Int. Ed* **2016**, *55*, 11198-11202.

[21] S. N. Patil, S. G. Tilve, *Tetrahedron Letters* **2016**, *57*, 3371-3375.

[22] I. Kalvet, T. Sperger, T. Scattolin, G. Magnin, F. Schoenebeck, *Angew. Chem. Int. Ed.* **2017**, *56*, 7078-7082.

[23] I. D. Hills, G. C. Fu, *J. Am. Chem. Soc.* **2004**, *126*, 13178-13179.

[24] B. Berzina, I. Mutule, E. Suna (Latvian Institute of Organic Synthesis), WO2015063543 (A1), **2015**.

[25] G. M. Sheldrick, *Acta Crystallogr.* **2015**, *A71*, 3-8.

[26] O. V. Dolomanov, L. J. Bourhis, R. J. Gildea, J. A. K. Howard, H. Puschmann, *J. Appl. Crystallogr.* **2009**, *42*, 339-341.

[27] G. M. Sheldrick, *Acta Crystallogr.* **2015**, *C71*, 3-8.
